# Genome wide characterization and expression analysis of *CrRLK1L* gene family in wheat unravels their roles in development and stress-specific responses

**DOI:** 10.3389/fpls.2024.1345774

**Published:** 2024-03-26

**Authors:** Nilesh D. Gawande, Subramanian Sankaranarayanan

**Affiliations:** Department of Biological Sciences and Engineering, Indian Institute of Technology Gandhinagar, Palaj, Gujarat, India

**Keywords:** abiotic stress, biotic stress, CrRLK1L, gene expression, plant reproduction

## Abstract

*Catharanthus roseus receptor-like kinase 1-like* (*CrRLK1L*) genes encode a subfamily of receptor-like kinases (RLK) that regulate diverse processes during plant growth, development, and stress responses. The first CrRLK1L was identified from the *Catharanthus roseus*, commonly known as Madagascar periwinkle. Subsequently, CrRLK1L gene families have been characterized in many plants. The genome of *T. aestivum* encodes 15 *CrRLK1L* genes with 43 paralogous copies, with three homeologs each, except for *-2-*D and *-7-*A, which are absent. Chromosomal localization analysis revealed a markedly uneven distribution of *CrRLK1L* genes across seven different chromosomes, with chromosome 4 housing the highest number of genes, while chromosome 6 lacked any *CrRLK1L* genes. Tissue-specific gene expression analysis revealed distinct expression patterns among the gene family members, with certain members exhibiting increased expression in reproductive tissues. Gene expression analysis in response to various abiotic and biotic stress conditions unveiled differential regulation of gene family members. Cold stress induces CrRLK1Ls *-4-B* and *-15-A* while downregulating *-3-A* and *-7B*. Drought stress upregulates *-9D*, contrasting with the downregulation of *-7D*. *CrRLK1L-15-B* and *-15-D* were highly induced in response to 1 hr of heat, and combined drought and heat stress, whereas *-10-B* is downregulated. Similarly, in response to NaCl stress, only *CrRLK1L1* homeologs were induced. *Fusarium graminearum* and *Claviceps purpurea* inoculation induces homeologs of *CrRLK1L-6* and *-7*. The analysis of *cis*-acting elements in the promoter regions identified elements crucial for plant growth and developmental processes. This comprehensive genome-wide analysis and expression study provides valuable insights into the essential functions of CrRLK1L members in wheat.

## 1 Introduction

The plant receptor-like kinases (RLKs) are transmembrane receptor proteins localized in the cell membrane, constituting a well-studied family of kinases. RLKs are crucial in regulating various intercellular activities and plant growth and developmental processes by sensing numerous extracellular cues (Shiu and Bleecker, 2003). RLKs, comprising an intracellular serine/threonine kinase domain, a transmembrane domain, and a varied extracellular domain, facilitate cellular signaling through interactions with different partners via their transmembrane and juxtamembrane domains. The RLK families are sub-classified based on their N-terminal extracellular domain, determining their ligand specificity (Greeff et al., 2012).

The *Catharanthus roseus* receptor-like kinase 1-like (*CrRLK1L*) gene family belongs to a specific subfamily of RLKs and is characterized by three distinct domains: an extracellular ligand-binding domain, transmembrane domain, and kinase domain. The extracellular domain of CrRLK1Ls has homology with the carbohydrate-binding domain but lacks residues important for carbohydrate-rich ligand binding (Boisson-Dernier et al., 2011). *CrRLK1L* genes were first discovered as novel RLKs in Madagascar periwinkle (Schulze-Muth et al., 1996). *CrRLK1L* gene families have been widely characterized in various plant species, resulting in the identification of diverse members within the gene family.

*CrRLK1L* genes play crucial roles in plant growth, development, and stress responses (Stegmann et al., 2017; Zhang et al., 2020a). In Arabidopsis, six of the 17 subfamily members have been identified as cell growth regulators, and function in cell-cell communication, and cell wall remodeling during vegetative and reproductive development (Lindner et al., 2012). The Arabidopsis CrRLK1Ls have been clustered into ten clades, and their functional characterization has shed light on their involvement in plant reproduction. For instance, Arabidopsis CrRLK1Ls, including At-BUPS1 (AT4G39110.1), At-BUPS2 (AT2G21480.1), At-ANX1 (AT3G04690.1), and At-ANX2 (AT5G28680.2), form a complex that functions in maintaining the pollen tube integrity and preventing premature rupture of the pollen tube before reaching the female gametophyte (Ge et al., 2017). Other CrRLK1L members in Arabidopsis include At-FER (AT3G51550.1), At-HERK1 (AT3G46290.1), and At-ANJEA (AT5G59700.1), which are involved in pollen tube reception by the synergid cells and the prevention of polytubey (Bordeleau et al., 2022). The most versatile member, FERONIA (FER), named after the Etruscan goddess fertility, acts as a receptor for Rapid Alkalization Factors (RALFs) and regulates numerous plant developmental processes as well as stress responses (Duan et al., 2010; Yu et al., 2012; Li et al., 2015a; Zhao et al., 2018). FERONIA and ANJEA are also involved in establishing various pollination barriers through the regulation of reactive oxygen species (Zhang et al., 2021; Huang et al., 2023). FERONIA is additionally involved in mediating pathogen responses, as loss-of-function FER plants exhibit increased resistance to certain bacterial and fungal pathogens (Keinath et al., 2010; Kessler et al., 2010; Masachis et al., 2016). Besides these members, other CrRLK1Ls, like At-MDS1 (AT5G38990.1), At-MDS2 (AT5G39000.1), At-MDS3 (AT5G39020.1), and At-MDS4 (AT5G39030.1), are known to be involved in plant immunity and metal ion stress responses (Richter et al., 2017; Liu et al., 2021).

The common bread wheat, *T. aestivum*, is among the most important cereal crops in the world. Wheat was the first crop to be domesticated and is the primary staple food crop grown globally (Giraldo et al., 2019). Belonging to the Triticeae family, wheat encompasses nearly 300 species, including its closest relatives, *Hordeum vulgare* (barley) and *Secale cerelae* (rye). Allopolyploidation through hybridization with species from the *Aegilops* genus was a breakthrough in the evolution of Triticum species. The divergence between the diploid AA genome species of Triticum, *T. urartu*, and *T. monococcum* occurred less than a million years ago. The first polyploidization event occurred 0.5 million years ago between *T. urartu* (AA genome) and *Aegilops speltoides* (SS genome), which led to the origin of two species, namely, *T. turgidum* (AABB genome) and *T. timopheevii* (AAGG genome). Approximately 10,000 years ago, the second hybridization event occurred between *T. turgidum* (AABB genome) and the wild wheat species *Aegilops tauschii* (DD genome), which gave rise to *T. aestivum* (AABBDD genome) (Matsuoka, 2011).

Wheat occupies the largest total harvested area among cereal crops, yet its productivity remains the lowest (Abhinandan et al., 2018). The detrimental impact of various abiotic and biotic stresses on wheat plants throughout different growth stages results in significant production losses. Therefore, it is crucial to comprehend the effects of these stresses to drive advancements in wheat improvement programs. The availability of complete genome sequences, transcriptomics data, and the utilization of biotechnological approaches to unravel gene functions can open up new avenues for enhancing crop improvement. Previous genome wide analysis of the *CrRLK1L* gene family has been carried out in various plant species, including *Arabidopsis thaliana* (L.), *Oryza sativa L., Solanum lycopersicum, Nicotiana benthamiana*, and *Gossypium raimondii*. The rice genome has 16, the tomato has 24, *Nicotiana benthamiana* has 31, and the diploid cotton species *G. raimondii* has 44 *CrRLK1L* genes (Lindner et al., 2012; Nguyen et al., 2015; Niu et al., 2016; Rao et al., 2021; Ma et al., 2023). These studies prompted us to further research CrRLK1L gene families in wheat (*T. aestivum*) to unravel their role in plant growth, development, and stress responses. In this study, we analyzed conserved domains, gene structure, and functional motif analysis of CrRLK1L gene family members, along with inferring their evolutionary relationship with Arabidopsis and other monocot species. Additionally, we examined tissue-specific gene expression patterns and the changes in gene expression in response to abiotic and biotic factors using available transcriptome datasets. This research aims to investigate the potential roles of Ta*-CrRLK1L* genes in plant reproduction and abiotic and biotic stress responses, providing a valuable resource for functional characterization and crop improvement.

## 2 Materials and methods

### 2.1 Collection of sequences for *CrRLK1L* gene families in *Triticum aestivum*

Full-length coding and protein sequences for the 16 *CrRLK1L* from rice were retrieved from the Rice Genome Annotation Project database (http://rice.plantbiology.msu.edu/). *CrRLK1L* homologs in wheat, hereafter denoted as Ta*-CrRLK1L*, were searched by using rice FL coding sequences in the NR database at NCBI (https://www.ncbi.nlm.nih.gov/) in *Triticum aestivum*. Sequences with high-scoring hits and maximum similarity with rice *CrRLK1Ls* were collected, and duplicates were eliminated. The NR sequences were used to search for respective A, B, and D homeologs in the Ensembl plants database (https://plants.ensembl.org/index.html), and genomic, CDS, and protein sequences for Ta-CrRLK1L with high-scoring hits and more than 95% identity were collected. Transcript variants that cover the full-length coding sequences were selected. Ta*-CrRLK1L* nucleotide coding sequences from Ensembl plants were used to search against the TSA and EST databases at NCBI for further sequence confirmation. The start codon position in the wheat genome for each Ta*-CrRLK1L* homeolog was determined by a blastn search of coding regions in the International Wheat Genome Sequencing Consortium (IWGSC) RefSeq v2.1 genome assembly at the WHEAT URGI database (https://wheat-urgi.versailles.inra.fr/). The physical properties, like the molecular weight of the protein and isoelectric point (pI), were calculated using the Expasy Compute pI/MW tool (https://web.expasy.org/compute_pi/). Subcellular localization for the proteins was predicted by WoLF PSORT (https://wolfpsort.hgc.jp/).

### 2.2 Compilation of gene sequences for *CrRLK1L* from other plant species

Protein sequences for genes encoding CrRLK1L for closely related species like *Aegilops tauschii* and *Hordeum vulgare* were retrieved from the NCBI database using balstp search with wheat protein sequences as queries. Protein sequences for *Brachypodium distachyon* were collected from Ensembl plants using *T. aestivum* nucleotide coding sequences as query, while sequences for Arabidopsis CrRLK1L gene families were retrieved from the TAIR database (https://www.arabidopsis.org/), and rice sequences were retrieved from the Rice Genome Annotation Project database (http://rice.plantbiology.msu.edu/). Sequences that share the highest sequence identity with wheat CrRLK1Ls were selected. E value cut-off of zero was used.

### 2.3 Conserved domain and phylogenetic analysis

Conserved domains for the Ta-CrRLK1L proteins were confirmed by the Simple Modular Architecture Research Tool (SMART) (http://smart.embl-heidelberg.de/) and Batch Conserved Domain (CD)-Search tool (https://www.ncbi.nlm.nih.gov/Structure/cdd/wrpsb.cgi) at NCBI, and active sites in the Ta-CrRLK1L domains were predicted by PROISTE (http://prosite.expasy.org/). Multiple sequence alignment for Ta-CrRLK1Ls was carried out by ClusatlW, and conserved domain features and active sites were visualized using the Jalview program (https://www.jalview.org/). For phylogenetic analysis, the multiple sequence alignment for protein sequences was carried out by ClustalW (https://www.ebi.ac.uk/Tools/msa/clustalo/), and the evolutionary relationships for CrRLK1L in *T. aestivum* and other five species, namely *Ae. tauschii*, *A. thaliana*, *B. distachyon*, *H. vulgare*, and *O. sativa*, were inferred by analyzing full-length amino acid sequences in MEGA11 (Tamura et al., 2021). Ta*-CrRLK1L* A homeolog copies were used in the analysis, except for Ta*-CrRLK1L7*, for which D copy was used. In total, eighty-eight CrRLK1L protein sequences were used in the analysis. Sequences were aligned by ClustalW with default parameters, and a phylogenetic tree was constructed using the Jukes-Cantor model by the Neighbour-joining method and 1000 bootstrap iterations. The phylogenetic tree was visualized by Tree of Life (iTOL) (https://itol.embl.de/login.cgi).

### 2.4 Gene duplication, chromosomal location, gene structure prediction, and motif analysis

Gene duplication analysis for the Ta-*CrRLK1L* gene families was conducted using cut-off criteria that the sequence alignment had >80% coverage of that of the longer gene, and the aligned region had the identity of >80% at the nucleotide level among the gene pairs (Wang et al., 2016). The Ka/Ks (nonsynonymous substitution rate/synonymous substitution rate) ratio, which determines the selection pressure on the duplicated genes, was calculated for the paralogous gene pairs using TBtools (Chen et al., 2020). Gene duplications were illustrated using circos using shinyCircos-V2.0 (https://venyao.xyz/shinycircos/) (Wang et al., 2023). Chromosome length for each copy in wheat was collected from the Ensembl Plants database, and start and end positions for Ta*-CrRLK1L* genes were determined from the (IWGSC) RefSeq v2.1 genome assembly. The position of the Ta*-CrRLK1L* genes on the chromosomes was represented using MG2C v2.1 (http://mg2c.iask.in/mg2c_v2.1/) (Chao et al., 2021). Intron/exon junctions were determined by comparing Ta*-CrRLK1L c*DNA sequences from the NR database at NCBI with genomic sequences from Ensembl by using Splign (https://www.ncbi.nlm.nih.gov/sutils/splign/splign.cgi?textpage=online&level=form), and the intron/exon junctions were visualized by Gene Structure Display Server 2.0 (GSDS) (http://gsds.gao-lab.org/). Conserved motifs in CrRLK1L proteins were predicted using Multiple Em for Motif Elicitation (MEME) Suite 5.5.2 (http://meme-suite.org/tools/meme) with ten motifs as the number of motifs parameter and the GenomeNet Database (https://www.genome.jp/tools/motif/).

### 2.5 Cis-acting elements prediction

The nucleotide sequences from the upstream 2 Kb regions of the start codons of Ta-*CrRLK1L* genes were retrieved from the Ensembl Plant database and analyzed for cis-acting elements. Cis-acting elements were determined by using PlantCARE (https://bioinformatics.psb.ugent.be/webtools/plantcare/html/).

### 2.6 Gene expression and co-expression analysis of *CrRLK1L* gene families

Tissue-specific gene expression and gene expression in response to stress conditions for Ta-*CrRLK1L* gene families were analyzed using *T. aestivum* transcriptome datasets available at SRA (Sequence Read Achieve) repositories in the NCBI database (https://www.ncbi.nlm.nih.gov/sra). To determine the tissue-specific relative gene expression levels, the transcriptome dataset of seventy-one tissues of wheat cultivar Azhurnaya, available at eFP Browser (http://bar.utoronto.ca/efp_wheat/cgi-bin/efpWeb.cgi), was used (Ramírez-González et al., 2018). Additionally, the gene expression levels in the different tissue types were analyzed from the tissue-specific expression data from BCS cv-1 Development in Wheatomics 1.0 database (http://wheatomics.sdau.edu.cn/expression/wheat.html).The relative gene expression levels in five tissue types, namely, inflorescence, leaf at the whole plant seed formation stage, root at the cotyledon emergence stage, seed at the fruit ripening stage, and stem, were analyzed from the transcriptome datasets available at SRA database at NCBI (Pingault et al., 2015). Tissue-specific expression in the stamen and pistil tissues was analyzed by using BioProject: PRJEB36244 datasets at SRA repositories (https://www.ncbi.nlm.nih.gov/sra). Differential gene expression in response to abiotic factors such as drought, heat, combined stress (Liu et al., 2015), cold (Li et al., 2015b), and salt stress induced by NaCl (Bio Project Accession: PRJNA632706) was carried out by using the transcriptome datasets available at SRA repositories at NCBI. The gene expression changes in response to infections by *Claviceps purpurea*, which causes ergot disease, and *Fusarium graminiearum*, responsible for head blight in wheat, were analyzed using transcriptome datasets obtained from SRA (**Supplementary Table S1**).

The relative gene expression levels across five different tissue types and changes in gene expression in response to abiotic and biotic stresses were calculated as FPKM (Fragments Per Kilobase Per Million). The FPKM changes in gene expression were determined by search of Ta-*CrRLK1L* gene Ensembl Plant database identifiers against the *T. aestivum* RNA-seq Database (http://ipf.sustech.edu.cn/pub/wheatrna/) at the Plant Public RNA-seq Database (Zhang et al., 2020b), using restricted SRA accessions search for biological replicates for each dataset for five tissue types or abiotic or biotic stresses. This database is comprised of a pipeline that uses tools such as HISAT2 (Kim et al., 2015; Kim et al., 2019) to align raw reads to the reference genome and StringTie (Pertea et al., 2015) to assemble and quantify transcripts. The heatmap for FPKM values for the relative level of gene expression and the fold change in response to stress conditions were visualized by TBtools (Chen et al., 2020).

Gene co-expression network analysis for the moderately or highly expressed *CrRLK1Ls* in tissues was analyzed using tissue-specific RNA-Seq network in the Wheat co-expression network (WheatCENet) database using default parameters and positive co-expression. This database comprises of RNA-seq datasets for tissue specific gene expression in wheat (http://bioinformatics.cau.edu.cn/WheatCENet) (Li et al., 2023). The AgriGO v2.0 database (http://bioinformatics.cau.edu.cn/WheatCENet/GOanalysis.php) was utilized for the GO enrichment analysis of the co-expressed network nodes identified from the WheatCENet database using the Singular Enrichment Analysis (SEA) tool in the *T. aestivum* (Chinese spring species) with the default parameters and a False Discovery Rate (FDR) value of 0.05. The query consisted of the co-expressed network nodes Ensembl IDs of the genes.

## 3 Results

### 3.1 *In silico* analysis identified 15 members of the *CrRLK1L* gene family *in T. aestivum*

In total, forty-three genes encoding CrRLK1Ls were identified in the hexaploid genome of *Triticum aestivum*, which consists of fifteen paralogous genes with three homeologous copies each, namely A, B, and D, from their progenitor species, except for Ta-*CrRLK1L2* and Ta-*CRLK1L7*, which had missing -*2-*D and -*7-*A copies. However, the blastn search for Ta-*CrRLK1L2* at IWGSC RefSeq v2.1 and Ensembl plants had a hit with 88% identity within the coding regions of the genes on Chr3A. To confirm the presence of a -*3-*D copy in the diploid progenitor, a blastn search in the *Ae. tauschii* genome in the NCBI database was done, which also did not show a high sequence similarity hit and suggested that *-2-*D may also be missing in *Ae. tauschii.* Ta*-CrRLK1L2-*A and *2-*B had 789 aa and 814 aa length sequences in the Ensembl database, which were more like transcript variants, and these sequences were corrected using TSA and wheat WGA_v0.4 scaffolds at the IWGSC database. Among *CrRLK1L*s, -*7-B* was the shortest paralog with a length of 630 aa and showed missing amino acids in the N-terminal region, which was also confirmed using the IWGSC database and whole genome shotgun (WGS) contigs at the NCBI database. Sequences for *CrRLK1L*s were confirmed from at least two independent databases, including Ensembl Plants and the NR, TSA, and EST databases at NCBI. The transcript variants for Ta-*CrRLK1L* gene family members, -*11-*B (two variants), -*4-*B (two variants), and -*14-*D (4 variants), were identified from the Ensembl plant database, and the correct variant that codes for FL protein was selected for further analysis (**Table 1**, **Supplementary Table S2A**).

**Table 1:** Sequence confirmation for *CrRLK1L* genes in *Triticum aestivum*.

Gene	Chr	aa	MW	pI	Start codon	Alignment	Ensembl IDs	TSA IDs	EST IDs	NR IDs	Localization
Ta-*CrRLK1L1*-A	5A	846	93	6.88	593319527	(±)	TraesCS5A02G397100.1	NA	NA	XM_044527090.1	PM
Ta-*CrRLK1L1-B*	5B	848	93.2	6.66	582057336	(±)	TraesCS5B02G402100.1	NA	NA	XM_044537865.1	PM
Ta-*CrRLK1L1-D*	5D	846	92.9	6.66	475127910	(±)	TraesCS5D02G406800.1	NA	NA	XM_044546050.1	PM
Ta-*CrRLK1L2*-A	3A	839	92.7	6.3	44901081	(+/+)	TraesCS3A02G072500.1*	NA	NA	XM_044482702.1	PM
Ta-*CrRLK1L2-B*	3B	840	93.0	6.61	65473404	(+/+)	TraesCS3B02G086600.1*	GILY01003800.1	NA	XM_044491216.1	PM
Ta-*CrRLK1L3*-A	5A	911	99.2	6.18	540676639	(+/+)	TraesCS5A02G330500.1	GILY01022517.1	HX029436.1	XM_044526491.1	PM
Ta-*CrRLK1L3-B*	5B	910	99.1	6.58	518340138	(+/+)	TraesCS5B02G330700.1	GILY01026266.1	HX141661.1	XM_044533983.1	PM
Ta-*CrRLK1L3-D*	5D	885	96.4	6.74	428697175	(+/+)	TraesCS5D02G336300.1	IAAL01004003.1	CJ906519.1	AK448106.1	PM
Ta-*CrRLK1L4*-A	3A	908	97.3	6.12	109466537	(±)	TraesCS3A02G308100.1	GILY01002072.1	HX072674.1	XM_044483324.1	PM
Ta-*CrRLK1L4-B*	3B	909	97.5	5.83	154502792	(±)	TraesCS3B02G151200.1	GILY01004238.1	NA	XM_044491877.1	PM
Ta-*CrRLK1L4-D*	3D	902	97	5.9	93598471	(+/+)	TraesCS3D02G134400.1	GILY01008167.1	NA	XM_044499947.1	PM
Ta-*CrRLK1L5*-A	4A	890	96	6.16	181465287	(+/+)	TraesCS4A02G133800.1	GEWU01281019.1	CJ667826.1	XM_044507489.1	PM
Ta-*CrRLK1L5-B*	4B	893	96.2	6.1	373930661	(±)	TraesCS4B02G171100.1	JV866858.1	NA	XM_044513925.1	PM
Ta-*CrRLK1L5-D*	4D	890	96	6.23	301285682	(±)	TraesCS4D02G173100.1	NA	JZ888240.1	XR_006447369.1	PM
Ta-*CrRLK1L6*-A	5A	873	94	5.49	679888473	(±)	TraesCS5A02G514500.1	GJAR01010904.1	GIJS01080162.1	XM_044528115.1	PM
Ta-*CrRLK1L6-B*	4B	869	93.7	5.63	637896073	(+/+)	TraesCS4B02G345400.1	GILY01017244.1	BJ288583.1	XM_044515803.1	PM
Ta-*CrRLK1L6-D*	4D	872	94.2	5.34	497695665	(+/+)	TraesCS4D02G340500.1	GJAR01010903.1	D862711.1	AK454253.1	PM
Ta-*CrRLK1L7-B*	2B	630	69.2	6	740594727	(+/+)	TraesCS2B02G536700.1*	GJAR01082108.1	NA	XM_037631706.1	PM
Ta-*CrRLK1L7-D*	2D	822	89.3	5.94	604946059	(+/+)	TraesCS2D02G509800.1	NA	NA	XM_044478779.1	PM
Ta-*CrRLK1L8*-A	4A	849	91.2	7.59	115710317	(+/+)	TraesCS4A02G102900.1	GILY01011736.1	NA	XM_044507140.1	PM
Ta-*CrRLK1L8-B*	4B	848	91.1	6.73	430809222	(±)	TraesCS4B02G201600.1	GILY01016151.1	NA	XM_044514343.1	PM
Ta-*CrRLK1L8-D*	4D	849	91.1	6.93	349251680	(±)	TraesCS4D02G202300.1	GILY01019194.1	GH724703.1	AK447161.1	PM
Ta-*CrRLK1L9*-A	1A	892	96	6.11	399730575	(+/+)	TraesCS1A02G227300.1	JP843605.1	BQ170203.1	XM_044476330.1	PM
Ta-*CrRLK1L9-B*	1B	892	96.1	6.11	434871334	(+/+)	TraesCS1B02G241400.1	GFFI01002932.1	CA644342.1	XM_044549574.1	PM
Ta-*CrRLK1L9-D*	1D	893	96.1	5.98	319190397	(+/+)	TraesCS1D02G228900.1	JP843602.1	CJ776640.1	XM_044594081.1	PM
Ta-*CrRLK1L10*-A	2A	845	94.3	7.64	712778715	(±)	TraesCS2A02G463000.1	GILY01056615.1	BJ311332.1	XM_044603099.1	PM
Ta-*CrRLK1L10-B*	2B	845	94.1	7.1	690085072	(±)	TraesCS2B02G484700.1	GFFI01006924.1	CJ828757.1	XM_044470162.1	PM
Ta-*CrRLK1L10-D*	2D	845	94.3	7.93	572179262	(±)	TraesCS2D02G463600.1	GFFI01023000.1	BJ305745.1	XM_044478390.1	PM
Ta-*CrRLK1L11*-A	1A	844	93.5	7	108940325	(±)	TraesCS1A02G108100.1	GILY01047392.1	NA	XM_044462494.1	PM
Ta-*CrRLK1L11-B*	1B	845	93.8	7.94	167737188	(±)	TraesCS1B02G130300.2	GILY01018384.1	BQ240349.1	XM_044535279.1	PM
Ta-*CrRLK1L11-D*	1D	801	88.7	8.69	109363772	(+/+)	TraesCS1D02G110900.1	NA	GH726518.1	XM_044592692.1	Chl
Ta-*CrRLK1L12*-A	4A	892	96.7	5.96	891038	(+/+)	TraesCS4A02G001100.1	NA	NA	XM_044506147.1	PM
Ta-*CrRLK1L12-B*	4B	889	96.6	5.51	707871	(±)	TraesCS4B02G002300.1	AAL01003834.1	NA	AK447962.1	PM
Ta-*CrRLK1L12-D*	4D	890	96.6	5.66	624327	(+/+)	TraesCS4D02G001000.1	NA	LU084346.1	AK455935.1	PM
Ta-*CrRLK1L13*-A	7A	845	91.2	5.14	27396002	(±)	TraesCS7A02G055000.1	GAEF01048892.1	LU004232.1	XM_044570699.1	PM
Ta-*CrRLK1L13-B*	4A	844	91.1	5.14	714717418	(±)	TraesCS4A02G441600.1	NA	BF482703.1	XM_044505410.1	PM
Ta-*CrRLK1L13-D*	7D	844	91.2	5.12	26712831	(±)	TraesCS7D02G050100.1	GAEF01049320.1	LU061168.1	XM_044585135.1	PM
Ta-*CrRLK1L14*-A	7A	841	94	6.46	504309871	(±)	TraesCS7A02G340700.1	GILY01042054.1	JZ884359.1	XM_044567157.1	Chl
Ta-*CrRLK1L14-B*	7B	873	93.7	6.46	454608446	(±)	TraesCS7B02G241900.1	GILY01045260.1	CK162158.1	XM_044580593.1	Vac
Ta-*CrRLK1L14-D*	7D	841	96.8	6.75	434948804	(+/+)	TraesCS7D02G338800.3	GILY01048908.1	CJ707292.1	XM_044587009.1	Chl
Ta-*CrRLK1L15*-A	2A	847	92.8	6.46	389183312	(±)	TraesCS2A02G253200.1	GFFI01088856.1	NA	XM_044600996.1	Vac
Ta-*CrRLK1L15-B*	2B	843	92.6	6.53	375538545	(+/+)	TraesCS2B02G272800.1	GILY01059171.1	NA	XM_044467908.1	Vac
Ta-*CrRLK1L15-D*	2D	845	92.6	6.66	308836841	(+/+)	TraesCS2D02G253800.1	JV890479.1	CJ918402.1	XR_006425141.1	Vac

Chr represent Ta-*CrRLK1L*s gene locations on chromosomes, aa is amino acid lengths, MW- Molecular Weight in Kilo Dalton, and localization represent subcellular localization for proteins determined by using WoLF PSORT. Ensembl plants, NR, TSA and ESTs database identifiers for genes are given. TraesCS3A02G072500.1* and TraesCS3B02G086600.1* genes in Ensembl plants database showed transcripts that code for 789 aa and 814 aa, these genes were corrected using wheat-WGA_v0.4-scaffolds at IWGSC database (https://wheat-urgi.versailles.inra.fr/). TraesCS2B02G536700.1* code for 630 aa and has missing N-terminal amino acids, which was confirmed by Whole Genome Shotgun (WGS) contigs at NCBI and wheat WGA_v0.4-scaffolds at IWGSC database. PM, Chl, and, Vac denote plasma membrane, chloroplast, and vacuole localization for CrRLK1Ls, respectively.

The protein lengths and molecular weights for Ta-CrRLK1Ls were 801–911 aa and 88.7-99.2 KDa, respectively. Ta*-CrRLK1L6*-A and -*13-B* were translocated from 4A to 5A and 7B to 4A chromosomes, respectively. Most homoeologous copies of Ta-*CrRLK1L* showed more than 93.5% similarity in the protein-coding regions within their homeologs, except for -*5-*B and -*5-*D, which had 91.0% similarity, respectively. The sub-cellular localization predicted by using the WoLF PSORT tool showed that 36 Ta-CrRLK1Ls were localized to the plasma membrane. Surprisingly, *-14-*B, *-15-*A, *-15-*B, and *-15-*D were found localized to the vacuole, while *-9-*D, *-12-*A, and *-12-*D were localized to the chloroplast (**Table 1**).

Blast searches using *T. aestivum CrRLK1L* protein-coding sequences in monocot species *Ae. tauschii*, *B. distachyon*, and *H. vulgare* identified 14, 11, and 15 CrRLK1L encoding genes, respectively. The *CrRLK1L2-*D copy was missing in *Ae. tauschii* (**Supplementary Table S2B**).

### 3.2 Ta*-CrRLK1Ls* members have highly conserved protein domains and show evolutionary conservation with closely related species

**Figure 1:**
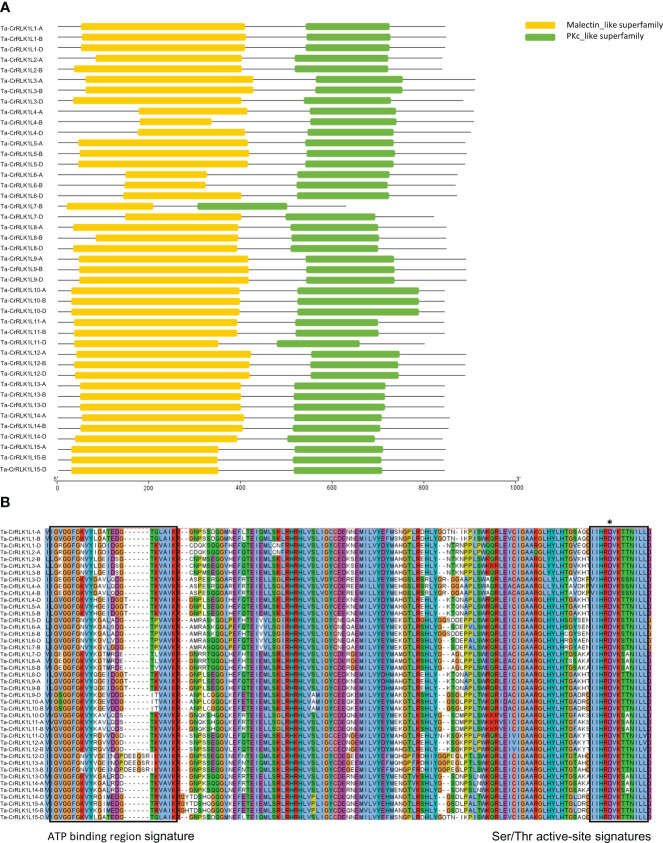
Conserved domains and multiple sequence alignment of *CrRLK1L* gene family in *T. aestivum*. **(A)** represent conserved characteristic domains such as a malectin-like domain and a catalytic domain for serine/threonine protein kinases determined by SMART ((http://smart.embl-heidelberg.de/) and Batch CD-search tool (https://www.ncbi.nlm.nih.gov/Structure/cdd/wrpsb.cgi) at NCBI. **(B)** Full-length protein sequences were aligned by ClustalW and visualized by Jalview (https://www.jalview.org/). Conserved motifs in the protein kinase domain, such as protein kinases ATP-binding region signature (PS00107) and serine/threonine kinases active-site signatures (PS00108) determined by PROSITE (https://prosite.expasy.org/) are represented by boxes, and active site residue (D) is marked by an asterisk*.

Multiple sequence alignment and conserved domain prediction using SMART and NCBI Batch-CD Search tool showed that *T. aestivum* CrRLK1Ls had highly conserved characteristic domains that included a malectin-like domain (PF12819) and a catalytic domain for serine/threonine protein kinases (SM000220) (**Figure 1A**). The lengths of the malectin-like domain in Ta-CrRLK1L varied between 270 and 378 aa, except for the *-7-B* gene copy, which had a 198 aa malectin-like domain. This suggests that the point mutation caused the truncated malectin-like domain in the -7B copy of Ta-CrRLK1L. The serine/threonine protein kinase domain lengths were ranged from 265 to 281 aa. Sequence analysis using PROSITE showed that Ta-CrRLK1Ls were comprised of featured domains that include an ATP-binding region signature (PS00107) and an active site signature for serine/threonine protein kinases (PS00108). The serine/threonine protein kinases had the active site residue D, which is directly involved in the catalytic functions predicted by PROSITE (**Figure 1B**).

**Figure 2:**
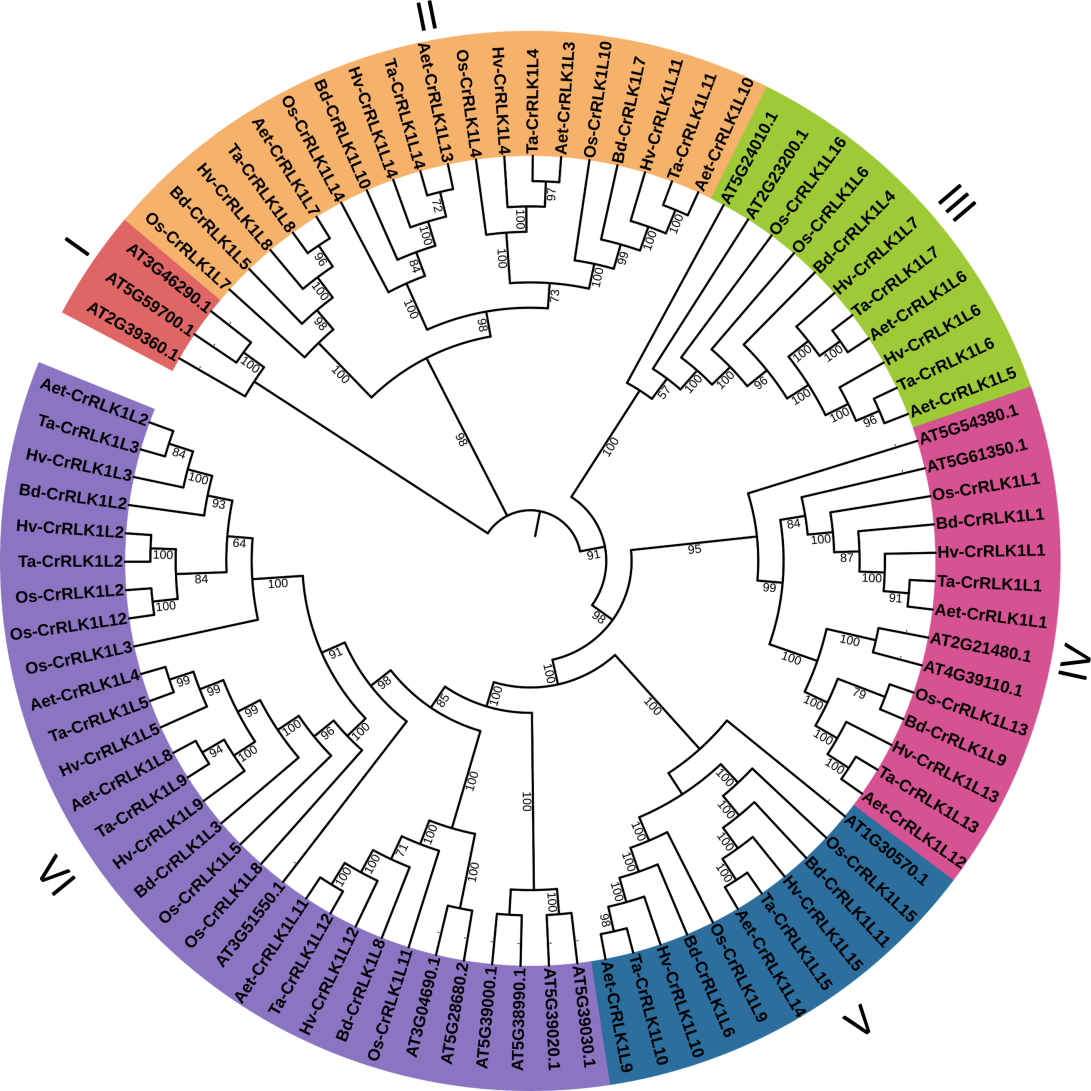
Phylogenetic analysis of CrRLK1L in *T. aestivum* and other species. This analysis involved 88 amino acid sequences. All positions with less than 95% site coverage were eliminated, i.e., fewer than 5% alignment gaps, missing data, and ambiguous bases were allowed at any position. There was a total of 622 positions in the final dataset. Evolutionary analyses were conducted in MEGA11. Phylogenetic stress was visualized by iTOL (https://itol.embl.de/).

Phylogenetic analysis of CrRLK1Ls using full-length protein sequences showed that more closely related species like *T. aestivum*, *Ae. tauschii*, and *H. vulgare* were clustered together. The paralogs of Ta-CrRLK1Ls were clustered with Arabidopsis CrRLK1Ls, which have been known to be involved in plant reproduction and pollen-pistil interaction in Arabidopsis. At-*FER* was clustered with Ta*-*CrRLK1L5, -9, At-ANX1 and At-ANX2 were clustered with -12, At-HERK2 was clustered with -10, At-BUPS1 and At-BUPS2 were clustered with -13, At-CAP was clustered with -1, and At-HERK1 and At-ANJEA were clustered with -4, -8, -11 and *-*14 (**Figure 2**, **Supplementary Figure S1**), which suggests that these genes may also have a similar role in plant reproduction.

**Figure 3:**
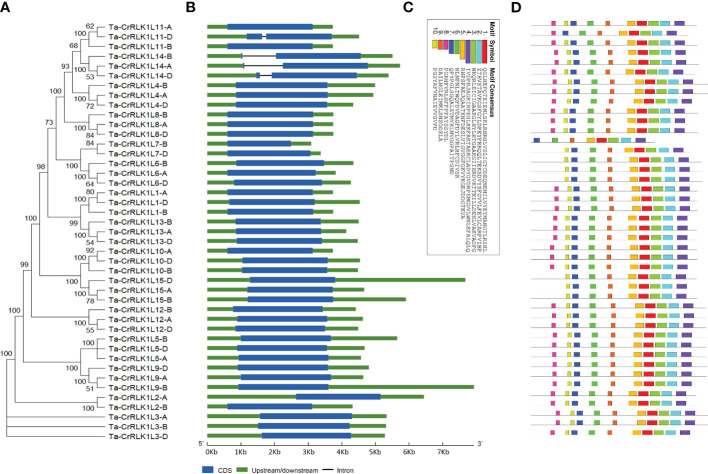
Structural organization of *CrRLK1L* gene families in *Triticum aestivum*. **(A)** A phylogenetic tree was constructed using Neighbor-joining method and 1000 bootstrap iterations by MEGA11 (https://www.megasofware.net/). **(B)** Intron/exon structure for gene family members was displayed using the Gene Structure Display Server (http://gsds.gao-lab.org/). Blue boxes are the exons, green is the upstream or downstream region, and lines between boxes denote introns. **(C, D)** are the ten identified motifs in Ta-CrRLK1Ls protein sequences by using MEME Suite 5.5.2 (https://meme-suite.org/meme/index.html).

Phylogenetic analysis in *T. aestivum* revealed clustering of closely related CrRLK1Ls such as -1 and -13, -5 and -9, -6 and -7, -10 and -15, as well as -11 and -14 (**Figure 3A**). The intron/exon junctions determined by Splign showed that most Ta-*CrRLK1Ls* had no introns and were comprised of single exons, except for the paralog -*11-*D and homeologs of -*14*, which have a single intron and two exons, suggesting the structural diversity within gene family (**Figure 3**, **Supplementary Table S3A**). Ta*-CrRLK1-11-*D copy had the smallest and largest introns of 124 bp and 1149 bp, respectively. The homeolog of *-14* had shorter first exons of 36 bp, 36 bp, and 111 bp for *-14-*A, *-14-*B, and *-14-*D, respectively. The MEME suite predominantly predicted motifs associated with serine/threonine protein kinase domains (**Figures 3C, D**, **Supplementary Table S3B**).

### 3.3 Ta-CrRLK1Ls display varied distribution in seven chromosomes and are absent in chromosome 6

**Figure 4:**
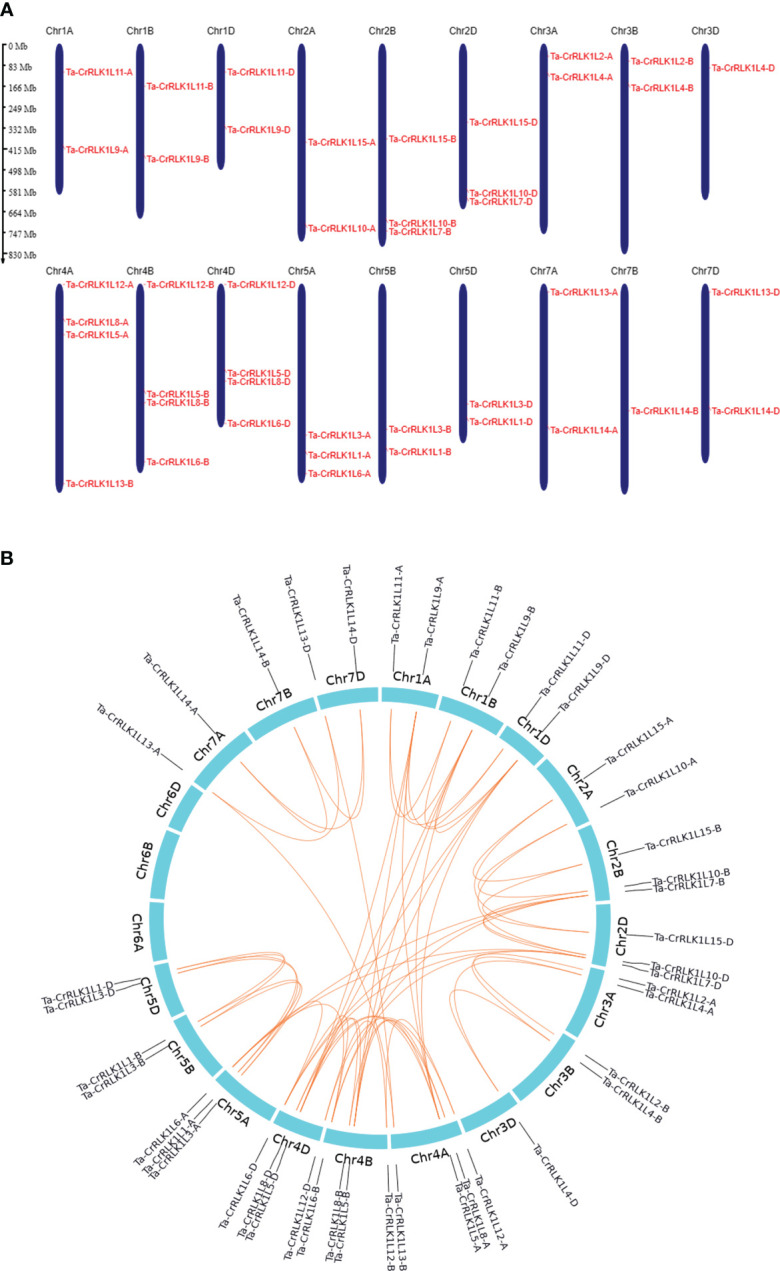
Distribution of genes across chromosomes and gene duplication in *CrRLK1L*s in *Triticum aestivum.* **(A)** The distribution of Ta-*CrRLK1L* genes on six chromosomes is given, and gene names are represented in red. Chromosome 6 did not have the distribution of the *CrRLK1Ls*. Genes were mapped by their physical locations, and the scale bar given is in megabases. Gene positions on the chromosomes were visualized using MG2C v2.1 (http://mg2c.iask.in/mg2c_v2.1/). **(B)** Gene duplication for *CrRLK1L*s in *T. aestivum* is analyzed and illustrated using shinyCircos-V2.0 (https://venyao.xyz/shinycircos/). The lines between the paralogs indicate the duplication among gene family members.

Chromosome 4A, 4B, and 4D had the highest distribution of *CrRLK1L* genes and showed the distribution of four genes, including homeologs for Ta*-CrRLK1L5*, -*8* and -*12*, and paralogs -*6B* and -*6D*. However, 4A showed the translocated *-13-*B paralog from 7B to 4A chromosome (**Figure 4A**, **Table 1**). Chromosomes 3D and 7B had a distribution of single *CrRLK1L* genes that included *-4-*D and *-14-*B, respectively. Chromosome 6 did not have any distribution of *CrRLK1L* genes and, hence, was eliminated (**Figure 4A**).

Gene duplication analysis using the criteria of an 80% cut-off for alignment coverage and sequence identity at nucleotide level among Ta-*CrRLK1L* gene families showed that only the gene pair *CrRLK1L6* and *-7*, and *-5* and *-9* paralogs had the gene duplication events (**Figure 4B**). Other *CrRLK1L* paralogs did not meet these criteria. The Ka/Ks ratio analysis suggested that these duplicated gene pairs had a value of less than 0.45, indicating that the evolution of these *CrRLK1L* genes is under purifying selection (**Supplementary Table S4**).

### 3.4 Ta*-CrRLK1L* transcripts show altered expression in different tissues with *CrRLK1L-12* and -*13* highly expressed in the anther

**Figure 5:**
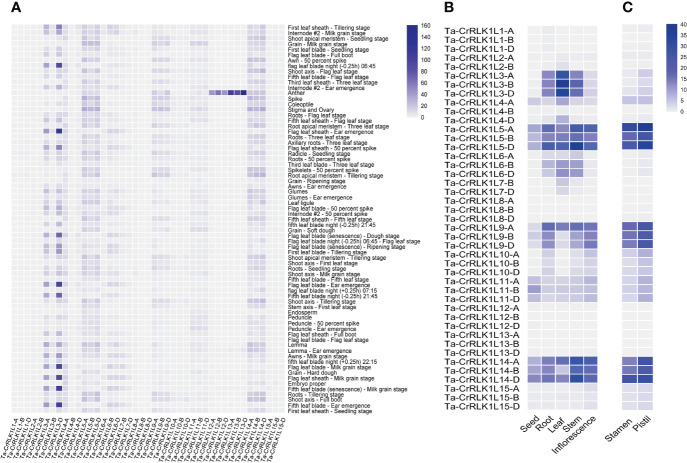
Gene expression analysis of *CrRLK1L* gene families in *Triticum aestivum.* Heatmaps represent the relative level of gene expression analyzed across **(A)** Seventy-one tissues in Azhurnaya spring wheat by using the wheat eFP browser (https://bar.utoronto.ca/). Values are expressed as Transcripts Per Million (TPM). **(B)** Relative gene expression levels in five tissue types namely seed, root, leaves, stem and inflorescence were analyzed from the transcriptome datasets by Pingault et al. (2015), and **(C)** Gene expression in immature stamen and pistil tissues was analyzed from the transcriptome datasets with Bio Project accession PRJEB36244 from the SRA database at NCBI. Values for B and C are expressed as Fragments Per Kilobase Per Million (FPKM).

Gene expression analysis for *CrRLK1L*s in *T. aestivum* across seventy-one tissues in Azhurnaya spring wheat showed that *CrRLK1L-12* and *-13* had higher expression levels in anther tissues. The homeolog -*13-D* exhibited the highest expression level at 158.4 TPM, making it the most highly expressed paralog in the *CrRLK1L* gene family. Similarly, paralog *-12-B* also showed a higher expression level of 61 TPM in anther tissues. (**Figure 5A**, **Supplementary Table S5A**). Gene expression for different tissue types analyzed from the Wheatomics database (http://wheatomics.sdau.edu.cn/) were also in agreement with the gene expression levels of *-12* and -*13* in anther as well as other tissues (**Supplementary Table 5B**). In the phylogenetic analysis, Ta-CrRLK1L-12 grouped closely with At-ANX1 and At-ANX2, whereas Ta-CrRLK1L-13 clustered with At-BUPS1 and At-BUPS2 (**Figure 2**, **Supplementary Figure S1**). These genes have a role in maintaining pollen tube integrity and preventing premature rupture before reaching synergid cells. This suggests that Ta-CrRLK1L-12 and -13 may have similar functions in wheat. Ta-*CrRLK1L5*, *-9*, and *-14* exhibited moderate expression levels in the stigma and ovary tissues and were clustered with At-FER, whereas -11 and -14 were clustered with At-HERK1 and At-ANJ, known for their roles in pollen tube reception at synergid cells during reproduction, suggesting their involvement in pollen tube reception during fertilization.

The relative level of gene expression analyzed across five tissue types was in agreement with the analyzed expression for seventy-one tissue types for *CrRLK1L3* and showed that it was highly expressed in leaf at whole plant fruit formation (30–50% moisture stage), with expression values of 29.2 to 36.3 FPKM (**Figure 5B**, **Supplementary Table S6**). Ta*-CrRLK1L5* and *-14* were expressed across five tissue types with different expression levels. Paralog -*5-*B was least expressed in seed with 8.8 FPKM, while *-5-D* was highly expressed in stem with 34.5 FPKM. Homeologs of *-6* were expressed in stem, leaf, and root tissues from 3.43 to 13.89 FPKM.

To explore Ta-*CrRLK1L’s* role in plant reproduction, gene expression was analyzed in immature stamen and pistil tissues of a cultivar Fielder. Ta-*CrRLK1L5, -9*, and *-14* exhibited higher expression than other family members in both stamen and pistil tissues; however, the gene expression levels in pistil tissues were higher than in stamen tissues. *-5-*A and *-14-*D had a higher level of expression than their homeologs, with 38.3 and 29 FPKM in pistil tissues, respectively, which was in agreement with the seventy-one tissues transcriptome data (**Figure 5C**, **Supplementary Table S7**). Similar paralogs were also expressed in stamen tissues at 27 and 31 FPKM values, suggesting dual roles of these genes in male as well as female side in pollen-pistil interaction.

Gene co-expression network analysis of seven moderately or highly expressed paralogs, namely *-3A, -5A, -9A, -11A, -12A, -13A*, and *-14A* using the WheatNet database revealed the functional and regulatory roles in morphogenesis and developmental processes (**Supplementary Tables S8A–C**). GO enrichment analysis for *-3A* co-expression network nodes showed the enrichment in enzyme linked receptor protein signaling pathway (GO:0007167), plant epidermal cell differentiation (GO:0090627), root epidermal cell differentiation (GO:0010053) and cell surface receptor signaling pathway (GO:0007166). Co-expression nodes for *-5A* showed the enrichment mostly in the signaling pathways such as cellular response to organic cyclic compound (GO:0071407), transmembrane receptor protein tyrosine kinase signaling pathway (GO:0007169), and steroid hormone mediated signaling pathway (GO:0043401). Co-expression network of -*9A* showed enrichment in cellular component organization (GO:0016043), embryo sac development (GO:0009553), ubiquitin protein ligase binding (GO:0031667), symplast (GO:0055044) and External encapsulating structure (GO:0030312). Similarly, for *-12A* and *-13A* co-expression network enrichment in the developmental growth (GO:0048589), unidimensional cell growth (GO:0009826), cell growth (GO:0016049) and developmental growth involved in morphogenesis (GO:0060560O) was observed, suggesting the redundancy in the functioning of these *CrRLK1L*s. These results suggested that these *CrRLK1L*s control the cell morphogenesis and developmental processes through different pathways while others act redundantly though similar pathways. The details of the positive interactions of the *CrRLK1L*s with the co-expressed genes, and co-expression nodes, edges, and GO analysis is given (**Supplementary Figure 2** and **Supplementary Tables S8A-C**).

### 3.5 Expression of *CrRLK1Ls* is altered in response to environmental stimuli

**Figure 6:**
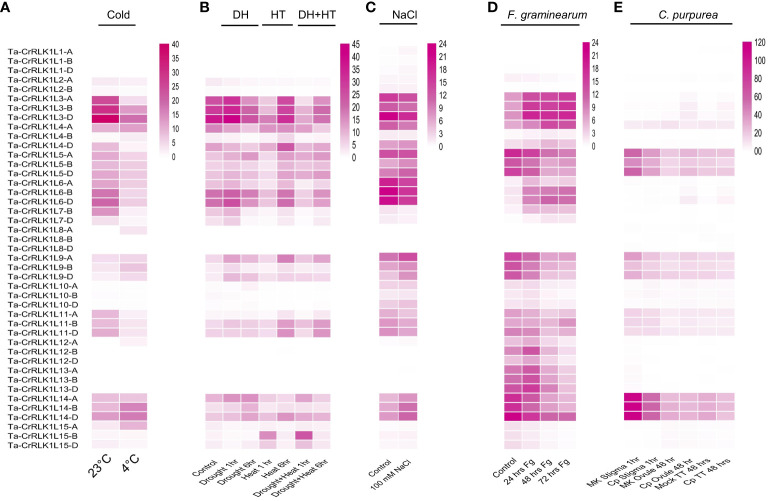
Gene expression analysis in response to abiotic and biotic stresses. Gene expression was analyzed for Ta-*CrRLK1L* genes in response to specific abiotic factors, including **(A)** Cold, **(B)** Drought (DH), heat (HT) and combined stress (DH+HT), and **(C)** NaCl (salt) stress, using transcriptome datasets from the NCBI Sequence Read Archive (SRA). Transcriptome analyses for biotic factors, such as **(D)** (*F*) *graminearum* (*Fg*) (Bio Project accession PRJNA522013), and **(E)** Infection to *C*. *purpurea* (dataset from Tente et al., 2021), were conducted with NCBI SRA datasets. In the figures, “MK” denotes mock control samples, “Cp” represents *C*. *purpurea* inoculated samples, and “TT” indicates ovary-transmitting tissues. The color scale reflects gene expression levels, measured in FPKM values (Fragments Per Kilobase Million).

Most of the *CrRLK1L* paralogs were upregulated or downregulated in response to one or more abiotic factors. In response to the cold treatments, the paralogs Ta-*CrRLK1L4-*B and *-15-*A were up-regulated by 3.0 and 3.3-fold, respectively. The paralogs that were downregulated to 15%, 18%, and 21% compared to control conditions included -*3-*A*, -7-*B, and *-11-*A*. CrRLK1L-3-*A had the highest 18% decrease in gene expression, from 24.69 FPKM to 4.42 FPKM. Other paralogs had a 30–47% decrease in the gene expression level in response to cold stress, which included one of the homeologs of Ta-*CrRLK1L3*, *-4, -6, -7*, and -*11* (**Figure 6A**, **Supplementary Table S9**).

The Ta-*CrRLK1L* gene family members showed changes in gene expression levels in response to drought, heat, and combined stress. The homeologs of *-9* and paralogs *-10-*A, and *-14-*B were induced by more than 2.3-folds in response to drought stress of 1 hr and 6 hr, while homeologs of *-2, -4, -7, -9, -10*, and *-15* were downregulated in the same, with *4-B* exhibiting highest downregulation of 25% in the 6 hr drought stress treatment. The paralogs upregulated in response to 1 hr or 6 hr of heat stress included -*4-*B*, -9-*A*, -10-*D*, -11, -15-*B and *-15-*D. In contrast, *-2, -3, -4, 6, -7, -9, -10*, and *-11* were downregulated, with the highest downregulation for *10-*B from 1.04 to 0.04 FPKM. In combined stress, -*15-*B, was induced 13.9-fold, while *10-*B was downregulated from 1.04 FPKM to 0.02 FPKM. The homeologs *-7-*B and -7*-*D were downregulated to 12% of their control levels, with FPKM values of 7.28 and 3.94 (**Figure 6B**, **Supplementary Table S10**).

Changes in gene expression analyzed in response to 100 mM NaCl treatments Ta-*CrRLK1L-1-*A had the highest induction of 5.77-fold compared to *-1-*B and -*1-*D, which were induced by 3.35 and 2.93-fold, respectively. However, the lower expression levels of 0.12, 0.08, and 0.23 FPKM for these homeologs were detected in control treatments. These results suggest that Ta-*CrRLK1L1* may have a role in salt stress responses (**Figure 6C**, **Supplementary Table S11**).

### 3.6 Expression of *CrRLK1L* transcripts is altered in response to head blight and ergot funguses

In response to *Fusarium graminearum* (*Fg*) inoculation, Ta-*CrRLK1L-7-*D was highly induced from 0.08 FPKM in control to 0.81, 1.85, and 1.75 FPKM in 24 hr, 48 hr, and 72 hr post-inoculation treatments. *-6-*D was induced by a 6.93-fold increase in 48 hr post-*Fg* inoculation. However, in response to 72 hr post-inoculation*, -15* homeologs were downregulated to 10-12% of their control values (**Figure 6D**, **Supplementary Table S12**).

*Claviceps purpurea* (*Cp*) infection in the stigma induced *-6-*D and *-15-*D paralogs from 0.06 FPKM in mock to 0.50 and 0.57 FPKM in 1 hr post-inoculation treatment, whereas the homeologs *-12-*A *and -12-*D were downregulated to 14% and 11% in the treatment compared to their control (**Figure 6E**, **Supplementary Table S13** *).* The paralogs *-3-*A and -*6-*D were induced by 10.26 and 10.92-fold in response to 48 hr post *Cp* inoculation in the ovule. However, in the control conditions, *-3-*A and *-6-*D have lower expression of 0.14 and 0.06 FPKM, respectively. -*6-*D *and -3-*A were also upregulated 48 hr post-inoculation in the ovary and transmitting tissue to a fold change of 7.04 and 4.26, respectively.

### 3.7 Various stress-responsive and hormonal-responsive *cis*-acting elements regulating plant development are present in the promoter regions of *CrRLK1L*s in *T. aestivum*

**Figure 7:**
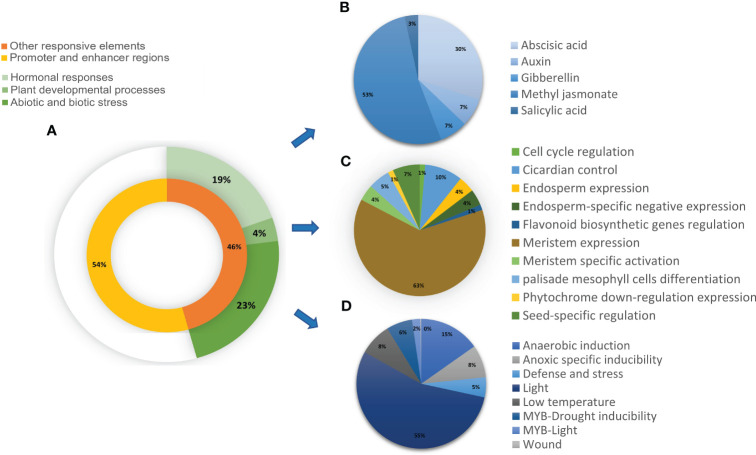
Distribution of the predicted cis-acting elements in *CrRLK1L* gene families in *T. aestivum*. The cis-acting elements predicted by analysis of 2kb upstream regions of genes by PlantCARE (https://bioinformatics.psb.ugent.be/webtools/plantcare/html/) were categorized into **(A)** Promoter and enhancer regions and other responsive elements. Other responsive elements were categorized into three main subgroups that include cis-acting elements involved in **(B)** Hormonal responses **(C)** Plant developmental processes, and **(D)** Abiotic and biotic stress. **(B–D)** are further classified based on their specific roles.

The prediction of *cis*-acting elements in the putative promoter regions of *CrRLK1L* genes revealed that 54% consisted of core promoter and enhancer region elements, including TATA and CAAT boxes. The remaining 46% was categorized into three groups, namely, *cis*-acting elements associated with hormonal responses, plant developmental processes, and abiotic and biotic stress conditions (**Figure 7A**). These categories were further subcategorized. For instance, hormonal responses were divided into abscisic acid, auxin, gibberellin, and salicylic acid (**Figure 7B**), while other elements were grouped based on their functions (**Figures 7C, D**). All Ta*-CrRLK1L* genes are comprised of *cis*-acting elements for light and abscisic acid. Ta*-CrRLK1L1* had *cis*-acting elements for palisade mesophyll cell differentiation, and *-14* had meristem-specific activation elements, which fell under the plant developmental processes category. The paralogs *-9-*B, *-12-*A, *-12-*D, *-13-*A, and *-13-*B possessed elements for seed-specific regulation. *Cis*-acting elements associated with defense and stress were predicted in all *CrRLK1L* genes except *-10*, *-11*, and *-14*. Additionally, at least one of the homeolog *CrRLK1L* genes showed *cis*-acting elements for methyl jasmonate (**Supplementary Table S14**).

## 4 Discussion

The *CrRLK1L* gene family in *T. aestivum* comprises 15 members and 43 paralogous genes. The coding regions of these genes exhibited a high similarity of more than 93.5%, as reported in various studies (Brunetti et al., 2018). The paralogs *-2-*D and *-7-A* in the progenitor species *Ae. tauschii* and *T. urartu* or *T. dicoccoides* did not have high sequence similarity in the coding regions, which suggests that these gene copies may also be absent in the progenitor species. The erroneous sequences for *CrRLK1Ls* from Ensembl Plants databases were corrected by wheat genome shotgun (WGS) contigs sequences at the NCBI database. The reverse translocations in the *T. aestivum* genome are most commonly observed, including the translocation between 4AL/5AL and 4AL/7 (Devos et al., 1995). Similar translocations were found in gene family members Ta-*CrRLK1L6* and *-13*. Surprisingly, *-13-*D and *-14* homeologs had introns, also found in *T. dicoccoides* when searched using blastn in the NCBI WGS assembly.

CrRLK1L gene families in Arabidopsis consist of 17 members, of which FER, ANJ, HERK1, BUPS1/2, and ANX1/2 are the critical players in plant reproduction and have roles in guiding the pollen tube to the synergid cells of the ovule for fertilization. The FER-ANJ complex acts in the stigma papillae and has an essential role in pollen germination, where it is proposed to maintain the ROS levels in the stigma papilla in the absence of the pollen and act as a negative regulator for stigma hydration (Zhou et al., 2021). Arabidopsis CrRLK1L members BUPS1/BUPS2 that complex with ANX1 and ANX2 act on the pollen tube side and function in the maintenance of pollen tube integrity and prevent its rupture before it reaches the synergid cells (Johnson et al., 2019). Phylogenetic analysis of CrRLK1L showed that ANX1/2 were clustered with Ta-CrRLK1L12, and BUPS1/2 were clustered with Ta-CrRLK1L13, which suggests that these genes are the Arabidopsis homologs for ANX and BUPS in wheat. This was supported by the tissue-specific gene expression analyzed across the seventy-one tissues of wheat, where Ta*-CrRLK1L12* and Ta*-CrRLK1L13* were highly expressed in anther tissues. However, gene expression analysis of immature stamen or pistil tissues during meiosis in wheat flowers at Zadok stages 41-49 did not show expression of these genes, which suggests the specificity of these genes to function in the later stages of plant reproduction when the pollen germinates on the stigma. Ta*-CrRLK1L5, -9*, and *-14* were also expressed in stigma and ovary tissues in seventy-one tissue types and had a relative level of gene expression in the range 18.25–32 FPKM, 15.12–15.38 FPKM, and 21.16–28.17 FPKM. Similar genes were also expressed in the analyzed transcriptome datasets for immature stamen and pistil and in five tissue types transcriptome datasets. The clustering of these genes suggests that *-5* and *-9* belong to the FER, and *-4*, *-11*, and *-14* belong to HERK, CURVY1, and ANJEA. Ta*-CrRLK1L-1* and *-14* copies showed the presence of specific palisade mesophyll cell differentiation and meristem-specific activation *cis*-acting elements in their promoter regions, which indicate that these genes may have a role in plant growth and regulation.

Co-expression network analysis of *CrRLK1Ls* and their GO enrichment using the network node genes for Ta-*CrRLK1L-3A*, *-5A*, *-9A*, *-12A*, and *-13A* provided a comprehensive detail about the multiple roles of *CrRLK1L* genes in the plant developmental processes in *T. aestivum*. Ta-*CrRLK1L3-A* is highly and moderately expressed in the leaf and root tissues based on the five tissue types expression datasets and the co-expressed node network of these genes showed the enrichment in the signaling and cellular differentiation process. Similarly, -9A is moderately expressed in different tissues such as root, leaf, stem and inflorescence and showed the enrichment in the GO terms associated with the cellular organization. The presence of *early nodulin like*-protein, members of which are involved in pollen tube reception in Arabidopsis (Hou et al., 2016), in the co-expression node network of Ta-*CrRLK1L12-A* and -*13* A, which are mostly expressed in the pollens suggest possible regulatory networks between these genes. However, in the agriGO v2.0 database we were unable to find GOs for *-11-A* and *-14A*, which may be due to the smaller number identified co-expression node network for these genes below ten.

Characterization of various gene families in *T. aestivum* involved in developmental processes and stress responses have been studied using RNA-seq, and the diversity in the gene expression and responses in the tissues as well as abiotic stresses such as cold, drought, heat, and biotic stress conditions such as infection to *F. graminearum*, has also been observed (Riaz et al., 2021; Gawande et al., 2022; Ke et al., 2022; An et al., 2023). The gene expression in the specific tissues or in response to stress condition indicate the possible roles of the gene family members in the developmental processes or under stress conditions.

FER is a versatile CrRLK1L that functions in plant growth and development, abiotic stress, hormonal signaling, and plant immunity responses (Ji et al., 2020). In Arabidopsis, the responses to drought, cold, and heat in the FER mutant, *fer-4*, showed that FER acts as a negative regulator of drought stress and a positive regulator of the cold and heat stress responses (Chen et al., 2021). To determine the response of Ta*-CrRLK1L* genes, we analyzed the gene expression from the available transcriptome datasets. The cold stress-responsive gene *-3-A* was clustered with FER, which had the highest downregulation (18% downregulated compared to control) in response to cold stress. Another downregulated gene was the homeologs of -*6*, which clustered with AT5G24010.1 and AT2G23200.1. Ta*-CrRLK1L15-A* was induced more than 3.3-fold and clustered with HERK2 in Arabidopsis. At least one homeolog of -*3, -4, -6*, and -*9* was downregulated in response to 1 hr of heat and 1 hr of combined stress, whereas -*15-*D was induced in similar treatments. Among these genes, most were clustered with FER, HERK1, and ANJEA, whereas the upregulated copy *-15-D* was clustered with HERK2. Ta*-CrRLK1L-12* and *-13* also responded to *F. graminearum* infections, suggesting that these genes may also have a role in the head blight caused by *F. graminearum.* HERK2 homolog Ta*-CrRLK1L15* responded to varied stress conditions, including cold, heat, combined drought and heat, and *F. graminearum* infection.

Gene expression analysis and promoter mining for *cis*-acting elements showed interesting results. Abscisic acid and light-responsive elements were found in almost all the promoter regions of *CrRLK1Ls*, highlighting the role of *CrRLK1L* in plant developmental processes and ABA-mediated stress responses. In the moderately expressed root-specific homeologs of genes *-5*, *-9*, and -*14*, auxin-responsive elements, either TGA (AACGA) or AuxRR-core (GGTCCATC) were found, which indicates that these genes may have roles in the root development through auxin response (**Supplementary Table S14**).

The promoter analysis of stress-responsive *CrRLK1L* genes showed the presence of stress-responsive elements. For example, in the cold stress-induced *CrRLK1L-3A* gene promoter, low temperature or cold-responsive cis-acting element LTR (CCGAAA) was found. Similar cis-acting elements were found in the cold stress response genes -*4-*B, -*7-*B, -*9-*D, and -*15-*A, which were either upregulated or downregulated in response to cold stress. Interestingly, the MBS cis-acting element (CAACTG), which stands for MYB Binding Site and functions in drought stress responses, was identified in downregulated -paralogs *-4-*B and *-7-*D, and upregulated paralog *-9B*. Similar elements were also found in the downregulated -*2-*A and -*2-*B paralogs in response to combined drought and heat stress treatment for 1hr. Drought and salinity stress responses are also controlled through ABA-dependent signaling (Narusaka et al., 2003), we have observed ABRE elements (ACGTG) in the promoter region of homeologs of *-1*, which is induced by NaCl treatment. However, it is not essential that the presence of these cis-acting elements only affect gene expression and this requires the further experimental validations using molecular experiments that determines promoter activity.

Similarly, in biotic stress responses, the highly expressed paralog -*7D* contained a salicylic acid-responsive TCA element (CATCTTTTT). Salicylic acid application induces tolerance against fungal species in plant species (Chakraborty, 2021). *Fg*-induced paralogs -*3-*B and -*3-*D, induced 72 hours post-*Fg* inoculation, were composed of defense and stress-responsive TC-rich repeat elements (GTTTTCTTAC). The role of methyl jasmonate has been known to be in stress tolerance against pathogens and herbivores (Turner et al., 2002). Paralogs induced in response to *C. purpurea*, namely -*3-*A, -*3-*B, and -*6-*B, also showed the presence of Methyl jasmonate-responsive *cis*-acting elements. The presence of these *cis-*acting elements in the *CrRLK1L* genes, coupled with the expression data, supports the role of the *CrRLK1L* gene family in *T. aestivum* in plant developmental processes and adaptation to stresses.

## 5 Conclusions

We conducted an extensive *in silico* genome-wide analysis of the *CrRLK1L* gene family in wheat, providing valuable insights into the potential roles of these genes for the first time. In the wheat genome, we identified 15 *CrRLK1L* genes and 43 paralogs. Furthermore, our investigation of the Ta*-CrRLK1L* genes revealed the presence of the various *cis*-acting elements involved in specific biological processes, such as plant growth, development, and stress responses. Analyzing publicly available transcriptome data sets, we found diverse expression patterns of most Ta*-CrRLK1s* in different tissues, including reproductive tissues. Moreover, most *CrRLK1L* genes were upregulated or downregulated in response to abiotic or biotic factors. The data presented in our study will serve as a valuable resource for future functional characterization and validation of CrRLK1L proteins in wheat. Additionally, this knowledge will facilitate the development of targeted strategies for crop improvement, leveraging the potential of the *CrRLK1L* gene family.

## Data availability statement

The original contributions presented in the study are included in the **Supplementary Material**. All the T. aestivum and other species sequences are given as a supplementary files with IDs from different databases. The source of transcriptome datasets used in this study are given as **Supplementary Table S1**.

## Author contributions

NG: Formal analysis, Methodology, Writing – original draft, Writing – review & editing, Conceptualization. SS: Formal analysis, Supervision, Writing – review & editing.
