# Supplementary figures and images for: Genome wide characterization and expression analysis of CrRLK1L gene family in wheat unravels their roles in development and stress-specific responses
Source: Front Plant Sci. 2024 Mar 26;15:1345774. doi: 10.3389/fpls.2024.1345774 (PMC11002176; doi:10.3389/fpls.2024.1345774)

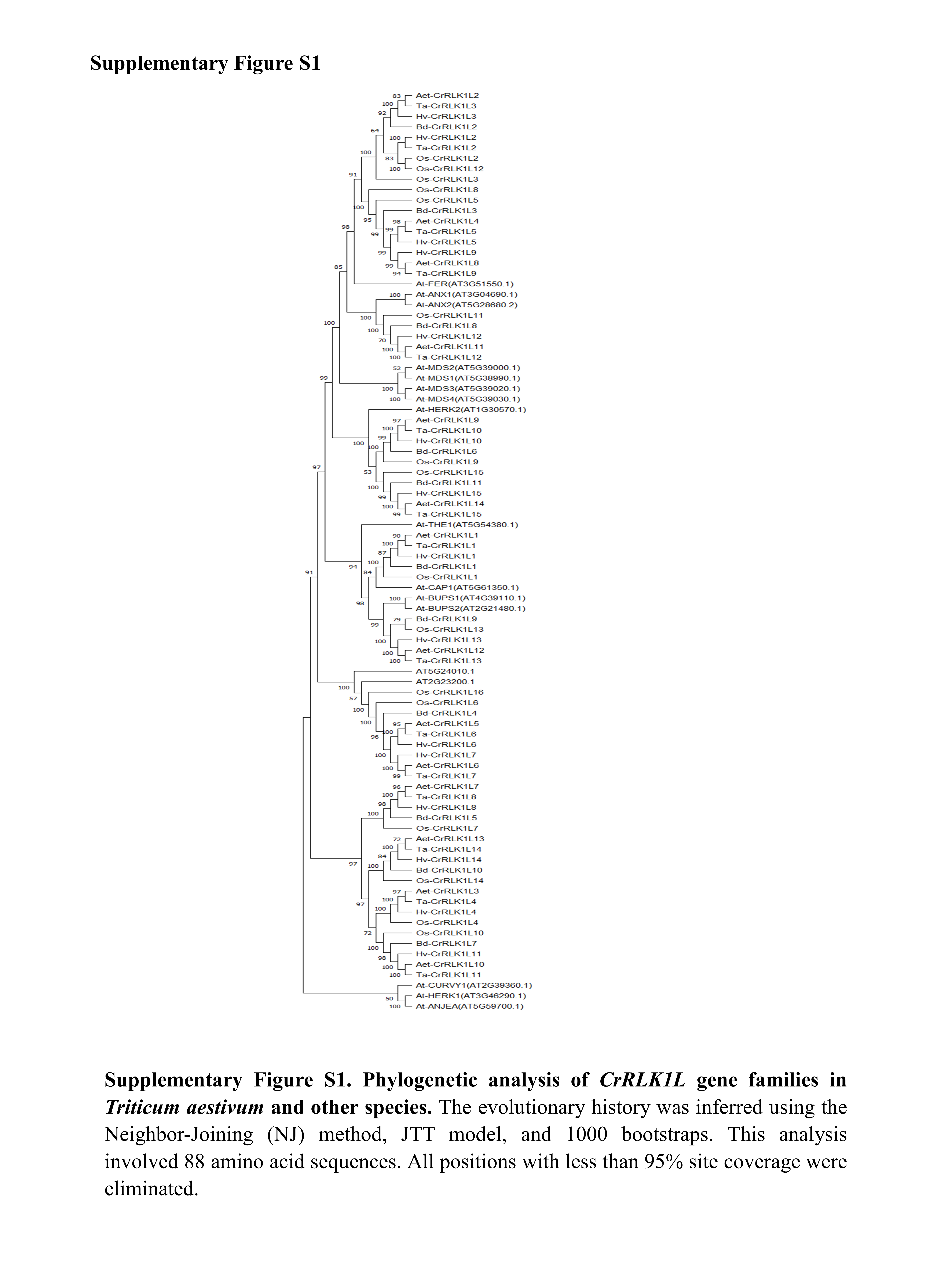

Supplement: Supplementary file 1 [file Image_1.tif]

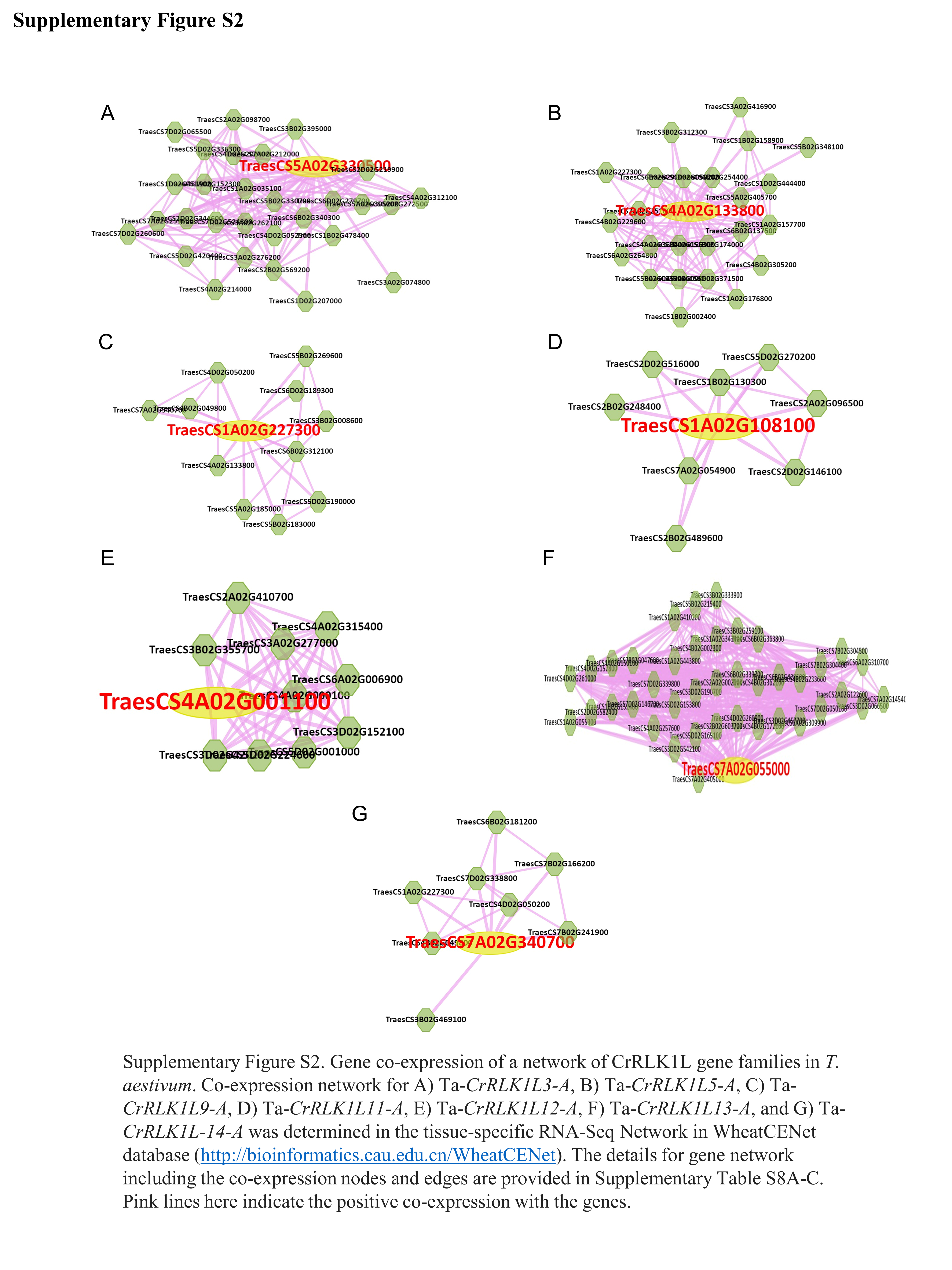

Supplement: Supplementary file 2 [file Image_2.tif]
