# Supplementary material for: Genome wide characterization and expression analysis of CrRLK1L gene family in wheat unravels their roles in development and stress-specific responses
Source: Front Plant Sci. 2024 Mar 26;15:1345774. doi: 10.3389/fpls.2024.1345774 (PMC11002176; doi:10.3389/fpls.2024.1345774)
Supplement: Supplementary file 4 [file Table_2.docx]

**Supplementary Table S2A. Nucleotide and amino acid sequences for CrRLK1L gene families in *T. aestivum*.** Sequences were collected from the Ensembl plants database and *-2-B* and *-2-D* copy was corrected from WGS contigs for *T. aestivum* at NCBI.

CDS sequences:

>Ta-*CrRLK1L1-*A CDS sequence

ATGCCAGCATTGGCAATTTTGGCCAGAAGCATGGCTCCGTGCAAGAGAGTGCCGATGTTC

TTGATCCTCTTCATCCTTTCCATTACTCGTGTAGCCACCACCAATGCAATTGCATCAAAA

GTAGATCGGTTCGTGCCTCAAGACAACTACCTCCTCAGCTGCGGGGCATCCGCTGCTGTG

CAGGTTGACGATGGCAGGACATTCCGCTCTGATCCTGAGTCGGTGTCATTTCTGTCAACC

CTGACGGACATCAAGATCGCCGCTAAAGCATCCCTGGCTTCTGCTTCACCATTATCCCCA

CTTTACCTTGATGCAAGAGTATTCTCTGATATCTCAACCTATAGCTTCTTCATCTCCCAG

CCTGGTCGCCATTGGATCCGGCTCTACTTCTTACCTATCACCGATAGCCAATACAACCTC

ACCACTGCAACATTTTCCGTGTCCACTGATAGCATGGTCCTTCTCCATGATTTCTCCTTC

ATAGCCAGTCCTCCAAACCCTGTGTTTAGGGAGTATCTTGTGTCAGCACAGGGAGACAAC

TTAAAGATCATCTTTACCCCAAAGAAAAACTCGATAGCATTCATCAATGCTATCGAGGTT

GTCTCAGCCCCACCAAGCCTTATTCCAAATACCACCACCAGAATGGGTCCTCAGGACCAG

TTTGACATATCCAACAATGCATTGCAGGTTGTCTACCGGCTGAACATGGGCGGTGCACTG

GTTACATCGTTTAATGACACACTAGGCAGAACCTGGCAGCCAGATGCACCCTTTCTGAAG

CTTGAGGCAGCAGCAGAGGCAGCTTGGGTTCCTCCTAGAACCATCAAGTACCCTGATGAC

AAGACTCTCACACCCCTCATCGCTCCAGCAAGCATCTACTCAACAGCACAGCAGATGGCA

TCAACAAATATCACAAATGCAAGATTCAACATAACATGGCAAATGGTTGCAGAGCCAGGA

TTCAGGTACCTTATCCGCCTACATTTCAGCGACATTGTCAGCAAGACACTCAATAGCCTC

TACTTCAATGTCTATATTAATGGCATGATGGCTGTTGCCAACCTTGATCTATCGAGCCTG

ACGATGGGGCTTGCAGTAGCCTACTACAAGGACTTGATTGCTGAGTCTTCCAGCATCATC

AATTCCACCCTTCTAGTCCAGGTTGGCCCAAACACAATCGACTCCGGCGACCCCAATGCC

ATCCTTAATGGCCTTGAGATCATGAAGATAAGCAATGAAGCAAGCAGCTTAGATGGCCTT

TTTTCACCAAAAACAAGCTCAGAAGTTAGTAAGACAACACTGACTGGCATAGCATTTGCT

TTGGCAGCAACAGCTGCATTGGCTGTAGTTATATGCTACAGGCGAAACCGTAAACCGGAA

TGGCAGAGGACAAACAGCTTCCATTCTTGGTTTCTTCCACTGAACTCGTCCTCGAGCTTC

ATGAGCAGTTGCAGCAGGCTCTCCAGAAATCGCTTTGGCTCCACAAGGACCAAGAGTGGA

TTTTCGAGCGTGTTTGCATCCAGTGCTTATGGATTGGGACGCTATTTCACCTTCGTCGAA

ATTCAGAAAGCCACGAAAAACTTTGAAGAAAAGGGTGTTATTGGTGTTGGTGGCTTCGGA

AAAGTTTATCTTGGTGCTACTGAAGATGGCACACAGCTGGCTATCAAGCGAGGCAATCCA

TCATCTGATCAAGGTATGAATGAGTTTCTGACTGAAATTCAAATGTTATCTAAACTTCGC

CACCGCCACCTGGTTTCACTCATTGGCTGTTGTGATGAGAACAACGAGATGATATTAGTT

TATGAGTTCATGTCAAATGGTCCACTAAGGGATCATCTGTATGGTGACACAAACATCAAG

CCTATCTCTTGGAAGCAGCGCCTTGAAGTTTGCATTGGGGCAGCAAAGGGTCTGCATTAT

CTTCATACAGGTTCAGCTCAGGGCATAATTCACCGTGATGTCAAGACTACCAACATCCTA

CTTGATGAAAATTTTGTCGCCAAGGTTGCTGATTTTGGCCTATCAAAAGATGCTCCATCC

CTCGAACAAACTCATGTGAGCACTGCTGTCAAAGGAAGCTTTGGGTATCTTGATCCAGAG

TACTTCAGACGTCAACAGCTGACAGATAAGTCTGATGTATACTCTTTTGGTGTGGTACTC

TTTGAAGTGCTGTGTGCAAGGCCAGCCATCAATCCAGCCCTTCCAAGAGACCAAGTGAAT

CTGGCAGAGTGGGCCCGTACATGGCACCGCAAGGGGGAGCTTGGCAAAATAATTGATCCC

AATATTGCAGGACAAATCAGGTCTGATTCACTTGAGATGTTTGCTGAGGCTGCAGAGAAA

TGCCTTGCTGACTATGGAGTCGACCGGCCAACAATGGGAGACGTGCTATGGAAACTTGAA

TTTGCCTTGCAACTTCAAGAGAAGGGTGATGTCGTTGACGGCACCAGTGATGGGATCGCA

ATGAAGAGCTTGGAGGTGACCAATGTGGATAGCATGGAGAAATCTGGTAATGCTATCCCA

TCTTATGTCCAAGGAAGATGA

>Ta*-CrRLK1L1-*B CDS sequence

ATGCCAGCATTGGCAATTTTGGCCAGAAGCAGCCGCATGGCCGAGTGGGAGAGGGTACCA

ATGTTCTTGATCCTCTTCATCCTTTCCATTACTAGTGTAGCCACCACCAATGCAATTGCA

TCAAAAGTTGATCGGTTCGTGCCTCAAGACAACTACCTCCTCAGCTGTGGGGCATCCGCT

GCTGTGCAGGTTGACGATGGCAGGACATTCCGCTCTGATCCTGAGTCGGTATCGTTTCTG

TCAACCCCGACAGACATCAAGATTGCCGCTAAAGCATCTCTGGCTTCTGCTTCGCCATTA

TCTCCACTTTACCTTGACGCAAGAGTATTCTCTGATATCTCAACCTATAGCTTCTTCATC

TCCCAGCCTGGTCGCCACTGGATCCGTCTCTACTTCTTGCCTATCACCGACACCCAATAC

AACCTCACCACTGCAACATTTTCTGTGTCCACTGAGAGCATGGTTCTCCTCCATGATTTC

TCGTTCATAGCCAGTCCTCCAAACCCTGTGTTTAGGGAGTATCTTGTGTCAGCACAGGGA

GACAACTTAAAGATCATCTTTACTCCAAAGAAAAACTCGATAGCATTCATCAATGCGATC

GAGGTTGTCTCAGCCCCACCAAGCCTTATTCCAAATACCACCACCAGAATGGGTCCTCAG

GACCAGTTTGACATATCCAACAATGCATTGCAGGTTGTCTACCGGCTGAACATGGGCGGT

GCACTGGTTACGTCGTTCAATGACACACTAGGCAGAACCTGGCTGCCAGATGCACCCTTT

CTGAAGCTTGAGGCAGCAGCAGAGGCAGCTTGGGTTCCTCCTAGAACCATCAAGTACCCT

GATGACAAGACTCTCACACCCCTCATCGCTCCAGCAAGCATCTACTCAACAGCACAGCAG

ATGGCCTCAACAAATATCACAAATGCAAAATTCAACATAACCTGGGTAATGGTTGCAGAG

CCGGGATTCAGGTACCTTATCCGCCTACATTTCAGCGACATTGTCAGCAAGACACTCAAT

AGCCTCTACTTCAATGTCTATATTAATGGCATGATGGCTGTTGCCAACCTTGATCTATCG

AGCCTGACAATGGGGCTTGCAGTAGCCTACTACAAGGACTTGATTGCAGAATCTTCCAGC

ATCATCAATTCCACCCTTGTAGTCCAGGTTGGCCCAAGCACAATCGACTCCGGCGACCCC

AATGCCATCCTTAATGGCCTTGAGATCATGAAGATAAGCAATGAAGCAAGCAGCCTAGAT

GGCCTTTTTTCACCAAAAACAAGCTCAGAAGCTAGTAAGAGGACACTGACTGGCATAGCA

TTTGCTTTGGCAGCAACAGCTGCATTGGCTGTGGTTATATGCTACAGGCGAAACCGTAAA

CCGGCATGGCAGAGGACAAACAGCTTCCACTCTTGGTTTCTTCCACTAAACTCGTCCTCG

AGCTTCATGAGCAGCTGCAGCAGGCTCTCCAGAAATCGCTTTGGCTCCACAAGGACCAAG

AGTGGATTTTCGAGCGTGTTTGCATCCAGTGCTTATGGATTGGGGCGCTATTTCACCTTC

GTGGAAATTCAGAAAGCCACGAAAAACTTCGAAGAAAAGGGTGTTATTGGTGTTGGTGGC

TTCGGAAAAGTTTATCTTGGTGCTACTGAAGATGGCACACAGCTGGCAATCAAGCGAGGC

AATCCATCATCTGATCAAGGTATGAATGAGTTTCTGACTGAAATTCAAATGCTATCAAAA

CTTCGCCACCGCCACCTGGTTTCACTCATTGGCTGTTGTGATGAGAACAATGAGATGATA

TTAGTTTATGAGTTCATGTCAAATGGTCCGCTAAGGGATCATCTGTATGGTGACACAAAC

ATCAAGCCTATCTCTTGGAAGCAGCGCCTTGAAGTTTGCATTGGGGCAGCAAAGGGTCTG

CATTATCTTCATACAGGTTCAGCTCAGGGCATAATTCACCGTGATGTCAAGACTACCAAC

ATCCTACTTGATGAAAATTTTATCGCCAAGGTCGCTGATTTTGGCCTATCAAAAGATGCT

CCATCCCTCGAACAAACTCATGTGAGCACTGCTGTCAAAGGAAGCTTTGGGTATCTTGAC

CCAGAGTACTTCAGACGTCAACAGCTGACAGATAAGTCTGATGTATACTCTTTTGGTGTG

GTACTCTTTGAAGTGCTGTGTGCAAGACCGGCCATCAATCCATCTCTTCCAAGAGACCAA

GTGAATCTAGCTGAGTGGGCCCGTACATGGCACCGCAAGGGGGAGCTTGGTAAAATAATT

GATCCCAATATCGCAGGGCAAATCAGGCCTGATTCACTTGAGATGTTTGCTGAGGCTGCT

GAGAAATGCCTTGCTGACTATGGAGTCGACCGGCCAACAATGGGAGATGTGTTATGGAAA

CTTGAATTTGCCTTGCAACTTCAAGAGAAGGGTGATGTTGTTGATGGCGCCAGTGATGGG

ATCCCAATGAAGAGCTTGGAGGTGTCCAATGTGGATAGTATGGAGAAATCTGGTAATGCT

ATCCCATCTTATGTCCAAGGAAGATGA

>Ta-*CrRLK1L1-*D CDS sequence

ATGCCAGCATTGGCAATTTTGGCCAGAAGCATGGCTGAGTGCAAGAGAGTGCCCATGTTC

TTGATCCTCTTCATCCTTTCCATTACTAGTGTAGCCACCACCAATGCAATTGCATCAAAA

GTAGATCGGTTCGTGCCTCAAGACAACTACCTCCTAAGCTGCGGGGCATCAGCTGCTGTG

CAGGTTGACGATGGCAGGACATTCCGCTCTGATCCTGAGTCGGTATCGTTTCTGTCAACC

CCGACGGACATCAAGATCGCCGCTAAAGCATCCCTGGCTTCTGCTTCACCATTATCCCCA

CTTTACCTTGATGCAAGAGTATTCTCTGATATCTCAACCTATAGCTTCTTCATCTCCCAG

CCTGGTCGCCATTGGATCCGGCTCTACTTCTTACCTATCACCGATAGCCAATACAACCTC

ACCACGGCAACATTTTCCGTGTCCACTGATAGCATGGTCCTTCTCCATGATTTCTCCTTC

ATAGCCAGTCCTCCAAACCCTGTGTTTAGGGAATATCTTGTGTCAGCACAGGGAGACAAC

TTAAAGATCATCTTTACCCCAAAGAAAAACTCGATAGCATTCATCAATGCTATCGAGGTC

GTCTCAGCCCCACCAAGCCTTATTCCAAATACCACCACCAGAATGGGTCCTCAGGACCAG

TTTGACATATCTAACAGTGCATTGCAGGTTGTCTACCGGCTGAACATGGGTGGTGCACTG

GTTACATCGTTTAATGACACACTAGGCAGAACCTGGCAGCCAGATGCACCTTTTCTGAAG

CTTGAGGCAGCAGCAGAGGCAGCTTGGGTTCCTCCTAGAACCATCAAGTACCCTGATGAC

AAGACTCTCACACCCCTCATCGCTCCAGCAAGCATCTACTCGACAGCACAGCAGATGGCC

TCAACAAATATCACAAATGCAAGATTCAACATAACATGGCAAATGGCTGCAGAGCCGGGA

TTCAGGTACCTTATCCGCCTACATTTCAGCGACATTGTCAGCAAGACACTCAATAGCCTC

TACTTCAATGTCTATATTAATGGCATGATGGCTGTTGCCAACCTTGATCTATCGAGCCTG

ACAATGGGGCTTGCAGTAGCCTACTACAAGGACTTGATTGCTGAGTCTTCCAGCATCATC

AATTCCACCCTTGTAGTCCAGGTTGGCCCAAACACAATCGACTCCGGCGACCCCAATGCC

ATCCTTAATGGCCTTGAGATCATGAAGATAAGCAATGAAGCAAACAGCTTAGATGGCCTT

TTTTCACCAAAAACAAGCTCAGAAGTTAGTAAGACGACACTGACTGGCATAGCATTTGCT

TTGGCAGCAACAGCTGCATTGGCTGTAGTTATATGCTACAGGCGAAACCGTAAACCAGCA

TGGCAGAGGACAAACAGCTTCCATTCTTGGTTTCTTCCACTGAACTCGTCCTCAAGCTTC

ATGAGCAGTTGCAGCAGGCTCTCCAGAAATCGCTTTGGCTCCACAAGGACCAAGAGTGGA

TTTTCGAGCGTGTTTGCATCCAGTGCTTATGGATTGGGGCGCTATTTCACCTTCGTCGAA

ATTCAGAAAGCCACGAAAAACTTTGAAGAAAAGGGTGTTATTGGTGTTGGTGGCTTCGGA

AAAGTTTATCTTGGTGCTACTGAAGATGGCACACAGCTGGCTATCAAGCGAGGCAATCCA

TCATCTGATCAAGGTATGAATGAGTTTCTGACTGAAATTCAAATGTTATCAAAACTTCGC

CACCGCCACCTGGTTTCACTCATTGGCTGTTGTGATGAGAACAACGAGATGATATTAGTT

TATGAGTTCATGTCAAATGGTCCACTAAGGGATCATCTGTATGGTGACACAAACATCAAG

CCTATTTCTTGGAAGCAGCGCCTTGAAGTTTGCATTGGGGCAGCAAAGGGTCTGCATTAT

CTTCATACAGGTTCAGCTCAGGGCATAATTCACCGTGATGTCAAGACTACCAACATCCTA

CTTGATGAAAATTTTGTCGCCAAGGTCGCTGATTTTGGCCTATCAAAAGATGCTCCATCC

CTCGAACAAACTCATGTGAGCACTGCTGTCAAAGGAAGCTTTGGGTATCTTGATCCAGAG

TACTTCAGACGTCAACAGCTGACAGATAAGTCTGATGTATACTCTTTTGGTGTGGTACTC

TTTGAAGTGCTGTGTGCAAGACCAGCCATCAATCCAGCCCTTCCAAGAGACCAAGTGAAT

CTGGGAGAGTGGGCCCGTACATGGCACCGCAAGGGGGAGCTTGGTAAAATAATTGATCCC

AATATAGCAGGACAGATCAGGCCTGATTCACTTGAGATGTTTGCTGAGGCTGCTGAGAAA

TGCCTTGCTGACTATGGAGTTGACCGGCCAACAATGGGAGACGTGCTATGGAAACTTGAA

TTTGCCTTGCAACTTCAAGAGAAGGGTGATGTTGTTGACGGCGCCAGTGATGGGATCGCA

ATGAAGAGCTTGGAGGTGACCAATGTGGATAGCATGGAGAAATCTGGTAATGCTATCCCA

TCTTATGTACAAGGAAGATGA

>Ta*-CrRLK1L2-*A CDS sequence

ATGGTGCTCCCAACCTTACCGGTTACCCTCACATTCCTCACACTGTTAGCCCTCTTGTCG

ATTGCCAAGGCGGCTGATAACAACTCCACAACCTCTGGCCTCATCCTCCTAAACTGCGGA

GAATCTACCCAAGACGATGATGATGGTGGTCGTTCTTGGGATGGGGACACCGGCTCCATA

TTCGCGCCATCAATGAAAGGAGATGCAGCCATTGCTTTAGGTCAACCCCCTTCACTCACC

CCCAGGGTTCCTTATACAACTGCACGCATCTTTACTTCAAATTACACCTATTCCTTCCCT

GTCAGTCCAGGCCGAATGTTCTTACGCCTATACTTCCTTTCAACTGCTTACGAATACTAT

GCTGTCTCAGATGCCGTCTTCGGAGTCACGGCACGGAATCTTGTCCTCTTAAAAGACTTC

AATGCTTTGCAAACAGCTCAGGCGATCACTTCTGCCTACCTTGTGCGTGAATTCTCGGTG

AATGTTTCTTCAGGCAGCTTGGACCTCACCTTTGCACCATCAGCACATCAGTATGGGTCT

TATGCATTTGTGAATGGCATTGAGATTGTGCCCACGCCTGACATCTTCGCAACACCTGAC

ATAAGATTTGTCAGCGGTGATAACACATCTCCATTCACATTCGACGCTGACATGAGCCTC

CAGACTATGTACCGGCTCAATGTTGGGGGCCCAGCCATTTCCCCGAAAGGTGACTCGGGC

TTTTACCGCTCATGGGCCAATGATGCCCCATACATACTTGGTGGCTTTGGGTTGACCTTT

TGGAAAAATGATAATTTGACTATCAGTTATACATCTAGAGTGCCGAATTACACCGCCCCA

GTTGATGTCTATGGTACAGCTCGGTCCATGGGGCCAACTGCACAGATCAACCTGAACTAC

AACCTTACATGGATTTTACCGGTTGATGCGGGTTTCTTTTACCTCCTAAGGTTTCATTTC

TGTGAGATTAAGTATCCTATTACCAAGGTGAATCAGAGGTCGTTCTTCATCTACATCAAC

AACCAGACAGCGCAGGAGCAAATGGATGTCATCTTCCGGAGCGGAGGAATCGGTAGACCA

ACATACACTGAATATGTTATCATGGCTATTGGTTCTGGTCAGGTGGACATGTGGATTGCA

CTTCACCCTGATCTTTCAAGTAAACCACAGTATTCTGATGCAATACTGAATGGTCTCGAG

GTCTTCAAGCTACAGAATTACGGACCGAGCAATCTTGCTGGGCTCAATCCTCCACTTCCG

CAAAAGCCTGATGTGAATCCTAATAGGCTATCTAGCGGTGAAAGAAAAACCAAAGGTGGC

ATACAAGCAACCATCGGTGGTACTGCTGGTGGTTTTGCTTTATTGTTGATTGCCCTTTTC

AGCATGTGTGTTATCTACAGACGGAAGAAGGCAGCGAAGAGTCCCGGCAAGACCGACTAT

GGACATGTGAAGCATCCAACTAAATGCATAAAGTCTACATGTGATCTTGTACGTCATTTC

TCATTTGCTAAAATTCAAGTTGCCACCAAAGACTTTGATGAAGCACTTATCATCGGCAGA

GGCGGTTTCGGGAATGTCTACATCGGCGATATAGATGGAGGGACAAAGGTGGCGATCAAG

CGATGTGACCAGAAATCCCAACAAGGCTTTCATGAGTTCCAGACTGAAATCGAGATGCTG

TGCAATTTCCGCCATCGCCACCTTGTGTCTCTGATTGGCTATTGTGAGGAGAAGAATGAG

ATGATTCTGGTGTATGACTACATGGCTCATGGAACACTGCGTGAGCATTTGTACAACACC

AGGAACCCACCACTGCCGTGGCAGCAGCGCCTTGAGATTTGCATCGGTGCAGCCCGAGGA

CTGCATTACCTCCACACCGGCGTAGAGCAAGGAATCATCCACCGTGACGTCAAGACCACC

AACATCCTACTGGATGATAGGTTAATGGCAAAGGTTTCCGACTTCGGTTTGTTTAAGGCT

AGTCCAGACATTGGCAACACCCACATGAGCACTGCTGTTAAGGGCACCTTTGGATATCTT

GATCTGGAGTACTTCCGGCAGCAGCGTCTCACCAAAAAATCAGATGTGTACTCCTTCGGG

GTTGTGTTGTTTGAGACCCTGTGTGCGCGCCCTGTGATAAATACTGAGCTCCCTTATGAG

CAAGTGAGCTTGCGTGACTGGGTGGTATCTTGCCGAAAGAAAGGTGTACTCGAGGAGATT

GTTGACCCCTGTGTTAAGGAGGAAATCACCCTTGAGTGCTTCAGGATATTTGCAGAGATA

GCGGAGAAATGCGTTGCTGATCGTAGCATAGATAGGCCATCAATGGGTGATGTACTTTGG

AACCTTGAGGTCGCACTCCAGCTTCAGGATAGTGCAAGCTACAACACCAGCTGTGCTGAG

GGTGCATCATCTCTTCAGATCAGCGGAGTGCATTCAGGCAAACCATCAACCAACTCAACA

ATTAGCGTTGCAGCACAGGAAGCCATATTTTCAGATATTGCACATCCAGAAGGCCGATAA

>Ta*-CrRLK1L2-*B CDS sequence

ATGGTGCTCCCAACCTTACCGGTTACCCTCACATTCCTCACACTGCTAGCTCTCTTGTCG

ATTGCCAAGGCGGCTGATAACAACTCCACAACCTCTGGCCTTATCCTCCTAAATTGCGGA

TCATCAACCCAAAACGATGATGATAGTGGTCGTACTTGGGATGGGGACACCGGCTCCAAA

TTCGCGCCATCAATGAAAGGAGTTGCAGCCATTGCTTTAGGCCAAACCCCTTCACTCACC

CCCAGGGTTCCTTATACAACTGCACGCATCTTTACTTCAAATTACACCTATTCCTTCCCT

GTCAGTCCAGGCCGAATGTTCTTACGCCTATACTTCTTTTCAACTGCTTACGAATACTAT

GCTGTCTCAGATGCCGTCTTCGGAGTCACGTCACGGAATCTTGTCCTCTTAAATGACTTC

AATGCTTTGCAAACAGCTCAGGCGATCACTTCTGCCTACCTTGTGCGTGAATTCTCGGTG

AATGTTTCTTCAGGCAGCTTGGACCTCACCTTTGCACCATCAGCACAACAGTATGGGTCT

TATGCATTTGTGAATGGCATTGAGATTGTGCCCACGCCTGACATCTTCGCAACACCTGAC

ATAAGATTAGTCAGCGGTGATAACACATCTCCATTCACATTCGATGCTGACATGAGCCTC

CAGACTATGTACCGGCTCAATGTCGGGGGCCCAGCCATTTCCACGGAAGGTGACTCGGGC

TTTTACCGCTCATGGGCCAATGATGCCCAATACATACTTGGTGGCTCTGGGTTGACCTTT

TGGAAAAATGATAATTTGACTATCAGTTATACATCTAGAGTGCCGAATTACACCGCCCCA

GTTGATGTCTATGGTACAGCTCGGTCGATGGGGCCAACTGCACAGATCAACCTGAACTAC

AACCTTACATGGATTTTTCCGGTTGATGCAGGTTTCTTTTACCTCCTAAGGTTCCATTTC

TGTGAGATTAAGTATCCTATTACCAAGGTGAATCAGAGGTCATTCTTCATCTACATCAAC

AACCAGACAACGCAGAAGCAAATGGATGTCATCGTCCGGAGCGGAGGAATCGGTAGACCA

ACGTACACTGAATATGTTATCATGGCTATTGGTTCTCGTCAGGTGGACATGTGGATTGCA

CTTCACCCTGATCTTTCAAGTAAACCACAGTATTCGGATGCAATTCTGAATGGTCTCGAG

GTCTTCAAGCTACAGAATTACGGACCGAGCAATCTTGCTGGGCTCAGTCCTCCACTTCCG

CAAAAGCCTGATGTGAATCCTACTAGGCTATCTAATGGTGAAAGAAAATCCAAAGGTGGC

ATACAAGCAATCATCGGTGGTACTACTGGTGGTTTTGCTTTATTGTTGATTGCCCTTTTC

AGCATGTGTGTTATATACAGACGGAAGAAGGTAGCGAAGAGTCCCGGCAAGACCGACTAT

GGACATGTGAAGCATCCAACTAAATGCATAAAGTCTACATGTGATCTTGTACGTCATTTC

TCATTTGCTAAAATTCAAGTTGCCACCAAAGACTTTGATGAAGCACTTATTATCGGCAGA

GGCGGTTTCGGGAATGTCTACATCGGCGATATAGATGGAGGGACAAAGGTGGCAATCAAG

CGATGTGACCAGAAATCCCAACAAGGCTTTCATGAGTTCCAGACTGAAATCGAGATGCTG

TGCAATTTCCGCCATCGCCACCTTGTGTCTCTGATTGGCTATTGTGAGGAGAAGAATGAG

ATGATTCTGGTGTATGACTACATGGCTCATGGAACACTGCGTGAGCATCTGTACAACACC

AGGAACCCACCACTACCGTGGCAGCAGCGCCTTGAGATTTGCATCGGTGCAGCCCAAGGA

CTGCATTACCTCCACACCGGCGTAGAGCAAGGAATCATCCACCGTGACGTCAAGACCACC

AACATCCTACTGGATGATAGGTTAATGGCAAAGGTTTCCGACTTCGGTCTGTCTAAGGCT

AGTCCAGACATTGGAAACACCCACATGAGCACTGCTGTGAAGGGCACCTTTGGATATCTT

GATCCGGAGTACTTCCGGCTGCAGCGTCTCACCAAAAAATCAGATGTGTACTCTTTCGGG

GTCGTGTTGTTTGAGACCCTGTGTGCGCGCCCTGTGATAAACACTGAGCTCCCTTATGAG

CAAGTGAGCTTGCGTGACTGGGCGCTATCTTGCTGGAAGAACGGTGTACTTGAGGAGATT

GTTGACCCCCGTGTTAAGGAGGAAATCACCCCTGAGTGCTTCAGGGTTTTTGCAGAGATA

GCAGAGAAATGTGTAGCTGATCGTAGCATAGAGAGGCCATCAATGGGTGATGTACTTTGG

AACCTTGAGGTCGCACTCCAGCTGCAGCAGGCTAGTGCAAGCTACAACAGCAACCGTGCA

GAGGGTGCTTCATCTCTTCAGATCAGCGCGGTGCATTCAGACAAACCATCCACCAACTCA

ACAATTAGCATCGCAGCACAGGAAGCCATATTTTCAGATATTGCGCATGCAGAAGGCCGA

TAA

>Ta*-CrRLK1L3-*A CDS sequence

ATGCAAACATCCTGTAGCAAGTTGATCCGATGGAGTCCCCAATTCTTTGATTCCGGTGCA

CCAACCGCCGCAAATTCCAAAATGGCTTTTCCAGCCCTACCAGCTACCCTCACATGCCTC

ACACTGTTAGCTCTCTTGTCGCTGGCCATGGCGGCTGATAACAACTCCACCGGCCTCATC

CTCATAAACTGCGGAGCATCAGTCCAAGAAGACGATGATAATGGTCGTACTTGGGACGGA

GACACCGGCTCCAAGTTCGCGCCATCACTGAAAGGAGTTACAGCCACTGCTCCAAACCAA

GACCCTTCACTCCCCTCCACCGTCCCTTTTATGACCGCGCGCATCTTCGCTTCAAACTAC

ACCTATTCCTTCTCTGTCACCCCAGGCCGCGTGTTCTTACGCCTCTACTTCTATCCGGTT

GCTTATCCAAACTACGCCGTCGCAGATGCCTTCTTCAGTGTCACGACACCGAATCTTGTC

CTCTTAAATGATTTCAATGCTTCGCAAACAGTTCAGGCGATCAGTTCTGCCTACCTTGTG

CGCGAGTTCTCGGTGAATGTTTCTTCAGGCAGCTCCTTGGACCTCACCTTTGCCCCATCA

GCACATCACAATGGTTCTTACGCGTTTGTGAACGGCATTGAGATTGTTTCCACTCCTGAC

ATCTTCACAGCACCTGACACAAGATATGTCGGTGATAACACATCTCCATTCACATTCGAC

TCTGCCATGGCCGTCCAGACTATGTACCGGCTCAATGTCGGGGGCCAAGCCATTTCCCCG

AAAGGTGACTCGGGCTTCTACCGCTCATGGGCCAATGATGCCCCCTACATATTTGGTGGC

TCTGGGGTGACCTTCTCCAAGGATGACAATTTGACTATCACCTATACATCCAAAGTGCCG

AATTACACGGCGCCAGTTGATGTCTATGGTACAGCTCGGTCGATGGGGCCAACTGCACCG

ATCAACCTGAACTACAACCTTACATGGATTTTACCGGTTGATGCGGGGTTCAGTTACCTC

CTGAGGTTCCATTTCTGTGAGATTCAGTATCCTATTACAAAGCAGAATCAGAGGTCCTTC

TTCATCTACATCAACAACCAGACAGCGCAGGAGCAAATGGATGTCATCGTCTGGAGCGGC

GGAATCGGTAGAACAACATACACAGACTATGTTATCCTGACTGCTGGCTCCGGCCAGGTG

GACATGTGGATTGCACTTCACCCTGATCTTTCAAGTAGACCAGAGTATTTTGATGCAATA

CTGAATGGTCTTGAGATCTTCAAGCTACAGAATTACGGAGCATCGAACAATCTTGCCGGG

CTCAATCCTCCACTTCCACAAAAGCCAGCTGATGCCAGTCCCGGCGCGGCATCTGGCAAA

GTGAAATCTGTCGCGGCTATCATAGGTGGAGCTGTTGGTGGTTTCGTAGTGCTGCTGGTC

ACATGTTTTGGCATTTGCATCATCTGCAAACGAAAGAACAAGAGCAAGAAGAAGAAGAAG

ATATCCAAGGATCCTGGTGGTAAATCTGAAGATGGTCACTGGACTCCTCTCACCGAGTAC

AGCGGATCACGATCAGCTATGTCGGGAAACACGGCCACCACCGGGTCGACACTGCCATCC

AACCTCTGCCGCCACTTCACTTTCGCCGAGCTTCAGACCGCCACCAAGAACTTCGACCAG

GCCTTCCTGCTCGGCAAAGGTGGGTTCGGGAACGTGTACCTCGGGGAGATCGACAGCGGC

ACCAAGGTGGCGATCAAGCGGTGCAACCCGATGTCGGAGCAGGGCGTCCATGAGTTCCAG

ACGGAGATCGAGATGCTGTCCAAGCTCCGGCACCGCCACCTCGTGTCCCTCATCGGCTAC

TGCGAGGACAAGAGCGAGATGATCCTGGTGTACGACTACATGGCCCACGGCACGCTCCGG

GAGCACCTGTACAACACCAAGAACCCGCCGCTGTCGTGGAAGCAGCGGCTGGAGATCTGC

ATCGGCGCCGCCCGGGGGCTCTACTACCTGCACACCGGCGTGAAGCACACCATCATCCAC

CGCGACGTCAAGACCACCAACATCCTGCTGGACGACAAGTGGGTCGCCAAGGTGTCCGAC

TTCGGGCTGTCCAAGACGGGGCCCAACATGGACGCCACCCACGTCAGCACCGTCGTCAAG

GGCAGCTTCGGGTACCTGGACCCGGAGTACTTCCGGCGGCAGCAGCTCTCGGAGAAGTCC

GACGTCTACTCCTTCGGCGTCGTGTTGTTCGAGGTGCTCTGCGCGCGCCCGGCGCTGAGC

CCCTCGCTGCCCAAGGAGCAGATCAGCCTCGCCGACTGGGCGCTGCGCTGCCAGAAGCAG

GGCGTGCTCGGCCAGGTCATCGACCCGGTGCTCCAGGGGAAGATCGCGCCCCAGTGCTTC

CTCAAGTTCACGGACACCGCGGAGAAATGCGTGGCCGACCGCAGCGTCGACAGGCCGTCC

ATGGGCGACGTCCTCTGGAACCTCGAGTTCGCGCTCCAGCTGCAGGAGAGCGAGGAGGAC

ACCGGCAGCCTCACGGAGGGGACGCTGTCGTCGTCGGGCGCGTCGCCCCTCGTCATGACC

AGACTGCAGTCGGACGAGCCGTCGATGGACGCAAGCACGACCACGACGAGCACGACCACG

ATGAGCATGACGGGACGGAGCATCGCGAGCATGGACTCGGACGGGCTGACGCCGAGCGCC

GTCTTCTCGCAGATCATGCATCCGGATGGCAGGTGA

>Ta-*CrRLK1L3***-B** CDS sequence

ATGCAAACATCCTGTAGCAAGTTGATCCGATGGAGTCCCCAATTCTTTGATTCTGGTACA

CCAACCACCGCAAAATCCAAAATGGCTTTCCCAGCCCTACCAGTTACCCTCACATGCCTC

ACACTGTTAGCTCTCTTATCACTCGCCATGGCGGCTGATAACAACTCCACTGGCCTCATC

CTCGTAAATTGCGGAGCATCGACCCAAGAAGCTGATGATAGTGGTCGTACTTGGGTCGGG

GACACCGGCTCCAAGTTCGCGCCATTATTGAAAGGAGTTGCAACCACTGCTCCAAACCAA

GACCCTTCACTCCCCTCCACGGTCCCTTTTATGACTGCACGCATCTTCACTTCAAACTAC

ACCTATTCCTTCTCTGTCAACCCAGGCCGCATGTTCTTACGCCTCTACTTCTATCCGGTT

GCTTATGCAAACTATGCCGTCTCAGATGCCTTCTTCAGTGTCACGACACGGAATCTTGTC

CTCTTAAATGATTTCAGTGCTTCGCAAACAGCTCAGGCGATCACTTCTGCCTTCCTTGTG

CGCGAGTTCTCGGTGAATGTTTCTTCAGGATCCTCCTTGGACCTCACCTTTGCCCCATCA

GCACATCGCAATGGTTCTTACGCATTTGTGAACGGCATTGAGATTGTGCCCACTCCTGAC

ATCTTCACAGCACCTGACACAAGATATGTCGGTGATAACACAGCCCCATTCTCATTCGAC

GCTGGCATGGCCGTCCAGACTATGTACCGGCTCAATGTCGGGGGCCAAGCCATTTCCCCG

AAAGGTGACTCGGGCTTCTACCGCTCATGGGCCAATGATGCCCCCTACATATTTGGTGGC

TCTGGGGTGACCTTCTCCAAAGATGATAATTTGACCATCACCTATACATCCAACGTGCCG

AATTACACGGCGCCAGTTGATGTCTATGGTACAGCTCGGTCGATGGGGCCAACTGCACAG

ATCAACCTCAACTACAACCTTACATGGATTTTACCCGTTGATGCGGGGTTCAGTTACCTC

CTAAGGTTCCATTTCTGTGAGATTCAGTATCCTATTACAAAGCAGAATCAGCGGTCCTTC

TTCATCTACATCAACAATCAGACAGCGCAGGAGCAAATGGATGTCATCGTCTGGAGCGGA

GGAATCGGTAGAACAGCATACACAGACTATGTTATCATGGCTGTTGGTTCTGGTCAGGTG

GACATGTGGATTGCACTTCACCCTGATCTTTCAAGTAAACCAGAGTATTTTGATGCAATA

CTGAATGGTCTTGAGATCTTCAAGCTACAGAATTACGGATCACCGAACAATCTTTCTGGG

CTCAATCCTCCACTTCCACAAAAGCCAACTGATGCCAGTCCCGGCGCGGCGTCTGGCAAA

ATGAAATCTGTCGCGGCTATCATAGGTGGAGCTGTTGGTGGTTTCGCAGTGCTGCTGGTC

ACATGTTTTGGCGTTTGCATCATCTGCAAACGAAAGAACAAGAAGAACAAGAAGAAGATA

TCCAAGGATCCTGGTGGTAAATCTGAAGATGGTCACTGGACTCCTCTCACCGAGTATAGC

GGATCACGGTCAGCTATGTCGGGAAACACGGCCACAACCGGGTCGACGCTGCCATCCAAC

CTCTGCCGCCACTTCACTTTCGCCGAGCTTCAGACCGCCACCAAGAATTTCGACCAGGCC

TTCCTGCTCGGCAAAGGTGGGTTCGGGAACGTGTACCTCGGGGAGATCGACAGTGGCACC

AAGGTGGCGATCAAGCGGTGCAACCCGATGTCCGAGCAGGGCGTCCACGAGTTCCAGACG

GAGATCGAGATGCTGTCCAAGCTCCGGCACCGCCACCTGGTGTCCCTCATCGGCTACTGC

GAGGACAAGAGCGAGATGATCCTGGTGTACGACTACATGGCCCACGGCACGCTCCGGGAG

CACCTCTACAACACCAAGAACCCGCCGCTGTCGTGGAAGAAGCGGCTCGAGATCTGCATC

GGCGCCGCCCGGGGCCTCTACTACCTGCACACCGGCGTGAAGCACACCATCATCCACCGC

GACGTCAAGACCACCAACATCCTGCTGGACGACAAGTGGGTCGCCAAGGTCTCCGACTTC

GGGCTGTCCAAGACGGGGCCCAACATGGACGCCACCCACGTCAGCACCGTCGTCAAGGGC

AGCTTCGGGTACCTCGACCCGGAGTACTTCCGGCGGCAGCAGCTCTCGGAGAAGTCCGAC

GTCTACTCCTTCGGCGTCGTGCTGTTCGAGGTGCTCTGCGCGCGCCCCGCGCTGAGCCCC

TCGCTGCCCAAGGAGCAGATCAGCCTCGCCGACTGGGCGCTGCGCTGCCAGAAGCAAGGC

GTGCTCGGCCAGGTCATGGACCCGGTGCTCCAGGGGAAGATCGCGCCCCAGTGCTTCCTC

AAGTTCACGGACACCGCGGAGAAATGCGTGGCCGACCGCAGCGTCGACCGGCCGTCCATG

GGCGACGTCCTCTGGAACCTCGAGTTCGCGCTCCAGCTGCAGGAGAGCGAGGAGGACACC

GGCAGCCTCACTGAGGGGACACTGTCGTCGTCGGGCGCGTCGCCTCTCGTCATGACCAGA

CTGCAGTCGGACGAGCCGTCGACGGACGCAAGCACCACCACGACGACCACGACCACGATG

AGCATGACGGGACGGAGCATCGCGAGCGTGGACTCGGACGGGCTGACGCCGAGCGCCGTT

TTCTCGCAGATCATGCATCCGGATGGCAGGTGA

>Ta-*CrRLK1L3*-D CDS sequence

ATGGCGTTCCCAGCCCTACCAGTTACCCTCACATGCCTCATACTGTTATCTCTCTTGTCG

CTTGCCATGGCGGCTGATAACAACTCCACTGGCCTCATCCTCGTAAATTGCGGTGCATCA

GTGCAAGGCGACGATGATAGTGGCCGTACTTGGGACGGGGACACCGGCTCCAAGTTCGCG

CCATCATTGAAAGGAGTTGCAGCCACTGCTCCAAACCAAGACCCTTCGCTCCCCTCCACG

GTCCCTTTTATGACCGCACGCATCTTCACTTCAAACTACACATATTCCTTCTCTGTCAAA

CCAGGCCGCATGTTCTTGCGCCTCTACTTCTATCCGGTTGCTTATCCAAACTATGCCGTC

TCAGATGCCTTCTTCAGTGTCACGACGCCGAAACTTGTCCTCTTAAATGACTTCAGTGCT

TCGCAAACAGCTCAGGCGATCACTTCTGCCTTCCTTGTGCGTGAGTTCTCGGTGAATGTT

TCTTCAGGATCCTCCTTGGACCTCACCTTCGCCCCATCTGCACATCGCAATGGTTCTTAC

GCATTTGTGAACGGCATTGAGATTGTGCCCACTCCTGACATCTTCACAGCACCTGACACA

AGAAATGTCGGTGATAACACAGCCCCATTCTCATTCGACACTAGCTCGAGCCTCCAGACT

ATGTACCGGCTCAATGTCGGGGGCCAAGCCATTTCCCCGAAAGGTGACTTGGGGGGCTTC

TACCGCTCATGGGCGAATGACGCCCCGTACATAGCTGGTGGCTCTGGGGTGACCTTCTCC

AAAGATGATAATTTGACCATCACTTATACATCCAAAGTGCCGAAGTACACGGCGCCACCT

GATGTCTATGGTACAGCTCGGTCGATGGGGCCAACTGCACAGATCAACCTCAACTACAAC

CTTACATGGATTTTACCGGTTGATGCGGGGTTCTTTTACCTCCTAAGGTTCCATTTCTGT

GAGATTCAGTATCCTATTATCAAGATCAATCAGAGGTCCTTCTTCATCTACATCAACAAC

CAGACAGCTCAGGAGCAAATGGATGTCATCGTCTGGAGCGGAGGAATCGGTAGAACAACA

TACACGGACTATGTTATCATGGCTGCTGGTTTCGGTCAGGTGGACATGTGGATTGCACTC

CACCCTGATCTTTCAAGCAGACCAGAGTATTTTGATGCAATACTGAATGGTCTGGAGGTC

TTCAAGCTACAGAATTACGGATCACCGAACAATCTTTCTGGGCTCAATCCTCCACTTCCA

CAAAAGCCAGCTGATGCCAGTCCCAGCGCGGCATCTGGCAAAATGAAATCGGTCGCGGCC

ATCATAGGTGGAGCTGTTGGTGGTTTCATAGTGCTACTGGCCGCGTGTTTTGGCGTTTGC

ATCATCTGCAAACGAAAGAACAAGAAGAAGAAGAAGAAGAAGACATCCAAGGATCCTGGT

GGTAAATCTGAAGATGGTCACTGGACTCCTCTCACCGAGTACAGCGGATCACGATCAGCC

ATGTCGGGAAACACGGCCACCACTGGGTCGACACTGCCATCCAATCTCTGCCGCCACTTC

ACTTTCGCCGACCTTCAGACCGCCACCAAGAACTTCGACCAAGCCTTCCTGCTCGGCAAA

GGTGGGTTCGGGAACGTGTACCTCGGGGAGATCGACAGCGGCACCAAGGTGGCGATCAAG

CGGTGCAACCCGATGTCGGAGCAGGGCGTCCATGAGTTCCAGACGGAGATCGAGATGCTG

TCCAAGCTCCGGCACCGGCACCTCGTGTCCCTCATCGGCTACTGCGAGGACAAGAGCGAG

ATGATCCTGGTGTACGACTACATGGCCCACGGCACGCTCCGGGAGCACCTCTACAACACC

AAGAACCCGCCGCTGTCGTGGAAGCAGCGGCTGGAGATCTGCATCGGCGCCGCCCGGGGC

CTCTACTACCTGCACACCGGCGTGAAGCACACCATCATCCACCGCGACGTCAAGACCACC

AACATCCTGCTGGACGACAAGTGGGTCGCCAAGGTCTCCGACTTCGGGCTGTCCAAGACG

GGCCCCAACATGGACGCCACCCACGTCAGCACCGTCGTCAAGGGCAGCTTCGGGTACCTC

GACCCGGAGTACTTCCGCCGGCAGCAGCTCTCGGAGAAGTCCGACGTCTACTCCTTCGGC

GTCGTGCTCTTCGAGGTGCTCTGCGCGCGCCCCGCGCTGAGCCCCACGCTGCCCAAGGAG

CAGATTAGCCTGGCCGACTGGGCGCTGCGCTGCCAGAAGCAGGGCGTGCTCGGCCAGGTC

ATCGACCCGGTGCTCCAGGGGAAGATCGCGCCCCAGTGCTTCCTCAAGTTCACGGACACC

GCGGAGAAATGCGTGGCCGACCGCAGCGTCGACAGGCCGTCCATGGGCGACGTCCTCTGG

AACCTCGAGTTCGCGCTCCAGCTGCAGGAGAGCGAGGAGGACACCGGCAGCCTCACGGAG

GGGACGCGGTCGTCGTCCGGCGCGTCGCCCCTCGTCATGACCAGGCTGCAGTCGGACGAG

CCGTCGACGGACGCAAGCACCACCACGACGTCCACGACCACGATGAGCATGACGGGGCGG

AGCATCGCGAGCATGGACTCGGACGGGCTGACCCCGAGCGCCGTTTTCTCGCAGATCATG

CATCCGGATGGCAGGTGA

>Ta-*CrRLK1L4*-A CDS sequence

ATGGCCGCGACGGCGAGGCTCCGGCGAGCTCGACCTCGCGGCGTGCTCGGGCTCGTGTCG

GCGCTGCTCGTCTGCGGCGCTGCGGCGTACGCGCCGGAGGACAACTACCTCGTCAGCTGC

GGCTCCTCGCTGGACACGCCCGTGGGCCGGAGGCTCTTCCTCGCCGACGACGGCGGCTCC

GGCTCCGGCGCGGTCACCCTGACGTCCCCTCGCAGCGCCGCGGTGAAGGCCTCGCCGGAC

CTGGTGTCCGGCTTCCGCGACGCCGCGCTGTACCAGAACGCCAGGGTCTTCTCCGCGCCT

TCATCATACTCGTTCGCCATCAGGCGCCGCGGCCGGCACTTCCTCCGCCTCCACTTCTTC

CCCTTCGTGTACCGGAGCTACGACCTCGCCGCCGCGGCCAGGGCGTTCAAGGTGTCCACG

CAGGACGCCGTGCTGCTCGAGGACGGCGTCCCGGCGCCCGAGCCCGGCAACGCGTCGACG

TCATCGTCGCCCCAGCCGGCGCGCGTGGAGTTCCTCCTGGACGTCGCGCGCGACACGCTC

GTGGTCTCGTTCGTGCCGCTCGTCGACGGGGGCATCGCCTTCGTCAACGCCGTCGAGGTC

GTCTCCGCGCCCGACGGCCTCGTCGCCGACGCCGCCGAGTCGTCGACGGGCCGCCCAGAG

CCCATCCCCGCCGCGCTGCCGCTGCAGACGGCCTACCGCCTCAACGTGGGCGGCCCGGCC

GTCGCGCCCGACGACGACGCGCTCTGGCGAGAGTGGACCACCGACCTGCGATTCCTCTCC

CATTCCGTGGCCGACGCGGTGACTCGGGAGGTCCGCTACAACGGGACGCCGAACCGCCTA

CCCGGGCAAGCGACGGCGACCGACGCGCCGGACGTCGTCTACGCCACGGCGAGGGAGCTC

GTGATCAACAGCAGCTCATTCGACGGGCAGAAACAGATGGCGTGGCAGTTCGACGTCGAC

GCCAGCTCCAGCTACTTCATCAGGTTCCACTTCTGCGACATCGTCGGCAAGGCTCCCCAC

CAGCTCCACATCAACGCCTACGTCGACGACGCCAGTCACGCCACCGTGCTGACGGACCTT

GACCTCGCCGCCGTCGGCGATGGTGCGCTGGCGTTCCCGTACTACAAGGACTTCGTGTTG

CCTGCTAGCGAAGCCTCCGGGAAACTCGCCGTCCATGTTGGCCCTCTGGCAAATAAGATC

GTGATGCCCGCTGCCATCCTCAATGGGATTGAGATCATGAAGATGCACCTGAGCGCCGGC

TCTGTCGTCGTCGTCGAGCCGGCGGCGGGGGCAGCCAAGTCGCGTTTCGCCGTCCTTCTT

GGCTCCGTGTGCGGACCGCTCGCTTTCGTGTCCATCGCCGTTGCTCTCGCCATTGTCCTC

AGGAAGAAGAGGAAGAAGGAGGGGGAGGAAGAGGAGGAAAGTGATAAGAAGCAGCCGACG

CCGACGCAGAGCCAGTCGTCCACGCCATGGATGCCACTCCTCGGCCGCCTCAGCGTTCGC

GGCGCCATTGCGTCAGGGTCGTCAAGCTTCACTACTGCCGGTAACACTCCGGGAACCAGC

CCCAGGGCTGCCGCCGCGGTGATGCCGAGCTACCGTTTCCCGCTCGCCGTGTTGCAAGAC

GCGACGCGCAACTTCGACGACAGCCTGATCATCGGAGAGGGAGGGTTCGGCAAGGTGTAC

GGCGCCGTGCTCCAGGACGGCACTAAGGTCGCCGTGAAGCGCGCGAGCCCGGAGTCGCGG

CAGGGCGCGCGGGAGTTCCGCACGGAGATCGAGCTGCTGTCCGGGCTGCGCCACCGCCAC

CTTGTGTCGCTCGTCGGCTACTGCGACGAGCGGGAGGAGATGATCCTGCTGTACGAGTAC

ATGGAGCACGGCTCGCTGCGGAGCCGGCTGTACGGCCGCGGCGGCGCGGCGCCGCTGAGC

TGGGCGCAGCGGCTGGAGGCGTGCGCCGGCGCGGCGAGGGGCCTCCTTTACCTGCACACG

GCCGTGGACAAGCCGGTGATCCACCGCGACGTCAAGTCGTCCAACATCCTGCTGGACGGC

GACCTCACGGGCAAGGTGGCCGACTTCGGGCTGTCCAAGGCCGGGCCGGTGCTGGACGAG

ACGCACGTCAGCACGGCGGTGAAGGGCAGCTTCGGGTACGTCGACCCGGAGTACTGCCGG

ACGAGGCAGCTGACGGCCAAGTCCGACGTGTACTCGCTGGGCGTCGTGCTGCTGGAGGCC

GTCTGCGCGCGGCCCGTCGTCGACCCGAGGCTGCCGAAGCCCATGTCGAACCTGGTGGAG

TGGGGGCTGCACTGGCAGGGCAGGGGCGAGCTGGAGAAGATCGTGGATCGGCGCATCGCC

GCCGCGGCGAGGCCGGCGGCGCTGAGGAAGTACGGCGAGACGGTGGCCAGGTGCCTGGCG

GAGCGCGGCGCCGACCGGCCGGCCATGGAGGACGTGGTGTGGAACCTGCAGTTCGTGATG

CGGCTGCAGGAGGGTGACGGCCTGGACTTCTCCGACGTGAGCAGCCTCAACATGGTGACA

GAGCTCACGCCGCCACCTCGCCGTCAGAGAAGCGCAGTTGATAGCGACGGCCTGGCCCTC

TCCGATGTGAGCAGCCTCAACATGGTTACAGAGCTCACGCCGCCACAAACCGGCAGCGTG

GAGGGAGACGGCGTAGCCGACGACGATTTCACCGATGCATCCATGAGAGGGACCTTCTGG

CAGATGGTCAATGTCCGCAGCAGATGA

>Ta-*CrRLK1L4*-B CDS sequence

ATGGCCGCGACGGCGAGGCTCCGGCCGGCACGAGCTCGCGGCGTGCTCTGGAGCGTCTCG

GTGTGGCTCGTCTGCGGCGCTGCGGCGTACGCGCCGGAGGACAACTACCTCGTCAGCTGC

GGCTCCTCGCTGGACACGCCGGTGGGCCGGAGGCTCTTCCTCGCCGACGACGGCGGCTCC

GGCTCCGGCTCCGGCGCGGTCACCCTGACGTCCCCTCGCAGCGCCGCGGTGAAGGCCCCG

CCGGACCTGGTGTCCGGCTTCCGCGACGCCGCGCTGTACCAGAACGCCAGGGTGTTCTCC

GCGCCCTCTTCCTACTCCTTCGCCATCAGGCGCCGCGGCCGGCACTTCCTCCGCCTCCAT

TTCTTCCCCTTCGTGTACCGGAGCTACGACCTCGCCGTGGCGGCCAGGGCGTTCAAGGTG

TCCACGCAGGACGCCGTGCTGCTCGAGGACGGCGTCCCGGCGCCCGAGCCCGGCAACGCG

TCGACGTCGACGTCGCCCCAGCCGGCGCGCGTGGAGTTCCTCCTGGACGTCGCGCGCGAC

ACGCTCGTGGTCTCGTTCGTGCCGCTCGTCGACGGGGGCATCGCGTTCGTGAACGCCGTC

GAGCTCGTCTCCGTGCCCGACGACCTCGTCGCCGACGCGGCGGACTCGTCGACGGGCCGG

CCAGAGCCGATCCCCGCCGCGCTGCCGCTGCAGACGGCCTACCACCTCAACGTGGGCGGC

CCGGCCGTCGCGCCCGACGACGACGCGCTCTGGCGAGAATGGACTACCGACCAGCCCCTC

TCCGATCCTAGGGTCGACGCGGTGACTCGGGAGGTTCGTTACAACAGGACGCTGAACCGC

CTGCCCGGGCAAGCGACGGCGACCGACGCGCCGGACATCGTCTACGCCACGGCGAGGGAG

CTCGTGATCAACCGCAGCTCATTCGACGGGCAGAAACAGATGGCGTGGCAGTTCGACGTC

GACGCGGGCTCCAGCTATTTCATCAGGTTCCACTTCTGCGACATCGTCAGCGAGGCTCCC

CACCAGCTCCACATCAACGCCTACGTCGATGACGCCAGTCACGCCACCGTGCTGACGGAC

CTTGACCTCGCCGCCGTCGGCGATGGTGCGCTGGCGTTCCCGTACTACAAGGACTTCGTG

TTGCCTGCTAGCGAAGCGTCCGGGAAACTCGCCGTCCATGTTGGCCCGTTGGCAAATAAG

ATCGTGATGCCCGCCGCCATCCTCAATGGGATTGAGATCATGAAGATGCACCTGAGCGCC

GGCTCTGTCGTCGTCGTAGAGCCGGCGGCGGGGGCAGCCAAGTCGCGTCTCGCCGTCATT

CTTGGCTCCGTGTGTGGAGCGCTCGCTTTCGTGTCCATCGCCATTGCTCTCGCCATTGTC

CTTAGGAAGAAGAAGGGGGAGGGGGAGGAGGGTGTTAAGGAGCAGCCGACGCCGACGCGG

AGCCAGTCGTCCACGCCATGGATGCCACTCCTCGGCCGCCTCAGCGTTCGCGGCGCCATT

GCATCAGGATCGTCAAGCTTCACTACCGCCGGTAACACTCCGGGAGCGAGCCCGAGGGCT

GCTGCTGCTGCCGCCGCCGCGGTGGTGCCAAGCTACCGTTTCCCGCTCGCCATGTTGCAA

GACGCGACGCGCAACTTCGACGACAGCCTGATCATCGGAGAGGGAGGGTTCGGCAAGGTG

TACGGCGCCGTGCTCCAGGACGGCACCAAGGTCGCCGTGAAGCGCGCGAGCCCGGAGTCG

CGGCAGGGCGCGCGGGAGTTCCGCACGGAGATCGAGCTTCTGTCCGGGCTGCGCCACCGC

CACCTGGTGTCGCTCGTCGGCTACTGCGACGAGCGGGAGGAGATGATCCTGCTGTACGAG

TACATGGAGCACGGCTCGCTGAGGAGCCGGCTGTACGGCCGCGGCGGCGCGGCGCCGCTG

AGCTGGGCGCAGCGGCTGGAGGCGTGCGCCGGCGCGGCGAGGGGCCTCCTGTACCTGCAC

ACGGCCGTGGACAAGCCGGTGATCCACCGCGACGTCAAGTCGTCCAACATCCTGCTGGAC

GGCGACCTCACGGGCAAGGTGGCCGACTTCGGGCTGTCCAAGGCCGGGCCGGTGCTCGAC

GAGACGCACGTGAGCACGGCCGTCAAGGGCAGCTTCGGGTACGTCGACCCGGAGTACTGC

CGGACGAGGCAGCTGACGGCCAAGTCCGACGTGTACTCGCTGGGCGTCGTGCTGCTGGAG

GCCGTCTGCGCGCGCCCCGTCGTCGACCCGAGGCTGCCGAAGCCCATGTCGAACCTGGTG

GAGTGGGGGCTGCACTGGCAGGGCAGAGGCGAGCTGGAGAAGATCGTGGACCGGCGCATC

GCCGCCGTGGCGAGGCCGGCGGCGCTGAGGAAATACGGCGAGACGGTGGCCAGGTGCCTG

GCGGAGCGCGGCGCCGACCGGCCGGCCATGGAAGACGTGGTGTGGAACCTGCAGTTCGTG

ATGCGGCTGCAGGAGGGCGACGGCCTGGACTTCTCCGACGTGAGCAGCCTCAACATGGTG

ACAGAGCTCACGCCGCCTCGCCGTCAGAGAAGCGCGGTCGATTGCGACGGCCTGGACTTC

TCCGACGTGAGCAGCCTCAACATGGTTACAGAGCTCACGCCGCCTAAAACCGGCAGCATG

GAAGGAGACGGTGTAGCCGACGATGACGATTTCACAGACGCATCCATGAGAGGGACCTTT

TGGCAGATGGTCAATGTCCGCAGCAGATGA

>Ta-*CrRLK1L4*-D CDS sequence

ATGGCCGCGACGGCGAGGCTCCGGCCAGCGCGAGCACGCGGCGTGCTCTGGGTCGTCTCG

GTGTTGCTCGTCTGCGGCGCTGCGGCGTACAAGCCTGAGGACAACTACCTCGTCAGCTGT

GGGTCCTCGCTGGACACGCCGGTGGGCCGGAGGCTCTTCCTCGCCGACGACGGCGCCTCC

GGCGCGGTCACCCTGACGTCCCCTCGCAGCGCCGCGGTGAAGGCCCCGCCGGACCTGGTG

TCCGGCTTCCGCGACGCCGCGCTGTACCAGAACGCCAGGGTGTTCTCCGCGCCCTCCTCC

TACTCCTTCGCCATCAGGCGCCGCGGCCGGCACTTCCTCCGCCTCCACTTCTTCCCCTTC

GTGTACCGGAGCTACGACCTCGCCGCGGCGGCCAGGGCGTTCAAGGTGTCCACGCAGGAC

GCCGTGCTGCTCGAGGACGGCATCCCGGCGCCCGAGCCCGGCAACGCGTCGACGTCGACG

TCGCCCCAGCCGGCGCGCCTGGAGTTCCTCCTGGACGTCGCGCGCGACACGCTGGTGGTC

TCGTTCGTGCCGCTCGCCGACGGGGGCATCGCCTTCGTCAACGCCGTCGAGCTCGTCTCC

GTGCCCGACGGCCTCGTCGCCGACGCGGCGGACTCGTCGACGGGCCGGCCGGAGCCCATC

CCCGCCGTGCTGCCGCTGCAGACGGCCTACCGCCTCAACGTGGGCGGCCCGGCCGTCGCG

CCCGACGACGACGCGCTCTGGCGAGAGTGGACCACCGACCTGCGATTCCTCTCCCATTCT

GTGGCCGACGCGGTGACTCGGGAGGTCCGCTACAACGGGATGCTGAACCGGCTGCCCGGG

CAAGCGACGGCGACCGACGCGCCGGACATCGTCTACGCCACGGCGAGGGAGCTCGTGATC

AACGGCAGCTCATTCGACGGGCAGAAACAGATGGCGTGGCAGTTCGACGTCGACACCAGC

TCCAGCTACTTCATCAGGTTCCACTTCTGCGACATCGTCGGCAAGGCTCCCCACCAGCTC

CACATCAACGCCTACGTCGATGACGCCACCGTGAAGCAGGACCTCGACCTCGCCGCCGTC

GGCGATGGTGCGCTGGCGTTCCCGTACTACACGGACTTCGTGTTGCCTGCTAGCGAGGCG

TCCGGGAAACTCGCCGTCCATGTTGGCCCTCTGGCAAATAAGATCGTCATGCCCGCCGCC

ATCCTCAATGGGATTGAGATCATGAAGATGCACCTGAGCGCCGGGTCCGTCGTCGTCGTC

CAGCCGGCGGCGGGGGCAGCCAAGTCGCGTTTCGCCGTCGTTCTTGGCTCCGTGTGTGGA

GCGCTCGCTTTCATATCCGTCGCCGTTGCTCTCGCCATTGTCCTTAGGAAGAAGGAGAAG

GAGAAGGAGGTGGAGGAGGGTGCCAAGGAGCAGCCGACGCCGACGCAGAGCCAGTCGTCC

ACGCCATGGATGCCACTCCTCGGCCGCTTCAGCGTTCGCGGCGCCATTGCGTCAGGATCG

TCAAGCTTCACCACTGCCGGGAACACTCCGGGAGCGAGCCCCAGGGCTGCTGCCGCTGCC

GCGGTGATGCCGAGCTACCGTTTCCCGCTCGCTATGTTGCAAGACGCGACGCGCAACTTC

GACGACAGCCTCATCATCGGAGAGGGAGGGTTCGGCAAGGTGTACGGCGCCGTGCTCCAG

GACGGCACCAAGGTCGCCGTGAAGCGCGCGAGCCCGGAGTCGCAGCAGGGCGCGCGGGAG

TTCCGCACGGAGATCGAGCTGCTGTCCGGGCTGCGCCACCGCCACCTGGTGTCCCTCGTC

GGCTACTGCGACGAGCGGGAGGAGATGATCCTGCTGTACGAGTACATGGAGCACGGCTCG

CTGAGGAGCCGGCTGTACGGCCGCGGCGGCGCGGCGCCGCTGAGCTGGGCGCAGCGGCTG

GAGGCGTGCGCCGGCGCGGCGAGGGGCCTCCTGTACCTGCACACGGCCGTGGACAAGCCG

GTGATCCACCGCGACGTCAAGTCGTCCAACATCCTTCTGGACGGCGACCTCGCGGGCAAG

GTGGCCGACTTCGGGCTCTCCAAGGCCGGGCCGGTGCTCGACGAGACGCACGTCAGCACG

GCGGTGAAGGGCAGCTTCGGGTACGTCGACCCGGAGTACTGCCGGACGAGGCAGCTGACG

GCCAAGTCCGACGTGTACTCGCTGGGCGTCGTGCTGCTGGAGGCCGTCTGCGCGCGCCCC

GTCGTCGACCCGAGGCTGCCGAAGCCCATGTCGAACCTGGTGGAGTGGGGGCTGCACTGG

CAGGGCAGGGGCGAGCTGGAGAAGATCGTGGACCGGCGCATCGCGGCCGCGGCGAGGCCC

GCGGCGCTGAGGAAGTACGGCGAGACGGTGGCCAGGTGCCTGGCGGAGCGGGGCGCCGAC

CGGCCGGCCATGGAGGACGTGGTGTGGAACCTGCAGTTCGTGATGCGGCTGCAGGAGGGC

GACGGCCTGGACTTCTCCGACGTGAGCAGCCTCAACATGGTGACAGAGCTCACGCCGCCT

CGCCGTCAGAGAAGCGCGGTTGATCACGACGGGCTGGACTACTCCGACGTGAACAGCCTC

AACATGGTTACAGAGCTCACGCCGCCTCAAACCGGCAGCGTGGAGGGAGACGGCGAAGCC

GATGATGATTTCACAGACGCATCCATGAGAGGGACCTTCTGGCAGATGGTCAATGTCCGC

AGCAGATGA

>Ta-*CrRLK1L5*-A CDS sequence

ATGAGAGGAGGCCCGAGATGCGCGCTCCTGCTCCTCGTGGCCGCCGCGGCGCTTGTCCCC

GCGGCGCGGGCGCAGGGGGCGACCGCGCCCGCGCCCTCGGCGGGGGCCCCGTTCGTGCCG

CGGGACGACATCCTGCTCGACTGCGGCGCCACGGGGAAGGGGAACGACACGGACGGCCGC

CAGTGGGACGGCGACGCCGGGTCCAAGTACGCGCCGCCCAACCTCGCCTCGGCCAGCGCC

GGGGCGCAGGACCCCTCCGTGCCGCAGGTGCCCTACCTCACCGCGCGGGTCTCGGCGGCG

CCCTTCACCTACTCCTTCCCGCTCGGCCCCGGCCGCAAGTTCCTCAGGCTGCACTTCTAC

CCGGCCAACTACTCCAACCGCGACGCCGCCGACGCCTTCTTCTCCGTCTCCGTCCCGGCC

GCCAAGGTCACGCTCCTCTCCAACTTCAGCGCCTACCAGACCACCACGGCGCTCAACTTC

GCCTACATCGTGCGCGAGTTCTCCGTCAACGTCACCGGCCAGAACCTCGACCTCACCTTC

ACCCCGGAGAAGGGCCACCCCAACGCCTACGCCTTCATCAACGGCATCGAGGTCGTCTCC

TCCCCTGACCTCTTTGGCATCGCCACGCCGCAATTCGTCACCGGTGATGGCAACAGCCAG

CCATACGAGATGGATCCTGCTGCTGCTCTGCAGACCATGTATCGGCTCAACGTCGGAGGC

CAGGCCATCTCCCCTTCCAAGGACTCCGGCGGGGCTCGGTCATGGGACGACGACACGCCT

TACATCTATGGTGCAGGGGCTGGGGTATCGTACCAGAACGATCCCAATGTCACAATCATC

TACCCTGACAATGTGCCGGGATATGTGGCACCTTCGGATGTCTACGCCACCGCGCGATCA

ATGGGGCCAGACAAGGGTGTAAACATGGCCTACAATCTCACCTGGATATTGCAGGTGGAT

GCTGGGTACCAATACCTTGTGAGGCTCCATTTCTGTGAGATACAATCCCCATTTACTAAA

CCCAATCAGCGGGTGTTCAACATCTACCTCAACAACCAGACTGCCATTGAAGGTGCTGAT

GTGATCCAGTGGGCGGATCCCAATGGTATTGGTACCCCAGTGTACAAGGACTATGTGGTG

AGCACTGTGGGTTCTGGGATTTTGGATTTCTGGGTGGCTCTACACCCAGATGCAGAGACG

AAGCCACAGTACTATGATGCTATTCTCAATGGGATGGAGGTGTTCAAGCTGCAACTTACT

AATGGGAGCCTCGTGGGGCTCAATCCTGTCCCAAGTGCTGATCCACCAGCGCATAGCGGG

TCAGGAGACAAGAAATCTAAAGTCGCGCCTATTGTTGGTGGAGTAATTGGAGGTTTGGCA

GTGCTTGCGCTTGGATATTGCTGCTTCATCTGCAAGCGTCGGAGGAAAGCGGCCAAGGCT

AGCGGCATGAGTGACGGCCATTCTGGTTGGCTGCCATTGTCGCTGTATGGCCATTCACAC

ACTTCAAGCTCAGCCAAGTCGCATGCTACAGGGAGTTATGCTTCATCTTTGCCGTCCAAC

CTGTGCCGCCATTTCTCCTTTGCAGAGATCAAGGCCGCAACCAAAAACTTTGATGAGTCA

CGGATCCTTGGCGTTGGTGGGTTCGGTAAAGTCTACCATGGAGAGATTGACGGGGGCACA

ACTAAGGTGGCTATCAAGCGTGGCAATCCCTTGTCTGAGCAGGGCATACATGAGTTCCAG

ACTGAAATTGAAATGTTGTCAAAGCTCCGGCACCGTCATCTTGTGTCGCTGATTGGTTAC

TGCGAGGAGAAGAACGAGATGATCCTGGTCTATGACTACATGGCTCATGGAACTCTGCGT

GAGCACCTATACAAGACCCAGAATGCACCGCTTAGCTGGAGGCAGCGTTTGGAGATCTGC

ATCGGTGCAGCTCGTGGGCTTCACTACCTTCACACCGGTGCAAAGCACACCATTATCCAC

CGTGATGTGAAGACGACAAACATCCTCCTGGATGAGAAATGGGTTGCCAAGGTTTCAGAT

TTTGGTCTGTCCAAGACTGGGCCATCGATGGATCACACACATGTGAGCACAGTTGTCAAG

GGCAGTTTTGGTTATCTAGATCCTGAATATTTCCGCAGGCAGCAGCTCACTGAGAAATCT

GATGTGTATTCGTTTGGCGTGGTGCTGTTCGAGGTCCTTTGTGCTCGGCCTGCCTTGAAC

CCCACTCTTGCAAAGGAAGAAGTTAGCTTGGCAGAGTGGGCACTGCACTGCCAAAAGAAG

GGAATTCTTGATCAGATTGTTGATCCCTACCTGAAGGGAAAGATTGTTCCGCAGTGCTTC

AAGAAGTTTGCCGAGACGGCTGAGAAGTGTGTTGCCGACAATGGCATCGAGCGCCCTTCG

ATGGGAGATGTGCTTTGGAACTTGGAGTTTGCTCTTCAGATGCAGGAAAGCGCGGAGGAG

AGTGGAAGCATTGGGTGCGGGATGTCAGATGAGGGCACTCCCCTCGTGATGGTTGGAAAG

AAGGATCCCAATGACCCATCAATTGATTCCAGCACCACTACGACCACAACAACTTCCCTA

AGCATGGGTGACCAAAGTGTCGCGAGCATCGATTCGGATGGGCTGACGCCGAGCGCGGTG

TTCTCACAGATCATGAACCCCAAGGGGCGGTGA

>Ta-*CrRLK1L5*-B CDS sequence

ATGAGAGGAGGCCCGAGATGCGCGCTCCTGCTCCTCGCGGCTCTGGCCGCCGCCGCGGCG

CTTGCCCCCGCGGCGTGGGCGCAGGGGGCGACCGCGCCAGCGCCCTCGGCGGGGCCCCCG

TTCGTGCCGCGGGACGACATCCTGCTCGACTGCGGCGCCACGGGGAAGGGGAACGACACG

GACGGCCGCCAGTGGGACGGCGACGCCGGGTCCAAGTACGCGCCGCCCAACCTGGCCTCG

GCCACCGCCGGGGCGCAGGACCCCTCGGTGCCGCAGGTGCCCTACCTCACCGCGCGGGTC

TCGGCGGCGCCCTTCACCTACTCATTCCCGCTCGGCCCCGGTCGCAAGTTCCTCAGGCTG

CACTTCTACCCGGCCAACTACTCCAATCGCGACGCCGCCGACGCCTTCTTCTCCGTCTCC

GTCCCGGCCGCCAAGGTCACGCTCCTCTCCAACTTCAGCGCCTACCAGACCACCACGGCG

CTCAACTTCGCCTACATCGTACGCGAGTTCTCTGTCAACGTCACCGGCCAGAACCTCGAC

CTCACCTTCACCCCGGAGAAGGGCCACCCCAACGCCTACGCCTTCATCAACGGCATCGAG

GTCGTCTCCTCCCCCGACCTCTTTGACCTCGCCACGCCGCAATTAGTCACCGGTGACGGC

AACAGCCAGCCATACGAGATGGATCCTGCTGCTGCTCTGCAGACCATGTATCGGCTCAAC

GTCGGAGGCCAGGCCATCTCCCCTTCCAAGGACTCCGGCGGGGCTCGGTCATGGGACGAC

GACACGCCTTACATCTATGGTGCAGGGGCTGGGGTATCGTACCAGAACGATCCCAATGTC

ACAATCACCTACCCTGACAATGTGCCGGGATATGTGGCACCTTCGGATGTCTATGCCACG

GCGCGATCAATGGGGCCAGACAAGGGTGTAAACATGGCCTACAATCTCACCTGGATATTG

CAGGTGGATGCTGGGTACCAATACCTTGTGAGGCTCCATTTCTGTGAGATACAATCCCCA

TTTACTAAACCCAACCAGCGGGTGTTCAACATCTACCTCAACAACCAGACTGCCATGGAA

GGTGCTGATGTGATCCAGTGGGCGGATCCCAATGGTATTGGTACCCCAGTATACAAGGAC

TATGTGGTGAGCACTGTTGGTTCTGGGATTATGGATTTCTGGGTGGCTCTACACCCAGAT

GCAGGGACGAAGCCACAGTATTATGATGCTATTCTCAATGGGATGGAGGTGTTCAAGCTG

CAACTTACTAATGGGAGCCTCGTGGGGCTCAATCCTGTCCCAAGTGCTGATCCACCAGCG

CATAGCGGGTCAGGAGACAAGAAATCTAAAGTCGCGCCTATTGTTGGTGGAGTAATTGGA

GGTTTGGCAGTGCTTGCGCTTGGATATTGCTGCTTCATCTGCAAGCGTCGGAGGAAAGCG

GCCAAGGCTAGCGGCATGAGTGACGGCCATTCTGGTTGGCTGCCATTGTCGCTGTATGGC

CATTCACACACTTCAAGCTCAGCCAAGTCGCATGCTACAGGGAGTTATGCTTCATCTTTG

CCGTCCAACCTGTGCCGCCATTTCTCCTTTGCAGAGATCAAGGCCGCAACCAAAAACTTT

GATGAGTCACGGATCCTTGGTGTTGGTGGGTTCGGTAAAGTCTACCATGGAGAGATTGAC

GGGGGCACAACTAAGGTGGCTATCAAGCGTGGCAATCCCTTGTCTGAGCAGGGCATACAT

GAGTTCCAGACTGAAATTGAAATGTTGTCAAAGCTCCGGCACCGTCATCTTGTGTCTCTG

ATTGGTTACTGCGAGGAGAAGAACGAGATGATCCTGGTCTATGACTACATGGCTCATGGA

ACTCTGCGTGAGCACCTATACAAGACCCAGAATGCACCGCTTAGCTGGAGGCAGCGTTTG

GAGATCTGCATCGGTGCAGCTCGTGGGCTTCACTACCTTCACACCGGTGCAAAGCACACC

ATTATCCACCGTGATGTGAAGACGACAAACATCCTCCTGGATGAGAAATGGGTTGCCAAG

GTTTCAGATTTTGGTCTGTCCAAGACTGGGCCATCGATGGATCACACACATGTGAGCACA

GTTGTCAAGGGCAGTTTTGGTTATCTAGATCCTGAATATTTCCGCAGGCAGCAGCTCACT

GAGAAATCTGATGTGTATTCGTTTGGTGTGGTGCTGTTCGAGGTCCTTTGTGCTCGGCCT

GCCTTGAACCCCACCCTTGCAAAGGAAGAAGTTAGCTTGGCAGAGTGGGCACTGCACTGC

CAAAAGAAGGGAATTCTTGATCAGATTGTTGATCCCTACCTGAAGGGAAAGATTGTTCCC

CAGTGCTTCAAGAAGTTTGCCGAGACGGCTGAGAAGTGTGTTGCCGACAATGGCATCGAG

CGCCCTTCGATGGGAGATGTGCTTTGGAACTTGGAGTTTGCTCTTCAGATGCAGGAAAGC

GCGGAGGAGAGTGGAAGCATTGGGTGTGGGATGTCAGATGAGGGCACTCCCCTCGTGATG

GTTGGAAAGAAGGATCCGAATGACCCATCAATTGATTCCAGCACCACTACGACCACAACA

ACTTCCTTAAGCATGGGTGACCAAAGTGTCGCGAGCATTGACTCGGATGGGCTGACGCCG

AGCGCCGTGTTCTCACAGATCATGAACCCCAAGGGGCGGTGA

>Ta-*CrRLK1L5*-D CDS sequence

ATGAGAGGAGGCCCGAGATGCGCGCTCCTGCTCCTCGTGGCCGCCGCGGCGCTTGTCCCC

GCGGCGCGGGCGCAGGGGGCGACCGCGCCCGCGCCCTCGTCGGGGGTCCCGTTCGTGCCG

CGGGACGACATCCTGCTCGACTGCGGCGCCACGGGGAAGGGGAACGACACGGACGGCCGC

CAGTGGGACGGCGACGCCGGGTCCAAGTACGCGCCGCCGAAGCTCGCCTCAGCCAGCGCT

GGGGCGCAGGACCCATCGGTGCCGCAGGTGCCCTACCTCACCGCGCGGGTCTCGGCGGCG

CCCTTCACCTACTCCTTCCCGCTCGGCCCCGGCCGCAAGTTCCTCAGGCTGCACTTCTAC

CCGGCCAACTACTCCAACCGCAACGCCGCCGACGCCTTCTTCTCCGTCTCCGTCCCGGCT

GCCAAGGTCACGCTCCTCTCCAACTTCAGCGCCTACCAGACCACCACGGCGCTCAACTTC

GCCTACATCGTGCGCGAGTTCTCCGTCAACGTCACCGGCCAGAACCTCGACCTCACCTTC

ACCCCGGAGAAGGGCCACCCCAACGCCTACGCCTTCATCAACGGCATCGAGGTCGTCTCC

TCCCCCGACCTCTTTGACCTCGCCACGCCGCAATTAGTCACCGGTGACGGCAACAGCCAG

CCGTACGAGATGGATCCTGCTGCTGCTCTGCAGACCATGTATCGGCTCAACGTCGGAGGC

CAGGCCATCTCCCCTTCCAAGGACTCCGGCGGGGCTCGGTCATGGGACGACGACACGCCT

TACATCTATGGTGCAGGGGCTGGGGTGTCGTACCAGAACGATCCCAGTGTCGCAATCACC

TACCCTGACAATGTGCCGGGATATGTGGCACCTTCGGATGTCTATGCCACGGCGCGATCA

ATGGGGCCAGACAAGGGTGTAAACATGGCCTACAATCTCACCTGGATATTGCAGGTGGAT

GCTGGGTACCAATACCTTGTGAGGCTCCATTTCTGTGAGATACAATCCCCATACACTAAA

CCCAATCAGCGGGTGTTCAACATCTACCTCAACAACCAGACTGCCATGCAAGGTGCTGAT

GTGATCCAGTGGGCGGATCCCAATGGTATTGGTACCCCAGTGTACAAGGACTATGTGGTG

AGCACTGTGGGTTCTGGGATTATGGATTTCTGGGTGGCTCTACATCCAGATGCAGAAACC

AAGCCACAGTACTATGATGCTATTCTCAATGGGATGGAGGTGTTCAAGCTGCAACTTACT

AATGGGAGCCTCGTGGGGCTCAATCCTGTCCCAAGTGCTGATCCACCAGCGCATAGCGGG

TCAGGAGACAAGAAATCATTAGTCGCGCCTATTGTTGGTGGAGTAATTGGAGGTTTGGCA

GTGCTTGCGCTTGGATATTGCTGCTTCATCTGCAAGCGCCGGAGGAAAGCTGCCAAGGCT

AGCGGCATGAGTGATGGCCATTCTGGTTGGCTGCCGTTGTCGCTGTATGGCCATTCACAC

ACTTCAAGCTCAGCCAAGTCGCATGCTACAGGGAGTTATGCTTCATCTTTGCCGTCCAAC

CTGTGCCGCCATTTCTCCTTTGCAGAGATCAAGGCCGCAACAAAAAACTTTGACGAGTCA

CGGATCCTTGGTGTTGGTGGGTTCGGTAAAGTTTACCATGGAGAGATTGACGGGGGCACA

ACTAAAGTGGCTATCAAGCGTGGCAATCCCTTGTCTGAGCAGGGCATACATGAGTTCCAG

ACTGAAATTGAAATGTTGTCAAAGCTCCGGCACCGTCATCTTGTGTCGCTGATTGGTTAC

TGCGAGGAGAAGAATGAGATGATCCTGGTCTATGACTACATGGCTCATGGAACTCTGCGT

GAGCACCTATACAAGACCCAGAATGCACCGCTTAGCTGGAGGCAGCGTTTGGAGATCTGC

ATCGGTGCAGCTCGTGGGCTTCACTACCTTCACACTGGTGCAAAGCACACCATTATCCAC

CGTGATGTGAAGACGACAAACATCCTCCTGGATGATAAATGGGTTGCCAAGGTTTCAGAT

TTTGGTCTGTCCAAGACTGGGCCATCGATGGATCACACACATGTGAGCACAGTTGTCAAG

GGCAGTTTTGGTTATCTAGATCCTGAATATTTCCGCAGGCAGCAGCTCACTGAGAAATCT

GATGTGTATTCGTTTGGTGTGGTGCTGTTCGAGGTCCTTTGTGCTCGGCCTGCCTTGAAC

CCCACTCTTGCAAAGGAAGAAGTTAGCTTGGCAGAGTGGGCACTGCACTGCCAAAAGAAG

GGAATTCTTGATCAGATTGTTGATCCCTACCTGAAGGGAAAGATTGTTCCGCAGTGCTTC

AAGAAGTTTGCCGAGACGGCTGAGAAGTGTGTTGCCGACAATGGCATCGAGCGCCCTTCG

ATGGGAGATGTGCTTTGGAACTTGGAGTTTGCTCTTCAGATGCAGGAAAGCGCGGAGGAG

AGTGGAAGCATTGGGTGTGGGATGTCAGATGAGGGCACTCCCCTCGTGATGGTTGGAAAG

AAGGATCCCAATGACCCATCAATTGATTCCAGCACCACTACGACCACAACAACTTCCCTA

AGCATGGGTGACCAAAGTGTCGCGAGCATCGACTCGGATGGGCTGACGCCGAGCGCGGTG

TTCTCACAGATCATGAACCCCAAGGGGCGGTGA

>Ta-*CrRLK1L6*-A CDS sequence

ATGGCCGTCCACGTCGTGCTCCCCCTCCTCCTCCTCCTTCTCGTCGCCACGGTCCTCCCT

TACACCGCCCTCGCCGCCTTCTCCCCGGACTTCAAGATCTTCCTCGCGTGCGGCGCGGGA

GCCGACGTGCCCTTCCCGTCCGACAACCCCGCGCGCACCTTCGTGCGGGACGACGGCTAC

CTCTCGCAGGGGGGCGCCGCCGCGGTGTCTGCCAGTGCCAGCTCCAACGCGGCCTCCCCT

CTGTACGCCGCCGCGCGCGCCGACACCTCGGCCTTCTCCTACCGCCTCACCTACCCTGCC

GCGCCGGACGCGTCGTCGTTCCTCGTCCTGCGCCTCCACTTCTTCCCGTTCGTCCCCGCC

TCCTCCTCCTCCACCAGTCTTTCCTCCGCGCGGTTCACCGTCTCGGTCCTCGACGCCTAC

GCCCTGCTGCCCGCCTTCTCGCCGCCGGCCGACGGCGTCGTCAAGGAGTTCTTCGTCCCG

CGCGGAGCCTCCGGCGGCGACTTCACCGTCAGGTTCGCCCCGGAAGCCGGCTCCTCCGCG

TTCGTCAACGCCGTCGAGCTGTTCTCGGCCCCGCCGGAGCTGCTGTGGAACAACACGGCG

GTGCCGGTGGACCCTGTGGGGAGCAATGACCTGCCCGAGTGGCCGCTGGACGCGCTGGAG

ACGGTGTACCGCCTCAACGTCGGCGGGCCCCTGCTGACCAACGGGAACGACACGCTGTGG

CGGACGTGGCTCCCCGACGACCCCTACCTCTTCGGCGCGCCCGGGCAGTCGGTGGTGAAC

AACACCCCCAGCCCGATCATCTACGCCCCGTCCAACGGTTACACACAGGAGGTGGCGCCG

GACGTGGTGTACAAGACGCAGCGCGCGGCGAACGTGACGGACCTCCTGCAGGCGACAACC

CCGGGCTTCAACTTCAACGTCACGTGGACGTTCCCGGCGGATCAGGGGTCCCGCTACCTC

GTCCGCCTCCACTTCTGCGACTACGAGGTGGTCAGCTCCGTCGTCGGCACTGTCATCGTC

TTCAACGTCTACGTCGCGCAAGCCATTGGCACTCCAGACCTCACGCCGAGTGCTCGGGCG

AGGCAGTCGAACGAGGCCTTCTACATCGACTACGCGGCCATGGCGCCGAGAGCCGGGAAC

CTCACCGTCAGCATCGGCAGGTCGAAGAAAAGCAGCAAAGGCGGCATACTGAACGGCCTG

GAGATCATGAAGCTGCAAACCGTTAATCTGAGCTCGACGGGGTCGCACGGCCGGACGAAG

AGAATCGTCATAATCGTGCTCGCGACGGTGCTCGGCGCCGCCGTCCTTGCTTCCGCGGTG

CTCTGCTTTTTCGTCGTGCGGCGGAGGAAGCGGAGGCAGGTGGCGCCGCCGGGGTCGACG

GAGGACAAGGAGAGCACGCAGCTGCCGTGGTCACCGTACACGCAGGAAGGCATCTCCGGC

TGGGCCGACGAGTCGGCGAACCGGTCCAGCGAGGGCACCACCGCCAGGATGCAGAGGGTG

AGCACCAAGCTGCACATCTCGCTGGCGGAGCTCAAGGCCGCCACGGACAACTTCCACGAC

CGCAACCTCATCGGCGTCGGCGGGTTCGGCAACGTGTACAAGGGCGCGCTCGCGGACGGC

ACGCCCGTGGCTGTGAAGCGCGCCATGCGCGCCTCCAAGCAGGGCCTGCCGGAGTTCCAC

ACGGAGATCGTGGTGCTGTCCGGCATCCGGCACCGGCACCTCGTCTCGCTCATCGGCTAC

TGCAACGAGCAGGCGGAGATGATTCTGGTGTACGAGTACATGGAGAAGGGCACGCTGCGG

GGCCACCTGTACGGCGGCTCCGACGACGAGCCGCCGCTCTCGTGGAAGCAGCGGCTCGAG

ATCTGCATCGGCGCCGCCAGGGGCCTGCACTACCTGCACAGCGGCTACTCCGAGAACATC

ATCCACCGCGACGTCAAGTCCACCAACATCCTCCTCGGCACCGACGGCGGCGGCAGCACC

GGCGGCGGCGCGATCATCGCCAAGGTGGCCGACTTCGGTCTCTCGCGCATCGGGCCGTCG

CTGGGGGAGACGCACGTCAGCACGGCCGTCAAGGGCAGCTTCGGGTACCTCGACCCCGAA

TACTTCAAGACGCAGCAGCTCACGGACCGCTCCGACGTCTACTCCTTCGGCGTGGTGCTC

TTCGAGGTGCTCTGCGCGCGCCCGGTCATCGACCAGAGCCTGGACCGCGACCAGATCAAC

ATCGCCGAGTGGGCCGTCAGGATGCACGGGGAGGGGAAGCTCGACAAGATCGCCGACGCC

AGGATCGCCGGCGAGGTGAACGACAACTCGCTGCGCAAGTTCGCCGAGACGGCCGAGAGG

TGCCTGGCCGACTACGGCGCGGACCGGCCGTCCATGGGCGACGTGCTGTGGAACCTCGAG

TACTGCCTGCAGCTGCAGGAGACGCACGTCAACAGGGACGCGTTCGAGGACAGCGGCGCC

GTCGCCACGCAGCTCCCCGCCGACGTGGTCGTGCCGCGGTGGGTGCCATCGTCCACCAGC

CTGCTGATGATGGACGACGCGGACGAGACGGGCCTGAGCATGACCGACCTCGCCGATAGC

CAGGTCTTCTCCCAGCTGAACGCCCGTGGCGAGGGGCGATGA

>Ta-*CrRLK1L6*-B CDS sequence

ATGGCCGTCCACGTCGTACCACCCCTCCTCCTCCTCCTCGCCACGGCCCTCCCGTACTCC

GCCCTCGCCGTTTTCTCCCCGGATTTCTCCTTCTTCCTCGCGTGCGGCGCAGGCGCCGAC

GTCACCTTCCCGTCAGACAACCCCACGCGCACCTTCGTGCGGGACGACGGCTACCTCTCG

CAAGGGCGTCCCGCCGCGGTGTCTGCAAACGCCAGCTCCGGCGCGGCCTCCAACCCTCTG

TACGCCGCCGCGCGCGCCGACAGCTCGGCCTTCTCCTACCGCCTCGCGTACCCCGCCACG

GCGGGCGCGTCGTCGTTCCTCGTCCTGCGCCTCCACTTCTTCCCGTTCGTCCCCGCCTCC

TCCTCCACCAGCCTTTCCTCCGCGCGCTTCACCGTCTCGGTCCTCGACGCCTACGCCCTG

CTGCCCGCCTTCTCGCCGCCGGCCGACGGCGTCGTCAAGGAGTTCTTCGTCCCGCGCGGC

GGGTCGAAAGAATTCACCATCAGGTTCAGCCCGGACGCCGGCTCCTCCGCTTTCGTCAAT

GCAGTCGAGCTGTTCCCAGCCCCGCAGCAGCTGCTGTGGAACGGCTCCAACTCGGTGGTG

CCGGTGGGCGTCCTGGGGAACGACGACTTGGCCCAGTGGCAGCTGGACGCGCTGGAGACG

GTGTACCGCCTCAACGTCGGCGGGCCCAAGGTGACCAGGGAGAACGACACGCTCTGGCGG

ACGTGGCTCCCCGACGGCGCCTACCTCTTCGGCGCCCCCGGGCAGTCGGTGGTGAACAAC

ACCTCCAGCCCGATCATCTACAACCCGCCGAACACAAGGGAGGTGGCACCGGACGTGGTG

TACAGGACGCAGCGCGCGGCGAACGTGACGGACTTCCTGCGGGCGACAACGCCGGGCCTG

AACTTCAACGTCACGTGGACGTTCCCGGCGGAGGCAGGGTCCCGCTACCTCGTCCGCCTC

CACTTCTGCGACTATGAGGTGGTCAGCTCCGTCGTCGGTGTTGGCATCGTCTTCAACGTC

TATGTCGCGCAAGCCATTGGCAGCAGAGACCTCGCGCCGAATGCTCAGGCGACTCAGCCG

AACGAGCCCTTATATCTTGACTACGCGGCCACGGCGCCGAGAGCTGGGAACCTCACCGTC

AGCATTGGCACGTCGTCGAAAAGCAGCGGGGGCGGCATACTGAACGGGCTGGAGATCATG

AAGCTGCAATCCGTCGACCTGAGCTCGCCGGGGTCGCATGCCCTGACGAAGAGAAGCATC

ATCATCATCGTGCTCGCGACGGTGCTCGGCGCCGCCGTCCTTGCGTGCGCGGTGCTCTGC

TTTTTCGTCGTGCGGCGGAGGAAGCGCAGACAGGTGGCGCCGCCGGCGTCGAAGGAGGAT

AAGGAGAGCACGCAGCTGCCGTGGTCACCGTACACGCAGGAAGGCATCTCCGGGTGGGCC

GACGAGTCGACGAACCGGTCCAACGAGGGCACGACCGCCAGGATGCAGAGGGTGAGCACC

AAGCTGCACATCTCGCTGCCGGAGCTCAAGGCCGCCACGGACAACTTCCACGAGCGCAAC

CTCATCGGCGTCGGCGGGTTCGGCAACGTGTACAAGGGCGCGCTCTCCGACGGCACGCCC

GTGGCGGTGAAGCGCGCCATGCGCGCCTCCAAGCAGGGCCTGCCGGAGTTTCAGACCGAG

ATCGTGGTGCTGTCCGGCATCCGGCACCGGCACCTGGTGTCGCTCATCGGCTACTGCAAC

GAGCAGGCGGAGATGATCCTGGTGTACGAGTACATGGAGAAGGGCACGCTGCGGAGCCAC

CTGTACGGCTCCGACGAGCCGGTGCTGTCGTGGAAGCAGCGGCTCGAGATCTGCATCGGC

GCCGCCAGGGGCCTGCACTACCTGCACAGCGGCTACTCGGAGAACATCATCCACCGCGAC

GTCAAGTCCACCAACATCCTCCTCGGGACCGACGACGGCGGCAGCACCGGCGGCGGCGCG

ATCATCGCCAAGGTGGCCGACTTCGGGCTGTCGCGCATCGGGCCATCGCTGGGGGAGACG

CACGTCAGCACGGCGGTGAAGGGCAGCTTCGGGTACCTGGACCCTGAGTACTTCAAGACG

CAGCAGCTCACGGACCGCTCCGACGTCTACTCCTTCGGCGTGGTGCTCTTCGAGGTGCTC

TGCGCGCGCCCGGTCATCGACCAGAGCCTCGACCGCGACCAGATCAACATCGCTGAGTGG

GCCGTGAGGATGCACGGGGAGGGGAAGCTCGACAAGATCGCCGACGCCAGGATCGCGGGT

GAGGTGAACGACAACTCGCTGCGCAAGTTCGCCGAGACGGCGGAGAAGTGCCTCGCGGAC

TACGGCGCCGACCGCCCCTCCATGGGCGACGTGCTGTGGAACCTCGAGTACTGCCTCCAG

CTGCAGGAGACACACGTCAACAGGGACGCCTTCGAGGACAGCGGCGCCGTCGCCACGCAG

CTCCCCGCCGACGTCGTCGTGCCGCGCTGGGTGCCGTCGTCGACGAGTCTGCTCATGATG

GACGACGCGGACGAGACGGGACTGAGCATGACCGAGCTCGCCGACAGCCAGGTCTTCTCC

CAGCTCAACGCTCGCGGCGAGGGACGATGA

>Ta-*CrRLK1L6*-D CDS sequence

ATGGCCGTCCACGTCGTACCACCCCTCCTCCTCCTCCTCCTCGCCACGGCCCTCCCGTAC

ACCGCCCTCGCCGTTTTCTCCCCGGATTTCTCCTTCTTCCTCGCGTGCGGCGCAGGCGCG

GACGTCCCCTTCCCATCCGACAACCCCACGCGCACCTTCGTGCGGGACGACGGCTACCTC

TCGCAAGGGCGCCCCGCCGCGGTGTCTGCCAGTGCCAGCTCCGGCGCGGCCTCCAACCCT

CTGTACGCCGCCGCGCGCGCCGACAGCTCGGCCTTCTCCTACCGCCTCGCGTACCCCGCC

ACGGCGGGCGCGTCGTCGTTCCTCGTCCTGCGCCTCCACTTCTTCCCGTTCGTCCCCGCC

TCCTCCTCCACCAGCCTTTCCTCCGCGCGCTTCACCGTCTCAGTCCTCGACGCCTACGCC

CTCCTGCCTACCTTCTCGCCGCCGGTCGCCGGCGTCGTCAAGGAGTTCTTCGTCCCGCGC

GACGGGTCGAAAGATTTCACCATCAGGTTCACCCCGGACGCCGGCTCCTCCGCGTTCGTC

AACGCCGTCGAGCTGTTCTCGGCCCCGCCGGAGCTGCTGTGGAACAACACGGCGGTACCG

GTGGACCCCGTGGGGAGCAATGACCTGCCCGAGTGGCCGCTGGACGCGCTGGAGACGGTG

TACCGCCTCAACGTCGGCGGGCCCATGGTGACCAAGGAGAACGACACGCTCTGGCGGACG

TGGCTTCCCGACGGCCCCTACCTCTTCGGCGCCCCCGGGCAGTCGGTTGTGAACAGCACC

TCCAGCCCGATCATGTACGACCCGTCCAACGGTTACACACAGGATGTGGCGCCGGACGTG

GTGTACAGGACGCAGCGCGCGGCGAACGTGACGGACCTCCTGGTGGCGACAACGCCGGGC

CTGAACTTCAACGTCACGTGGACGTTCCCGGCGGAGCAGGGGTCCCGCTACCTCGTCCGC

CTCCACTTCTGCGACTATGAGGTGGTCAGCTCCGTCGTCGGTGTTGGCATCGTCTTCAAC

GTCTATGTCGCGCAAGCCATCGGCACTCCAGCCCTCTCGCCAAAGGATCGGGCGAGGCAG

TCGAACGAGGCCTTTTACATGGACTACGCGGCCAGGGCGCCGAGAGCCGGGAACCTCACC

GTCAGCATCGGCTGGTTGCGGCAAAGCAGCGGAGGCGGCATACTCAACGGGCTGGAGATC

ATGAAGCTGCAATCCGCCGACCCGAGCTTGACGGTGTCGCACGGCCTGACGAAGAGAAGC

ATCATCATCATCGTGCTCGCGACGGTGCTCGGCGCCGCCGTCCTTGCGTGCGCGGTGCTC

TGCTTTTTCGTCGTGCGGCGGACGAAGCGGAGGCAGGTGGCGCCGCCGGCGTCGACGGAG

GATAAGGAGAGCACGCAGCTGCCGTGGTCACCGTACACGCAGGAAGGCATCTCCGGCTGG

GCCGACGAGTCGACGAACCGGTCCAGCGAGGGCACCACCGCCAGGATGCAGAGGGTGAGC

ACCAAGCTGCACATCTCGCTGGCGGAGCTCAAGGCCGCCACGGACAACTTCCACGACCGC

AACCTCATCGGCGTCGGCGGGTTCGGCAACGTGTACAAGGGCGCGCTCGCCGACGGCACG

CCCGTGGCGGTGAAGCGCGCCATGCGCGCCTCCAAGCAGGGCCTGCCGGAGTTCCACACG

GAGATCGTGGTGCTGTCCGGCATCCGGCACCGCCACCTCGTGTCGCTCATCGGCTACTGC

AACGAGCAGGCAGAGATGATCCTGGTGTACGAGTACATGGAGAAGGGCACGCTGCGGAGC

CACCTGTACGGCGGCTCCGACGACGAGCCGCCGCTCTCGTGGAAGCAGCGGCTCGAGATC

TGCATCGGCGCCGCCAGGGGCCTGCACTACCTGCACTGCGGCTACTCGGAGAACATCATC

CACCGCGACGTCAAGTCCACCAACATCCTCCTCGGCACCGACGACGGCGGCAGCACCGGC

GGCGGCGCCATCATCGCCAAGGTGGCCGACTTCGGGCTGTCGCGCATCGGGCCATCGCTG

GGGGAGACGCACGTCAGCACGGCGGTGAAGGGCAGCTTCGGGTACCTGGACCCGGAGTAC

TTCAAGACGCAGCAGCTCACGGACCGCTCCGACGTCTACTCCTTCGGCGTGGTGCTCTTC

GAGGTGCTCTGCGCGCGCCCCGTCATCGACCAGAGCCTCGACCGCGACCAGATCAACATC

GCCGAGTGGGCCGTCAGGATGCACGGGGAGGGGAAGCTCGACAAGATCGCCGACGCCAGG

ATCGCCGGCGAGGTCAACGACAACTCGCTGCGCAAGTTCGCCGAGACGGCGGAGAGGTGC

CTGGCTGACTACGGCGCGGACCGGCCGTCCATGGGCGACGTGCTCTGGAACCTCGAGTAC

TGCCTCCAGCTGCAGGAGACGCACGTCAACAGGGACGCCTTCGAGGACAGCGGCGCCGTC

GCCACGCAGCTCCCCGCCGACGTGGTCGTGCCGCGGTGGGTGCCGTCGTCCACGAGCCTG

CTGATGATGGACGACGCGGACGAGGCGGGCCTGAGCATGACCGAGCTCGCCGACAGCCAG

GTCTTCTCCCAGCTCAACGCACGCGGCGAGGGACGATGA

>Ta-*CrRLK1L7*-B CDS sequence

ATGCTGTGGAATAGCTCCGTGACGCCCGTGGGAGCCGTGGTGAAGGACGACATGGACCTG

TGGCAGCGGCAGCCGCTGGAGACGGTCTATCGCCTCAACGTCGGAGGGCCCAAGGTGACC

ATTGAGAACGACACGCTGTGGCGGACGTGGCTGCCCGACGGTCCCTACCTCTACGACGCC

TCCGGGCTGTCGGTGGTGAGCAACACCTCCAACCCGATCATCTACGATTCATCGAACGGA

TACACGAGGGAGGTGGCGCCAGATGTCGTGTACCAGACCCAGCGCATGGCGAACGTGACG

GACTTACTGGCGGCGACAACCCCGGGCCTGAACTTCAACCTCACGTGGACGTTCCCGGCG

GTGAAGGGGTCCCACTACCTCGTCCGCCTCCACTTCTGCGACTACGAGGTGGTCAGCTCC

GTCGTCGGCGTTGGCATCGTCTTCAACGTCTACATCGCGCAGACCATTGGCACTCCAGAC

CTCACGCCGAATGCTCGGGCGACTCAGTCGAACGAGGTCTTTTACATGGACTACGCGGCC

AGGGCGCCGAGCACCGGGAACCTCACGGTGAGCATCGGCTGGTCGTCGAAAAGGAGCGGA

GGTGGGATACTGAACGGGCTAGAGATTATGAGGCTGCCGCCCGTTGATTTGAGCTCGAGG

AGGTACGGCAGGACGAAGAGGACCATTGTCATTACGGTGTCGGCAGTGCTCGGCGCCGCC

GTTCTTGCTTGCGTGGTGCTCTGCTTTTTCGGCGTGCCGTATACGAAGTACAGCGGCTCC

GGCTGGGCTGAGCAGTTCACGAACCGATGGTCCAGAGAGGGCAAGACCAGCGGGTTGCAG

AGTGTGAGCACGAAGCTGCACATCGCTCTCGCGAAGATCAAGGCCGCCACGGACAACTTC

CACGAGCGCAACCTCATCGGCGTGGGCGGGTTCGGGAACGTGTACAAGGGCGTGCTCGTT

GACGGCACGCCAGTGGCGGTGAAGCGCGCCATGCGCGCCTCGCAGCAGGGGTTGCCGGAG

TTCCAGACGGAGATCGTGGTGCTGTCCGGCATCCGGCACCGGCACCTGGTGTCGCTCATT

GGGTACTGCAACGAGCAGGCGGAGATGATACTGGTGTACGAATACATGGAGAAAGGCACG

CTGCGGAGCCACCTGTACGGTTCCGACGAGCCGGCGTTGTCATGGAAGCAGAGGCTGGAG

ATCTGCATCGGCGCGGCGAGGGGCCTGCACTACCTGCACAGAGGCTACGCGGAGAACATC

ATCCACCGTGACGTCAAGTCGACCAACATCCTCCTCGGGAGCGACGGCGGCAGCACCGGT

GGCGTGATCGCCAAGGTGGCCGACTTCGGGCTGTCGCGCATCGGGCCGTCGTTCGGGGAG

ACGCACGTGAGCACGGCGGTGAAGGGCAGCTTCGGGTACCTGGACCCGGGGTACTTCAAG

ACGCAGCAGCTGACGGACCGGTCGGACGTCTACTCCTTCGGCGTGGTGCTGTTGGAGGTG

CTCTGCGCGCGACCTGTGATCGACCAGAGCCTGGACCACGGCCGGATCAACATCGCCGAA

TGGGCCGTGAGGATGCGCAGGGAAGGGCGGCTCGACAAGATGGCCGACCCGAGGATCGCC

GGCGAGGTGGACGAGGAGTCGCTGCTCAAGTTCGCAGAAACCGCTGAGAAGTGCCTGGCG

GAGTGCTGGGTGGACCGGCCGTCCATGGGCGACGTGCTGTGGAACCTGGAGTATTGCCTA

CAGCTGCAGGAGACCAATATCACCGGGGACGGACTCGACGACATGGTACCGTCGTCGACG

AGCTTGTTGATGGACGAGACCGACTTGAGCATGACCAATGTCGCCGACAGCAAGGTATTC

TCCCAGCTGAGCGCCCGCGGCGAGGGACGATGA

>Ta-*CrRLK1L7*-D CDS sequence

ATGGCCGTCCGCGGCATACTCCTCGCCCTCCTCCTCGCCATGGTTCTCCCGCGCGCCATC

CTCGCCGCCTTCTCTCCCGGCTTCCAATATTTCCTCGCATGCGGCGCCAACTCCGCCGTC

TCCTTCCCGTCCGATTCCCCCGCCAACATCTTCGTCCCCGACGCCGCCTACCTCTCGCCC

GCGGGCGCTCCGGCGGTGTCCGCCAGCTCCACCCTCGCCTCCCCGCCAGCTCTGTACGCC

GCCGCGCGCGCGGACATCTCGGCCTTCTCGTACCGCCTCCCTAGCCCCGCCTCGCCAGAC

ACGTCGTCATTCCTCGTCCTGCGCCTCCACTTCTTCCCCTCCTTCCCCGCCACCTCCTCT

CAGTATGTCATCAACATCTTGTCCGCGCGCTTCAACGTTTCGGTCGCCGACGCCTACGCT

CTGCTGTCCTCCTTCTCGCCTCCGGCCGCCGGCGTCGTCAAGGAGTTCTTCGTCCCGCGC

GACCTCTTCGATGGCCACTTCCACGTCACGTTCACCCCGGACGCCGGCTCCACCGCCTTC

GTCAACGCCATCGAGCTGTTCTCGGCCCCGCCGGAGATGCTGTGGAATGGCCCCGTGACG

CCGGTGGGAGCCGTGGTGAAGGACGACATGGACCTGTGGCAGCGGCAGCCGCTGGAGACG

GTCTATCGCCTCAACGTCGGCGGGCCCAAGGTGATAATTGAGAACGACACGCTGTGGCGG

ACGTGGCTGCCCGACGGCCCCTACCTCTACGACGCCTCCGGGCTGTCGGTGGTGAGCAAC

ACCTCCAGCCCGATCATCTACGATTCATCGAACGGATACACGAGGGAGGTGGCGCCGGAC

GTCGTGTACCAGACCCAGCGCATGGCGAACGTGACGGACTTACTGGCGGCGACAACCCCA

GGCCTGAACTTCAATCTCACGTGGACGTTCCCGGCGGTGAAGGGGTCCCGCTACCTCGTC

CGCCTCCACTTCTGCGACTACGAGGTGGTCAGCTCCGTCGTCGGCGTCGGCATCGTCTTC

AACGTCTACATCGCACAGGCCATTGGCACTCCAGACCTCACGCCGAATGCTCGGGCGACT

CAGTCGAACGAGGTCTTTTACAAGGACTACGCGGCCAGGGCGCCGAGCGCCGGGAACCTC

ACGGTGAGCATCGGCTGGTCGTCGAAAAGCAGCGGAGGTGGCATACTGAACGGGCTAGAG

ATTATGAGGCTGCCGCCCGTCGATTTGAGCTCGAGGAGGTACGGCAGGACGAAGAGGACC

ATTGTCATTACGGTGTCGGCAGTGCTCGGCGCCGCCGTTCTTGCTTGCGTGGTGCTCTGC

TTTTTCGGCGTGCCGTACACGAAGTACAGTGGCTCCGGCTGGGCTGAGCAGTTCATGAAC

CGATGGTCCAGAGAGCGCAAGACCGGCGGGATGGAGAGTGTGAGCAGGAAGCTGCACATC

GCGCTCGCGAAGATCAAGGCCGCCACGGACAACTTCCACGAGCGTAACCTCATCGGCGTG

GGCGGGTTCGGGAACGTGTACAAGGGCGTGCTCGGTGACGGCACGCCAGTGGCGGTGAAG

CGCGCCATGCGCGCCTCGCAGCAGGGGTTGCCGGAGTTCCAGACGGAGATCGTGGTGCTG

TCCGGCATCCGGCACCGGCACCTGGTGTCGCTCATCGGGTACTGCAACGAGCAGGCGGAG

ATGATACTGGTGTACGAATACATGGAGAAAGGCACGCTGCGGAGCCACCTGTACGGCTCC

GACGAGCCGGCGTTGTCATGGAAGCAGAGGCTGGAGATCTGCATCGGCGCGGCGAGGGGC

CTGCACTACCTGCACAGAGGCTACGCGGAGAACATCATCCACCGTGACGTCAAGTCGACC

AACATCCTCCTCGGGAGCGACGACGGCAGCACCGGTGGCGTGATCGCCAAGGTGGCCGAC

TTCGGGCTGTCGCGCATCGGGCCGTCGTTCGGGGAGACGCACGTGAGCACGGCGGTGAAG

GGCAGCTTCGGGTACCTGGACCCGGGGTACTTCAAGACGCAGCAGCTGACGGACCGGTCG

GACGTCTACTCCTTCGGCGTGGTGCTGTTGGAGGTGCTCTGCGCGCGACCTGTGATCGAC

CAGAGCCTGGACCACGGCCGGATCAACATCGCCGAATGGGCCGTGAGGATGCGCAGGGAA

GGGCGGCTCGACAAGATGGCCGACCCGAGGATCGCCGGCGAGGTGGACGAGGAGTCGCTG

CTCAAGTTCGCAGAGACCGCTGAGAAGTGCCTGGCGGAGGCCTGGGTGGACCGGCCGTCC

ATGGGCGACGTGCTGTGGAACCTGGAGTATTGCCTACAGCTGCAGGAGACCAACATCACC

GGGGACGAACTCGACGACATGGTGCCGTCGTCGACGAGCTTGTTGATGGACGAGACCGGC

TTGAGCATGACCAATGTCGCCGACAGCAAGGTATTCTCCCAGCTGAGCGCCCGCGGCGAG

GGACGATGA

>Ta-*CrRLK1L8*-A CDS sequence

ATGCCGCCGGTTCCTGACATGCTCGTGCGGCTCCTCGTCGCGTCCGTGCTGCTCGGCGCA

GCCAGTGGCGCGTTTACCCCCGCGGACACCTACCTCGTCCTCTGCGGCACGTCGGCGAGC

GCCACCGTTGCCGCGGGACGGACGTTCGTCGGGGACGCCCGTCTGCCCGCCAAGTCGCTG

GCCGCGCCGCAGAGCGTCGAGGCCAACACGTCGCTGACCGCGGTCGTCCCGTCCGGCGAG

TCGCAGCTTTACCGGTCCGCGCGCGTCTTCACCGCGCCGGCTTCCTACACGTTCGCCGTC

AAGCAGCCCGGCCGGCACTTCGTGCGCCTCCACTTCTTCCCCTTCCCGTACCGGTCCTAC

GACATGGCCGCGGACGCCGCGTTCAACGTGTCCGTGCAGGGCGCGGTGCTCGTCAACGGG

TACGCGCCCAAGAACGGCACGGCGGAGCTCAGGGAGTTCTCGCTGAACGTCACCGGTGCC

ACGCTGGTGATCGCCTTCGCGCCGACGGGGAAGCTCGCGTTCGTGAACGCCATCGAGGTC

GTGTCGGTCCCCGACGAGCTCATCGCCGACACGGCCAGGATGGTGGGCGGGGCCGTCCAG

TACACCGGGCTGTCGACGCAGGCGCTGGAGACGATCCACCGGATCAACATGGGCGTCCCC

AAGATCACGCCCGGCAACGACACGCTGGGGAGGACGTGGCTGCCGGACCAGAGCTTCCAG

CTCAACACCAACCTAGCGCAGCATAAAGACGCCAAGCCCTTGACGATCAAATACGACGAG

AAGTCGGCGCTCTCCTCCGCGTACACGGCGCCGGCGGAGGTCTACGCGACGGCGACGAGG

CTGAGCACGGCGGGCGAGACCAGCACCATCAACGTGCAGTTCAACATCAGCTGGAGGTTC

GACGCCCCGGCCGGGTCGGATTACCTGCTCCGGTTCCACTGGTGCGACATCGTCAGCAAG

GCGGCCATGGGAATGGCCTTCAACGTCTACGTCGGCGGGTCGGTGGTGCTCGAAAACTAC

GAGATTTCGCGTGACACGTTCAACCGGCTGTCCATACCGGTGTACAAGGACTTCCTCCTG

GGCGCCAAGGACGCCAAGGGCGCCATCACCGTGAGCATCGGGTCGTCGACCGAGGACAAC

GCGTTGCCCGACGGCTTCCTCAACGGCCTCGAGATCATGAGGGTAGTCGGGAGCGCCGGC

GCCGGCGCTCCCGCCGCGTCCGCGCGCAGTTCAAAGGTCAAAATCGGGATCATCGCCGGC

TCGGCCGTCTGCGGGGCCACGCTGGTGATGGTGCTCGGGTTCATCGCCTTCAGGACGCTG

CGCGGGAGGGAGCCGGAGAAGAAGCAGCCGTCCGACACCTGGTCGCCCTTCTCGGCGAGC

GCGCTGGGCTCTCGCTCGCGCTCCCGGAGCTTCTCCAAGAGCAACGGGAACACCGTCCTG

CTCGGGCAGAACGGCGCCGGCGCCGGGTACAGGATCCCGTTCGCGGCGCTCCAGGAGGCG

ACCGACGGGTTCGACGAGGCGATGGTCATCGGCGAGGGCGGGTTCGGGAAGGTGTACAAG

GGCACGATGCGCGACGAGACGCTGGTGGCCGTGAAGCGCGGCAACCGGCGGACGCAGCAG

GGGCTGCACGAGTTCCACACGGAGATCGAGATGCTGTCCCGGCTGCGCCACCGCCACCTG

GTCTCGCTCATCGGCTACTGCGACGAGCGCGGGGAGATGATCCTGGTGTACGAGTACATG

GCCATGGGCACGCTGCGGAGCCACCTGTACGGCGCCGGCCTCCCGCCGCTGTCGTGGGAG

CAGAGGCTCGAGGCCTGCATCGGCGCCGCGCGGGGGCTGCACTACCTCCACACCGGCTCC

GCCAAGGCGATCATCCACCGGGACGTCAAGTCGGCCAACATCCTCCTGGACGAGAGCTTC

ATGGCCAAGGTGGCCGACTTCGGGCTGTCCAAGAACGGGCCGGAGCTGGACAAGACGCAC

GTGAGCACCAAGGTGAAGGGCAGCTTCGGGTACCTGGACCCGGAGTACTTCCGGCGGCAG

ATGCTGACGGAGAAGTCGGACGTCTACTCCTTCGGCGTGGTCCTGCTGGAGGTGCTGTGC

GCCCGCACGGTCATCGACCCGACGCTGCCCCGGGAGATGGTGAGCCTGGCCGAGTGGGCG

ACGCCGTGTCTCAGGAACGGTCGGCTCGACCAGATCGTCGACCAGAGGATCGCCGGGACG

ATACGGCCGGGGTCGCTCAAGAAGCTCGCGGACACGGCCGAGAAGTGCCTCGCCGAGTAC

GGGGTGGAGCGGCCCACCATGGGGGACGTGCTCTGGTGCCTCGAGTTCGCGCTGCAGCTG

CAGGTGGGGTCGTCAGACGGCTCGGACGTCGACACCATGTTGCCGCCGGCGCCGCCTGTG

CCCGTGAAAACGCCAGAGGTTCAGCGTAGCCTGTCCGCCGCTACCATGGCGACCGACGCT

GCTGCCATGACCACCAACTTGGGTGATCTAGACGGAATGTCCCTGAGCGGAGTATTCTCG

AAGATGATCAAGAGCGACGAGGTCAGGTGA

>Ta-*CrRLK1L8*-B CDS sequence

ATGCCGCCGTTTCCTGACATGCTCGTGCGGCTCCTCCTCGCGTCCGTGCTGCTCAGCGCA

GCCAGTGGCGCGTTTACCCCCGCGGACAACTACCTCGTCATCTGCGGCACGTCGGCGAGC

GCCACCGTCGCCCCGAGACGTTCGTTCGTCGGGGACGCCCGCCTGCCCGCCAAGTCGCTG

GCCGCGCCGCAGAGCGTCGAGGCCAACACGTCGCTGACCGCGGTCGTCCCGTCCGGCGAG

TCGGAGCTTTACCGGTCCGCGCGAGTCTTCACCGCACCAGCTTCCTACACGTTCGCCGTC

AAGCAGCCCGGCCGGCACTTCGTGCGCCTCCACTTCTTCCCCTTCTCGTACCGGTCCTAC

GACATGGCCGCGGACGCCGCGTTCAACGTGTCCGTGCAGGGCGCGGTGCTCGTCAACGGG

TACACGCCCAAGAACGGCACGGCAGAGCTCAGGGAGTTCTCCGTGAACGTCACCGGGGGA

ACGCTGGTGATCGCGTTCGCGCCGACGGGGAAGCTCGCGTTCGTGAACGCCATCGAGGTC

GTGTCCGTCCCCGACGAGCTCATCGCCGACACGGCCAGGACGGTGGGCGGCGCCGTCCAG

TACACCGGGCTGTCGACGCAGGCGCTGGAGACGATCCACAGGATCAACATGGGCATTCCC

AAGATCACGCCCGGCAACGACACGCTGGGGAGGACGTGGCTGCCGGACCAGAGCTTCCAG

CTCAACACCAACTTGGCGCAGCACAAAGACGCCAAGCCCTTGACGATCAAATACGACGAG

AAATCGGCACTCTCTTCCCCGTTCACGGCGCCGGCGGAAGTCTACGCGACGGCGACGAGG

CTGAGCACAGCGGGCGAGACCAGCACCATCAACGTGCAGTTCAACATCAGTTGGAGGTTC

GACGCCCCGGCCGGGTCGGATTACCTGCTCCGGTTCCACTGGTGCGACATCGTCAGCAAG

GCGGCCATCGGAATGGCCTTCAACGTCTACGTCGGCGGGTCGGTGGTGCTCGACAACTAC

GAGATCTCGCGTGACACGTTCAACCGGCTATCCATACCGGTGTACAAGGACTTCGTCCTG

GGCGCCAAGGACGCCAAGGGCGCCATCACCGTGAGCATCGGGTCGTCGACCGAGGACAAC

ACATTGCCTGACGGCTTCCTGAACGGCCTCGAGATCATGAGGGTAGTCGGGAGCGCCAGC

GCCGGCGCCGGCGCTCCCGCCGCGTCCCCGCCCAGTTCAAAGGTCAAAATCGGGATCATC

GCCGGCTCGGCCGTTTGCGGGGCCACGCTGGTAACGGTGCTCGGGTTCATCGCCTTCAGG

ATGCTGCGCGGGAGGGAGCCGGAGAAGAAGCAGCCGTCCGACACCTGGTCGCCGTTCTCG

GCGAGCGCGTTGGGCTCTCGCTCGCGCTCTCGGAGCTTTTCCAAGAGCAACGGGAACACC

GTCCTGCTCGGGCAGAACGGCGCCGGCGCCGGGTACAGGATCCCGTTCGCGGCGCTCCAG

GAGGCGACCGGCGGGTTCGACGAGGGGATGGTCATCGGCGAGGGCGGGTTCGGGAAGGTG

TACAAGGGCACGATGCGGGACGAGACGCTGGTGGCCGTGAAGCGCGGCAACCGGCGGACG

CAGCAGGGGCTGCACGAGTTCCACACGGAGATCGAGATGCTGTCCCGGCTGCGCCACCGG

CACCTGGTCTCGCTCATCGGCTACTGCGACGAGCGCGGCGAGATGATCCTCGTGTACGAG

TACATGGCCATGGGCACGCTGCGGAGCCACCTGTACGGCGCCGGCCTCCCGCCCCTGTCG

TGGGAGCAGAGGCTGGAGGCCTGCATCGGCGCCGCGCGGGGGCTGCACTATCTCCACACC

AGCTCCGCCAAGGCGATCATCCACCGGGACGTCAAGTCGGCCAACATCCTCCTCGACGAG

AGCTTCATGGCCAAGGTGGCCGACTTCGGGCTGTCCAAGAACGGGCCGGAGCTGGACGAG

ACGCACGTGAGCACCAAGGTGAAGGGCAGCTTCGGGTACCTGGACCCGGAGTACTTCCGG

CGGCAGATGCTGACGGAGAAGTCGGACGTCTACTCCTTCGGCGTGGTCCTGCTGGAGGTG

CTCTGCGCCCGCACCGTCATCGACCCCACGCTGCCGCGGGAGATGGTGAGCCTGGCAGAG

TGGGCGACGCCGTGTCTCAGAAACGGCCAGCTCGACCAGATCGTCGACCAGAGGATCGCC

GGGACGATACGGCCGGGGTCGCTCAAGAAGCTCGCGGACACGGCCGACAAGTGCCTCGCC

GAGTACGGGGTGGAGCGGCCCACCATGGGGGACGTGCTGTGGTGCCTCGAGTTCGCGCTG

CAGCTGCAGGTGGCGTCCTCAGACGTCTCGGACGCCGACACCATGTTGACGCCGCCCGTG

CCCGTGAAAACGCCCGAGGTTCAGCGTAGCCTGTCCGCCGCTACCGTGGCGACTGACGCT

GCCATGACCACCAACTTGGGTGATCTAGACGGAATGTCCCTGAGCGGAGTATTCTCCAAG

ATGATCAAGAGCGACGAGGTCAGGTGA

>Ta-*CrRLK1L8*-D CDS sequence

ATGCCGCCGGTTCTTGACATGCTCGTGCGGCTCCTCGTCGCGTCCGTGCTGCTCGGCGCA

GCCAGTGGCGCGTTTACCCCCGCGGACAACTACCTCGTCCTCTGCGGCACGTCGGCGAGC

GCCACCGTCGCCGCCGGACGGACGTTCGTCGGGGACGCGCGTCTGCCGGCCAAGTCGCTG

GCCGCGCCGCAGAGCGTCGAGGCCAACACATCGCGGACCGCGGTCGTCCCGTCCGGCGAG

TCGGAGCTGTATCGGTCCGCACGCGTGTTCACCGCGCCAGCTTCCTACACGTTCGCCGTC

AAGCAGCCCGGCCGGCACTTCGTGCGCCTTCACTTCTTCCCCTTCCCCTACAGGTCTTAC

GACATGGTGGCGGACGCCGCGTTCAACGTGTCCGTGCAGGGCGCGGTGCTCGTCAACGGG

TACACGCCCAAGAACGGCACAGCGGAGCTCAGGGAGTTCTCCGTGAACGTCACCGGGGGC

ACGCTGGTGATCGCGTTCGCGCCGACGGGGAAGCTCGCGTTCGTGAACGCCATCGAGGTC

GTGTCGGTCCCCGACGAGCTCATCGCCGACATGGCCAGGATGGTGGACGGCGCCGTCCAG

TACACCGGGCTGTCGACGCAGGCGCTGGAGACGATCCACAGGATCAACATGGGCGTTCCC

AAGATCACGCCCGGCAACGACACGCTGGGGAGGACGTGGTTGCCGGACCAGAGCTTCCAG

GTCAACACCGACCTAGCGCAGCACAAAGACGCCAAGCCCCTGACGATCAAATACGACGAG

AAATCGGCACTCTCGTCCGCGTACACGGCGCCGGCGGAGGTCTACGCGACGGCGACGAGG

CTGAGCACGGCGGGCGAGACCAGCACCATCAACGTGCAGTTCAACATCAGCTGGAGGTTC

GACGCCCCGGCCGGGTCGGATTACCTGCTCCGGTTCCACTGGTGCGACATCGTCAGCAAG

GCAGCCATGGGAATGGCCTTCAACGTCTACGTCGGCGGGGCGGTGGTGCTCGACAACTAC

GAGATTTCGCGTGACACGTTCAACCGGCTATCCATACCGGTGTACAAGGACTTCCTCCTG

GGCGCCAAGGACGCCAAGGGCGCCATCACCGTGAGCATCGGGTCGTCCACCGAGGACAAC

GCGTTGCCTGACGGCTTCCTCAACGGCCTCGAGATCATGAGTATAGTCGGGAGCGCCGGC

GCCGGCGCTGCCGCCACGTCCCCGCGCAGTTCAAAGGTCAAAATCGGGATCATCGCCGGC

TCGGCCGTCTGCGGGGCCACGCTGGTGATGGTGCTCGGGTTCATCGCCTTCAAGATGCTG

CGCGGGAGGGAGCCGGAGAAGAAGAAGCCGGCCGACGCCTGGTCGCCGTTCTCGGCGAGC

GCGCTGGGCTCTCGCTCGCGCTCCCGGAGCTTCTCCAAGAGCAACGGGAACACCGTCCTG

CTCGGGCAGAACGGCGCCGGCGCCGGGTACAGGATCCCGTTCGCGGCGCTCCAGGAGGCG

ACCGGCGGGTTCGACGAGGGGATGGTCATCGGCGAGGGCGGGTTCGGGAAGGTGTACAAG

GGCACGATGCGCGACGAGACGGTGGTGGCCGTGAAGCGCGGCAACCGGCGGACGCAGCAG

GGGCTGCACGAGTTCCACACGGAGATCGAGATGCTGTCCCGGCTGCGCCACCGGCACCTG

GTCTCGCTCATCGGCTACTGCGACGAGCGCGGCGAGATGATCCTCGTGTACGAGTACATG

GCCATGGGCACGCTGCGGAGCCACCTCTACGGCGCCGGCCTCCCGCCCCTGTCGTGGGAG

CAGAGGCTGGAGGCCTGCATCGGCGCCGCGCGGGGGCTGCACTACCTCCACACCGGCTCC

GCCAAGGCCATCATCCACCGGGACGTCAAGTCGGCCAACATCCTCCTCGACGAGAGCTTC

ATGGCCAAGGTGGCCGACTTCGGGCTGTCCAAGAACGGGCCGGAGCTGGACAAGACGCAC

GTGAGCACCAAGGTGAAGGGCAGCTTCGGGTACCTGGACCCGGAGTACTTCCGGCGGCAG

ATGCTGACGGAGAAGTCGGACGTTTACTCCTTCGGCGTGGTCCTGCTGGAGGCGCTCTGC

GCCCGCACCGTCATCGACCCGACGCTGCCGCGGGAGATGGTGAGCCTGGCGGAGTGGGCG

ACGCCGTGTCTCAGAAACGGCCAGCTCGACCAGATCGTCGACCAGAGGATCGCCGGGACG

ATACGGCCGGGGTCGCTCAAGAAGCTCGCGGACACGGCCGAGAAGTGCCTCGCCGAGTAC

GGGGTGGAGCGGCCCACCATGGGGGACGTGCTCTGGTGCCTCGAGTTCGCGCTGCAGCTG

CAGGTGGGGTCGTCAGACAGCTCGGACGTCGACACCATGTTGCCGCCGGCGCCGCCCGTG

CCCGTGAAAACGCCTGAGGTTCAGCGTCGCCTGTCCGCCGCTACCGTGGCGACTGACGCT

GCTGCCATGACCACCAACTTGGGTGACCTAGACGGAATGTCCCTGAGCGGAGTATTCTCG

AACATGATCAAGAGCGACGAGGTCAGGTGA

>Ta-*CrRLK1L9*-A CDS sequence

ATGGGAGGAGGCCCGAGATGCGCGCTCCTGCTGCTCGTGGCCGCCGCGGCCGCCGCGCTT

GTCCCCGCGGCGTGGGCGCAGGACCCGACCGCGCCGGCGCCCTCGGGGGCCCCCTTCGTG

CCGCGGGACGACATCCTGCTCGACTGCGGCGCCACGGGGAAGGGCAACGACACGGACGGC

CGGGAGTGGCGTGGCGACGCCGGCTCCAAGTACGCGCCGCCGAACCTCGCCTCCGCCGAC

GCGGGGGCGCAGGACCCCTCGGTGCCGCAGGTGCCCTACCTCACCGCGCGGGTCTCCGCG

GCGCCCTTCACCTACTCCTTCCCGCTCGGCCCCGGCCGCAAGTTCCTCCGCCTGCACTTC

TACCCGGCCAACTACTCCGGCCGCGCCGCCGCCGACGCCTTCTTCTCCGTCTCCGTCCCG

GCAGCCAAGGTCACGCTCCTCTCCAACTTCAGCGCCTACCAGACCGCCACGGCGTTCAAC

TTCGCCTACCTCGTGCGCGAGTTCTCCGTCAACGTCACCGGCCCGACCCTCGACCTCACC

TTCACCCCGGAGAAGGGGCGCCCCAACGCCTACGCCTTCATCAACGGCATCGAGGTCGTC

TCCTCCCCGGATCTCTTCGACCTCGCCACACCGTTCTTCGTCACCGGTGACGGCAACAAC

CAGCCGTTCCCGATGGACCCCGGTGCTGCTCTGCAGACCATGTACCGGCTCAACGTCGGA

GGCCAGGCCATCTCCCCTTCCAAGGACTCCGGCGGGGCTCGGTCATGGGACGACGACACG

CCCTACATTTACGGTGCAGGGGCTGGGGTGTCGTACCCGAACGATCCCAATGTCACAATC

ACCTACCCTCCCAGTGTGCCGGGATATGTGGCGCCACTGGATGTCTATGCCACGGCGCGA

TCAATGGGGATAGACAAGGGTGTGAACTTGGCCTACAATCTCACCTGGATAGTGCAGGTG

GATGCTGGGTTCACATACCTTGTGAGGCTCCATTTCTGCGAGATACAATCCCCAATTGAT

AAGCCGAATCAGCGGGTGTTCAACATTTACCTCAACAACCAGACTGCCGTGGAAGGTGCT

GATGTGCTCCAGTGGGTGGATCCGCGTAGTACCGGTACCCCATTGTACAAGGATTTCGTG

GTCGGCACTGTGGGTTCAGGGATTATGGATTTCTGGGTGGCTCTTCATCCGGATATACGG

AACAAGCCACAGTACTATGATGCTATTCTCAATGGGATGGAGGTGTTCAAGCTGCAACTT

ACTAATGGGAGCCTCGCGGGGCCAAACCCTGTCCCAAGTGCTGATCCAGCGGCGCATACC

GGGCAAGGGAAGAAGAGTTCGCTTGTCGGGCCTATTGCTGGTGGAGTAATTGGAGGTTTG

GCAGTGCTTGCACTTGGATATTGCTGCTTCATTTGCAAGAGGCGGAGGAAAGTGGCCAAG

GATGCCGGCATGAGTGATGGCCATTCTGGTTGGCTGCCGTTGTCGCTGTATGGCAATTCA

CACACTTCAAGCTCAGCCAAGTCGCATGCTACAGGGAGTATTGCTTCATCTTTGCCATCC

AACCTGTGCCGCCATTTCTCCTTTGCAGAGATCAAGGCTGCAACGAAGAACTTTGACGAG

TCACGGATCCTTGGTGTTGGTGGGTTCGGTAAAGTTTACCAGGGAGAGATCGATGGGGGC

ACAACTAAGGTGGCTATCAAGCGTGGCAACCCCTTGTCTGAGCAGGGCATACATGAGTTC

CAGACTGAAATTGAAATGCTGTCAAAGCTCCGCCACCGCCATCTTGTGTCGTTGATTGGT

TACTGCGAGGACAAGAATGAGATGATCCTGGTCTATGACCACATGGCCCATGGAACTCTG

CGTGAGCACCTATACAAGACCCAGAATGCGCCGCTTAGCTGGAGGCAGCGTTTGGAGATC

TGCATTGGTGCAGCCCGTGGGCTGCACTACCTTCACACCGGTGCAAAGCACACCATTATC

CACCGTGATGTGAAGACCACAAACATCCTCCTGGATGAGAAATGGGTCGCCAAGGTTTCA

GATTTTGGTCTGTCCAAGACTGGGCCGTCGATGGATCACACACATGTGAGCACAGTTGTC

AAGGGCAGTTTCGGTTACCTAGATCCTGAATACTTCCGCAGGCAGCAGCTCACCGAGAAG

TCTGATGTGTATTCATTTGGCGTGGTGCTGTTCGAGGTCCTCTGTGCTCGGCCTGCCTTG

AACCCCACTCTTGCAAAGGAAGAAGTCAGCCTGGCAGAATGGGCATTGCACTGCCAGAAG

AAGGGAATTCTGGATCAGATTGTTGATCCCTACCTAAAGGGAAAGATCGTTCCGCAGTGC

TTCAAGAAGTTTGCCGAGACAGCTGAGAAGTGCGTTGCCGACAACGGCATCGAGCGCCCT

TCGATGGGAGATGTGCTTTGGAACCTGGAGTTTGCTCTTCAGATGCAGGAAAGCGCGGAG

GAGAGCGGAAGCTTCGGCTGTGGGATGTCGGATGAGGAGGGCGCTCCCCTGGTGATGGCT

GGAAAGAAGGATCCCAATGACCCGTCAATCGATTCAAGCACCACCACGACCACGACAACT

TCCCTAAGCATGGGCGACCAGAGCGTCGCGAGCATCGACTCGGACGGGCTGACGCCGAGC

GCGGTCTTCTCGCAGATCATGAACCCCAAGGGGCGGTGA

>Ta-*CrRLK1L9*-B CDS sequence

ATGGGAGGAGGCCCGAGATGCGCGCTCCTGCTGCTCGCCGCGGCCGCCGCTTGCGCGGCG

CTTGTCCCCGCGGCGTGGGCGCAGGGCTCGACCGCGCCCGCGCCCTCGGGGGCCCCCTTC

GTGCCGCGGGACGACATCCTGCTCGACTGCGGCGCCACGGGGAAGGGCAACGACACGGAC

GGCCGGGAGTGGCGCGGCGACGCCAGCTCCAAGTACGCGCCGCCGAACCTCGCCTCGGCC

GACGCGGGGGCGCAGGACCCCTCGGTGCCGCAGGTGCCCTACCTCACTGCGCGGGTCTCC

GCGGCGGCCTTCACCTACTCCTTCCCGCTCGGCCCCGGCCGCAAGTTCCTCCGGCTGCAC

TTCTACCCGGCCAACTACTCCAACCGCGACGCGGCCGACGCCTTCTTCTCCGTCTCCGTC

CCGGCCGCCAAGGTCACGCTCCTCTCCAACTTCAGCGCCTACCAGACCGCCACGGCCCTC

AACTTCGCCTACCTCGTACGGGAGTTCTCCGTCAACGTCACTGGCCCGACCCTCGACCTC

ACCTTCACCCCGGAGAAGGGACGCCCCAACGCCTACGCCTTCATCAACGGCATCGAGGTC

GTCTCCTCTCCGGATCTCTTTGACCTCGCCACACCGTTCTTCGTCACCGGTGACGGCAAC

AACCAGCCATTCCCGATGGATCCCGGTGCTGCTCTGCAGACCATGTATCGGCTCAACGTC

GGAGGCCAGGCGATCTCCCCTTCCAAGGACTCCGGCGGGGCTCGGTCATGGGACGACGAC

ACGCCTTACATTTATGGTGCAGGGGCTGGGGTAACGTACCCGAACGATCCCAATGTCACA

ATCACCTATCCTGACAGTGTGCCGGGATATATGGCGCCTTCGGATGTCTATGCCACGGCC

CGATCAATGGGGATAGACAAGAATGTGAACTTGGCCTACAATCTCACCTGGATAGTGCAG

GTGGATGCTGGGTTCACATACCTTGTGAGGCTCCATTTCTGTGAGATACAATCCCCAATT

GATAAGCCAAATCAGCGGGTGTTCAACATTTACCTCAACAACCAGACTGCCGTGGAAGGT

GCTGATGTGATCCAGTGGGTGGATCCGCTTAGTACTGGCACCCCATTGTATAAGGATTAT

GTGGTCGGCACTGTGGGTTCAGGGATTATGGATTTCTGGGTGGCTCTTCATCCGGATATA

CGGAACAAGCCACAGTACTATGATGCTATTCTCAATGGGATGGAGGTGTTCAAGCTGCAA

CTTAGTAATGGGAGCCTCGCGGGGCCAAACCCTGTCCCAAGTGCTGATCCACAGGCGCAT

ACCGGGCAAGGGAAGAAGAAATCACTAGTCGGGCCTATTGCTGGTGGAGTAATTGGAGGT

TTGGCAGTGCTTGCACTTGGATATTGCTGCTTCATTTGCAAGAGGCGGAGGAAAGCGGCC

AAGGATACTGGCATGAGTGATGGCCATTCTGGTTGGCTGCCGTTGTCGCTGTATGGCAAT

TCACACACTTCAAGCTCAGCCAAGTCGCATGCTACAGGGAGTATTGCTTCATCTTTGCCA

TCGAACCTGTGTCGCCATTTCAGCTTTGCAGAGATCAAGGCTGCAACGAAAAACTTTGAC

GAGTCACGGATCCTTGGTGTTGGTGGGTTCGGTAAAGTTTACCAGGGCGAGATCGATGGG

GGCACAACTAAGGTGGCTATCAAGCGTGGCAATCCCTTGTCTGAGCAGGGTATACATGAG

TTCCAGACTGAAATTGAAATGCTGTCAAAGCTCCGCCACCGCCATCTTGTGTCGCTGATT

GGTTACTGCGAGGACAAGAATGAGATGATCCTGGTCTATGACCACATGGCTCATGGAACT

CTGCGTGAGCACCTATACAAGACCCAGAATGCACCGCTTAGCTGGAGGCAGCGTTTGGAG

ATCTGCATTGGTGCAGCTCGTGGGCTGCACTACCTTCACACCGGTGCAAAGCACACCATT

ATCCACCGTGATGTGAAGACAACAAACATCCTACTGGATGAGAAATGGGTCGCCAAGGTT

TCAGATTTTGGTCTGTCCAAGACTGGGCCATCGATGGATCATACACATGTGAGCACAGTT

GTCAAGGGCAGTTTTGGTTACCTAGATCCTGAATACTTCCGCAGGCAGCAGCTCACCGAG

AAATCTGATGTCTATTCGTTTGGTGTGGTGCTGTTCGAGGTCCTCTGTGCTCGGCCTGCC

TTGAACCCGACTCTTGCAAAGGAAGAAGTTAGCCTGGCAGAATGGGCATTGCACTGCCAG

AAGAAGGGAATTCTGGATCAGATTGTTGATCCCTACCTAAAGGGAAAGATTGTTCCGCAG

TGCTTCAAGAAGTTTGCCGAGACAGCTGAGAAGTGCGTTGCCGACAATGGCATCGAGCGC

CCTTCGATGGGAGATGTGCTTTGGAATTTGGAGTTTGCTCTTCAGATGCAGGAAAGTGCG

GAGGAGAGCGGAAGCTTTGGCTGTGGGATGTCGGATGAGGGCACTCCCCTGGTGATGGCT

GGAAAGAAGGATCCCAATGACCCATCGATCGATTCAAGCACCACCACGACCACGACAACT

TCCCTAAGCATGGGCGACCAAAGTGTCGCGAGCATCGACTCGGACGGGCTGACGCCGAGC

GCCGTGTTCTCACAGATCATGAACCCCAAGGGGCGGTGA

>Ta-*CrRLK1L9*-D CDS sequence

ATGGGAGGAGGCCCGAGATGCGCGCTCCTGCTGCTCGCCGCGGCCGCCGCCTGCGCGGCG

CTTGTCCCGGCGGCGTGGGCGCAGGCCCCGACAGCGCCGGCGCCCTCGGGGGCTCCCTTC

GTGCCGCGGGACGACATCCTGCTCGACTGCGGCGCCACGGGGAAGGGGAACGACACAGAC

GGCCGGGAGTGGCGCGGCGACGCCGGCTCCAAGTACGCGCCGCCGAACCTCGCCTCGGCC

GACGCGGGGGCGCAGGACCCCTCGGTGCCTCAGGTGCCCTACCTCACCGCGCGGGTCTCC

GCGGCGGCCTTCACCTACTCCTTCCCGCTCGGCCCCGGCCGCAAGTTCCTCCGGCTGCAC

TTCTACCCGGCCAACTACTCCAACCGCGACGCCGCCGACGCCTTCTTCTCCGTCTCCGTC

CCGGCCGCCAAGGTCACGCTCCTCTCCAACTTCAGCGCCTACCAGACCGCCACGGCGCTC

AACTTCGCCTACCTCGTGCGCGAGTTCTCCGTCAACGTCACCGGCCCGACCCTCGACCTC

ACCTTCACCCCGGAGAAGGGACGCCCCAACGCCTACGCCTTCATCAATGGCATCGAGGTC

GTCTCCTCCCCAGATCTCTTTGACCTCGCCACACCGCTCTTCGTCACCGGTGACGGCAAC

AACCAGCCATTCCCGATGGATCCTGGTGCTGCTCTGCAGACCATGTATCGGCTCAACGTC

GGAGGCCAGGCGATCTCCCCTTCCAAGGACTCCGGCGGGGCTCGGTCATGGGACGACGAC

ACGCCTTACATTTATGGTGCAGGGGCTGGGGTAACGTACCCGAACGATCCCAATGTCACA

ATCACCTACCCTGACAATGTGCCGGGATATGTGGCGCCTTCGGATGTCTATGCCACGGCG

CGATCAATGGGGATAGACAAGAATGTGAACTTGGCCTACAATCTCACCTGGATAGTGCAG

GTGGATGCTGGGTTCACATACCTTGTGAGGCTCCATTTCTGTGAGATACAATCCCCAATT

ACTAAGCCGAATCAGCGGGTGTTCAACATTTACCTCAACAACCAGACTGCCGTGGAAGGT

GCTGATGTGATCCAGTGGGTGGATCCGCTTAGTACTGGCACCCCATTGTATAAGGATTAT

GTGGTCAGCACTGTGGGTTCAGGGATTATGGATTTCTGGGTGGCTCTACATCCGAATACA

GGGAGCAAGCCACAGTACTATGATGCTATTCTCAATGGGATGGAGGTGTTCAAGCTGCAG

CTTAGTAATGGGAGCCTTGCGGGGCCAAACCCTGTCCCCAGTGCTGATCCACCGGCGCAT

ACCGGGCAAGAGAAGAAGAATTCACTAGTCGGGCCTATTGCCGGTGGAGTAATTGGAGGT

TTGGTAGTGCTTGCACTTGGATATTGCTGCTTCATTTGCAAGAGGCGGAGGAAAGTGGCG

AAGGATGCCGGCATGAGTGATGGCCATTCTGGTTGGCTGCCGTTGTCGCTGTATGGCAAT

TCACACACTTCAAGCTCAGCCAAGTCGCATGCTACAGGGAGTATTGCTTCATCTTTGCCA

TCCAACCTGTGTCGCCATTTCTCGTTTGCAGAGATCAAGGCTGCAACGAAAAACTTTGAC

GAGTCACGGATCCTTGGTGTTGGTGGGTTCGGCAAAGTTTACCAGGGAGAGATCGATGGG

GGCACAACTAAGGTGGCTATCAAGCGTGGCAATCCCTTGTCTGAGCAGGGTATACATGAG

TTCCAGACTGAAATTGAGATGCTGTCAAAGCTCCGCCACCGCCATCTTGTGTCGCTGATT

GGTTACTGCGAGGACAAGAATGAGATGATCCTGGTCTATGACCACATGGCCCATGGAACT

CTGCGTGAGCACCTATACAAGACCCAGAATGCACCGCTTAGCTGGAGGCAGCGCTTGGAG

ATCTGCATTGGTGCAGCTCGTGGGCTGCACTACCTTCACACCGGTGCAAAGCACACCATC

ATCCACCGTGATGTGAAGACGACAAACATACTCCTGGATGAGAAATGGGTCGCCAAGGTT

TCAGATTTTGGTCTGTCCAAGACTGGGCCGTCGATGGATCACACACATGTGAGCACAGTT

GTCAAGGGCAGTTTTGGATACCTAGATCCTGAATACTTCCGCAGGCAGCAGCTCACCGAG

AAATCTGATGTCTATTCATTTGGTGTGGTGCTGTTCGAGGTCCTCTGTGCTCGGCCTGCC

TTGAACCCCACTCTTGCAAAGGAAGAAGTCAGCCTGGCAGAATGGGCATTGCACTGCCAG

AAGAAGGGAATTCTGGATCAGATTGTTGATCCCTACCTAAAGGGAAAGATTGTTCCTCAG

TGCTTCAAGAAGTTTGCCGAGACAGCTGAGAAGTGTGTTGCCGACAATGGCATCGAGCGC

CCTTCGATGGGAGATGTGCTTTGGAACCTGGAGTTTGCTCTTCAGATGCAGGAAAGCGCG

GAGGAGAGCGGAAGCTTCGGCTGTGGGATCTCGGATGAGGAGGGCACTCCCCTCGTGATG

GCTGGAAAGAAGGATCCCAATGACCCGTCGATCGATTCAAGCACCACCACGACCACGACA

ACTTCCCTAAGCATGGGCGACCAAAGCGTCGCGAGCATCGACTCGGACGGGCTGACGCCG

AGCGCCGTGTTCTCGCAGATCATGAACCCCAAGGGGCGGTGA

>Ta-*CrRLK1L10*-A CDS sequence

ATGCTCCGGACGAGGATACTCGTGCTGGCTGCTGTGAGCATCGTGTTCGCCAACCTGCAG

TTCTTGAAGGCTCACGGGAGGGAGCTGTTTCTGAGCTGCGGCTCCAACGCCACCGCCGAT

GCCGATGGCCGGAGATGGATCGGCGACATGGCCCCTGACCTGAATTTCACTCTGAGCAGC

CCGGGGATCGCTGCTCTGCTGGCCGGGAGCAGCAATGGCAGTGAAATCATGGCGCCGGTG

TACCGCTCCGCGCGCTTCTTCACCACGACATCTTGGTATGACTTCAGCCTGCTGCCGGGG

AACTACTGCGTCAGGCTGCATTTCTTCCCGTCCGCATTCAGGAATTTCAGTGCAAACGGT

TCAGTGTTTGATGTCGTTGCCAATGACTTCAAGCTGGTGTCCAAGTTTAACGTGTCGGAG

GAGATAGTTTGGAGAAACTCAGTGAGCAATTCGGCCGCCACTGCGGTTGTCAAGGAGTAC

TTTCTTGCAGTCAATGGTTCTCGCCTGCAGATCGAGTTTGATCCAAGGCCCGGTTCATTT

GCATTTGTGAATGCGATCGAGGTGATGCTCACTCCAGATAATTCCTTCAACGGCATGGTG

AACAAAGTTGGTGGTGTGGATGTGCACATTCCTCCTGAATTAAGCGGCCGAGCTGTTGAG

ACTATGTATCGACTGAATATTGGAGGGCCTGCACTTGCATCTTCACATGATCAGTATCTT

CATAGACCATGGTACACTGATGAAGCATTCATGTTTTCTGCCAATGCTGCTTTGATTGTG

TCCAATACTTCAGCCATAAAGTATGTCTCAAGCAATGACTCCTCAATTGCTCCCCTTGAT

GTCTATGAGACCGCGAGAATCATGGGCAACAACATGGTCATGGACAAGAGGTTTAATGTG

ACATGGCGGTTCTTTGTCCACCCCAATTTTGATTACTTGGTCCGCCTTCATTTTTGCGAG

CTTGTCTATGACAAGCCCAGCCAGAGGATCTTCAAGATCTACATCAACAACAAGACAGCT

GCTGAGAACTACGATGTGTACAACAAAGCCGGAGGTATTAACAAGGCATATCATGAGGAC

TACTTTGATAGTTTGCCGCAGCAGGTAGACTCGCTCTGGCTTCAGCTAGGCCCGGACTCC

ATGACCAGTGCTTCAGGTACGGATGCACTTCTCAATGGTTTGGAGATATTCAAGCTCAGC

AGGAGTGGCAACCTTGATTATGTGCTTGGTCATATTGATATGGGCAACAAAAGGGGGCGT

TCCAAGGGTCGGAGCAGGATAGGTTTATGGGAAGAAGTTGGTATTGGCTCGGCCGCTTTT

GTGGCCCTGGTAAGTGTTGCTCTATTCTCATGGTGCTATGTAAGGAGGAAACGAAAAGCT

GTTAACGAGGAGGTCCCTGCTGGTTGGCACCCTCTGGTCCTCCATGAGGCTATGAAAAGC

ACTACAGATGCCCGCGCATCCAAAAAAGCACCCTTGGCACGCAATTCATCTTCCATTGGT

CATAGGATGGGCAGGCGATTCAGCATTGCAGATATTAGAGCTGCCACAAAAAACTTTGAC

GAGTCATTGGTCATTGGTTCTGGAGGTTTTGGCAAGGTTTACAAGGGTGAGGTCGATGAT

GGCATTACAGTCGCAATCAAGCGTGCAAATCCATTATGTGGTCAGGGCCTGAAAGAATTT

GAAACAGAGATCGAGATGCTCTCCAAGCTTAGGCACCGGCACCTTGTTGCAATGATTGGC

TATTGTGAAGAGCAGAAGGAGATGATTCTGGTCTATGAATACATGGCCAAGGGGACATTG

CGAAGCCATCTCTATGGAAGTGGCCTACCACCTTTGACATGGAAGCAACGGATTGATGCC

TGCATTGGTGCGGCCAGGGGCCTTCACTACCTCCACACGGGAGCAGATCGCGGTATAATT

CATAGGGATGTTAAGACTACTAACATCCTGTTGGACAAGAACTTTGTTGCAAAAATAGCA

GATTTTGGGTTGTCGAAAACTGGACCAACACTGGACCAGACCCATGTTAGTACAGCAATC

AGGGGTAGCTTCGGGTATCTTGATCCAGAGTACTTCCGGAGGCAGCAATTGACACAAAAA

TCTGATGTGTATTCTTTTGGTGTGGTTCTCTTTGAAGTTGCTTGTGCCAGGCCGGTTATA

GACCCTTCAGTGCCGAAGGATCAAATCAACTTGGCAGAATGGGCTATGAGATGGCAGCGT

CAGCGTTCGCTGGAAGCAATAGCGGATCCACGGCTGGATGGTGACTACTCGCCAGAATCC

TTGAAGAAGTTTGGTGATATCGCGGAGAAGTGTCTTGCTGATGATGGGAGAACCAGGCCA

TCAATGGGTGAGGTCTTGTGGCACCTGGAGTATGTGCTGCAGCTCCATGAAGCTTACAAA

CGCAACGTGGATTGCGAGTCATTTGGAAGCAGTGAACTGGGGTTCGCTGATATGTCTTTT

AGCATGCCTCACATCAGGGAGGGAGAAGAGGAGCATCACCCAAAGAAATCAGGTATCAGA

GAAGATTCAGCCCCTTGA

>Ta-*CrRLK1L10*-B CDS sequence

ATGCTCCGGATGAGGATACTCGTGCTGGCTTCTGTGAGCATCGTGTTCGCCAACCTGCAG

TTCTTGAAGGCTCATGGGAGGGAGCTGTTTCTGAGCTGCGGCTCCAACGCCACCGCCGAT

GCCGATGGCCGGAGATGGATCGGCGACATGGCCCCTGACCTGAATTTCACTCTGAGCAGC

CCGGGGATTGCTGCTCTCTTGGCCGGGAGCACCAATGGGAGTGAAATCATGGCACCGGTG

TACCGCTCGGCGCGCTTCTTTACCACGACATCTTGGTATGACATCAGCGTGCTGCCGGGG

AACTACTGTGTCAGGCTGCATTTCTTCCCGTCCGCATTCGGGAATTTCAGTGCAAATGGT

TCAGTGTTTGATGTCGTCGCCAATGAGTTCAAGCTGGTGTCGAAGTTTAACGTTTCGGAG

GAGATTGTTTGGAGAAATTCAGTGAGCAATTCAGCTGCCACTGCGGTTGTCAAGGAGTAC

TTTCTTGCAGTCAATAGTTCTCGCCTGCAGATCGAGTTTGATCCAAGGCCCGGTTCATTT

GCATTTGTTAATGCGATCGAGGTGATGCTCACTCCAGATAATTCCTTCAACAGCACAGTG

AACAAAGTTGGTGGTGTGGATGTGCACATTCCTCCTGAATTAAGCGGCCGAGCTATTGAG

ACCATGTATAGGCTCAACATTGGAGGGCCTGCACTTGCATCTTCACATGATCAGTATCTT

TATAGACCATGGTACACTGATGAAGCCTTCATGTTTTCTGCGAATGCTGCTTTGACCGTG

TCCAATACGTCAGCCATAAAGTATGTCTCAAGCGGCGACTCCTCAATTGCTCCCATCGGT

GTCTATGAGACTGCAAGAATCATGGGCAACAACATGGTCATGGACAAACGGTTCAATGTG

ACATGGCGGTTCGTTGTCCATCCCAATTTCGATTACATGGTCCGTCTTCATTTTTGCGAG

CTTGTCTATGACAAGCCCAGCCAGAGGATCTTCAAGATCTACATCAACAACAAGACAGCT

GCTGAGAACTACGATGTGTATGACAAGGCTGGAGGAATTAACAAGGCATATCATGAGGAC

TACTTTGACAGCTTGCCGCAACAGGTAGACTCACTCTGGCTTCAGCTAGGCCCGGACTCC

ATGACCAGTGCTTCAGGCACGGATGCACTTCTCAATGGTTTGGAGATATTCAAGATCAGC

AGGAGTGGCAACCTTGACTATGTGCTTGGTCATATTGATATGGGCAACAAAAGGGGCCGT

TCCAAGGGTCGGAGCAGGTTAGGTTTATGGGAAGAAGTTGGTATTGGCTCAGCCGCTTTT

GTGGCACTGGCAAGTGTTGCTCTATTCTCATGGTGCTATGTAAGGAGGAAACGGAAAGCT

GTCGACGAGGAGGTCCCTGCTGGTTGGCACCCTCTGGTCCTTCATGAGGCTATGAAAAGC

ACTACAGATGCCCGCGCATCCAAAAAAGCACCATTGGCACGCAATTCATCTTCCATTGGT

CATAGGATGGGCAGACGATTCAGCATTGTAGATATTAGGGCTGCCACAAAGAACTTTGAC

GAGTCATTGGTCATTGGTTCTGGAGGTTTTGGCAAGGTTTACAAGGGTGAGGTCGATGAT

GGCATTACAGTTGCAATCAAGCGTGCAAATCCATTATGTGGCCAGGGCCTGAAAGAATTT

GAAACAGAGATCGAGATGCTCTCCAAGCTTAGGCACCGGCACCTTGTTGCGATGATTGGC

TATTGTGAAGAGCAGAAGGAGATGATTCTGGTCTATGAATACATGGCCAAGGGGACATTG

CGAAGCCATCTCTATGGAAGTGGCCTACCACCTTTGACATGGAAGCAACGGATTGATGCC

TGCATTGGTGCGGCCAGGGGCCTTCACTACCTCCACACAGGAGCAGACCGAGGTATAATT

CATAGGGATGTTAAGACTACTAACATCCTGTTGGACAAGAACTTTGTTGCAAAAATAGCA

GATTTTGGGTTGTCGAAAACTGGACCAACACTGGACCAGACCCATGTTAGTACAGCAATC

AGGGGTAGCTTCGGGTATCTTGATCCAGAGTACTTCCGGAGGCAGCAATTGACACAAAAA

TCCGACGTGTATTCTTTTGGTGTGGTGCTCTTTGAAGTTGCTTGTGCCAGGCCGGTTATA

GACCCTTCAGTGCCGAAGGATCAAATCAACTTGGCAGAATGGGCTATGCGATGGCAGCGT

CAGCGTTCGCTGGAAGCAATAGCGGATCCACGGCTGGATGGTGACTACTCGCCAGAATCC

TTGAAGAAGTTTGGTGATATCGCAGAGAAGTGTCTTGCTGATGATGGGAGAACCAGGCCA

TCAATGGGTGAGGTTTTGTGGCACCTGGAGTATGTGTTGCAGCTCCATGAAGCTTACAAA

CGCAACGTGGATTGCGAGTCGTTTGGAAGCAGTGAACTGGGGTTCGCTGATATGTCTTTT

AGCATGCCTCACATCAGAGAAGGAGAAGAGGAGCATCACCCAAAGAAATCTGGTATCAGA

GAAGATTCAGCCCCTTAA

>Ta-*CrRLK1L10*-D CDS sequence

ATGCTCCGGATGAGGATACTCGTGCTGGCCGCTGTGAGCATCGTGTTCGCCAACCTGCAG

TTCTTGAAGGCTCACGGGAGGGAGCTGTTTCTGAGCTGCGGCTCCAACGCCACCGCCGAT

GCCGATGGCCGGAGATGGATCGGCGACATGGCCCCTGGCCTGAATTTCACTCTGAGCAGC

CCGGGAATCGCTGCTCTGCTGGCCGGGAGCAGCAATGGCAGTGAAATCATGGCGCCGGTG

TACCGCTCCGCGCGCTTCTTTACCACCACATCTTGGTATGACTTCAGCCTGCTGCCGGGG

AACTACTGCGTCAGGCTGCATTTCTTCCCGTCCACATTCAGGAATTTCAGTGCAAACGGT

TCAGTGTTTGATGTCGTCGCCAATGACTTCAAGCTGGTGTCCAAGTTTAACGTGTCGGAG

GAGATTGTTTGGAGAAACTCAGTGAGCAATTCAGCTGCCACTGCGGTTGTCAAGGAGTAC

TTTCTTGCAGTCAATAGTTCTCGCCTGCAGATCGAGTTTGATCCAAGGCCCGGTTCATTT

GCATTTGTGAATGCGATCGAGGTGATGCTCACTCCAGATAATTCCTTCAACGGCACGGTG

AACAAAGTTGGTGGTGTGGATGCACACATTCCTCCTGAATTAAGCGGCCGAGCTGTCGAG

ACCATGTATCGGCTGAACATTGGAGGGCCTGCACTTGCATCTTCACATGATCAGTATCTT

CATAGACCATGGTACACTGATGAAGCATTCATGTTTTCTGCCAATGTTGCTTTGATTGTG

TCCAATACTTCAGCCATAAAGTATGTCTCAAGCAACGACTCCTCAATTGCTCCCATCGAT

GTCTATGAGACCGCAAGAATCATGGGCAACAACATGGTCATGGACAAGCGGTTCAATGTG

ACATGGCGGTTCTTGGTCCACCCCAATTTTGATTACTTGGTCCGCCTTCATTTTTGTGAG

CTTGTCTATGACAAGCCCAGCCAGAGGATCTTCAAGATCTACATCAACAACAAGACAGCT

GCTGAGAACTATGATGTGTACAACAGGGCCGGAGGTATTAACAAGGCATATCATGAAGAC

TACTTTGATAGTTTGCCGCAGCAGGTAGACTCACTCTGGCTTCAGCTAGGCCCAGACTCC

ATGACCAGTGCTTCAGGTACCGATGCACTTCTCAATGGTTTGGAGATATTCAAGCTCAGC

AGGAGTGGCAACCTTGATTATGTGCTTGGTCATATTGATATGGGCAACAAAAGGGGGCGT

TCCAAGGGTCGGAGCAGGATAGGTTTATGGGAAGAAGTTGGTATTGGCTCGGCCGCTTTT

GTGGCACTGGCAAGTGTTGCTCTGTTCTCATGGTGCTATGTAAGGAGGAAACGAAAAGCT

GTTAACGAGGAGGTCCCTGCTGGTTGGCACCCTCTGGTCCTCCATGAGGCTATGAAAAGC

ACTACAGATGCCCGCGCATCCAAGAAAGCACCCTTGGCACGCAATTCATCTTCCATTGGT

CATAGGATGGGCAGACGATTCAGCATTGCAGATATTAGAGCTGCCACAAAAAACTTTGAC

GAGTCATTGGTCATTGGTTCTGGAGGTTTTGGCAAGGTTTACAAGGGTGAGGTCGATGAT

GGCATTACAGTCGCAATCAAGCGTGCAAATCCATTATGTGGTCAGGGACTGAAAGAATTT

GAAACAGAGATCGAGATGCTCTCCAAGCTTAGGCACCGGCACCTTGTTGCGATGATTGGC

TATTGTGAAGAGCAGAAGGAGATGATTCTGGTCTATGAATACATGGCCAAGGGGACATTG

CGAAGCCATCTCTATGGAAGTGGCCTACCACCTTTGACATGGAAGCAACGGATTGATGCC

TGCATTGGTGCGGCCAGGGGCCTTCACTACCTCCACACGGGAGCAGATCGGGGTATAATT

CATAGGGATGTTAAGACTACTAACATCCTGTTGGACAAGAACTTTGTTGCAAAAATAGCA

GATTTTGGGTTGTCGAAAACTGGACCAACACTGGACCAGACCCATGTTAGTACAGCAATC

AGGGGTAGCTTCGGGTATCTTGATCCAGAGTACTTCCGGAGGCAGCAATTGACACAAAAA

TCTGATGTGTATTCTTTTGGTGTGGTGCTCTTTGAAGTTGCTTGTGCCAGGCCGGTTATA

GACCCTTCAGTGCCGAAGGATCAAATCAACTTGGCAGAATGGGCTATGAGATGGCAGCGT

CAGCGTTCACTGGAAGCAATAGCGGATCCACGGCTGGATGGTGACTACTCGCCAGAATCC

TTGAAGAAGTTTGGTGATATCGCGGAGAAGTGTCTTGCTGATGATGGGAGAACCAGGCCA

TCAATGGGTGAGGTCTTGTGGCACCTGGAGTATGTGCTGCAGCTCCATGAAGCTTACAAA

CGCAATGTGGATTGCGAGTCATTTGGAAGCAGTGAACTGGGGTTTGCTGATATGTCTTTT

AGCATGCCTCACATCAGAGAGGGAGAAGAGGAGCATCACCCAAAGAAATCAGGTATCAGA

GAAGATTCAGCCCCTTGA

>Ta-*CrRLK1L11*-A CDS sequence

ATGGGCACCACTACCGAGCAGAAGATAGCTCTGCTCCTTCTTGGGACCATCTGGGTTCTT

CTTGGTACCTGCAATGCTGCTGAATTCACCCCTGCAGACAACTACCTCATCAACTGCGGC

TCTACGGTCGACGCTAATCTCAACGATGGGAGGGTCTTCAAAGCAGACAATTCCGGCTCG

ACTATATTGACATCACATCACAGCGTCCCCGCAAACACCTTGCCGGATGCAGTCATAAGT

TCTGACAATCCTGTGCTGTATAAAACTGCAAGGATATTCATTGTGCCGTCGTCCTACTCC

TTCAACATGAAGAGCCGTGGCCGGCATTTTGTCCGGCTACACTTCTTCGGTTTCAGATAC

CAGAGCTATGATCTTGCCGCGGCAAAGTTCAAAGTGTCCACCCAGCATGTTGTGTTACTT

GACAATTTCACTCCGCCGAGCAATTCCTCACTGTTGGTCAGGGAGTATTCGCTGAATATT

ACCGAGGATATGCTGATTCTCTCATTTGTGCCTCTTGGAAACAGCACATCTTTTATCAAT

GCTATTGAAGTAATATCTGTTCCTGATGATCTGATACAGGATTCAGCCCAAACTGTGAAT

CCCTCCGGCCAGTATCTTGGCCTTGCAACGCAGTCATTTCAGACATTCTACAGGATTAAT

GTAGGTGGACGGGAGGTGACCGTTGTCAATGACACACTCTCGCGTTCATGGGATACTGAC

CAGAACTTCTTCATAAATTCCACTACCACTGAACTGTTTGCTTACCAAGGGAAGCTGAAT

TATCAGAAAGGAGCTGCAACCAAGGAGGATGCACCGGACAGTGTGTACAACACTGCAAGG

CGGTTGGCTGTGCAAAATAGAACCAGCCCAGCGTCCAACATGACATGGCAATTTGATGTT

GACGGTCGTTCGAGCTATCTGATCCGGTTCCATTTTTGTGACATAGTGAGCAAGGCAGCA

TATTCCCTCTACTTTGATATTTATGTGGATGGCGGGTTAGCGTTAGAAAATCTTGACCTC

TCTGAAAAAGTTTTTGGTACCTTGGCTGTGCCATACTACACGGAATTTGTCTTGAAGTCA

AGCAATCCTTCTGGTAAGCTAAGTGTCGGCATTGGGCCTTCCAGCTTGAGCAATGTGGCA

CCAGATGGCATCTTGAACGGCCTGGAGATCATGAAGATGAACATCAGTACTGGCACTATT

TATGTTGTGTGGCCACCAGCAACGCCAAAAAGGAAACTGGCTATCATATTGGCCCCTGTT

CTCGGAGGTGTTGGTGCTGTTAGCATTGCTATTATTCTCTGCTTTGTCCTCAGAAGAAAG

AAGGAGAAGAAGCCGCGGCGGGCACCGACAAGTCGACCTTCAAGTTCTTGGTCACCACTT

ACCCTCAATGGCCTTAGCTTCCTTAGCATAGGTACCCGAACAACCAGCCGGACCACTCAT

ACATCTGGGACAAACAGTGATGTAAGCTACCGAATTCCTTTTGCTTTGCTGCAAGTGGCA

ACAAAACACTTCGACGAGCAGATGGTTGTCGGAGTTGGGGGGTTTGGGAAGGTATACAAA

GCAGTTCTGCAGGACAGCACCAAAGTCGCAGTCAAGCGGGGCAACCAGAAGTCCCACCAA

GGGCTCAAAGAATTCCGGACAGAGATCGAGCTGCTGTCGGGGCTGCGACACCGTCACCTC

GTGTCGCTCATCGGATATTGCGATGACCAGAACGAGATGATCTTGGTGTATGAGTACATG

GAGAAAGGCACGCTGAAGAGTCACCTGTATGGCAGTGACATGCCTCCCCTCAGCTGGAAG

AAAAGAGTGGAAATCTGCATAGGGGCTGCAAGGGGGCTCCACTACCTGCACACAGGTTTT

GCAAAGTCGATCATCCATCGTGATGTCAAGTCGGCAAACATTCTCCTCGACGAAAATCTC

ATGGCCAAGGTTTCTGATTTCGGTCTCTCGAAGACGGGACCTGAGTTGGATCAAACACAT

GTTAGCACTGCGGTGAAAGGGAGCTTCGGGTATCTTGACCCTGAGTACTACCGGAGGCAG

AAGCTGACCGACAAGTCGGATGTGTACTCATTCGGCGTGGTCTTGCTGGAGGTGATCTGT

GCGAGGCCAGTCATCGATCCGACGCTTCCAAGAGACATGATCAACCTTGCAGAATGGGCA

ATCAAGTGGCAGAAGAGGGGAGAGCTTGGTCAGATCGTCGATCAACGGATCGCTGGGACA

ATCAGGCCAGAGTCATTGAGGAAGTACGGTGAGACGGTCGAGAAGTGTCTTGTGGATTAC

GGCGTTGACCGCCCTACAATGGGTGATGTCCTGTGGAATCTGGAGTTTGTGCTCCAGCTG

CAGGAGGCCGGCCCGGACATCTCCAATGTCGACAGCATGAATCAGATCTCTGAACTTCCT

TCAGACGCCAGAAGGATGGGCTCTTTGGAGATCGGTACCGCAGACGAAGCAGACGAAGGC

CGCACGCACATGGATTACTCTCAGATGTCGACCAACGATGCCTTCTCGCAGCTGATGAAC

ACTGAAGGGAGGTGA

>Ta-*CrRLK1L11*-B CDS sequence

ATGGGCACCACTACCGAGCAAAAGATAGCTCTGCTCCTTCTTGGAACCATCTGGGTTCTT

CTTGGTACCTGCAATGCTGCTGAATTCACCCCTGCAGACAACTACCTCATCAACTGCGGC

TCCACGGTCGACGCTAATCTCCACGATGGGAGGGTCTTCAAAGCAGACAATTCCGGCTTG

ACTATATTGACATCACATCACAGCGTCCCCGCAAACACCTTGCCGGATGCAGTCATAAGT

TCTGACAATCCTGTGCTGTATCAAACTTCAAGGATATTCATTGTGCCGTCGTCCTACTCC

TTCAAGATGAAGAGCCGTGGCCGGCATTTTGTCCGGCTACACTTCTTCAGTTTCAGATAC

CAGAGCTATGATCTTGCCGCGGCAAAGTTCAAAGTGTCCACGCAGCATGTTGTGTTACTT

GACAATTTCACTCCGCCGAGCAATTCCTCACCATTGGTCAGGGAGTATTCGCTGAATATT

ACCGAGGATATGCTGATTCTCTCATTTGTGCCTCAGGGAAACAGCACATCTTTCATCAGT

GCTATTGAAGTAATATCTGTTCCTGATGATCTGATACAGGATTCAGCGCAAACTGTGAAT

CCCTCCGGCCAGTATCTCGGCCTTGCAACGCAGTCATTTCAGACATTCTACAGGATTAAT

GTGGGTGGACGGGAGGTGACCGTTGTCAATGACACACTCTCGCGTTCATGGGATACTGAC

CAGAACTTCTTCCTAAATTCCACTACCACTGAACTATTTGCTTACCAAGGGAAGCTGAAT

TATCAGAAAGGAGCTGCAACCAAGGAGGATGCACCGGACAGTGTGTACAACACTGCAAGG

CGGTTGGCTGTGCAAAATAGAACCAGCCCAGCGTCCAACATGACATGGCAATTTGATGTT

GACGGTCGTTCGAGCTATCTGATCCGGTTCCATTTTTGTGACATAGTGAGCAAGGCAGCA

TATTCCCTCTACTTTGATATTTATGTGGATGGCGGGTTAGCGTTAGAAAATCTTGACCTC

TCTGAAAAAGTTTTTGGTACCTTGGCTGTGCCATACTACACGGAATTTGTCTTGAAGTCA

AGCAATCCTTCTGGTAAGCTAAGTGTCGGCGTTGGGCCTTCCAGCTTGAACAATGTGGCG

CCAGATGGCATCTTGAACGGCCTGGAGATCATGAAGATGAACATCAGTACTGGCACTATT

TATGTTGTGTGGCCACCAGCACCGCCAAAAAGGAAACTGGCTATCATATTGGGCTCTGTT

CTCGGAGGTGTTGGTGCTGTTAGCATTGCTATTATTCTCTGCTTTGTCCTCAGAAGAAAG

AAGAAGGAGAAGAAGCCGCGGCGGGCACCGACAAGTCGACCTTCAAGTTCTTGGTCACCA

CTTACCCTCAATGGCCTTAGCTTCCTTACCGTAGGTACCCGAACAACCAGCCGGACCACT

CATACATCTGGGACAAACAGTGATGTAAGCTACCGAATTCCTTTTGCTTTGCTGCAAGTG

GCAACAAAACACTTTGACGAGCAGATGGTTGTCGGAGTTGGGGGGTTTGGGAAGGTATAC

AAAGCAGTTCTGCAGGACAGCACCAAAGTCGCAGTCAAGCGGGGCAACCAGAAGTCCCAC

CAAGGGCTCAAAGAATTCCGGACAGAGATCGAGCTGCTGTCGGGGCTGCGACACCGTCAC

CTCGTGTCGCTCATCGGATATTGCGATGAGCAGAACGAGATGATCTTGGTGTATGAGTAC

ATGGAGAAAGGCACGCTGAAGAGTCACCTGTATGGCAGTGACATGCCTCCCCTCAGCTGG

AAGAAAAGAGTGGAAATCTGCATAGGGGCTGCAAGGGGGCTCCACTACCTGCACACAGGT

TTTGCAAAGTCGATCATCCATCGTGATGTCAAGTCGGCAAACATTCTCCTCGACGAAAAT

CTCATGGCCAAGGTTTCTGATTTCGGTCTCTCGAAGACGGGACCTGAGTTGGATCAGACA

CATGTTAGCACTGCGGTGAAAGGGAGCTTCGGGTATCTTGACCCTGAGTACTACCGGAGG

CAGAAGCTGACCGACAAATCGGATGTGTACTCATTTGGCGTGGTCTTGCTGGAGGTGATC

TGCGCGAGGCCAGTCATCGACCCGACGCTTCCAAGAGACATGATCAACCTTGCAGAATGG

GCAATCAAGTGGCAGAAGAGGGGAGAGCTTGGTCAGATTGTCGATCAACGGATCGCTGGG

ACAATCAGGCCAGAGTCATTGAGGAAGTACGGTGAGACGGTCGAGAAGTGTCTTGCGAAT

TACGGCGTCGACCGCCCTACCATGGGTGATGTACTGTGGAATCTGGAGTTTGTGCTCCAG

CTGCAGGAGGCCGGCCCGGACATCTCCAATGTCGACAGCATGAATCAGATCTCTGAACTT

CCTTCAGACGCCAGAAGGATGGGCTCTCTGGAGATCCGCACCGCAGACGAAGCAGACGAA

AGCCGCACAAACATGGATTACTCTCAGATGTCGACCAACGATGCCTTCTCGCAGCTGATT

AACACTGAAGGGAGGTGA

>Ta-*CrRLK1L11*-D CDS sequence

ATGGGCACCACTACCGGGCAAAAGACAGCTCTGCTCCTTCTTGGGACCCTCTGGGTTCTT

CTTGGTACCTGCAATGCTGCTGAATTCTCCCCTGCAGACAACTACCTCATCAACTGCGGC

TCCACAGTCGACGCTAATCTCCACGATGGGAGGGTCTTCAAAGCAGACAATTCCGGCTCG

ACTATATTGACATCACATCACAGCGTCCCTGCAAACACCTTGCCGGATGCAGTCATAAGT

TCTGACAATCCTGTGCTGTATCAAACTGCAAGGATATTCATTGTGCCGTCGTCCTACTCC

TTCAACATGAAGAGCCGTGGCCGGCATTTTGTCCGGCTACACTTCTTCGGTTTCAGATAC

CAGAGCTATGATCTTGCCGCGGCAAAGTTCAAAGTGTCCACCCAGCATGTTGTGTTACTG

GACAATTTCACTCCGCCGAGCAATTCCTCACCGTTGGATTCAGCGCAAACTGTGAATCCC

TCCGGCCAGTATCTCGGCCTTGCAACACAGTCATTTCAGACATTCTACAGGATTAATGTG

GGTGGACGGGAGGTGACCGTTGTCAATGACACACTCTCGCGTTCATGGGATACTGACCAG

AACTTCTTCATAAATTCCACTACCACTGAACTGTTTGCTTACCAAGGGAGGCTGAATTAT

CAGAAAGGAGCTGCAACCAAGGAGGATGCACCGGACAGTGTGTACAACACTGCAAGGCGG

TTGGCTGTGCAAAATAGAACCAGCCCAGCGTCCAACATGACATGGCAATTTGATGTTGAC

GGTCGTTCGAGCTATCTGATCCGGTTCCATTTTTGTGACATAGTGAGCAAGGCAGCATAT

TCCCTCTACTTTGATATTTATGTGGATGGCGGGTTAGCGTTAGAAAATCTTGACCTCTCT

GAAAAAGTTTTTGGTACCTTGGCTGTGCCATACTACACGGAATTTGTCTTGAAGTCAAGC

AATCCTTCTGGTAAGCTAAGTGTCGGCATTGGGCCTTCCAGCTTGAACAATGTGGCACTA

GATGGCATCTTGAACGGCCTGGAGATCATGAAGATGAACATCAGTACTGGCACTATTTAT

GTTGTGTGGCCACCAGCAACGCCAAAAAGGAAACTGGCTATCATATTGGGCCCTGTTCTC

GGAGGTGTTGGTGCTGTTAGCATTGCTATTATTCTCTGCTTTGTCCTCAGAAGAAAGAAG

AAGGAGAAGAAGCCGCGGCGGGCACCGACAAGTCGACCTTCAAGTTCTTGGTCACCACTT

ACCCTCAATGGCCTTAGCTTCCTTAGCATAGGTACCCGAACAACCAGCCGGACCACTCAT

ACATCTGGGACAAACAGTGATGTAAGCTACCGAATACCTTTTGCTTTGCTGCAAGTGGCA

ACAAAACACTTCGACGAGCAGATGGTTGTCGGAGTTGGGGGGTTTGGGAAGGTATACAAA

GCAGTTCTGCAGGACAGCACCAAAGTCGCAGTCAAGCGGGGCAACCAGAAGTCCCACCAA

GGGCTCAAAGAATTCCGGACAGAGATCGAGTTGCTGTCGGGGCTGCGACACCGTCACCTC

GTGTCGCTCATCGGATATTGCGATGAGCAGAACGAGATGATCTTGGTGTATGAGTACATG

GAGAAAGGTACGCTGAAGAGTCACCTGTATGGCAGTGACATGCCTCCCCTCAGCTGGAAG

AAAAGAGTGGAAATCTGCATAGGGGCTGCAAGGGGGCTCCACTACCTGCACACAGGTTTT

GCAAAGTCGATCATCCATCGTGATGTCAAGTCGGCAAACATTCTCCTCGACGAAAATCTC

ATGGCCAAGGTTTCTGATTTCGGTCTCTCGAAGACGGGACCTGAGTTGGATCAGACACAT

GTTAGCACTGCGGTGAAAGGGAGCTTCGGGTATCTTGACCCTGAGTACTACCGGAGGCAG

AAGCTGACCGACAAGTCGGATGTGTACTCATTCGGCGTGGTCTTGCTGGAGGTGATCTGC

GCGAGGCCAGTCATCGACCCGACGCTTCCAAGAGACATGATCAACCTTGCAGAATGGGCA

ATCAAGTGGCAGAAGAGGGGAGAGCTTGGTCAGATCGTCGATCAACGGATCGCTGGGACA

ATCAGGCCAGAGTCATTGAGGAAGTACGGTGAGACGGTCGAGAAGTGTCTTGCGGATTAC

GGCGTCGACCGCCCTACCATGGGTGATGTCCTGTGGAATCTGGAGTTTGTGCTCCAGCTG

CAGGAGGCCGGCCCGGACATCTCCAATGTGGACAGCATGAATCAGATCTCTGAACTTCCT

TCAGACGCCAGAAGGATGGGCTCTCTGGAGATCGGTACCGCAGACGAAGGCCGCACGAAC

ATGGATTACTCTCAGATGTCGACCAACGATGCCTTCTCGCAGCTGATGAACACTGAAGGG

AGGTGA

>Ta-*CrRLK1L12*-A CDS sequence

ATGGCGGCCGCCCGAGGCCGAGGCCGAGGCCGAGGCCGATGCGTCCTTCTCGCTGCCGTC

CTCCTCTTGACGGCGGTGGTCGGCGCAGACATATACAAGCCAACGGACTCCATTCTGGTT

CACTGCGGGTCGGACAAGGACGGGCAGGACGAGGACGGCAGGAAGTGGACCACCGACAAG

GACAGCAAGTGGCTCCCCGACGGCGGCAAGTCCTCCATCATGGGCACCGCCGACGTGGCG

GACCCGTCGCTCCCCTCCCCCGTGCCCTACATGACGGCGCGGGTCTTCCCCAAGGAGACC

GCCTACACCTTCCCCGTGGCCGACGCCGACCGCCACTGGGTGCGCCTCCACTTCTACCCG

GCGGCCTACCACGGCATCCCCGCCGACCACTTCTTCTTCTCCGTCACCACCTCCACCGGC

GTCACGCTGCTGCGCAACTTCAGCGTCTACATCACCGCCAAGGCCCTCACCCAGGCCTAC

ATCATCCGGGAGTTCTCCCTCCCTCCCTCCACCGTCGGCTCGCTCTCCCTCAAATTCACG

CCCACCGCCATGAACAACGCCTCCTACGCCTTCGTCAACGGCATCGAGGTCATCTCCATG

CCCAGCTTCTTCGGCGACCCGGCCACACTGGTCGGCCTCAACGACCAGTCCCTCGACGCC

AGCGCCGCCAACCTGCAGACCATGTACCGGCTCAGCGTCGGCGGCTCCTACATCCCGCCC

GCCAACGACTCCGGCCTGTCCCGCGAGTGGTTCTCCGACACGCCCTACGTCTACGGCGCC

GCCACGGGCGTCACCTTCGAGGCCAACGACACGGTCCCGATCAAGTACCCGGCCCCCGCC

GACGAGTACGCCGCGCCCGTCAGCATCTACGACTCGTTCCGCCACATGGGGCGCGACCCC

AAGATGAACAAGAACAACAACCTCACCTGGGTGTTCGAGGTGGACGGCAACTTCACCTAC

CTCCTCCGCCTCCACTTCTGCTCGCTCATGGAAGACAAGATCAACCAGGTCGTCTTCGCC

ATCCTCCTCAACAACAAGACCGCCACCACCACCGGCAGCGCCGACATCATCGCCTGGGCC

AAGGAGAAGAACCCTGCTAATCCCGGCGCGCCCGGCAAGGGCGTGCCGGTCTTCAAGGAC

TACGCGGTGTTCATGCCCGCCGCCCCGGCGGGCAACGACACCATCCTCTGGCTCACGCTG

CGCCCGGACACCGCCACCAGAACACAGTTCGTCAACGCTTTCCTCAACGGCCTGGAGGTG

TTTAAGGTGAGCGACGCCTCCGGCAACCTGGCCGGCCCAAACCCGGACATCTCCAAGATG

CTGGCGGAGGCCGAGCTGGGGGCCGTGGAGGGGCAGTTCAGGGAGAAGCCGAGCAACGTC

GGGGCGCTCATCGGCGGGGCGGCGGGCGGCGCGGCGGCGTTCGGGCTGGTGGCGGCCGTG

TGCTTCGTGGCGTACCAGAGCAAGAGGAGGAGGGAGCTGAGCAGCAGCCCGTCACACTCC

TCCTCCGGGTGGCTGCCGGTGTACGGCGGGTCGACGAGCGTGAGCAAGTCGTCGGGCGGC

AGGAGCGCGGCGACGCTCAACCCCAACATCACGGCCATGTGCCGGCACTTCTCGCTCCAG

GAGATAAAGTCGGCGACCAAGGGGTTCGACGAGTCGCTGGTGATCGGCGTGGGCGGGTTC

GGCAAGGTGTACCGCGGGGTGGTGGACGGGGACACCAAGGTGGCCGTCAAGCGGAGCAAC

CCGTCGTCGGAGCAGGGGGTACTGGAGTTCCAGACGGAGATCGAGATGCTGTCCAAGCTG

CGGCACAAGCACCTGGTGTCCCTCATCGGCTGCTGCGAGGACAACGGCGAGATGATCCTG

GTGTACGACTACATGGCGCACGGCACGCTGCGGGAGCACCTGTACAACAAGAGCGGCAAG

CCGCCGCTGCCGTGGAGGCAGCGGCTGGAGATCGTCATCGGCGCCGCCCGGGGGCTGCAC

TACCTCCACACGGGCGCCAAGTACACCATCATCCACCGGGACGTCAAGACCACCAACATC

CTGGTGGACGACAAGTGGGTGGCCAAGGTGTCCGACTTCGGCCTCTCCAAGACGGGGCCG

ACGGTGCAGAACCAGACGCACGTGAGCACCATGGTGAAGGGCAGCTTCGGGTACCTGGAC

CCGGAGTACTTCCGGCGGCAGAAGCTGACGGAGAAGTCGGACGTCTACTCGTTCGGCGTG

GTGCTGTTCGAGGTGCTGTGCGGGCGGCCGGCGCTGAACCCGAGCCTGCCGCGGGAGCAG

GTGAGCCTGGCGGACCACGCGCTGAGCTGCCAGCGGAAGGGCACCCTGGAGGAGATCGTG

GACCCGGTGCTGGAGGGGAAGATCGCGCCCGACTGCCTCAAGAAGTTCGCCGAGACGGCG

GAGAAGTGCCTGGCGGACCAGGGCGTGGACCGGCCGTCCATGGGCGACGTGCTGTGGAAC

CTCGAGTTCGCGCTGCAGATGCAGGACACCTTCGACAACGGCGGCAAGCCGCCCGAGGTC

GACGACTACAGCAGCAGCTTCACCATCGCCCAGCCGTCCATGGAGGAGAGCCTGGCGGCC

AACGCCGCGGCGCTCTCGCTCATCAGCGAGGACATGGACGAGGAGGACATTGCCAACTCC

GTCATCTTCTCCCAGATCGCTAAACCAACCGGACGATGA

>Ta-*CrRLK1L12*-B CDS sequence

ATGGCGGCCGCCCGAGGCCGAGGCGTCCTTCTCGCCGTCCTCCTCTTGACGACGGTGGCC

TTCGCGTTCGTCGGCGCGGACATATACAAGCCAACGGACTCCATTCTGGTTAACTGCGGG

TCGGACAAGGACGGGCAGGACGAGGACGGCAGGAAGTGGACCACCGACAAGGACAGCAAG

TGGCTCCCCGACGGCGGCAAGTCCTCCATCATGGGCACCGCCGACGTGTCGGACCCGTCC

CTCCCCTCCCCCGTGCCCTACATGACGGCGCGGGTCTTCCCTAAGGAGACCGCCTACACC

TTCCCCGTGTCCGACGCCGACCGCCACTGGGTGCGCCTCCACTTCTACCCGGCGGCTTAC

CACGACATCCCCGCCGACCACTTCTTCTTCTCCATCAGCACCTCCACCGGCATCACGCTG

CTGCGCAACTTCAGCGTCTACATCACCGCCAAGGCCCTCACCCAGGCCTACATCGTCCGG

GAGTTCTCCCTCCCTCCCTCCACCGCCGGCTCGCTCTCCCTCAAATTCACGCCCACTGCC

ATGAACAATGCCTCCTACGCCTTCGTCAATGGCATCGAGATCATCTCCATGCCCAACTTC

TTCGGCGACCCGGCCACGCTGGTCGGCCTCGACGACCAGTCCCTCGACGCCAGCGCCGGC

AACCTGCAGACCATGTACCGGCTTAGCGTCGGCGGCTCCTACATCCCGCCCACCAACGAC

TCCGGGCTGACCCGCGAGTGGTTCTCCGACACGCCCTACGTCTACGGCGCCGGCACGGGC

GTCACCTTCGAGGCCAACGACACGATCCCGATCAAGTACCCGGCCCCCGCCGACGAGTAC

GCCGCGCCCGTCAGCATCTATGACACGTTCCGCCACATGGGCCGCGACGCCAACCTGAAC

AAGAACAACAACCTCACCTGGGTGTTCGAGGTGGACGGCAACTTCACCTACCTCCTCCGC

CTCCACTTCTGCTCGCTCATGGAAGACAAGATCAACCAGGTCGTCTTCGCCATCCTCGTC

AACAACAAGACGGCCACCACCACCGGCAGCGCCGACATCATCGCCTGGGCCAAGGAGAAG

AACCCTGCTAATCCCGGCGCGCCCGGCAAAGGCGTGCCGGTCTTCAAGGACTACGCCGTG

TTCATGCCCGCCGCTCCGGCGGGCAACGACACCATCCTCTGGCTCACGCTGCGCCCAGAC

ACCGCCAGTAACCCACAGTTCGTCAACGCTTTCCTCAACGGCCTCGAGATCTTTAAGGTG

AGCGACGCCTCCGGCAACCTGGCCGGCCCAAACCCCGACATTTCTAAGATGCTGGCGGAG

GCCGAGCTGGGGGCCGTGGACGGGCAGTTCAGGGAGAAGCCGAGCAACGTCGGGGCGCTC

ATCGGCGGGGCGGTGGGCGGCGCGGCGGCATTCGGGCTGGTCGCGGCCGTGTGCTTCGTG

GCGTACCAGAGCAAGAGGGGGAGGGAGCTGAGCAGCAGCCCATCGCACTCCTCCTCCAGG

TGGCTGCCGGTGTACGGCAGCTCGCAGACGAGCGTGAGCAAGTCGTCGGGCGGGAGGAGC

GCGATGACGCTGAACCCCAACATCACGGCCATGTGCCGGCACTTCTCGTTCCAGGAGATA

AAGTCGGCGACCAAGGGGTTCGACGAGTCGCTGGTGATCGGCGTGGGCGGGTTCGGCAAG

GTGTACCGGGGGGTGGTGGACGGGGACACCAAGGTGGCCATCAAGCGGAGCAACCCGTCG

TCGGAGCAGGGGGTGCTGGAGTTCCAGACGGAGATCGAGATGCTGTCCAAGCTGCGGCAC

AAGCACCTGGTGTCCCTCATCGGGTGCTGCGAGGACAACGGCGAGATGATCCTGGTGTAC

GACTACATGGCGCACGGCACGCTGCGGGAGCACCTGTACAAGAGCGGCAAGCCGCCGCTG

CCGTGGAGGCAGCGGCTGGAGATCGTGATCGGCGCCGCCCGGGGGCTCCACTACCTCCAC

ACGGGCGCCAAGTACACCATCATCCACCGCGACGTCAAGACCACCAACATCCTCGTCGAC

GAGAAGTGGGTGGCCAAGGTGTCCGACTTCGGGCTGTCCAAGACGGGGCCGACGGTGCAG

AACCAGACGCACGTGAGCACCATGGTGAAGGGCAGCTTCGGGTACCTGGACCCGGAGTAC

TTCCGGCGGCAGAAGCTGACGGAGAAGTCGGACGTCTACTCGTTCGGCGTGGTGCTGTTC

GAGGTGCTGTGCGGGCGGCCGGCGCTGAACCCTAGCCTGCCGCGGGAGCAGGTGAGCCTG

GCGGACCACGCGCTGAGCTGCCAGCGGAAGGGCACCCTGGAGGAGATCATCGACCCGGTG

CTGGAGGGGAAGATCGCGCCCGACTGCCTCAAGAAGTTCGCCGAGACGGCGGAGAAGTGC

CTGGCGGACCAGGGCGTGGACCGGCCGTCCATGGGCGACGTGCTGTGGAACCTCGAGTTC

GCGCTGCAGCAGCAGGACACCTTCGAGAACGGCGGGAAGCCGCCCGAGGTGGACGACTAC

AGCAGCAGCTTCACCATCACCCCGCCGTCCATGGAGGAGAGCCTGGCGGCCAACGCGGCG

GCGCTGTCGCTCATCAGCGAGGACATGGACGAGGAGGACATCGCCAACAGCGTCATCTTC

TCCCAGATCGCAAAACCCACCGGACGATGA

>Ta-*CrRLK1L12*-D CDS sequence

ATGGCGGCCGCCCGAGGCCGAGGCGTCCTTCTCGCCGTCCTCCTCTTGATGATGGTGGCG

TTTGCGTTCGTCGGCGCAGACATATACAAGCCAACGGACTCCATTCTGGTTCACTGCGGG

TCGGACAAGGACGGGCAAGACGAGGACGGCAGGAAGTGGACCGCCGACAAGGACAGCAAG

TGGCTCCCCGACGGGGGCAAGTCCTCCGTCATGGGCACCGCCGACGTGCCGGACCCGTCG

CTCCCCTCCCCTGTGCCCTACATGACGGCGCGGGTCTTCCCCAAGGAGACCGCCTACACC

TTCCCCGTGGCCGACGCCGACCGCCACTGGGTGCGCCTCCACTTCTACCCGGCGGCCTAC

CACGGCATCCCCGCCGACCACTTCTTCTTCTCCGTCACCACCTCCACCGGCGTCACGCTG

CTCCGCAACTTCAGCGTCTACACCACCGCCAAGGCCCTCACCCAGGCCTACATCGTCCGG

GAGTTCTCCCTCCCTCCCTCCACCACCGGCTCGCTCTCCCTCAAATTCACGCCCACCGCC

ATGAACAACGCCTCCTACGCCTTCGTCAACGGCATCGAGATCATCTCCATGCCCAGCTTC

TTCGGCGACCCGGCCACGCTGGTCGGTCTCGACGACCAGTCCCTCGACGCCAGCGCCGGC

AACCTGCAGACCATGTACCGGCTCAGCGTCGGCGGCTCCTACATCCCTCCCGCCAACGAC

TCCGGGCTGTCCCGTGAGTGGTTCTCCGACACACCCTACGTCTACGGCGCCGCCACGGGC

GTCACCTTCGAGGCCAACGACACGATCCCGATCAAGTACCCGACCCCCGCCGACGAGTAT

GCCGCGCCCGTCAGCATCTACGACTCGTTCCGCCACATGGGGCGCGACCCCAAGATGAAC

AGGAACAACAACCTCACCTGGGTGTTCGAGGTGGACGGCAACTTCACCTACCTCCTCCGC

CTCCACTTCTGCTCGCTCATGGAAGACAAGATCAACCAGGTCGTCTTCGCCATCCTCGTC

AACAACAAGACGGCCACCACCACCGGCAGCGCCGACATCATCGCCTGGGCCAAGGAGAAG

AACCCTGCTAATCCCGGCGCGCCCGGCAAAGGCGTGCCGGTCTTCAAGGACTACGCCGTG

TTCATGCCCGCCGCTCCGGCAGGCAGCGACACCATCCTCTGGCTCACGCTGCGCCCAGAC

ACCGCCACCAATCCACAGTTCGTCAACGCTTTCCTCAACGGCCTGGAGGTCTTTAAGGTG

AGCGACGCCTCCGGCAACCTGGCCGGCCCAAACCCCGACATCTCCAAGATGCTGGCGGAG

GCCGAGCTGGGGGCCGTGGACGGGCAGTTCAGGGAGAAGCCGAGCAACGTCGGGGCGCTC

ATCGGCGGGGCGGCGGGCGGCGCGGCGGCGTTCGGGCTGGTGGCGGCCGTGTGCTTCGTG

GCGTACCAGAGCAAGAGGAGGAGGGAGCTGAGCAGCAGCCCGTCGCACTCCTCCTCCGGG

TGGCTGCCGGTGTACGGCGGCAACTCGCAGACGAGCGTGAGCAAGTCGTCGGGCGGCAGG

AGCGCGGTGACGCTGAACCCCAACATCACGGCCATGTGCCGGCACTTCTCGTTCCAGGAG

ATAAAGTCGGCGACCAAGGGGTTCGACGAGTCGCTGGTGATCGGCGTGGGCGGGTTCGGG

AAGGTGTACCGGGGGGTGGTGGACGGGGACACCAAGGTGGCCATCAAGCGGAGCAACCCG

TCGTCGGAGCAGGGGGTGCTGGAATTCCAGACGGAGATCGAGATGCTGTCCAAGCTGCGG

CACAAGCACCTGGTGTCCCTCATCGGGTGCTGCGAGGACAACGGCGAGATGATCCTGGTG

TACGACTACATGGCGCACGGCACGCTGCGGGAGCACCTGTACAAGAGCGGCAAGCCGCCG

CTGCCGTGGAGGCAGCGGCTGGAGATCGTGATCGGCGCCGCCCGGGGGCTCCACTACCTC

CACACGGGCGCCAAGTACACCATCATCCACCGCGACGTCAAGACCACCAACATCCTGGTG

GACGAGAAGTGGGTGGCCAAGGTGTCCGACTTCGGGCTGTCCAAGACGGGGCCGACGGTG

CAGAACCAGACGCACGTGAGCACCATGGTGAAGGGCAGCTTCGGGTACCTGGACCCGGAG

TACTTCCGGCGGCAGAAGCTGACGGAGAAGTCGGACGTCTACTCGTTCGGCGTGGTGCTG

TTCGAGGTGCTGTGCGGGCGGCCGGCGCTGAACCCGAGCCTGCCGCGGGAGCAGGTGAGC

CTGGCGGACCACGCGCTGAGCTGCCAGCGGAAGGGCACCCTGGAGGAGATCATCGACCCG

GTGCTGGAGGGGAAGATCGCGCCCGACTGCCTCAAGAAGTTCGCCGAGACGGCGGAGAAG

TGCCTGGCGGACCAGGGCGTGGACCGGCCGTCCATGGGCGACGTGCTGTGGAACCTCGAG

TTCGCGCTGCAGCAGCAGGACACCTTCGAGAACGGCGGGAAGCCGCCCGAGGTGGACGAC

TACAGCAGCAGCTTCACCATCACCCCGCCGTCCATGGAGGAGAGCCTGGCGGCCAACGCG

GCGGCGCTCTCGCTCATCAGCGAGGACATGGACGAGGAGGACATCGCCAACAGCGTCATC

TTCTCCCAGATCGCAAAACCCACCGGACGATGA

>Ta-*CrRLK1L13*-A CDS sequence

ATGGTGCGCCGCGGGGCGCTCCCGCTGGCGCTGCTGGCCGTGCTCGCGACGCTGACGGCC

GTGGCGGGGCAGGGGAAGCCGGTCACGGACAACGGCTCGGGCGGCGGGTCGGGGCCGTCC

AAGTTCACGCCCAAGGACGCCTTCTACATCGACTGCGGCGGCACGGCCGCCGCCGACACC

AAGGACGGCAAGTCCTTCAAGACCGACGCGGAGGCCAACAGCCTGCTCTCCGCCAGGGAC

AACATCAAGGTCGCCGACGACAAGGCCGACGTGCCGTCGCACCTCTACCGCTCCGCGCGG

GTCTTCAAGGAGGAGGCCGTCTACAACTTCCCGCTCACGGCCCCCGGCTGGCACTTCATC

CGGCTCTACTTCTTCCCCATCAAGAGCGGGGAGGCCGACCTCGCGGCGGCCACGTTCGAC

GTGTCCACCGCCGTTAACGTCCTTCTCCACGGCTTCACCCCCGAGGCGAAGGCGGTCATG

AAGGAGTACATCGTCAACGCCACGGAGAACAAGCTCGAGCTCAAGTTCACCCCGCAGTCG

GGCTCGGCGTTCATCAACGCCATCGAGGTCGTCAACGCCCCCGACGAGCTCATCAGCAAG

ACGGCCCTGACGGTGTCGCCGCTAGCCGAGACAAGCGGGTTGTCAGAGGCTGCGTACCAG

GTGGTGTGCCGGCTCAACGTCGGTGGCCCGCCCATCGGCCCCGTGAACGACACGCTCGGC

CGGCAGTGGGAGGACGACGGGCAGTACCTGAACCCCAAGGACGCCGGGACGGAGGTGTCG

GTGCCGACGAGCGCGATCAAGTACCCCGACGCGTTCCCGGCGACCAAGCTCGTGGCACCC

ACGGCGGTGTACGCGACCGCCCGCCACATGGCTGAATCCGGCGTCGCGAACCAGAACTTC

AACGTGTCGTGGAAGGTGGACGTGGACCCGTCGTTCGACTATCTCGTCCGCCTCTTCTTC

GCCGACATCATAAGCACGTCCGCCAACGACCTCTACTTCAACGCGTACATCAACGGCCGC

AAGGCCATCTCCGCCCTGGACCTCTCCACCATCACCGGCGACCTGGCCGCGCCCTACTAC

AAGGACTTCGTGGTGAACTCGTCGGTCAACACCGACGGCCACATTATCATCGGGGTCGGG

CCGCTGGGGCAGGACACGGGCCGCAACGACGCGCTGCTCAACGGCGCGGAGGTGCTCAAG

ATGAGCAACTCGGTGGGCAGCCTGGACGGCGAGTTCGGCGTGGACGGCCGGATGGTGGAC

GACGGCAGCGGCACCCGCAAAGTGGTCGCTGCCGTGGGGTTCGCCATGATGTTCGGCGCC

TTCGCCGGCCTGGGATGCATGGTGGTGAAGTGGCACCGGCGGCCGCAGGACTGGGACCGG

CGCAACAGCTTCTCGTCGTGGCTGCTGCCCATCCACACGGGCCAGTCCTTCTCCAACGGC

AAGGGGTCCAAGAGCGGCTACACCTTCTCCTCCACCGCGGGGCTGGGCCACTTCTTCACC

TTCGCGGAGATGTCAGAGGCGACCAAGAACTTCGACGAGAGCGCCATCATCGGCGTGGGA

GGGTTCGGCAACGTGTACGTGGGCGAGATCAACGACCCCGACGAGGAGGGGTCCAGGATC

AAGGTGGCCATCAAGCGCGGGAACCCGTCGTCGGAGCAGGGCATCAACGAGTTCAACACC

GAGATCCAGATGCTGTCCAAGCTCCGGCACCGCCACCTCGTGTCCCTCATCGGCTACTGC

GACGAGGGCGAGGAGATGATCCTCGTCTACGAGTTCATGCAGCACGGGCCCTTCCGCGAC

CACATCTACGGCGGCCCCGAGGGCCTGCCCACGCTCTCCTGGAAGCAGCGCCTCGAGATC

TGCATCGGCGCCGCCAGGGGCCTCCACTACCTCCACACCGGCACCGCGCACGGGATCATC

CACCGGGACGTCAAGACCACCAACATCCTCCTCGACGAAAAGTTCGTGGCCAAGGTGGCC

GACTTCGGCCTCTCCAAGGACGGCCCCGGGATGAACCAGCTGCACGTCAGCACCGCCGTC

AAGGGCAGCTTCGGGTACCTCGACCCGGAGTACTTCCGGTGCCAGCAGCTGACCGACAAG

TCGGACGTCTACTCCTTCGGGGTGGTGCTGCTGGAGACGCTGTGCGCGCGGGCGCCCATC

GACCCGCAGCTGCCGCGCGAGCAGGTCAGCCTCGCCGAGTGGGGCCTGCAGTGGAAGCGC

AAGGGCCTCATCGAGAAGATCATGGACCCCAACCTCAACGGCAAGGTCAACCCGGAGTCG

CTCGCCAAGTTCGCCGAGACCGCCGAGAAGTGCCTCTGCGAGTTCGGCAGCGACCGCCTC

TCCATGGGCGACGTGCTCTGGAACCTCGAGTACGCGCTGCAGCTGCAGGAGGCCAACCCG

CCCGAGGGCGCCACCGACGCCGACGACGCCGACGCCTCCATCGTCTCCTCCGCCAGCGGC

GTCACCACCGTGCCCGACCAGTCCACCACCTCCGCCAACGAGCTCTTCGCGCAGCTCGCC

GACATGAAGGGGAGATGA

>Ta-*CrRLK1L13*-B CDS sequence

ATGGTGCGCCGCGGGACGTTCCCGCTGGCGCTGCTGGCCGTGCTGGCGACGCTGACGGCC

GTGGCGGGGCAGGGGAAGCCGGTCACGGACAACGGCTCGGGCGGCGCGTCGGGGCCGGCC

AAGTTCACGCCCAAGGACGCCTTCTACATCGACTGCGGCGGCACGGCCGCCGCCGACACC

AAGGACGGCAAGTCCTTCAAGACCGACGCGGAGGCCAACAGCCTGCTCTCCGCCAGGGAC

AACATCAAGGTCGCCGACGACAAGGCCGACGTGCCGTCGCACCTCTACCGCAGCGCGCGG

GTCTTCAAGGAGGAGGCCGTCTACAACTTCCCGCTCACGGCCCCCGGCTGGCACTTCATC

CGGCTCTACTTCTTCCCCATCAAGAGCGGGGAGGCCGACCTCGCGGCGGCCACGTTCGAC

GTGACCACCGCCGTGAACGTCCTTCTCCATGGCTTCACCGCCGAGGCGAAGGCGGTCATG

AAGGAGTACGTCGTCAACGCCACGGAGAACAAGCTCGAGCTCAAGTTCACCCCGCAGTCG

GGCGCGGCGTTCATCAACGCCATCGAGGTCGTCAACGCCCCTGACGAGCTCATCAGTAAG

ACGGCCCTGACGGTGTCGCCGCTAGCCGAGACAAGCGGGCTGTCAGAGGCTGCCTACCAG

GTGGTGTGCCGGCTCAACGTCGGTGGCCCGCCCATCGGCCCCGTGAACGACACGCTCGGC

CGGCAGTGGGAGGACGACGGGCAGTACCTGAACCCCAAGGAGGCCGGGGCGGAGGTGTCG

GTGCCGACGAGCGCGATCAAGTACCCCGACGCGTTCCCGGCGACCAAGCTCGTGGCACCC

ACGGCGGTGTACGCGACCGCCCGCCACATGGCTGAATCCGGCGTCGCGAACCAGAACTTC

AACGTGTCGTGGAAGGTGGACGTGGACCCGTCGTTCGACTATCTCGTCCGCCTCTTCTTC

GCCGACATTATAAGCACGTCCGCCAACGACCTATACTTCAACGTGTACATCAACGGCCGC

AAGGCCATCTCCGCCCTGGACCTCTCCACCATCACCGGCGACCTGGCCGCGCCCTACTAC

AAGGACTTCGTGGTGAACTCGTCGGTCAATACCGACGGCCACATCATCATCGACGTCGGG

CCGCTAGGGCAGGACACGGGCCGCAACGACGCGCTGCTCAACGGCGCGGAGGTGCTCAAG

ATGACCAACTCGGTGGGCAGCCTGGACGGCGAGTACGGCGTGGACGGCCGGATGGTGGAC

GACGGCAGCGGCACCCGCAAGGTGGTGGCGGCCGTGGGGTTCGCCATGATGTTCGGCGCC

TTCGCCGGCCTGGGATGCATGGTGGTGAAGTGGCATCGGCGGCCGCAGGACTGGGAGCGG

CGCAACAGCTTCTCGTCGTGGCTGCTGCCGATCCACACGGGCCAGTCCTTCAGCAACGGC

AAGTCCAAGAGCGGTTACACCTTCTCCTCCACCGCGGGGCTGGGCCACTTCTTCACCTTC

GCGGAGATGTCAGAGGCGACCAAGAACTTCGCCGAGAGCGCCATCATCGGCGTGGGAGGG

TTCGGCAACGTGTACGTGGGCGAGATCAACGACCCCGACGAGGAGGGCTCCAGGATCAAG

GTGGCCATCAAGCGCGGGAACCCGTCGTCGGAGCAGGGCATCAACGAGTTCAACACCGAG

ATCCAGATGCTGTCCAAGCTCCGGCACCGCCACCTCGTCTCCCTCATCGGCTACTGCGAC

GAGGGCGAGGAGATGATCCTCGTCTACGAGTTCATGCAGCACGGGCCCTTCCGCGACCAC

ATCTACGGCGGGCCCGAGGGCCTGCCCACGCTCTCCTGGAAGCAGCGCCTCGAGATCTGC

ATCGGCGCCGCCAGGGGCCTCCACTACCTCCACACCGGCACCGCGCACGGGATCATCCAC

CGCGACGTCAAGACCACCAACATCCTCCTCGATGACAAGTTCGTGGCCAAGGTGGCCGAC

TTCGGCCTCTCCAAGGACGGCCCCGGGATGAACCAGCTGCACGTCAGCACCGCCGTCAAG

GGCAGCTTCGGGTACCTCGACCCGGAGTACTTCCGGTGCCAGCAGCTGACCGACAAGTCC

GACGTCTACTCATTCGGGGTGGTGCTGCTGGAGACGCTGTGCGCGCGGGCGCCCATCGAC

CCGCAGCTGCCGCGCGAGCAGGTCAGCCTCGCCGAGTGGGGCCTGCAGTGGAAGCGCAAG

GGCCTCATCGAGAAGATCATGGACCCCAACCTCGCCGGCAAGGTCAACCCGGAGTCGCTC

GCCAAGTTCGCCGAGACCGCCGAGAAGTGCCTCTGCGAGTTCGGCAGCGACCGCCTCTCC

ATGGGCGACGTGCTCTGGAACCTCGAGTACGCGCTGCAGCTGCAGGAGTCCAACCCACCC

GAGGGCGCCAGCGACGCCGACGACGCCGACGCCTCCATCGTCTCCTCCGCCAGCGGCGTC

ACCACCGTGCCCGACCAATCCACCACCTCCGCCAACGAGCTCTTCGCGCAGCTCGCCGAC

ATGAAGGGCAGGTGA

>Ta-*CrRLK1L13*-D CDS sequence

ATGGTGCGCCGCGGGGCGCTCCCGCTTGCGCTGCTGGCCGTGCTCGCGACGCTGACGGCC

GTGGCGGGGCAGGGGAAGCCGGTCACGGACAACGGCTCGGGCGGCGGGGCGGGGCCAGCC

AAGTTCACGCCCAAGGACGCCTTCTACATCGACTGCGGCGGCACGGCCGCCGCCGACACC

AAGGACGGCAAGTCCTTCAAGACCGACGCGGAGGCCAACAGCCTGCTCTCCGCCAGGGAC

AACATCAAGGTCGCCGACGACAAGGCCGACGTGCCGTCGCACCTCTACCGCTCCGCGCGG

GTCTTCAAGGAGGAGGCCGTCTACAACTTCCCGCTCACGGCCCCCGGCTGGCACTTCATC

CGGCTCTACTTCTTCCCCATCAAGAGCGGGGAGGCCGACCTCGCGGCGGCCACGTTCGAC

GTGAGCACCGCCGTCAACGTCCTTCTCCATGGCTTCACCCCCGAGGCGAAGGCGGTCATG

AAGGAGTACATCGTAAACGCCACGGAGAACAAGCTCGAGCTCAAGTTCACCCCGCAGTCG

GGCTCGGCGTTCATCAACGCCATCGAGGTCGTCAACGCCCCCGACGAGCTCATCAGTAAG

ACGGCCCTGACGGTGTCGCCGCTAGCCGAGACAAGCGGGTTGTCAGAGGCTGCGTACCAG

GTGGTGTGCCGGCTCAACGTCGGTGGCCCGCCCATCGGCCCCGTGAACGACACGCTCGGC

CGGCAGTGGGAGGACGACGAGAAGTACCTGAACCCCAAGGAGGCCGGGACGGAGGTGTCG

GTGCCGACGAGCGCGATCAAGTACCCCGACGCGTTCCCGGCGACCAAGCTCGTGGCACCC

ACGGCGGTGTATGCGACCGCCCGCCACATGGCTGAATCCGGCGTCGCGAACCAGAACTTC

AACGTGTCGTGGAAGGTGGACGTGGACCCGTCGTTCGACTATCTCGTCCGCCTCTTATTC

GCCGACATTATAAGCACGTCCGCCAACGACCTCTACTTCAACGTGTACATCAACGGCCGC

AAGGCCATCTCCGCCCTGGACCTCTCCACCATCACCGGCGACCTGGCCGCGCCCTACTAC

AAGGACTTCGTGGTGAACTCGTCGGTCAATACCGATGGCCACATTATCATCGACGTCGGG

CCGCTAGGGCAGGACACGGGCCGCAACGACGCGCTGCTCAACGGCGCGGAGGTGCTCAAG

ATGAGCAACTCGGTGGGCAGCCTGGACGGCGAGTACGGCGTGGACGGCCGGATGGTGGAT

GACGGCAGCGGCACCCGCAAAGTGGTGGCGGCCGTGGGGTTCGCCATGATGTTCGGCGCC

TTCGCCGGCCTGGGATGCATGGTGGTGAAGTGGCACCGGCGGCCGCAGGACTGGGAGCGG

CGCAACAGCTTCTCGTCGTGGCTGCTGCCCATCCACACGGGCCAGTCCTTCAGCAACGGC

AAGTCCAAGAGCGGCTACACCTTCTCCTCCACCGCGGGGCTGGGCCACTTCTTCACCTTC

GCGGAGATGTCAGAGGCGACCAAGAACTTCGACGAGAGCGCCATCATCGGCGTGGGAGGG

TTCGGCAACGTGTACGTGGGCGAGATCAACGACCCCGATGAGGAGGGGTCCAGGATCAAG

GTGGCCATCAAGCGCGGGAACCCGTCGTCGGAGCAGGGCATCAACGAGTTCAACACCGAG

ATCCAGATGCTGTCCAAGCTCCGGCACCGCCACCTCGTGTCCCTCATCGGCTACTGCGAC

GAGGGCGAGGAGATGATCCTCGTCTACGAGTTCATGCAGCACGGGCCCTTCCGCGACCAC

ATCTACGGCGGCCCCGAGGGCCTGCCCACGCTCTCCTGGAAGCAGCGCCTCGAGATCTGC

ATCGGCGCCGCCAGGGGCCTCCACTACCTCCACACCGGCACCGCGCATGGGATCATCCAC

CGCGACGTCAAGACCACCAACATCCTCCTCGACGACAAGTTCGTCGCCAAGGTGGCCGAC

TTCGGCCTCTCCAAGGACGGCCCCGGCATGAACCAGCTGCACGTCAGCACCGCCGTCAAG

GGCAGCTTCGGGTACCTCGACCCGGAGTACTTCCGGTGCCAGCAGCTGACCGACAAGTCC

GACGTCTACTCCTTCGGGGTGGTGCTGCTGGAGACGCTGTGCGCGCGGGCGCCCATCGAC

CCGCAGCTGCCGCGCGAGCAGGTCAGCCTCGCCGAGTGGGGCCTGCAGTGGAAGCGCAAG

GGCCTCATCGAGAAGATCATGGACCCCAACCTCAACGGCAAGGTCAACCCGGAGTCGCTC

GCCAAGTTCGCCGAGACCGCCGAGAAGTGCCTCTGCGAGTTCGGCAGCGACCGCCTCTCC

ATGGGCGACGTGCTCTGGAACCTCGAGTACGCGCTGCAGCTGCAGGAGGCCAACCCGCCC

GAGGGCGCCACCGACGCCGACGACGCCGACGCCTCCATCGTCTCATCCGCCAGCGGCGTC

ACCACCGTGCCCGACCAGTCCACCACCTCCGCCAACGAGCTCTTCGCGCAGCTCGCCGAC

ATGAAGGGGAGATGA

>Ta-*CrRLK1L14*-A CDS sequence

ATGCCCGCCGCGGGGCGCTCCGGTGGGCCGGGACAGGTCAACATTATGATGGGAAGGAGG

AAGTTGCAAGTGGTGACCTTGGCGATCTTGTGTTTCTGGTCATCTGCTGGGATCTGCAAA

GCACAATCAGTGGATTTCAAGCCTGCCGACAGCTACCTGGTTGACTGTGGGTCTGCCAAG

GGCACGACGGTTCTCGGGAGGGACTTCGCTGCCGATGGGGCAGCTCCGGTGACCGTGGCC

ACCTCCCAAGACATCCTTGCCGGCACCTCGGCCAACGGGGTGTCCTCGTTTGACAACCCG

GTGCTTTACCAGACTGCCCGCATCTTCACGAGCCCGTCATCCTATACTTTTCCTATCCAG

AAGCAGGGGCGGCATTTTGTTCGCCTCTACTTCTACCCCTTCATCTACCAGAGTTATGAT

CTCTCCACCGCCAAGTTCACCGTGTCGACCCAAGATGTGCTCCTGCTCAGTGATTTCCAG

CAGCCGGACAAGACGGCGCCACTGTTCAAGGAATACTCTTTGAACATCACACGTGACCAG

CTTGTCATTTCCTTCAAGCCGTCAAACGGAATTGCATTCATCAATGCAATTGAAGTGATT

TCTGTTCCAGATGATCTCATAGCCGATGTAGCCAATATGGTCAACCCTGTGCAGCAATAC

AGCGGTTTGACTACACAGTCACTGGAGACAGTGTATCGTGTCAACATGGGTGGTCCGAAG

GTCTTCCCGAACAATGATACCCTCTCGAGGACTTGGCAGAAGGATCAGAAGTACATACTG

AACCCCAGTGTGACTAAAACTGCTCAATATGGCAAGGCTATCAACTACAGGAAAGGTGGA

GCAACTCCACTGACGGCCCCTGATATTGTGTACAGTACAGCTACAGAATTGGCGGCTTCA

AACACATCCAACGCACTTTTCAACATGACATGGCAGTTTGATGTGGATGCAGGCTTCAGC

TATCTGATAAGATTTCACTTCTGTGATATAGTCAGCAAGGCACTGAACCAGCTCTACTTC

AATGCATATGTGGGAGGATTCTTTGCACAGCATGATCTTGATCTCTCAGAGCAATCGGTG

AATCAATTGGCTACAGCTATCTATGTTGATGTGGTTCTTTCTTCAAATGATGCGTCGAGC

AAGCTCAGCATCAGTATTGGTCCATCCACCTTGAACAATGCATTGCCTGATGGGATTCTG

AATGGCCTTGAGATTATGAAGATGGGCAGTGGCTCTGGTTCTGCTTTCACTGTTGGGAAT

AACGGTTCAAACAAAAAGTTGCCCATAATTATTGGCTCAGTCCTTGGGGTTGTTGGGCTT

CTGATAATTGTCCTTGTTGTGGTACTGCTTTGCCGGAGGAAGAAGACCGACGACAAGCAG

CACTCCAAGACCTGGATGCCTTTCTCTATCAATGGGCTCACGTCTCTCAGTACAGGAAGT

AGAACTTCTTATGGTACTACACTAACATCAGGTCTGAATGGAAGCTATGGATATCGCTTC

GCCTTCAATGTGCTCCAAGAAGCAACAAACAATTTTGATGAGAGCTGGGTGATTGGAGTT

GGAGGTTTTGGGAAAGTCTACAAGGGAGCTTTGAGGGATGACACAAAGGTTGCCGTGAAG

CGAGGAAACCCCAAGTCCCAGCAAGGTCTGAATGAGTTCCGGACAGAGATCGAACTCCTT

TCGCGTCTGCGTCATCGCCACCTGGTGTCTCTTATTGGGTACTGTGATGAAAGGAATGAG

ATGATCTTGGTCTATGAGTACATGGAGAACGGAACCGTCAAAAGCCACCTGTATGGTTCA

GACAACCCCTCACTCAACTGGAAGCAGCGGCTGGAGATCTGCATTGGAGCAGCAAGGGGG

CTACACTATCTTCATACCGGTTCTGCAAAGGCCATTATCCACCGTGATGTCAAGTCTGCA

AACATCTTGCTTGATGAGAATCTCCTCGCGAAAGTCGCCGACTTTGGGCTATCAAAGACT

GGGCCTGAGCTGGATCAGACTCACGTCAGCACTGCAGTGAAGGGCAGCTTTGGGTACCTC

GACCCTGAATACTTCCGAAGGCAGCAGCTGACCGAGAAGTCAGACGTCTACTCCTTCGGT

GTTGTCATGCTGGAGGTGCTCTGTGCGAGGCCGGTGATCGACCCTTCACTCCCGAGGGAA

ATGGTGAACTTGGCAGAGTGGGGAATGAAGTGGCAGAAGAGAGGGGAGCTGCACCAGATC

GTCGACCAGAAGCTTTCCGGCGCGATCAGGCCGGACTCTCTGAGGAAGTTCGGTGAGACG

GTGGAGAAGTGCCTGGCAGACTACGGCGTGGAGCGGCCGTCGATGGGGGACGTCCTCTGG

AACTTGGAGTATGTCCTGCAGCTCCAGGACGTGGATTCTTCGACCGTGTCGGACGTGAAC

AGCATGAACCGGATCGTCGACCTGTCATCGCAGGTTCAGCACGTCAGTGCCATGGAGAGC

ATCAGCGTGACGATGGCGGAGGACGGAGCTTTGCACGAGCCTGACCACGACCTCTCCGAC

GTGTCGATGAGCCGGGTTTTCTCTCAGCTGATCAAAGCCGAGGGGAGGTGA

>Ta-*CrRLK1L14*-B CDS sequence

ATGCCCGCCGCGGCGCGCTCCGGTGGACCGGGGCAGGCCAACATTATGATGGGGAGGAGG

AAGTTGCAAGCAGTGACCTTGGCGATCTTGTGTTTCTGGTCATCTGCTGGGGCACAAACA

GTGGATTTCAAGCCTGCCGACAACTACCTGGTTGACTGTGGGTCTGCCAAGGGCACGACG

GTTCTCGGGAGGGACTTCGCTGCCGATGGGGCATCTCCGGTGACAGTGTCAACCTCCCAA

GATATTCTTGCCGGCACCTCGGCCAACGGGGTGTCCTCGTTTGACAACCCGCTGCTTTAC

CAGACCGCCCGCATCTTCACGAGCCCGTCATCCTATACTTTTCCTATCCAGAAGCAGGGG

CGGCATTTTGTTCGTCTCTACTTCTTCCCCTTCATCTACCAGAGTTATGATCTCTCCACC

GCCAAGTTCACTGTGTCGACCCAAGATGTGCTCCTGCTCAGTGATTTCCAGCAGCCGGAC

AAAACTGCGCCGCTGTTCAAGGAATACTCTTTGAACATCACCCGTGACCAGCTTGTCATT

TCCTTCAAGCCGTCAAACGGGATTGCATTCATCAACGCAATTGAAGTGGTTTCTGTTCCA

GATGATCTCATAGCTGATGTAGCCAATATGGTCAACCCTGTGCAGCAGTACAGCGGTTTG

ACTACACAGTCACTGGAGACGGTGTATCGTGTCAACATGGGTGGTCCGAAGGTCTTCCCG

AGCAATGATACCCTCTCGAGGACTTGGCAGAAGGATCAGAAGTACATACTGAACCCCAGT

GTGACCAAAACTGCCCAATATGGCAAGCCTATCAAGTATAGGAAAGGCGGGGCAACTCCA

CTGACGGCCCCAGATATTGTGTACAGTACAGCTACAGAATTGGCGGCTGCAAACACTTCC

AACGCACTTTTCAACATGACATGGCAGTTTGATGTGGATGCAGGCTTCAGCTATCTGATA

AGATTTCACTTCTGTGATATAGTCAGCAAGGCACTGAACCAGCTCTACTTCAATGCATAT

GTGGGAGGCTTCTTTGCACAGCATGATCTTGATCTCTCAGAGCAATCGGTGAATCAACTG

GCTACAGCTATCTATGTTGACGTGGTTCTTTCTTCCAATGATGCATCTAGCAAGCTCAGC

ATCAGTATTGGTCCGTCCACCTTGAACAATGCATTGCCTGATGGGATTCTGAATGGCCTG

GAGATTATGAAGATGGGCAGTGGCTCTGGTTCTGCTTTCACTGTTGGGAATAACGGTTCA

AACAAAAGGTTGCCCATAATTATTGGCTCAGTCCTTGGGGTTGTTGGGCTTCTGATAATT

GTCCTTGTTGTGGTACTGCTTTGCCGGAGGAAGAAGACCGACGACAAGCAGCACTCGAAG

ACCTGGATGCCTTTCTCTATCAATGGGCTCACGTCTCTCAGTACAGGAAGCAGAACTTCC

TATGGTACTACACTAACATCAGGTCTGAATGGAAGCTATGGATATCGGTTTGCCTTCAAT

GTGCTCCAAGAAGCAACAAACAATTTTGATGAGAGCTGGGTGATTGGAGTCGGAGGTTTT

GGGAAAGTCTACAAGGGTGCCTTGAGGGATGACACAAAGGTTGCAGTGAAGCGAGGAAAC

CCCAAGTCCCAGCAAGGTCTCAATGAGTTCCGGACAGAGATCGAGCTCCTTTCACGTCTG

CGTCACCGCCACCTGGTGTCTCTTATTGGGTACTGTGATGAAAGGAATGAGATGATCTTG

GTCTATGAGTACATGGAGAACGGAACCGTCAAGAGCCACCTGTATGGTTCAGACAACCCC

TCACTCAACTGGAAGCAACGCCTGGAGATCTGCATTGGAGCAGCAAGGGGGCTACACTAT

CTTCATACAGGTTCTGCGAAGGCCATTATCCACCGTGATGTCAAGTCTGCAAACATCTTG

CTTGATGAAAATCTCCTTGCCAAAGTCGCCGACTTTGGGCTGTCAAAGACCGGGCCTGAG

CTGGATCAGACTCATGTCAGCACTGCAGTGAAGGGTAGCTTTGGTTACCTTGACCCTGAA

TACTTCCGAAGGCAGCAGCTGACTGAGAAGTCGGACGTCTACTCCTTCGGTGTTGTCATG

CTGGAGGTGCTCTGCGCGAGGCCGGTGATCGACCCTTCGCTCCCGAGGGAAATGGTGAAC

TTGGCAGAGTGGGGAATGAAGTGGCAGAAGAGAGGAGAGCTGCACCAGATCGTCGACCAG

AAGCTTTCCGGCGCGATCAGGCCGGACTCTCTGAGGAAGTTCGGTGAGACGGTGGAGAAG

TGCCTGGCAGACTACGGCGTGGAGCGGCCGTCGATGGGGGACGTCCTCTGGAACTTGGAG

TATGTCCTGCAGCTCCAGGATGTGGATTCTTCGACCGTGTCGGACGTGAACAGCATGAAC

CGGATCGTCGACCTGTCGTCGCAGGTTCAACATGTGGGTGCCATGGAGAGCATCAGCGTG

ACGATGGCGGAGGACGGAGCTTTGCACGAGCCTGACCACGACCTCTCCGACGTGTCGATG

AGCAGGGTTTTCTCACAGCTGATCAAAGCCGAGGGGAGGTGA

>Ta-*CrRLK1L14*-D CDS sequence

ATGGCAGGGGCCGCCGAGCGCACCTCTAGGCCACCGTCCAGCTCTACTCCCCTTTCCGCT

GCATTTGGCCGGAGGGGAGATCCCAGTCCCACCAGGACTGATTTCTACTTGGCCAACATT

ATGATGGGGAGGAGGAAGTTGCAAGTGGTGACCTTGGCGATCTTGTGTTTCTGGTCATCT

GCTGGGGTCTGCAAAGCACAAACAGTCGATTTCAAGCCTGCAGACAGCTACCTGGTTGAC

TGTGGGTCTACCAAGGGCACGACGGTTCTCGGGAGGGACTTCGCTGCCGATGGGGCATCT

CCGGTGACCGTGTCCACCTCCCAAGATATTCTTGCCGGCACCTCGGCCAACGGGGTGTCC

TCTTTTGACAACCCAGTGCTTTACCAGACCGCCCGCGTCTTCACGAGCCCGTCATCCTAT

ACTTTTCCGATCCAGAAGCAGGGGCGGCATTTTGTCCGTCTCTACTTCTACCCCTTCATC

TACCAGAGTTATGATCTCTCCACTGCCAAGTTCACCGTGTCGACCCAAGATGTGCTCCTG

CTCAGTGATTTCCAGCAGCCGGACAAGACGGCGCCGCTGTTCAAGGAATACTCTTTGAAC

ATCACCCGTGACCAGCTTGTTATTTCCTTCAAGCCGTCAAACGGAATTGCATTCATCAAC

GCTATTGAAGTGGTTTCTGTTCCAGATGATCTCATAGCAGATGTAGCCAATATGGTCAAC

CCTGTGCAGCAGTACAGCGGTTTGACTACACAGTCCCTGGAGACGGTGTATCGTGTTAAC

ATGGGTGGTCCGAAGGTCTTCCCGAACAATGATACCCTCTCGAGGACTTGGCAGAAGGAT

CAGAAGTACATACTGAACCCCAGTGTGACCAAAACTGCTGTATATGGCAAGGCTATCAAG

TACAGGAAAGGCGGGGCAACTCCACTGACGGCCCCAGATATTGTGTACAGTACAGCTACA

GAATTGGCGGCTTCAAACACATCCAACGCACTTTTCAACATGACATGGCAGTTTGATGTG

GATGCAGGCTTCAGCTATCTGATAAGATTTCACTTCTGTGATATAGTCAGCAAGGCACTG

AACCAGCTCTACTTCAATGCATATGTGGGAGGCTTCTTTGCACAGCATGATCTTGATCTC

TCAGAGCAATCGGTGAATCAACTGGCCACAGCTATCTATGTTGACGTGGTTCTTTCTTCC

AATGATGCATCTAGCAAGCTCAGCATCAGTATTGGTCCGTCCACCTTGAACAATGCATTG

CCTGATGGGATTCTGAATGGCCTTGAGATTATGAAGATGGGCAGTGGCTCTGGTTCTGCT

TTCACTGTTGGGAACAACGGTTCAAACAAAAAGTTGCCCATAATTATTGGCTCAGTCCTT

GGGGTTGTCGGGCTTCTGATAATTGTCCTTGTTGTGGTACTGCTTTGCCGGAGGAAGAAG

ACCGACGACAAGCAGCACTCCAAGACCTGGATGCCTTTCTCTATCAATGGGCTCACGTCT

CTCAGTACAGGAAGCAGAACTTCCTATGGTACCACACTAACATCAGGTCTGAATGGAAGC

TATGGATATCGCTTTGCCTTCAATGTGCTCCAAGAAGCAACAAACAATTTTGATGAGAGC

TGGGTGATTGGGGTCGGAGGTTTTGGGAAAGTCTACAAGGGTGCCTTGAGGGATGACACA

AAGGTTGCAGTGAAGCGAGGAAACCCCAAGTCCCAGCAAGGTCTCAATGAGTTCCGGACA

GAGATTGAGCTCCTTTCACGTCTGCGTCACCGCCACCTGGTGTCTCTGATTGGGTACTGT

GATGAAAGGAATGAGATGATCTTGGTCTACGAGTACATGGAGAACGGAACCGTCAAGAGC

CACCTGTATGGTTCAGACAACCCCTCACTCAACTGGAAGCAGCGGTTGGAGATCTGCATT

GGAGCAGCAAGGGGGCTACACTATCTTCATACTGGTTCTGCAAAGGCCATTATCCACCGT

GATGTCAAGTCTGCAAACATCTTGCTTGATGAGAATCTCCTTGCCAAAGTCGCCGACTTT

GGGCTGTCAAAGACTGGGCCTGAGCTGGATCAAACTCATGTCAGCACTGCAGTGAAGGGT

AGCTTTGGGTACCTTGACCCTGAATACTTCCGGAGGCAGCAGCTGACTGAGAAGTCGGAC

GTCTACTCCTTCGGTGTTGTCATGCTGGAGGTGCTCTGCGCGAGGCCGGTGATCGACCCT

TCGCTCCCGAGGGAAATGGTGAACTTGGCAGAGTGGGGGATGAAGTGGCAGAAGAGAGGG

GAGCTGCACCAGATCGTCGACCAGAAGCTTTCCGGCGCGATCAGGCCGGACTCTCTGAGG

AAGTTCGGCGAGACGGTGGAGAAGTGCCTGGCCGACTACGGCGTGGAGCGGCCGTCGATG

GGGGACGTCCTCTGGAATTTGGAGTATGTCCTGCAGCTCCAGGATGTGGATTCTTCGACC

GTGTCGGACGTGAACAGCATGAACCGGATCGTCGACCTGTCGTCACAAGTTCAGCATGTG

GGTGCCATGGAGAGCATCAGCGTGACGATGGCGGAGGACGGAGCTTTGCACGAGCCTGAC

CACGACCTCTCCGACGTGTCGATGAGCAGGGTTTTCTCTCAGCTGATCAAAGCTGAGGGG

AGGTGA

>Ta-*CrRLK1L15*-A CDS sequence

ATGAATTCCTCCGCCAATTTCCTGTCGATCCTGGTGCTGCTGGTGTTCTTGGCCGCGGGG

AATGCGCGAGCGCAGCCCCAGCCGATCCTCATCAACTGCGCCTCGGATTCCACCACCAGC

GTCGACGCCAGGACATGGATTGGGGATTCTTCCCCTTCCAACAACTTCACGCTCAGCTTC

CCCGGAGCCATCGCCTCGGCGGCTCCGGCTCCGGCTCCGGCTCCGGGAGTTGATGGAGAA

CAAGACCCGTACGGAGATTTGTACAAGACCGCCCGTGTCTTCAACGCCTCCTCCAGCTAC

AGGCTCGCCGTCGCCCCCGGGAGCTACTTCCTCCGCCTCCATTTCAGCCAGCAGTTCGCC

AATCTCGGCGCCCAGGAGCCCATCTTCAGTGTCGCGGCAAATGGCCTGAGGCTGCTCTCC

AAGTTCAGTGTCCACGGGGAGATTTCTTGGAGGGATTCCCAGATCAACTCAACCAGCAGC

GTCATCGTCAAGGAGTACCTTCTCAATGTCACTTCTGGTAAACTGGGCATTGAGTTCACC

CCCGATGAAGGGTCCTTCGCCTTCATCAATGCCATGGAGGTTCTCCCTGTGTCTGGCACC

TCTATTTTTGATTCAGTCAACAAGGTGGATGCTCATGGGTTGAAAGGCCCTTTTAGCCTC

GACGGCGGCGGGATCGAGACCATGTACAGGCTGTGTGTGGGATGCAGAGATGTACTGACG

AGGAAAGAGGATCCAGGATTGTGGAGGAGGTGGGATAAAGATGACCATTTCATATTCTCT

CTGAACGCCGCGAATTCCATCTTCAACTCTTCCAACATAAGTTATGTGTCTGCTGATGAT

CCCACGGTAGCCCCTTTGAGGCTCTATCAGAGTGCAAGGGTGCCAACAGAGAGTTCGGTC

TTGGGAAAGAAGTTCAATGTCTCATGGAGCTTTAACATTGACCCTGGCTTTGATTACTTG

GTCCGGCTGCATTTCTGCGAGCTGCAGTATGACAAGGCCGAGCAACGCAAGTTCAAGATT

TACATAAACAACAAGACCGCTGCAGAGGGCTATGATGTGCTTGCCAGAGCTGGGGGCAAG

AACAAGGCCTTTTATGAAGACTTCCTTGATGCTGCCTCACCGCAGATGGACACTCTTTGG

GTTCAGCTGGGGTCTGAGTCTTCAGCAGGTTCCGCGGCTGCTGATGCTCTTCTCAATGGC

ATGGAGATCTTTAAGGTCAGCCGGGAAGGAAATCTTGCCCATCCAACCGTCAGGATTGGA

GGCTTCAGTGGTGGCACAAGCAAACCAAAACGGAGCCCCAAGTGGGTGCTAATTGGTGCT

GCTTCCGGTCTGATAATTTTTATCGCAATTGCTGCTGCTCTTTATTTATGTTTCAATCTG

CGACGGAAGAAAAATAGTTCAGCCAGCAAAGCCAAGGACAATCCCCATGGTGCTGCACAT

ACCCGTTCTCCAACTCTTCTCACGGCTGGGGCATTTGGGAGCAAAAGGATGGGCAGGCGG

TTCACCATTGCAGAAATCAGAACAGCCACTGTGAACTTTGATGAGTCCTTGGTGATTGGG

GTTGGAGGCTTTGGCAAGGTCTACAGGGGTATAATGGAGGATGGCACTCGGGTGGCAATT

AAGAGGGGTTACACAGATTCTCACCAGGGTCAGGGTGTGAAGGAATTCGAAACTGAGATC

GAGATGCTCTCAAGGTTGCGGCACCGGCACCTTGTGCCCTTGATTGGCTATTGTGATGAG

CAAAACGAGATGGTCTTAGTTTATGAGCACATGGCAAATGGCACATTAAGGAGCCATCTT

TATGGAAGTGACCTTCCTGCTCTTACATGGAAGCAAAGGCTCGAAATATGTATCGGCGCA

GCACGAGGGCTTCACTACCTTCACACTGGGCTTGACAGAGGTATAATCCACAGGGACGTC

AAGACTACCAACATTTTGTTAGACAACAACCTTGTTGCCAAGATGGCAGATTTTGGCATC

TCAAAAGATGGTCCAGCTTTAGATCATACTCATGTTAGTACTGCTGTCAAAGGGAGTTTT

GGTTACCTCGATCCAGAGTACTATAGGAGACAGCAGTTAACGCCAAGTTCAGATGTGTAC

TCTTTTGGTGTCGTGCTGTTTGAAGTGCTGTGTGCTCGACCAGTCATAAATCCAACCCTG

CCAAGAGACCAGATAAACCTTGCTGACTGGGCTCTCAACAGGCAAAGGCACAAGTTACTT

GAGACCATAATCGACCTTCGATTGGATGGAAATTACACACTGGAGTCCATCAGAACATTC

AGCGAGATAGCAGAAAAATGCCTTGCAGATGAGGGGGTGAACCGGCCTTCGATGGGCGAA

GTCCTCTGGCACCTAGAGAGTGCTTTGCAGTTGGAACAAGGTCATCTGCAAAGCACAAAT

GGTGATGGTTGTTCAGACCCTCAACTGAAGCCTTCTGATGTACCTACCCATGTGGCGTGC

ATCAAAGAAGTTGAGCAATCCACTCGTCCAGGCTCCCACGATTCAGATGGGCAAGTTGTC

GATGTCAAGATTGAGGTGCCATGA

>Ta-*CrRLK1L15*-B CDS sequence

ATGAAATCTTCCGCGAATTTCCTGTCGATCCTGGTGCTGCTGGTGTTCCTGGCCGCGGGG

AATGCGCGAGCGCAGCCCCAGCCGATCCTCATCAACTGCGGCTCGGATTCCACCACCAGC

GTCGATGCCAGGACATGGATTGGGGATTCTTCCCCTTCCAACAACTTCACGCTCAGCTTC

CCGGGAGCCATCGCCTCGGCGGCTCCGGCTCCGGGAGTTGATGGAGAACAGGACCCGTAC

GGAGATTTGTACAAGACCGCCCGTGTCTTCAACGCCTCCTCCAGCTACAGGCTCGCCGTC

GCCCCCGGGAGCTACTTCCTCCGCCTCCATTTCAGCCAGCAGTTCGCCAATCTCGGCGCC

CAGGAGCCCATCTTCAATGTCGCGGCAAATGGCCTGAGGCTGCTCTCCAAGTTCAGTGTC

CACGGAGAGATTTCTTGGAGGGATTCCCAGATCAATTCAACTAGCAGCGTCATCGTCAAG

GAGTACCTTCTCAATGTCACTTCTGGTAAACTGGGCATTGAGTTCACCCCCGATGAAGGA

TCCTTCGCCTTCATCAATGCCATGGAGGTTCTACCTGTGTCTGGCACCTCAATTTTTGAT

TCAGTCAACAAGGTGGACGGTCATGGGTTGAAAGGCCCTTTTAGCCTCGACGGCAGCGGG

ATCGAGACCATGTACAGGCTGTGTGTGGGATGCATCGATGTACTGGCGAGGAAAGAGGAT

CCAGGATTGTGGAGGAGGTGGGATAAAGATGAGCATTTCATATTCTCTCTCAACGCCGCG

AGTTCCATCTTCAACTCTTCCAACATAAGTTATGTGTCTGCTGATGATCCCACAGTAGCC

CCTTTGAGGCTCTATCAGAGTGCAAGGGTGCCAACAGAGAGTTCGGTCTTGGGAAAGAAG

TTCAATGTCTCATGGAGCTTTAACATTGACCCTGGCTTTGATTACTTGGTCCGGCTGCAT

TTCTGCGAGCTGCAGTATGACAAGGCTGAGCAACGCAAGTTCAAGATTTACATAAACAAC

AAGACCGCTGCAGAGGGCTATGATGTGTTTGCCAGAGCTGGAGGGAAGAACAAGGCCTTT

TATGAAGACTTCCTTGATGCTGCCTCACCGCAGATGGACACTCTTTGGGTTCAGCTGGGG

TCTGAGTCTTCAGCAGGTTCCGCGGCTGCTGATGCTCTTCTGAATGGCATGGAGATCTTT

AAGGTCAGCCGGGAAGGAAATCTTGCCCATCCAACCGTCAGGATTGGAGGCATCAGTGGC

GGCGCAAGGAAACCAAAACGGAGCCCCAAGTGGGTGCTAATTGGTGCTGCTTCCGGTCTG

ATAATTTTTATCGCAATTGCTGGTGCTCTTTATTTCTGTTTCAATCTGCAAAGGAAGAAA

AATAGTTCGGCCAACAAAGCCAAGGACAATCTCCATGGTGTTACACATACCCGTTCTCCA

ACTCTTCGCACGGCTGGGGCATTTGGGAGCAAAAGGATGGGCAGGCGGTTCACCATTGCA

GAAATCAGAACAGCCACCGTGAACTTTGATGAGTCCTTGGTGATTGGGGTTGGAGGCTTT

GGCAAGGTCTACAGGGGTATAATGGAGGATGGCACTCGGGTGGCAATTAAGAGGGGTTAT

ACAGATTCTCACCAGGGTCAGGGTGTGAAGGAATTCGAAACTGAGATCGAGATGCTCTCA

AGGTTGCGGCACCGGCACCTTGTGCCCTTGATTGGCTATTGTGATGAGCAAAACGAGATG

GTCTTAGTTTATGAGCACATGGCAAATGGCACATTAAGGAGCCATCTTTATGGAAGTGAC

CTTCCTGCTCTTACATGGAAGCAAAGGCTCGAAATATGTATCGGCGCAGCACGAGGGCTT

CACTACCTTCACACTGGGCTTGACAGGGGTATAATCCACAGGGATGTCAAGACTACCAAC

ATTTTGTTGGACGACAACCTTGTTGCCAAGATGGCAGATTTTGGCATCTCAAAAGATGGT

CCAGCTTTAGATCATACTCATGTTAGTACTGCTGTCAAAGGGAGTTTTGGTTACCTCGAT

CCAGAGTACTATAGGAGACAGCAGTTAACGCCAAGTTCAGATGTGTACTCTTTTGGTGTC

GTGCTGTTTGAAGTGCTGTGTGCTCGACCAGTCATAAATCCAACCCTGCCAAGAGACCAA

ATAAACCTTGCTGACTGGGCTCTCAACAGGCAAAGGCACAGGTTACTTGAGACCATAATC

GACCTTCGATTGGATGGAAATTACACACTGGAGTCCATCAAGATATTCAGCGAGATAGCA

GAAAAATGCCTCGCAGATGAGGGGGTGAACCGGCCTTCGATGGGCGAAGTCCTCTGGCAC

CTAGAGAGTGCTTTGCAGTTGGAACAAGGTCATCCGCAAAGCACAAATGGTGATGGTTGC

TCAGACCCTCAACTGAAGCCTTCTGATGTACCTACCCGTGTGGCGTGCATCAAAGAAGTT

GAGCAATCCACTCGTCCAGGCTCCCACGATTCAGATGGGCAAGTTGTTGATGTCAAGATT

GAGGTGCCATGA

>Ta-*CrRLK1L15*-D CDS sequence

ATGAAATCCTCCGCCAATTTCCTGTCGATCCTGGTGCTGCTGGTGTTCCTGGCCGCGGAG

AATGCGCGGGCGCAGCCCCAGCCGATCCTCATAAACTGCGGCTCGGATTCCACCACCAGC

GTCGATGCCAGGACATGGATTGGGGATTCTTCCCCTTCCAACAACTTCACGCTCAGCTTC

CCCGGGGCCATCGCCTCGGCGGCTCCGGCTCCGGCTCCGGGAGTTGATGGAGAACAAGAC

CCGTACGGAGATTTGTACAAGACCGCCCGTGTCTTCAACGCCTCCTCCAGCTACAGGCTC

GCCGTCGCCCCCGGGAGCTACTTCCTCCGCCTCCATTTCAGCCAGCAGTTCGCCAATCTC

GGCGCCCAGGAGCCCATCTTCAGTGTCGCGGCAAATGGCCTGAGGCTGCTCTCCAAGTTC

AGCGTCCACGGAGAGATTTCTTGGAGGGATTCTCAGATCAACTCAACGAGCAGCGTCATC

GTCAAGGAGTACCTTCTCAATGTCACTTCTGGTAAACTGGGCATTGAGTTCACCCCCGAT

GAAGGGTCCTTCGCCTTCATCAATGCCATGGAGGTTCTACCTGTGTCTGGCACCTCAATT

TTTGATTCAGTCAACAAGGTGGATGCTCATGGGTTGAAAGGCCCTTTTAGCCTCGACGGC

GACGGGATCGAGACCATGTACAGGCTGTGTGTGGGATGCATCGATGTACTGCCGAGGAAA

GAGGATCCAGGATTGTGGAGGAGGTGGGATAAAGATGAGCATTTCATATTCTCTCTCAAC

GCCGCGAATTCCATCTTCAACTCTTCCAACATAAGTTATGTGTCTGCTGATGATCCCACA

GTAGCCCCTTTGAGGCTCTATCAGAGTGCAAGGGTGCCAACAGAGAGTTCGGTCTTGGGA

AAGAAGTTCAATGTCTCATGGAGCTTTAACATTGACCCTGGCTTTGATTACTTGGTCCGG

CTGCATTTCTGCGAGCTGCAGTATGACAAGGCTGAGCAACGCAAGTTCAAGATTTACATA

AACAACAAGACCGCTGCAGAGAGCTATGATGTGTTTGCCAGAGCTGGGGGCAAGAACAAG

GCCTTTTATGAAGACTTCCTTGATGCTGCCTCACCTCAGATGGACACTCTTTGGGTTCAG

CTGGGGGCTGAGTCTTCAGCAGGTTCCGCGGCTGCTGATGCTCTTCTCAATGGCATGGAG

ATCTTTAAGGTCAGCCGGGAAGGAAATCTTGCCCATCCAACCGTCAGGATTGGAGGCATC

AGTGGTGGTGCAAGCAAACCAAAACGGAGCCCCAAGTGGGTGCTAATTGGTACTGCTTCC

GGTCTGATAATTTTTATCGCAATTGCTGGTGGTCTTTATTTTGGTTTCAATCTGCGACGG

AAGAAAAATAGTTCAGCCAGCAAAGCCAAGGACAATCTCCATGGTGCTACACATACGCGT

TCTCCCACTCTTCGCACAGCTGGGGCATTTGGGAGCAACAGGATGGGCAGGCGGTTCACC

ATTGCAGAAATCAGAACAGCCACCGTGAACTTTGATGAGTCCTTGGTGATTGGGGTTGGA

GGCTTTGGCAAGGTCTACAAGGGTATAATGGAGGATGGCACTCGGGTGGCAATTAAGAGG

GGGCATACAGATTCTCACCAGGGTCAGGGTGTGAAGGAATTCGAAACTGAGATCGAGATG

CTCTCAAGGTTGCGGCACCGGCACCTTGTGCCCTTGATTGGCTATTGTGATGAGCAAAAC

GAGATGGTCTTAGTTTATGAGCACATGGCAAATGGCACATTAAGGAGCCATCTTTATGGA

AGTGACCTTCCTGCTCTTACATGGAAGCAAAGGCTTGAAATATGTATCGGCGCAGCACGA

GGGCTTCACTACCTTCACACCGGGCTTGACAGGGGTATAATCCACAGGGATGTCAAGACT

ACCAACATTTTGTTAGACGACAACCTTGTTGCCAAGATGGCAGATTTTGGCATCTCAAAA

GATGGTCCAGCTTTAGATCATACTCATGTTAGTACTGCTGTCAAAGGGAGTTTTGGTTAC

CTCGATCCAGAGTACTATAGGAGACAGCAGTTAACGCCAAGTTCAGATGTGTACTCTTTT

GGTGTCGTGCTGTTTGAAGTGCTGTGTGCTCGACCAGTCATAAATCCAACCCTGCCAAGA

GACCAAATAAACCTTGCTGACTGGGCTCTGAACAGGCAAAGGCACAGGTTACTTGAGACC

ATAATCGACCTTCGATTGGATGGAAATTACACACTGGCGTCCGTCAAGAAATCCAGCAAG

ATAGCAGAAAAATGCCTGGCAGATGAGGGGGTGAACCGGCCTTCGATGGGCGAAGTCCTC

TGGCACCTAGAGAGTGCTTTGCAGTTGGAACAAGGTCATCCGCAAAGCACAAATGCTGAT

GGTTGTTCAGACCCTCAACTGAAGCCTTCTGATGTACCTACCCGTGTGGCGTGCATCAAA

GAAGATGAGCAATCCACTCGTCCAGGCTCCCACAATTCAGATGGGCAAGTTGTTGATGTC

AAGATTGAGGTGCCATGA

Protein sequences:

>Ta-CrRLK1L1-A

MPALAILARSMAPCKRVPMFLILFILSITRVATTNAIASKVDRFVPQDNYLLSCGASAAVQVDDGRTFRSDPESVSFLSTLTDIKIAAKASLASASPLSPLYLDARVFSDISTYSFFISQPGRHWIRLYFLPITDSQYNLTTATFSVSTDSMVLLHDFSFIASPPNPVFREYLVSAQGDNLKIIFTPKKNSIAFINAIEVVSAPPSLIPNTTTRMGPQDQFDISNNALQVVYRLNMGGALVTSFNDTLGRTWQPDAPFLKLEAAAEAAWVPPRTIKYPDDKTLTPLIAPASIYSTAQQMASTNITNARFNITWQMVAEPGFRYLIRLHFSDIVSKTLNSLYFNVYINGMMAVANLDLSSLTMGLAVAYYKDLIAESSSIINSTLLVQVGPNTIDSGDPNAILNGLEIMKISNEASSLDGLFSPKTSSEVSKTTLTGIAFALAATAALAVVICYRRNRKPEWQRTNSFHSWFLPLNSSSSFMSSCSRLSRNRFGSTRTKSGFSSVFASSAYGLGRYFTFVEIQKATKNFEEKGVIGVGGFGKVYLGATEDGTQLAIKRGNPSSDQGMNEFLTEIQMLSKLRHRHLVSLIGCCDENNEMILVYEFMSNGPLRDHLYGDTNIKPISWKQRLEVCIGAAKGLHYLHTGSAQGIIHRDVKTTNILLDENFVAKVADFGLSKDAPSLEQTHVSTAVKGSFGYLDPEYFRRQQLTDKSDVYSFGVVLFEVLCARPAINPALPRDQVNLAEWARTWHRKGELGKIIDPNIAGQIRSDSLEMFAEAAEKCLADYGVDRPTMGDVLWKLEFALQLQEKGDVVDGTSDGIAMKSLEVTNVDSMEKSGNAIPSYVQGR

>Ta-CrRLK1L1-B

MPALAILARSSRMAEWERVPMFLILFILSITSVATTNAIASKVDRFVPQDNYLLSCGASAAVQVDDGRTFRSDPESVSFLSTPTDIKIAAKASLASASPLSPLYLDARVFSDISTYSFFISQPGRHWIRLYFLPITDTQYNLTTATFSVSTESMVLLHDFSFIASPPNPVFREYLVSAQGDNLKIIFTPKKNSIAFINAIEVVSAPPSLIPNTTTRMGPQDQFDISNNALQVVYRLNMGGALVTSFNDTLGRTWLPDAPFLKLEAAAEAAWVPPRTIKYPDDKTLTPLIAPASIYSTAQQMASTNITNAKFNITWVMVAEPGFRYLIRLHFSDIVSKTLNSLYFNVYINGMMAVANLDLSSLTMGLAVAYYKDLIAESSSIINSTLVVQVGPSTIDSGDPNAILNGLEIMKISNEASSLDGLFSPKTSSEASKRTLTGIAFALAATAALAVVICYRRNRKPAWQRTNSFHSWFLPLNSSSSFMSSCSRLSRNRFGSTRTKSGFSSVFASSAYGLGRYFTFVEIQKATKNFEEKGVIGVGGFGKVYLGATEDGTQLAIKRGNPSSDQGMNEFLTEIQMLSKLRHRHLVSLIGCCDENNEMILVYEFMSNGPLRDHLYGDTNIKPISWKQRLEVCIGAAKGLHYLHTGSAQGIIHRDVKTTNILLDENFIAKVADFGLSKDAPSLEQTHVSTAVKGSFGYLDPEYFRRQQLTDKSDVYSFGVVLFEVLCARPAINPSLPRDQVNLAEWARTWHRKGELGKIIDPNIAGQIRPDSLEMFAEAAEKCLADYGVDRPTMGDVLWKLEFALQLQEKGDVVDGASDGIPMKSLEVSNVDSMEKSGNAIPSYVQGR

>Ta-CrRLK1L1-D

MPALAILARSMAECKRVPMFLILFILSITSVATTNAIASKVDRFVPQDNYLLSCGASAAVQVDDGRTFRSDPESVSFLSTPTDIKIAAKASLASASPLSPLYLDARVFSDISTYSFFISQPGRHWIRLYFLPITDSQYNLTTATFSVSTDSMVLLHDFSFIASPPNPVFREYLVSAQGDNLKIIFTPKKNSIAFINAIEVVSAPPSLIPNTTTRMGPQDQFDISNSALQVVYRLNMGGALVTSFNDTLGRTWQPDAPFLKLEAAAEAAWVPPRTIKYPDDKTLTPLIAPASIYSTAQQMASTNITNARFNITWQMAAEPGFRYLIRLHFSDIVSKTLNSLYFNVYINGMMAVANLDLSSLTMGLAVAYYKDLIAESSSIINSTLVVQVGPNTIDSGDPNAILNGLEIMKISNEANSLDGLFSPKTSSEVSKTTLTGIAFALAATAALAVVICYRRNRKPAWQRTNSFHSWFLPLNSSSSFMSSCSRLSRNRFGSTRTKSGFSSVFASSAYGLGRYFTFVEIQKATKNFEEKGVIGVGGFGKVYLGATEDGTQLAIKRGNPSSDQGMNEFLTEIQMLSKLRHRHLVSLIGCCDENNEMILVYEFMSNGPLRDHLYGDTNIKPISWKQRLEVCIGAAKGLHYLHTGSAQGIIHRDVKTTNILLDENFVAKVADFGLSKDAPSLEQTHVSTAVKGSFGYLDPEYFRRQQLTDKSDVYSFGVVLFEVLCARPAINPALPRDQVNLGEWARTWHRKGELGKIIDPNIAGQIRPDSLEMFAEAAEKCLADYGVDRPTMGDVLWKLEFALQLQEKGDVVDGASDGIAMKSLEVTNVDSMEKSGNAIPSYVQGR

>Ta-CrRLK1L2-A

MVLPTLPVTLTFLTLLALLSIAKAADNNSTTSGLILLNCGESTQDDDDGGRSWDGDTGSIFAPSMKGDAAIALGQPPSLTPRVPYTTARIFTSNYTYSFPVSPGRMFLRLYFLSTAYEYYAVSDAVFGVTARNLVLLKDFNALQTAQAITSAYLVREFSVNVSSGSLDLTFAPSAHQYGSYAFVNGIEIVPTPDIFATPDIRFVSGDNTSPFTFDADMSLQTMYRLNVGGPAISPKGDSGFYRSWANDAPYILGGFGLTFWKNDNLTISYTSRVPNYTAPVDVYGTARSMGPTAQINLNYNLTWILPVDAGFFYLLRFHFCEIKYPITKVNQRSFFIYINNQTAQEQMDVIFRSGGIGRPTYTEYVIMAIGSGQVDMWIALHPDLSSKPQYSDAILNGLEVFKLQNYGPSNLAGLNPPLPQKPDVNPNRLSSGERKTKGGIQATIGGTAGGFALLLIALFSMCVIYRRKKAAKSPGKTDYGHVKHPTKCIKSTCDLVRHFSFAKIQVATKDFDEALIIGRGGFGNVYIGDIDGGTKVAIKRCDQKSQQGFHEFQTEIEMLCNFRHRHLVSLIGYCEEKNEMILVYDYMAHGTLREHLYNTRNPPLPWQQRLEICIGAARGLHYLHTGVEQGIIHRDVKTTNILLDDRLMAKVSDFGLFKASPDIGNTHMSTAVKGTFGYLDLEYFRQQRLTKKSDVYSFGVVLFETLCARPVINTELPYEQVSLRDWVVSCRKKGVLEEIVDPCVKEEITLECFRIFAEIAEKCVADRSIDRPSMGDVLWNLEVALQLQDSASYNTSCAEGASSLQISGVHSGKPSTNSTISVAAQEAIFSDIAHPEGR

>Ta-CrRLK1L2-B

MVLPTLPVTLTFLTLLALLSIAKAADNNSTTSGLILLNCGSSTQNDDDSGRTWDGDTGSKFAPSMKGVAAIALGQTPSLTPRVPYTTARIFTSNYTYSFPVSPGRMFLRLYFFSTAYEYYAVSDAVFGVTSRNLVLLNDFNALQTAQAITSAYLVREFSVNVSSGSLDLTFAPSAQQYGSYAFVNGIEIVPTPDIFATPDIRLVSGDNTSPFTFDADMSLQTMYRLNVGGPAISTEGDSGFYRSWANDAQYILGGSGLTFWKNDNLTISYTSRVPNYTAPVDVYGTARSMGPTAQINLNYNLTWIFPVDAGFFYLLRFHFCEIKYPITKVNQRSFFIYINNQTTQKQMDVIVRSGGIGRPTYTEYVIMAIGSRQVDMWIALHPDLSSKPQYSDAILNGLEVFKLQNYGPSNLAGLSPPLPQKPDVNPTRLSNGERKSKGGIQAIIGGTTGGFALLLIALFSMCVIYRRKKVAKSPGKTDYGHVKHPTKCIKSTCDLVRHFSFAKIQVATKDFDEALIIGRGGFGNVYIGDIDGGTKVAIKRCDQKSQQGFHEFQTEIEMLCNFRHRHLVSLIGYCEEKNEMILVYDYMAHGTLREHLYNTRNPPLPWQQRLEICIGAAQGLHYLHTGVEQGIIHRDVKTTNILLDDRLMAKVSDFGLSKASPDIGNTHMSTAVKGTFGYLDPEYFRLQRLTKKSDVYSFGVVLFETLCARPVINTELPYEQVSLRDWALSCWKNGVLEEIVDPRVKEEITPECFRVFAEIAEKCVADRSIERPSMGDVLWNLEVALQLQQASASYNSNRAEGASSLQISAVHSDKPSTNSTISIAAQEAIFSDIAHAEGR

>Ta-CrRLK1L3-A

MQTSCSKLIRWSPQFFDSGAPTAANSKMAFPALPATLTCLTLLALLSLAMAADNNSTGLILINCGASVQEDDDNGRTWDGDTGSKFAPSLKGVTATAPNQDPSLPSTVPFMTARIFASNYTYSFSVTPGRVFLRLYFYPVAYPNYAVADAFFSVTTPNLVLLNDFNASQTVQAISSAYLVREFSVNVSSGSSLDLTFAPSAHHNGSYAFVNGIEIVSTPDIFTAPDTRYVGDNTSPFTFDSAMAVQTMYRLNVGGQAISPKGDSGFYRSWANDAPYIFGGSGVTFSKDDNLTITYTSKVPNYTAPVDVYGTARSMGPTAPINLNYNLTWILPVDAGFSYLLRFHFCEIQYPITKQNQRSFFIYINNQTAQEQMDVIVWSGGIGRTTYTDYVILTAGSGQVDMWIALHPDLSSRPEYFDAILNGLEIFKLQNYGASNNLAGLNPPLPQKPADASPGAASGKVKSVAAIIGGAVGGFVVLLVTCFGICIICKRKNKSKKKKKISKDPGGKSEDGHWTPLTEYSGSRSAMSGNTATTGSTLPSNLCRHFTFAELQTATKNFDQAFLLGKGGFGNVYLGEIDSGTKVAIKRCNPMSEQGVHEFQTEIEMLSKLRHRHLVSLIGYCEDKSEMILVYDYMAHGTLREHLYNTKNPPLSWKQRLEICIGAARGLYYLHTGVKHTIIHRDVKTTNILLDDKWVAKVSDFGLSKTGPNMDATHVSTVVKGSFGYLDPEYFRRQQLSEKSDVYSFGVVLFEVLCARPALSPSLPKEQISLADWALRCQKQGVLGQVIDPVLQGKIAPQCFLKFTDTAEKCVADRSVDRPSMGDVLWNLEFALQLQESEEDTGSLTEGTLSSSGASPLVMTRLQSDEPSMDASTTTTSTTTMSMTGRSIASMDSDGLTPSAVFSQIMHPDGR

>Ta-CrRLK1L3-B

MQTSCSKLIRWSPQFFDSGTPTTAKSKMAFPALPVTLTCLTLLALLSLAMAADNNSTGLILVNCGASTQEADDSGRTWVGDTGSKFAPLLKGVATTAPNQDPSLPSTVPFMTARIFTSNYTYSFSVNPGRMFLRLYFYPVAYANYAVSDAFFSVTTRNLVLLNDFSASQTAQAITSAFLVREFSVNVSSGSSLDLTFAPSAHRNGSYAFVNGIEIVPTPDIFTAPDTRYVGDNTAPFSFDAGMAVQTMYRLNVGGQAISPKGDSGFYRSWANDAPYIFGGSGVTFSKDDNLTITYTSNVPNYTAPVDVYGTARSMGPTAQINLNYNLTWILPVDAGFSYLLRFHFCEIQYPITKQNQRSFFIYINNQTAQEQMDVIVWSGGIGRTAYTDYVIMAVGSGQVDMWIALHPDLSSKPEYFDAILNGLEIFKLQNYGSPNNLSGLNPPLPQKPTDASPGAASGKMKSVAAIIGGAVGGFAVLLVTCFGVCIICKRKNKKNKKKISKDPGGKSEDGHWTPLTEYSGSRSAMSGNTATTGSTLPSNLCRHFTFAELQTATKNFDQAFLLGKGGFGNVYLGEIDSGTKVAIKRCNPMSEQGVHEFQTEIEMLSKLRHRHLVSLIGYCEDKSEMILVYDYMAHGTLREHLYNTKNPPLSWKKRLEICIGAARGLYYLHTGVKHTIIHRDVKTTNILLDDKWVAKVSDFGLSKTGPNMDATHVSTVVKGSFGYLDPEYFRRQQLSEKSDVYSFGVVLFEVLCARPALSPSLPKEQISLADWALRCQKQGVLGQVMDPVLQGKIAPQCFLKFTDTAEKCVADRSVDRPSMGDVLWNLEFALQLQESEEDTGSLTEGTLSSSGASPLVMTRLQSDEPSTDASTTTTTTTTMSMTGRSIASVDSDGLTPSAVFSQIMHPDGR

>Ta-CrRLK1L3-D

MAFPALPVTLTCLILLSLLSLAMAADNNSTGLILVNCGASVQGDDDSGRTWDGDTGSKFAPSLKGVAATAPNQDPSLPSTVPFMTARIFTSNYTYSFSVKPGRMFLRLYFYPVAYPNYAVSDAFFSVTTPKLVLLNDFSASQTAQAITSAFLVREFSVNVSSGSSLDLTFAPSAHRNGSYAFVNGIEIVPTPDIFTAPDTRNVGDNTAPFSFDTSSSLQTMYRLNVGGQAISPKGDLGGFYRSWANDAPYIAGGSGVTFSKDDNLTITYTSKVPKYTAPPDVYGTARSMGPTAQINLNYNLTWILPVDAGFFYLLRFHFCEIQYPIIKINQRSFFIYINNQTAQEQMDVIVWSGGIGRTTYTDYVIMAAGFGQVDMWIALHPDLSSRPEYFDAILNGLEVFKLQNYGSPNNLSGLNPPLPQKPADASPSAASGKMKSVAAIIGGAVGGFIVLLAACFGVCIICKRKNKKKKKKKTSKDPGGKSEDGHWTPLTEYSGSRSAMSGNTATTGSTLPSNLCRHFTFADLQTATKNFDQAFLLGKGGFGNVYLGEIDSGTKVAIKRCNPMSEQGVHEFQTEIEMLSKLRHRHLVSLIGYCEDKSEMILVYDYMAHGTLREHLYNTKNPPLSWKQRLEICIGAARGLYYLHTGVKHTIIHRDVKTTNILLDDKWVAKVSDFGLSKTGPNMDATHVSTVVKGSFGYLDPEYFRRQQLSEKSDVYSFGVVLFEVLCARPALSPTLPKEQISLADWALRCQKQGVLGQVIDPVLQGKIAPQCFLKFTDTAEKCVADRSVDRPSMGDVLWNLEFALQLQESEEDTGSLTEGTRSSSGASPLVMTRLQSDEPSTDASTTTTSTTTMSMTGRSIASMDSDGLTPSAVFSQIMHPDGR

>Ta-CrRLK1L4-A

MAATARLRRARPRGVLGLVSALLVCGAAAYAPEDNYLVSCGSSLDTPVGRRLFLADDGGSGSGAVTLTSPRSAAVKASPDLVSGFRDAALYQNARVFSAPSSYSFAIRRRGRHFLRLHFFPFVYRSYDLAAAARAFKVSTQDAVLLEDGVPAPEPGNASTSSSPQPARVEFLLDVARDTLVVSFVPLVDGGIAFVNAVEVVSAPDGLVADAAESSTGRPEPIPAALPLQTAYRLNVGGPAVAPDDDALWREWTTDLRFLSHSVADAVTREVRYNGTPNRLPGQATATDAPDVVYATARELVINSSSFDGQKQMAWQFDVDASSSYFIRFHFCDIVGKAPHQLHINAYVDDASHATVLTDLDLAAVGDGALAFPYYKDFVLPASEASGKLAVHVGPLANKIVMPAAILNGIEIMKMHLSAGSVVVVEPAAGAAKSRFAVLLGSVCGPLAFVSIAVALAIVLRKKRKKEGEEEEESDKKQPTPTQSQSSTPWMPLLGRLSVRGAIASGSSSFTTAGNTPGTSPRAAAAVMPSYRFPLAVLQDATRNFDDSLIIGEGGFGKVYGAVLQDGTKVAVKRASPESRQGAREFRTEIELLSGLRHRHLVSLVGYCDEREEMILLYEYMEHGSLRSRLYGRGGAAPLSWAQRLEACAGAARGLLYLHTAVDKPVIHRDVKSSNILLDGDLTGKVADFGLSKAGPVLDETHVSTAVKGSFGYVDPEYCRTRQLTAKSDVYSLGVVLLEAVCARPVVDPRLPKPMSNLVEWGLHWQGRGELEKIVDRRIAAAARPAALRKYGETVARCLAERGADRPAMEDVVWNLQFVMRLQEGDGLDFSDVSSLNMVTELTPPPRRQRSAVDSDGLALSDVSSLNMVTELTPPQTGSVEGDGVADDDFTDASMRGTFWQMVNVRSR

>Ta-CrRLK1L4-B

MAATARLRPARARGVLWSVSVWLVCGAAAYAPEDNYLVSCGSSLDTPVGRRLFLADDGGSGSGSGAVTLTSPRSAAVKAPPDLVSGFRDAALYQNARVFSAPSSYSFAIRRRGRHFLRLHFFPFVYRSYDLAVAARAFKVSTQDAVLLEDGVPAPEPGNASTSTSPQPARVEFLLDVARDTLVVSFVPLVDGGIAFVNAVELVSVPDDLVADAADSSTGRPEPIPAALPLQTAYHLNVGGPAVAPDDDALWREWTTDQPLSDPRVDAVTREVRYNRTLNRLPGQATATDAPDIVYATARELVINRSSFDGQKQMAWQFDVDAGSSYFIRFHFCDIVSEAPHQLHINAYVDDASHATVLTDLDLAAVGDGALAFPYYKDFVLPASEASGKLAVHVGPLANKIVMPAAILNGIEIMKMHLSAGSVVVVEPAAGAAKSRLAVILGSVCGALAFVSIAIALAIVLRKKKGEGEEGVKEQPTPTRSQSSTPWMPLLGRLSVRGAIASGSSSFTTAGNTPGASPRAAAAAAAAVVPSYRFPLAMLQDATRNFDDSLIIGEGGFGKVYGAVLQDGTKVAVKRASPESRQGAREFRTEIELLSGLRHRHLVSLVGYCDEREEMILLYEYMEHGSLRSRLYGRGGAAPLSWAQRLEACAGAARGLLYLHTAVDKPVIHRDVKSSNILLDGDLTGKVADFGLSKAGPVLDETHVSTAVKGSFGYVDPEYCRTRQLTAKSDVYSLGVVLLEAVCARPVVDPRLPKPMSNLVEWGLHWQGRGELEKIVDRRIAAVARPAALRKYGETVARCLAERGADRPAMEDVVWNLQFVMRLQEGDGLDFSDVSSLNMVTELTPPRRQRSAVDCDGLDFSDVSSLNMVTELTPPKTGSMEGDGVADDDDFTDASMRGTFWQMVNVRSR

>Ta-CrRLK1L4-D

MAATARLRPARARGVLWVVSVLLVCGAAAYKPEDNYLVSCGSSLDTPVGRRLFLADDGASGAVTLTSPRSAAVKAPPDLVSGFRDAALYQNARVFSAPSSYSFAIRRRGRHFLRLHFFPFVYRSYDLAAAARAFKVSTQDAVLLEDGIPAPEPGNASTSTSPQPARLEFLLDVARDTLVVSFVPLADGGIAFVNAVELVSVPDGLVADAADSSTGRPEPIPAVLPLQTAYRLNVGGPAVAPDDDALWREWTTDLRFLSHSVADAVTREVRYNGMLNRLPGQATATDAPDIVYATARELVINGSSFDGQKQMAWQFDVDTSSSYFIRFHFCDIVGKAPHQLHINAYVDDATVKQDLDLAAVGDGALAFPYYTDFVLPASEASGKLAVHVGPLANKIVMPAAILNGIEIMKMHLSAGSVVVVQPAAGAAKSRFAVVLGSVCGALAFISVAVALAIVLRKKEKEKEVEEGAKEQPTPTQSQSSTPWMPLLGRFSVRGAIASGSSSFTTAGNTPGASPRAAAAAAVMPSYRFPLAMLQDATRNFDDSLIIGEGGFGKVYGAVLQDGTKVAVKRASPESQQGAREFRTEIELLSGLRHRHLVSLVGYCDEREEMILLYEYMEHGSLRSRLYGRGGAAPLSWAQRLEACAGAARGLLYLHTAVDKPVIHRDVKSSNILLDGDLAGKVADFGLSKAGPVLDETHVSTAVKGSFGYVDPEYCRTRQLTAKSDVYSLGVVLLEAVCARPVVDPRLPKPMSNLVEWGLHWQGRGELEKIVDRRIAAAARPAALRKYGETVARCLAERGADRPAMEDVVWNLQFVMRLQEGDGLDFSDVSSLNMVTELTPPRRQRSAVDHDGLDYSDVNSLNMVTELTPPQTGSVEGDGEADDDFTDASMRGTFWQMVNVRSR

>Ta-CrRLK1L5-A

MRGGPRCALLLLVAAAALVPAARAQGATAPAPSAGAPFVPRDDILLDCGATGKGNDTDGRQWDGDAGSKYAPPNLASASAGAQDPSVPQVPYLTARVSAAPFTYSFPLGPGRKFLRLHFYPANYSNRDAADAFFSVSVPAAKVTLLSNFSAYQTTTALNFAYIVREFSVNVTGQNLDLTFTPEKGHPNAYAFINGIEVVSSPDLFGIATPQFVTGDGNSQPYEMDPAAALQTMYRLNVGGQAISPSKDSGGARSWDDDTPYIYGAGAGVSYQNDPNVTIIYPDNVPGYVAPSDVYATARSMGPDKGVNMAYNLTWILQVDAGYQYLVRLHFCEIQSPFTKPNQRVFNIYLNNQTAIEGADVIQWADPNGIGTPVYKDYVVSTVGSGILDFWVALHPDAETKPQYYDAILNGMEVFKLQLTNGSLVGLNPVPSADPPAHSGSGDKKSKVAPIVGGVIGGLAVLALGYCCFICKRRRKAAKASGMSDGHSGWLPLSLYGHSHTSSSAKSHATGSYASSLPSNLCRHFSFAEIKAATKNFDESRILGVGGFGKVYHGEIDGGTTKVAIKRGNPLSEQGIHEFQTEIEMLSKLRHRHLVSLIGYCEEKNEMILVYDYMAHGTLREHLYKTQNAPLSWRQRLEICIGAARGLHYLHTGAKHTIIHRDVKTTNILLDEKWVAKVSDFGLSKTGPSMDHTHVSTVVKGSFGYLDPEYFRRQQLTEKSDVYSFGVVLFEVLCARPALNPTLAKEEVSLAEWALHCQKKGILDQIVDPYLKGKIVPQCFKKFAETAEKCVADNGIERPSMGDVLWNLEFALQMQESAEESGSIGCGMSDEGTPLVMVGKKDPNDPSIDSSTTTTTTTSLSMGDQSVASIDSDGLTPSAVFSQIMNPKGR

>Ta-CrRLK1L5-B

MRGGPRCALLLLAALAAAAALAPAAWAQGATAPAPSAGPPFVPRDDILLDCGATGKGNDTDGRQWDGDAGSKYAPPNLASATAGAQDPSVPQVPYLTARVSAAPFTYSFPLGPGRKFLRLHFYPANYSNRDAADAFFSVSVPAAKVTLLSNFSAYQTTTALNFAYIVREFSVNVTGQNLDLTFTPEKGHPNAYAFINGIEVVSSPDLFDLATPQLVTGDGNSQPYEMDPAAALQTMYRLNVGGQAISPSKDSGGARSWDDDTPYIYGAGAGVSYQNDPNVTITYPDNVPGYVAPSDVYATARSMGPDKGVNMAYNLTWILQVDAGYQYLVRLHFCEIQSPFTKPNQRVFNIYLNNQTAMEGADVIQWADPNGIGTPVYKDYVVSTVGSGIMDFWVALHPDAGTKPQYYDAILNGMEVFKLQLTNGSLVGLNPVPSADPPAHSGSGDKKSKVAPIVGGVIGGLAVLALGYCCFICKRRRKAAKASGMSDGHSGWLPLSLYGHSHTSSSAKSHATGSYASSLPSNLCRHFSFAEIKAATKNFDESRILGVGGFGKVYHGEIDGGTTKVAIKRGNPLSEQGIHEFQTEIEMLSKLRHRHLVSLIGYCEEKNEMILVYDYMAHGTLREHLYKTQNAPLSWRQRLEICIGAARGLHYLHTGAKHTIIHRDVKTTNILLDEKWVAKVSDFGLSKTGPSMDHTHVSTVVKGSFGYLDPEYFRRQQLTEKSDVYSFGVVLFEVLCARPALNPTLAKEEVSLAEWALHCQKKGILDQIVDPYLKGKIVPQCFKKFAETAEKCVADNGIERPSMGDVLWNLEFALQMQESAEESGSIGCGMSDEGTPLVMVGKKDPNDPSIDSSTTTTTTTSLSMGDQSVASIDSDGLTPSAVFSQIMNPKGR

>Ta-CrRLK1L5-D

MRGGPRCALLLLVAAAALVPAARAQGATAPAPSSGVPFVPRDDILLDCGATGKGNDTDGRQWDGDAGSKYAPPKLASASAGAQDPSVPQVPYLTARVSAAPFTYSFPLGPGRKFLRLHFYPANYSNRNAADAFFSVSVPAAKVTLLSNFSAYQTTTALNFAYIVREFSVNVTGQNLDLTFTPEKGHPNAYAFINGIEVVSSPDLFDLATPQLVTGDGNSQPYEMDPAAALQTMYRLNVGGQAISPSKDSGGARSWDDDTPYIYGAGAGVSYQNDPSVAITYPDNVPGYVAPSDVYATARSMGPDKGVNMAYNLTWILQVDAGYQYLVRLHFCEIQSPYTKPNQRVFNIYLNNQTAMQGADVIQWADPNGIGTPVYKDYVVSTVGSGIMDFWVALHPDAETKPQYYDAILNGMEVFKLQLTNGSLVGLNPVPSADPPAHSGSGDKKSLVAPIVGGVIGGLAVLALGYCCFICKRRRKAAKASGMSDGHSGWLPLSLYGHSHTSSSAKSHATGSYASSLPSNLCRHFSFAEIKAATKNFDESRILGVGGFGKVYHGEIDGGTTKVAIKRGNPLSEQGIHEFQTEIEMLSKLRHRHLVSLIGYCEEKNEMILVYDYMAHGTLREHLYKTQNAPLSWRQRLEICIGAARGLHYLHTGAKHTIIHRDVKTTNILLDDKWVAKVSDFGLSKTGPSMDHTHVSTVVKGSFGYLDPEYFRRQQLTEKSDVYSFGVVLFEVLCARPALNPTLAKEEVSLAEWALHCQKKGILDQIVDPYLKGKIVPQCFKKFAETAEKCVADNGIERPSMGDVLWNLEFALQMQESAEESGSIGCGMSDEGTPLVMVGKKDPNDPSIDSSTTTTTTTSLSMGDQSVASIDSDGLTPSAVFSQIMNPKGR

>Ta-CrRLK1L6-A

MAVHVVLPLLLLLLVATVLPYTALAAFSPDFKIFLACGAGADVPFPSDNPARTFVRDDGYLSQGGAAAVSASASSNAASPLYAAARADTSAFSYRLTYPAAPDASSFLVLRLHFFPFVPASSSSTSLSSARFTVSVLDAYALLPAFSPPADGVVKEFFVPRGASGGDFTVRFAPEAGSSAFVNAVELFSAPPELLWNNTAVPVDPVGSNDLPEWPLDALETVYRLNVGGPLLTNGNDTLWRTWLPDDPYLFGAPGQSVVNNTPSPIIYAPSNGYTQEVAPDVVYKTQRAANVTDLLQATTPGFNFNVTWTFPADQGSRYLVRLHFCDYEVVSSVVGTVIVFNVYVAQAIGTPDLTPSARARQSNEAFYIDYAAMAPRAGNLTVSIGRSKKSSKGGILNGLEIMKLQTVNLSSTGSHGRTKRIVIIVLATVLGAAVLASAVLCFFVVRRRKRRQVAPPGSTEDKESTQLPWSPYTQEGISGWADESANRSSEGTTARMQRVSTKLHISLAELKAATDNFHDRNLIGVGGFGNVYKGALADGTPVAVKRAMRASKQGLPEFHTEIVVLSGIRHRHLVSLIGYCNEQAEMILVYEYMEKGTLRGHLYGGSDDEPPLSWKQRLEICIGAARGLHYLHSGYSENIIHRDVKSTNILLGTDGGGSTGGGAIIAKVADFGLSRIGPSLGETHVSTAVKGSFGYLDPEYFKTQQLTDRSDVYSFGVVLFEVLCARPVIDQSLDRDQINIAEWAVRMHGEGKLDKIADARIAGEVNDNSLRKFAETAERCLADYGADRPSMGDVLWNLEYCLQLQETHVNRDAFEDSGAVATQLPADVVVPRWVPSSTSLLMMDDADETGLSMTDLADSQVFSQLNARGEGR

>Ta-CrRLK1L6-B

MAVHVVPPLLLLLATALPYSALAVFSPDFSFFLACGAGADVTFPSDNPTRTFVRDDGYLSQGRPAAVSANASSGAASNPLYAAARADSSAFSYRLAYPATAGASSFLVLRLHFFPFVPASSSTSLSSARFTVSVLDAYALLPAFSPPADGVVKEFFVPRGGSKEFTIRFSPDAGSSAFVNAVELFPAPQQLLWNGSNSVVPVGVLGNDDLAQWQLDALETVYRLNVGGPKVTRENDTLWRTWLPDGAYLFGAPGQSVVNNTSSPIIYNPPNTREVAPDVVYRTQRAANVTDFLRATTPGLNFNVTWTFPAEAGSRYLVRLHFCDYEVVSSVVGVGIVFNVYVAQAIGSRDLAPNAQATQPNEPLYLDYAATAPRAGNLTVSIGTSSKSSGGGILNGLEIMKLQSVDLSSPGSHALTKRSIIIIVLATVLGAAVLACAVLCFFVVRRRKRRQVAPPASKEDKESTQLPWSPYTQEGISGWADESTNRSNEGTTARMQRVSTKLHISLPELKAATDNFHERNLIGVGGFGNVYKGALSDGTPVAVKRAMRASKQGLPEFQTEIVVLSGIRHRHLVSLIGYCNEQAEMILVYEYMEKGTLRSHLYGSDEPVLSWKQRLEICIGAARGLHYLHSGYSENIIHRDVKSTNILLGTDDGGSTGGGAIIAKVADFGLSRIGPSLGETHVSTAVKGSFGYLDPEYFKTQQLTDRSDVYSFGVVLFEVLCARPVIDQSLDRDQINIAEWAVRMHGEGKLDKIADARIAGEVNDNSLRKFAETAEKCLADYGADRPSMGDVLWNLEYCLQLQETHVNRDAFEDSGAVATQLPADVVVPRWVPSSTSLLMMDDADETGLSMTELADSQVFSQLNARGEGR

>Ta-CrRLK1L6-D

MAVHVVPPLLLLLLATALPYTALAVFSPDFSFFLACGAGADVPFPSDNPTRTFVRDDGYLSQGRPAAVSASASSGAASNPLYAAARADSSAFSYRLAYPATAGASSFLVLRLHFFPFVPASSSTSLSSARFTVSVLDAYALLPTFSPPVAGVVKEFFVPRDGSKDFTIRFTPDAGSSAFVNAVELFSAPPELLWNNTAVPVDPVGSNDLPEWPLDALETVYRLNVGGPMVTKENDTLWRTWLPDGPYLFGAPGQSVVNSTSSPIMYDPSNGYTQDVAPDVVYRTQRAANVTDLLVATTPGLNFNVTWTFPAEQGSRYLVRLHFCDYEVVSSVVGVGIVFNVYVAQAIGTPALSPKDRARQSNEAFYMDYAARAPRAGNLTVSIGWLRQSSGGGILNGLEIMKLQSADPSLTVSHGLTKRSIIIIVLATVLGAAVLACAVLCFFVVRRTKRRQVAPPASTEDKESTQLPWSPYTQEGISGWADESTNRSSEGTTARMQRVSTKLHISLAELKAATDNFHDRNLIGVGGFGNVYKGALADGTPVAVKRAMRASKQGLPEFHTEIVVLSGIRHRHLVSLIGYCNEQAEMILVYEYMEKGTLRSHLYGGSDDEPPLSWKQRLEICIGAARGLHYLHCGYSENIIHRDVKSTNILLGTDDGGSTGGGAIIAKVADFGLSRIGPSLGETHVSTAVKGSFGYLDPEYFKTQQLTDRSDVYSFGVVLFEVLCARPVIDQSLDRDQINIAEWAVRMHGEGKLDKIADARIAGEVNDNSLRKFAETAERCLADYGADRPSMGDVLWNLEYCLQLQETHVNRDAFEDSGAVATQLPADVVVPRWVPSSTSLLMMDDADEAGLSMTELADSQVFSQLNARGEGR

>Ta-CrRLK1L7-B

MLWNSSVTPVGAVVKDDMDLWQRQPLETVYRLNVGGPKVTIENDTLWRTWLPDGPYLYDASGLSVVSNTSNPIIYDSSNGYTREVAPDVVYQTQRMANVTDLLAATTPGLNFNLTWTFPAVKGSHYLVRLHFCDYEVVSSVVGVGIVFNVYIAQTIGTPDLTPNARATQSNEVFYMDYAARAPSTGNLTVSIGWSSKRSGGGILNGLEIMRLPPVDLSSRRYGRTKRTIVITVSAVLGAAVLACVVLCFFGVPYTKYSGSGWAEQFTNRWSREGKTSGLQSVSTKLHIALAKIKAATDNFHERNLIGVGGFGNVYKGVLVDGTPVAVKRAMRASQQGLPEFQTEIVVLSGIRHRHLVSLIGYCNEQAEMILVYEYMEKGTLRSHLYGSDEPALSWKQRLEICIGAARGLHYLHRGYAENIIHRDVKSTNILLGSDGGSTGGVIAKVADFGLSRIGPSFGETHVSTAVKGSFGYLDPGYFKTQQLTDRSDVYSFGVVLLEVLCARPVIDQSLDHGRINIAEWAVRMRREGRLDKMADPRIAGEVDEESLLKFAETAEKCLAECWVDRPSMGDVLWNLEYCLQLQETNITGDGLDDMVPSSTSLLMDETDLSMTNVADSKVFSQLSARGEGR

>Ta-CrRLK1L7-D

MAVRGILLALLLAMVLPRAILAAFSPGFQYFLACGANSAVSFPSDSPANIFVPDAAYLSPAGAPAVSASSTLASPPALYAAARADISAFSYRLPSPASPDTSSFLVLRLHFFPSFPATSSQYVINILSARFNVSVADAYALLSSFSPPAAGVVKEFFVPRDLFDGHFHVTFTPDAGSTAFVNAIELFSAPPEMLWNGPVTPVGAVVKDDMDLWQRQPLETVYRLNVGGPKVIIENDTLWRTWLPDGPYLYDASGLSVVSNTSSPIIYDSSNGYTREVAPDVVYQTQRMANVTDLLAATTPGLNFNLTWTFPAVKGSRYLVRLHFCDYEVVSSVVGVGIVFNVYIAQAIGTPDLTPNARATQSNEVFYKDYAARAPSAGNLTVSIGWSSKSSGGGILNGLEIMRLPPVDLSSRRYGRTKRTIVITVSAVLGAAVLACVVLCFFGVPYTKYSGSGWAEQFMNRWSRERKTGGMESVSRKLHIALAKIKAATDNFHERNLIGVGGFGNVYKGVLGDGTPVAVKRAMRASQQGLPEFQTEIVVLSGIRHRHLVSLIGYCNEQAEMILVYEYMEKGTLRSHLYGSDEPALSWKQRLEICIGAARGLHYLHRGYAENIIHRDVKSTNILLGSDDGSTGGVIAKVADFGLSRIGPSFGETHVSTAVKGSFGYLDPGYFKTQQLTDRSDVYSFGVVLLEVLCARPVIDQSLDHGRINIAEWAVRMRREGRLDKMADPRIAGEVDEESLLKFAETAEKCLAEAWVDRPSMGDVLWNLEYCLQLQETNITGDELDDMVPSSTSLLMDETGLSMTNVADSKVFSQLSARGEGR

>Ta-CrRLK1L8-A

MPPVPDMLVRLLVASVLLGAASGAFTPADTYLVLCGTSASATVAAGRTFVGDARLPAKSLAAPQSVEANTSLTAVVPSGESQLYRSARVFTAPASYTFAVKQPGRHFVRLHFFPFPYRSYDMAADAAFNVSVQGAVLVNGYAPKNGTAELREFSLNVTGATLVIAFAPTGKLAFVNAIEVVSVPDELIADTARMVGGAVQYTGLSTQALETIHRINMGVPKITPGNDTLGRTWLPDQSFQLNTNLAQHKDAKPLTIKYDEKSALSSAYTAPAEVYATATRLSTAGETSTINVQFNISWRFDAPAGSDYLLRFHWCDIVSKAAMGMAFNVYVGGSVVLENYEISRDTFNRLSIPVYKDFLLGAKDAKGAITVSIGSSTEDNALPDGFLNGLEIMRVVGSAGAGAPAASARSSKVKIGIIAGSAVCGATLVMVLGFIAFRTLRGREPEKKQPSDTWSPFSASALGSRSRSRSFSKSNGNTVLLGQNGAGAGYRIPFAALQEATDGFDEAMVIGEGGFGKVYKGTMRDETLVAVKRGNRRTQQGLHEFHTEIEMLSRLRHRHLVSLIGYCDERGEMILVYEYMAMGTLRSHLYGAGLPPLSWEQRLEACIGAARGLHYLHTGSAKAIIHRDVKSANILLDESFMAKVADFGLSKNGPELDKTHVSTKVKGSFGYLDPEYFRRQMLTEKSDVYSFGVVLLEVLCARTVIDPTLPREMVSLAEWATPCLRNGRLDQIVDQRIAGTIRPGSLKKLADTAEKCLAEYGVERPTMGDVLWCLEFALQLQVGSSDGSDVDTMLPPAPPVPVKTPEVQRSLSAATMATDAAAMTTNLGDLDGMSLSGVFSKMIKSDEVR

>Ta-CrRLK1L8-B

MPPFPDMLVRLLLASVLLSAASGAFTPADNYLVICGTSASATVAPRRSFVGDARLPAKSLAAPQSVEANTSLTAVVPSGESELYRSARVFTAPASYTFAVKQPGRHFVRLHFFPFSYRSYDMAADAAFNVSVQGAVLVNGYTPKNGTAELREFSVNVTGGTLVIAFAPTGKLAFVNAIEVVSVPDELIADTARTVGGAVQYTGLSTQALETIHRINMGIPKITPGNDTLGRTWLPDQSFQLNTNLAQHKDAKPLTIKYDEKSALSSPFTAPAEVYATATRLSTAGETSTINVQFNISWRFDAPAGSDYLLRFHWCDIVSKAAIGMAFNVYVGGSVVLDNYEISRDTFNRLSIPVYKDFVLGAKDAKGAITVSIGSSTEDNTLPDGFLNGLEIMRVVGSASAGAGAPAASPPSSKVKIGIIAGSAVCGATLVTVLGFIAFRMLRGREPEKKQPSDTWSPFSASALGSRSRSRSFSKSNGNTVLLGQNGAGAGYRIPFAALQEATGGFDEGMVIGEGGFGKVYKGTMRDETLVAVKRGNRRTQQGLHEFHTEIEMLSRLRHRHLVSLIGYCDERGEMILVYEYMAMGTLRSHLYGAGLPPLSWEQRLEACIGAARGLHYLHTSSAKAIIHRDVKSANILLDESFMAKVADFGLSKNGPELDETHVSTKVKGSFGYLDPEYFRRQMLTEKSDVYSFGVVLLEVLCARTVIDPTLPREMVSLAEWATPCLRNGQLDQIVDQRIAGTIRPGSLKKLADTADKCLAEYGVERPTMGDVLWCLEFALQLQVASSDVSDADTMLTPPVPVKTPEVQRSLSAATVATDAAMTTNLGDLDGMSLSGVFSKMIKSDEVR

>Ta-CrRLK1L8-D

MPPVLDMLVRLLVASVLLGAASGAFTPADNYLVLCGTSASATVAAGRTFVGDARLPAKSLAAPQSVEANTSRTAVVPSGESELYRSARVFTAPASYTFAVKQPGRHFVRLHFFPFPYRSYDMVADAAFNVSVQGAVLVNGYTPKNGTAELREFSVNVTGGTLVIAFAPTGKLAFVNAIEVVSVPDELIADMARMVDGAVQYTGLSTQALETIHRINMGVPKITPGNDTLGRTWLPDQSFQVNTDLAQHKDAKPLTIKYDEKSALSSAYTAPAEVYATATRLSTAGETSTINVQFNISWRFDAPAGSDYLLRFHWCDIVSKAAMGMAFNVYVGGAVVLDNYEISRDTFNRLSIPVYKDFLLGAKDAKGAITVSIGSSTEDNALPDGFLNGLEIMSIVGSAGAGAAATSPRSSKVKIGIIAGSAVCGATLVMVLGFIAFKMLRGREPEKKKPADAWSPFSASALGSRSRSRSFSKSNGNTVLLGQNGAGAGYRIPFAALQEATGGFDEGMVIGEGGFGKVYKGTMRDETVVAVKRGNRRTQQGLHEFHTEIEMLSRLRHRHLVSLIGYCDERGEMILVYEYMAMGTLRSHLYGAGLPPLSWEQRLEACIGAARGLHYLHTGSAKAIIHRDVKSANILLDESFMAKVADFGLSKNGPELDKTHVSTKVKGSFGYLDPEYFRRQMLTEKSDVYSFGVVLLEALCARTVIDPTLPREMVSLAEWATPCLRNGQLDQIVDQRIAGTIRPGSLKKLADTAEKCLAEYGVERPTMGDVLWCLEFALQLQVGSSDSSDVDTMLPPAPPVPVKTPEVQRRLSAATVATDAAAMTTNLGDLDGMSLSGVFSNMIKSDEVR

>Ta-CrRLK1L9-A

MGGGPRCALLLLVAAAAAALVPAAWAQDPTAPAPSGAPFVPRDDILLDCGATGKGNDTDGREWRGDAGSKYAPPNLASADAGAQDPSVPQVPYLTARVSAAPFTYSFPLGPGRKFLRLHFYPANYSGRAAADAFFSVSVPAAKVTLLSNFSAYQTATAFNFAYLVREFSVNVTGPTLDLTFTPEKGRPNAYAFINGIEVVSSPDLFDLATPFFVTGDGNNQPFPMDPGAALQTMYRLNVGGQAISPSKDSGGARSWDDDTPYIYGAGAGVSYPNDPNVTITYPPSVPGYVAPLDVYATARSMGIDKGVNLAYNLTWIVQVDAGFTYLVRLHFCEIQSPIDKPNQRVFNIYLNNQTAVEGADVLQWVDPRSTGTPLYKDFVVGTVGSGIMDFWVALHPDIRNKPQYYDAILNGMEVFKLQLTNGSLAGPNPVPSADPAAHTGQGKKSSLVGPIAGGVIGGLAVLALGYCCFICKRRRKVAKDAGMSDGHSGWLPLSLYGNSHTSSSAKSHATGSIASSLPSNLCRHFSFAEIKAATKNFDESRILGVGGFGKVYQGEIDGGTTKVAIKRGNPLSEQGIHEFQTEIEMLSKLRHRHLVSLIGYCEDKNEMILVYDHMAHGTLREHLYKTQNAPLSWRQRLEICIGAARGLHYLHTGAKHTIIHRDVKTTNILLDEKWVAKVSDFGLSKTGPSMDHTHVSTVVKGSFGYLDPEYFRRQQLTEKSDVYSFGVVLFEVLCARPALNPTLAKEEVSLAEWALHCQKKGILDQIVDPYLKGKIVPQCFKKFAETAEKCVADNGIERPSMGDVLWNLEFALQMQESAEESGSFGCGMSDEEGAPLVMAGKKDPNDPSIDSSTTTTTTTSLSMGDQSVASIDSDGLTPSAVFSQIMNPKGR

>Ta-CrRLK1L9-B

MGGGPRCALLLLAAAAACAALVPAAWAQGSTAPAPSGAPFVPRDDILLDCGATGKGNDTDGREWRGDASSKYAPPNLASADAGAQDPSVPQVPYLTARVSAAAFTYSFPLGPGRKFLRLHFYPANYSNRDAADAFFSVSVPAAKVTLLSNFSAYQTATALNFAYLVREFSVNVTGPTLDLTFTPEKGRPNAYAFINGIEVVSSPDLFDLATPFFVTGDGNNQPFPMDPGAALQTMYRLNVGGQAISPSKDSGGARSWDDDTPYIYGAGAGVTYPNDPNVTITYPDSVPGYMAPSDVYATARSMGIDKNVNLAYNLTWIVQVDAGFTYLVRLHFCEIQSPIDKPNQRVFNIYLNNQTAVEGADVIQWVDPLSTGTPLYKDYVVGTVGSGIMDFWVALHPDIRNKPQYYDAILNGMEVFKLQLSNGSLAGPNPVPSADPQAHTGQGKKKSLVGPIAGGVIGGLAVLALGYCCFICKRRRKAAKDTGMSDGHSGWLPLSLYGNSHTSSSAKSHATGSIASSLPSNLCRHFSFAEIKAATKNFDESRILGVGGFGKVYQGEIDGGTTKVAIKRGNPLSEQGIHEFQTEIEMLSKLRHRHLVSLIGYCEDKNEMILVYDHMAHGTLREHLYKTQNAPLSWRQRLEICIGAARGLHYLHTGAKHTIIHRDVKTTNILLDEKWVAKVSDFGLSKTGPSMDHTHVSTVVKGSFGYLDPEYFRRQQLTEKSDVYSFGVVLFEVLCARPALNPTLAKEEVSLAEWALHCQKKGILDQIVDPYLKGKIVPQCFKKFAETAEKCVADNGIERPSMGDVLWNLEFALQMQESAEESGSFGCGMSDEGTPLVMAGKKDPNDPSIDSSTTTTTTTSLSMGDQSVASIDSDGLTPSAVFSQIMNPKGR

>Ta-CrRLK1L9-D

MGGGPRCALLLLAAAAACAALVPAAWAQAPTAPAPSGAPFVPRDDILLDCGATGKGNDTDGREWRGDAGSKYAPPNLASADAGAQDPSVPQVPYLTARVSAAAFTYSFPLGPGRKFLRLHFYPANYSNRDAADAFFSVSVPAAKVTLLSNFSAYQTATALNFAYLVREFSVNVTGPTLDLTFTPEKGRPNAYAFINGIEVVSSPDLFDLATPLFVTGDGNNQPFPMDPGAALQTMYRLNVGGQAISPSKDSGGARSWDDDTPYIYGAGAGVTYPNDPNVTITYPDNVPGYVAPSDVYATARSMGIDKNVNLAYNLTWIVQVDAGFTYLVRLHFCEIQSPITKPNQRVFNIYLNNQTAVEGADVIQWVDPLSTGTPLYKDYVVSTVGSGIMDFWVALHPNTGSKPQYYDAILNGMEVFKLQLSNGSLAGPNPVPSADPPAHTGQEKKNSLVGPIAGGVIGGLVVLALGYCCFICKRRRKVAKDAGMSDGHSGWLPLSLYGNSHTSSSAKSHATGSIASSLPSNLCRHFSFAEIKAATKNFDESRILGVGGFGKVYQGEIDGGTTKVAIKRGNPLSEQGIHEFQTEIEMLSKLRHRHLVSLIGYCEDKNEMILVYDHMAHGTLREHLYKTQNAPLSWRQRLEICIGAARGLHYLHTGAKHTIIHRDVKTTNILLDEKWVAKVSDFGLSKTGPSMDHTHVSTVVKGSFGYLDPEYFRRQQLTEKSDVYSFGVVLFEVLCARPALNPTLAKEEVSLAEWALHCQKKGILDQIVDPYLKGKIVPQCFKKFAETAEKCVADNGIERPSMGDVLWNLEFALQMQESAEESGSFGCGISDEEGTPLVMAGKKDPNDPSIDSSTTTTTTTSLSMGDQSVASIDSDGLTPSAVFSQIMNPKGR

>Ta-CrRLK1L10-A

MLRTRILVLAAVSIVFANLQFLKAHGRELFLSCGSNATADADGRRWIGDMAPDLNFTLSSPGIAALLAGSSNGSEIMAPVYRSARFFTTTSWYDFSLLPGNYCVRLHFFPSAFRNFSANGSVFDVVANDFKLVSKFNVSEEIVWRNSVSNSAATAVVKEYFLAVNGSRLQIEFDPRPGSFAFVNAIEVMLTPDNSFNGMVNKVGGVDVHIPPELSGRAVETMYRLNIGGPALASSHDQYLHRPWYTDEAFMFSANAALIVSNTSAIKYVSSNDSSIAPLDVYETARIMGNNMVMDKRFNVTWRFFVHPNFDYLVRLHFCELVYDKPSQRIFKIYINNKTAAENYDVYNKAGGINKAYHEDYFDSLPQQVDSLWLQLGPDSMTSASGTDALLNGLEIFKLSRSGNLDYVLGHIDMGNKRGRSKGRSRIGLWEEVGIGSAAFVALVSVALFSWCYVRRKRKAVNEEVPAGWHPLVLHEAMKSTTDARASKKAPLARNSSSIGHRMGRRFSIADIRAATKNFDESLVIGSGGFGKVYKGEVDDGITVAIKRANPLCGQGLKEFETEIEMLSKLRHRHLVAMIGYCEEQKEMILVYEYMAKGTLRSHLYGSGLPPLTWKQRIDACIGAARGLHYLHTGADRGIIHRDVKTTNILLDKNFVAKIADFGLSKTGPTLDQTHVSTAIRGSFGYLDPEYFRRQQLTQKSDVYSFGVVLFEVACARPVIDPSVPKDQINLAEWAMRWQRQRSLEAIADPRLDGDYSPESLKKFGDIAEKCLADDGRTRPSMGEVLWHLEYVLQLHEAYKRNVDCESFGSSELGFADMSFSMPHIREGEEEHHPKKSGIREDSAP

>Ta-CrRLK1L10-B

MLRMRILVLASVSIVFANLQFLKAHGRELFLSCGSNATADADGRRWIGDMAPDLNFTLSSPGIAALLAGSTNGSEIMAPVYRSARFFTTTSWYDISVLPGNYCVRLHFFPSAFGNFSANGSVFDVVANEFKLVSKFNVSEEIVWRNSVSNSAATAVVKEYFLAVNSSRLQIEFDPRPGSFAFVNAIEVMLTPDNSFNSTVNKVGGVDVHIPPELSGRAIETMYRLNIGGPALASSHDQYLYRPWYTDEAFMFSANAALTVSNTSAIKYVSSGDSSIAPIGVYETARIMGNNMVMDKRFNVTWRFVVHPNFDYMVRLHFCELVYDKPSQRIFKIYINNKTAAENYDVYDKAGGINKAYHEDYFDSLPQQVDSLWLQLGPDSMTSASGTDALLNGLEIFKISRSGNLDYVLGHIDMGNKRGRSKGRSRLGLWEEVGIGSAAFVALASVALFSWCYVRRKRKAVDEEVPAGWHPLVLHEAMKSTTDARASKKAPLARNSSSIGHRMGRRFSIVDIRAATKNFDESLVIGSGGFGKVYKGEVDDGITVAIKRANPLCGQGLKEFETEIEMLSKLRHRHLVAMIGYCEEQKEMILVYEYMAKGTLRSHLYGSGLPPLTWKQRIDACIGAARGLHYLHTGADRGIIHRDVKTTNILLDKNFVAKIADFGLSKTGPTLDQTHVSTAIRGSFGYLDPEYFRRQQLTQKSDVYSFGVVLFEVACARPVIDPSVPKDQINLAEWAMRWQRQRSLEAIADPRLDGDYSPESLKKFGDIAEKCLADDGRTRPSMGEVLWHLEYVLQLHEAYKRNVDCESFGSSELGFADMSFSMPHIREGEEEHHPKKSGIREDSAP

>Ta-CrRLK1L10-D

MLRMRILVLAAVSIVFANLQFLKAHGRELFLSCGSNATADADGRRWIGDMAPGLNFTLSSPGIAALLAGSSNGSEIMAPVYRSARFFTTTSWYDFSLLPGNYCVRLHFFPSTFRNFSANGSVFDVVANDFKLVSKFNVSEEIVWRNSVSNSAATAVVKEYFLAVNSSRLQIEFDPRPGSFAFVNAIEVMLTPDNSFNGTVNKVGGVDAHIPPELSGRAVETMYRLNIGGPALASSHDQYLHRPWYTDEAFMFSANVALIVSNTSAIKYVSSNDSSIAPIDVYETARIMGNNMVMDKRFNVTWRFLVHPNFDYLVRLHFCELVYDKPSQRIFKIYINNKTAAENYDVYNRAGGINKAYHEDYFDSLPQQVDSLWLQLGPDSMTSASGTDALLNGLEIFKLSRSGNLDYVLGHIDMGNKRGRSKGRSRIGLWEEVGIGSAAFVALASVALFSWCYVRRKRKAVNEEVPAGWHPLVLHEAMKSTTDARASKKAPLARNSSSIGHRMGRRFSIADIRAATKNFDESLVIGSGGFGKVYKGEVDDGITVAIKRANPLCGQGLKEFETEIEMLSKLRHRHLVAMIGYCEEQKEMILVYEYMAKGTLRSHLYGSGLPPLTWKQRIDACIGAARGLHYLHTGADRGIIHRDVKTTNILLDKNFVAKIADFGLSKTGPTLDQTHVSTAIRGSFGYLDPEYFRRQQLTQKSDVYSFGVVLFEVACARPVIDPSVPKDQINLAEWAMRWQRQRSLEAIADPRLDGDYSPESLKKFGDIAEKCLADDGRTRPSMGEVLWHLEYVLQLHEAYKRNVDCESFGSSELGFADMSFSMPHIREGEEEHHPKKSGIREDSAP

>Ta-CrRLK1L11-A

MGTTTEQKIALLLLGTIWVLLGTCNAAEFTPADNYLINCGSTVDANLNDGRVFKADNSGSTILTSHHSVPANTLPDAVISSDNPVLYKTARIFIVPSSYSFNMKSRGRHFVRLHFFGFRYQSYDLAAAKFKVSTQHVVLLDNFTPPSNSSLLVREYSLNITEDMLILSFVPLGNSTSFINAIEVISVPDDLIQDSAQTVNPSGQYLGLATQSFQTFYRINVGGREVTVVNDTLSRSWDTDQNFFINSTTTELFAYQGKLNYQKGAATKEDAPDSVYNTARRLAVQNRTSPASNMTWQFDVDGRSSYLIRFHFCDIVSKAAYSLYFDIYVDGGLALENLDLSEKVFGTLAVPYYTEFVLKSSNPSGKLSVGIGPSSLSNVAPDGILNGLEIMKMNISTGTIYVVWPPATPKRKLAIILAPVLGGVGAVSIAIILCFVLRRKKEKKPRRAPTSRPSSSWSPLTLNGLSFLSIGTRTTSRTTHTSGTNSDVSYRIPFALLQVATKHFDEQMVVGVGGFGKVYKAVLQDSTKVAVKRGNQKSHQGLKEFRTEIELLSGLRHRHLVSLIGYCDDQNEMILVYEYMEKGTLKSHLYGSDMPPLSWKKRVEICIGAARGLHYLHTGFAKSIIHRDVKSANILLDENLMAKVSDFGLSKTGPELDQTHVSTAVKGSFGYLDPEYYRRQKLTDKSDVYSFGVVLLEVICARPVIDPTLPRDMINLAEWAIKWQKRGELGQIVDQRIAGTIRPESLRKYGETVEKCLVDYGVDRPTMGDVLWNLEFVLQLQEAGPDISNVDSMNQISELPSDARRMGSLEIGTADEADEGRTHMDYSQMSTNDAFSQLMNTEGR

>Ta-CrRLK1L11-B

MGTTTEQKIALLLLGTIWVLLGTCNAAEFTPADNYLINCGSTVDANLHDGRVFKADNSGLTILTSHHSVPANTLPDAVISSDNPVLYQTSRIFIVPSSYSFKMKSRGRHFVRLHFFSFRYQSYDLAAAKFKVSTQHVVLLDNFTPPSNSSPLVREYSLNITEDMLILSFVPQGNSTSFISAIEVISVPDDLIQDSAQTVNPSGQYLGLATQSFQTFYRINVGGREVTVVNDTLSRSWDTDQNFFLNSTTTELFAYQGKLNYQKGAATKEDAPDSVYNTARRLAVQNRTSPASNMTWQFDVDGRSSYLIRFHFCDIVSKAAYSLYFDIYVDGGLALENLDLSEKVFGTLAVPYYTEFVLKSSNPSGKLSVGVGPSSLNNVAPDGILNGLEIMKMNISTGTIYVVWPPAPPKRKLAIILGSVLGGVGAVSIAIILCFVLRRKKKEKKPRRAPTSRPSSSWSPLTLNGLSFLTVGTRTTSRTTHTSGTNSDVSYRIPFALLQVATKHFDEQMVVGVGGFGKVYKAVLQDSTKVAVKRGNQKSHQGLKEFRTEIELLSGLRHRHLVSLIGYCDEQNEMILVYEYMEKGTLKSHLYGSDMPPLSWKKRVEICIGAARGLHYLHTGFAKSIIHRDVKSANILLDENLMAKVSDFGLSKTGPELDQTHVSTAVKGSFGYLDPEYYRRQKLTDKSDVYSFGVVLLEVICARPVIDPTLPRDMINLAEWAIKWQKRGELGQIVDQRIAGTIRPESLRKYGETVEKCLANYGVDRPTMGDVLWNLEFVLQLQEAGPDISNVDSMNQISELPSDARRMGSLEIRTADEADESRTNMDYSQMSTNDAFSQLINTEGR

>Ta-CrRLK1L11-D

MGTTTGQKTALLLLGTLWVLLGTCNAAEFSPADNYLINCGSTVDANLHDGRVFKADNSGSTILTSHHSVPANTLPDAVISSDNPVLYQTARIFIVPSSYSFNMKSRGRHFVRLHFFGFRYQSYDLAAAKFKVSTQHVVLLDNFTPPSNSSPLDSAQTVNPSGQYLGLATQSFQTFYRINVGGREVTVVNDTLSRSWDTDQNFFINSTTTELFAYQGRLNYQKGAATKEDAPDSVYNTARRLAVQNRTSPASNMTWQFDVDGRSSYLIRFHFCDIVSKAAYSLYFDIYVDGGLALENLDLSEKVFGTLAVPYYTEFVLKSSNPSGKLSVGIGPSSLNNVALDGILNGLEIMKMNISTGTIYVVWPPATPKRKLAIILGPVLGGVGAVSIAIILCFVLRRKKKEKKPRRAPTSRPSSSWSPLTLNGLSFLSIGTRTTSRTTHTSGTNSDVSYRIPFALLQVATKHFDEQMVVGVGGFGKVYKAVLQDSTKVAVKRGNQKSHQGLKEFRTEIELLSGLRHRHLVSLIGYCDEQNEMILVYEYMEKGTLKSHLYGSDMPPLSWKKRVEICIGAARGLHYLHTGFAKSIIHRDVKSANILLDENLMAKVSDFGLSKTGPELDQTHVSTAVKGSFGYLDPEYYRRQKLTDKSDVYSFGVVLLEVICARPVIDPTLPRDMINLAEWAIKWQKRGELGQIVDQRIAGTIRPESLRKYGETVEKCLADYGVDRPTMGDVLWNLEFVLQLQEAGPDISNVDSMNQISELPSDARRMGSLEIGTADEGRTNMDYSQMSTNDAFSQLMNTEGR

>Ta-CrRLK1L12-A

MAAARGRGRGRGRCVLLAAVLLLTAVVGADIYKPTDSILVHCGSDKDGQDEDGRKWTTDKDSKWLPDGGKSSIMGTADVADPSLPSPVPYMTARVFPKETAYTFPVADADRHWVRLHFYPAAYHGIPADHFFFSVTTSTGVTLLRNFSVYITAKALTQAYIIREFSLPPSTVGSLSLKFTPTAMNNASYAFVNGIEVISMPSFFGDPATLVGLNDQSLDASAANLQTMYRLSVGGSYIPPANDSGLSREWFSDTPYVYGAATGVTFEANDTVPIKYPAPADEYAAPVSIYDSFRHMGRDPKMNKNNNLTWVFEVDGNFTYLLRLHFCSLMEDKINQVVFAILLNNKTATTTGSADIIAWAKEKNPANPGAPGKGVPVFKDYAVFMPAAPAGNDTILWLTLRPDTATRTQFVNAFLNGLEVFKVSDASGNLAGPNPDISKMLAEAELGAVEGQFREKPSNVGALIGGAAGGAAAFGLVAAVCFVAYQSKRRRELSSSPSHSSSGWLPVYGGSTSVSKSSGGRSAATLNPNITAMCRHFSLQEIKSATKGFDESLVIGVGGFGKVYRGVVDGDTKVAVKRSNPSSEQGVLEFQTEIEMLSKLRHKHLVSLIGCCEDNGEMILVYDYMAHGTLREHLYNKSGKPPLPWRQRLEIVIGAARGLHYLHTGAKYTIIHRDVKTTNILVDDKWVAKVSDFGLSKTGPTVQNQTHVSTMVKGSFGYLDPEYFRRQKLTEKSDVYSFGVVLFEVLCGRPALNPSLPREQVSLADHALSCQRKGTLEEIVDPVLEGKIAPDCLKKFAETAEKCLADQGVDRPSMGDVLWNLEFALQMQDTFDNGGKPPEVDDYSSSFTIAQPSMEESLAANAAALSLISEDMDEEDIANSVIFSQIAKPTGR

>Ta-CrRLK1L12-B

MAAARGRGVLLAVLLLTTVAFAFVGADIYKPTDSILVNCGSDKDGQDEDGRKWTTDKDSKWLPDGGKSSIMGTADVSDPSLPSPVPYMTARVFPKETAYTFPVSDADRHWVRLHFYPAAYHDIPADHFFFSISTSTGITLLRNFSVYITAKALTQAYIVREFSLPPSTAGSLSLKFTPTAMNNASYAFVNGIEIISMPNFFGDPATLVGLDDQSLDASAGNLQTMYRLSVGGSYIPPTNDSGLTREWFSDTPYVYGAGTGVTFEANDTIPIKYPAPADEYAAPVSIYDTFRHMGRDANLNKNNNLTWVFEVDGNFTYLLRLHFCSLMEDKINQVVFAILVNNKTATTTGSADIIAWAKEKNPANPGAPGKGVPVFKDYAVFMPAAPAGNDTILWLTLRPDTASNPQFVNAFLNGLEIFKVSDASGNLAGPNPDISKMLAEAELGAVDGQFREKPSNVGALIGGAVGGAAAFGLVAAVCFVAYQSKRGRELSSSPSHSSSRWLPVYGSSQTSVSKSSGGRSAMTLNPNITAMCRHFSFQEIKSATKGFDESLVIGVGGFGKVYRGVVDGDTKVAIKRSNPSSEQGVLEFQTEIEMLSKLRHKHLVSLIGCCEDNGEMILVYDYMAHGTLREHLYKSGKPPLPWRQRLEIVIGAARGLHYLHTGAKYTIIHRDVKTTNILVDEKWVAKVSDFGLSKTGPTVQNQTHVSTMVKGSFGYLDPEYFRRQKLTEKSDVYSFGVVLFEVLCGRPALNPSLPREQVSLADHALSCQRKGTLEEIIDPVLEGKIAPDCLKKFAETAEKCLADQGVDRPSMGDVLWNLEFALQQQDTFENGGKPPEVDDYSSSFTITPPSMEESLAANAAALSLISEDMDEEDIANSVIFSQIAKPTGR

>Ta-CrRLK1L12-D

MAAARGRGVLLAVLLLMMVAFAFVGADIYKPTDSILVHCGSDKDGQDEDGRKWTADKDSKWLPDGGKSSVMGTADVPDPSLPSPVPYMTARVFPKETAYTFPVADADRHWVRLHFYPAAYHGIPADHFFFSVTTSTGVTLLRNFSVYTTAKALTQAYIVREFSLPPSTTGSLSLKFTPTAMNNASYAFVNGIEIISMPSFFGDPATLVGLDDQSLDASAGNLQTMYRLSVGGSYIPPANDSGLSREWFSDTPYVYGAATGVTFEANDTIPIKYPTPADEYAAPVSIYDSFRHMGRDPKMNRNNNLTWVFEVDGNFTYLLRLHFCSLMEDKINQVVFAILVNNKTATTTGSADIIAWAKEKNPANPGAPGKGVPVFKDYAVFMPAAPAGSDTILWLTLRPDTATNPQFVNAFLNGLEVFKVSDASGNLAGPNPDISKMLAEAELGAVDGQFREKPSNVGALIGGAAGGAAAFGLVAAVCFVAYQSKRRRELSSSPSHSSSGWLPVYGGNSQTSVSKSSGGRSAVTLNPNITAMCRHFSFQEIKSATKGFDESLVIGVGGFGKVYRGVVDGDTKVAIKRSNPSSEQGVLEFQTEIEMLSKLRHKHLVSLIGCCEDNGEMILVYDYMAHGTLREHLYKSGKPPLPWRQRLEIVIGAARGLHYLHTGAKYTIIHRDVKTTNILVDEKWVAKVSDFGLSKTGPTVQNQTHVSTMVKGSFGYLDPEYFRRQKLTEKSDVYSFGVVLFEVLCGRPALNPSLPREQVSLADHALSCQRKGTLEEIIDPVLEGKIAPDCLKKFAETAEKCLADQGVDRPSMGDVLWNLEFALQQQDTFENGGKPPEVDDYSSSFTITPPSMEESLAANAAALSLISEDMDEEDIANSVIFSQIAKPTGR

>Ta-CrRLK1L13-A

MVRRGALPLALLAVLATLTAVAGQGKPVTDNGSGGGSGPSKFTPKDAFYIDCGGTAAADTKDGKSFKTDAEANSLLSARDNIKVADDKADVPSHLYRSARVFKEEAVYNFPLTAPGWHFIRLYFFPIKSGEADLAAATFDVSTAVNVLLHGFTPEAKAVMKEYIVNATENKLELKFTPQSGSAFINAIEVVNAPDELISKTALTVSPLAETSGLSEAAYQVVCRLNVGGPPIGPVNDTLGRQWEDDGQYLNPKDAGTEVSVPTSAIKYPDAFPATKLVAPTAVYATARHMAESGVANQNFNVSWKVDVDPSFDYLVRLFFADIISTSANDLYFNAYINGRKAISALDLSTITGDLAAPYYKDFVVNSSVNTDGHIIIGVGPLGQDTGRNDALLNGAEVLKMSNSVGSLDGEFGVDGRMVDDGSGTRKVVAAVGFAMMFGAFAGLGCMVVKWHRRPQDWDRRNSFSSWLLPIHTGQSFSNGKGSKSGYTFSSTAGLGHFFTFAEMSEATKNFDESAIIGVGGFGNVYVGEINDPDEEGSRIKVAIKRGNPSSEQGINEFNTEIQMLSKLRHRHLVSLIGYCDEGEEMILVYEFMQHGPFRDHIYGGPEGLPTLSWKQRLEICIGAARGLHYLHTGTAHGIIHRDVKTTNILLDEKFVAKVADFGLSKDGPGMNQLHVSTAVKGSFGYLDPEYFRCQQLTDKSDVYSFGVVLLETLCARAPIDPQLPREQVSLAEWGLQWKRKGLIEKIMDPNLNGKVNPESLAKFAETAEKCLCEFGSDRLSMGDVLWNLEYALQLQEANPPEGATDADDADASIVSSASGVTTVPDQSTTSANELFAQLADMKGR

>Ta-CrRLK1L13-B

MVRRGTFPLALLAVLATLTAVAGQGKPVTDNGSGGASGPAKFTPKDAFYIDCGGTAAADTKDGKSFKTDAEANSLLSARDNIKVADDKADVPSHLYRSARVFKEEAVYNFPLTAPGWHFIRLYFFPIKSGEADLAAATFDVTTAVNVLLHGFTAEAKAVMKEYVVNATENKLELKFTPQSGAAFINAIEVVNAPDELISKTALTVSPLAETSGLSEAAYQVVCRLNVGGPPIGPVNDTLGRQWEDDGQYLNPKEAGAEVSVPTSAIKYPDAFPATKLVAPTAVYATARHMAESGVANQNFNVSWKVDVDPSFDYLVRLFFADIISTSANDLYFNVYINGRKAISALDLSTITGDLAAPYYKDFVVNSSVNTDGHIIIDVGPLGQDTGRNDALLNGAEVLKMTNSVGSLDGEYGVDGRMVDDGSGTRKVVAAVGFAMMFGAFAGLGCMVVKWHRRPQDWERRNSFSSWLLPIHTGQSFSNGKSKSGYTFSSTAGLGHFFTFAEMSEATKNFAESAIIGVGGFGNVYVGEINDPDEEGSRIKVAIKRGNPSSEQGINEFNTEIQMLSKLRHRHLVSLIGYCDEGEEMILVYEFMQHGPFRDHIYGGPEGLPTLSWKQRLEICIGAARGLHYLHTGTAHGIIHRDVKTTNILLDDKFVAKVADFGLSKDGPGMNQLHVSTAVKGSFGYLDPEYFRCQQLTDKSDVYSFGVVLLETLCARAPIDPQLPREQVSLAEWGLQWKRKGLIEKIMDPNLAGKVNPESLAKFAETAEKCLCEFGSDRLSMGDVLWNLEYALQLQESNPPEGASDADDADASIVSSASGVTTVPDQSTTSANELFAQLADMKGR

>Ta-CrRLK1L13-D

MVRRGALPLALLAVLATLTAVAGQGKPVTDNGSGGGAGPAKFTPKDAFYIDCGGTAAADTKDGKSFKTDAEANSLLSARDNIKVADDKADVPSHLYRSARVFKEEAVYNFPLTAPGWHFIRLYFFPIKSGEADLAAATFDVSTAVNVLLHGFTPEAKAVMKEYIVNATENKLELKFTPQSGSAFINAIEVVNAPDELISKTALTVSPLAETSGLSEAAYQVVCRLNVGGPPIGPVNDTLGRQWEDDEKYLNPKEAGTEVSVPTSAIKYPDAFPATKLVAPTAVYATARHMAESGVANQNFNVSWKVDVDPSFDYLVRLLFADIISTSANDLYFNVYINGRKAISALDLSTITGDLAAPYYKDFVVNSSVNTDGHIIIDVGPLGQDTGRNDALLNGAEVLKMSNSVGSLDGEYGVDGRMVDDGSGTRKVVAAVGFAMMFGAFAGLGCMVVKWHRRPQDWERRNSFSSWLLPIHTGQSFSNGKSKSGYTFSSTAGLGHFFTFAEMSEATKNFDESAIIGVGGFGNVYVGEINDPDEEGSRIKVAIKRGNPSSEQGINEFNTEIQMLSKLRHRHLVSLIGYCDEGEEMILVYEFMQHGPFRDHIYGGPEGLPTLSWKQRLEICIGAARGLHYLHTGTAHGIIHRDVKTTNILLDDKFVAKVADFGLSKDGPGMNQLHVSTAVKGSFGYLDPEYFRCQQLTDKSDVYSFGVVLLETLCARAPIDPQLPREQVSLAEWGLQWKRKGLIEKIMDPNLNGKVNPESLAKFAETAEKCLCEFGSDRLSMGDVLWNLEYALQLQEANPPEGATDADDADASIVSSASGVTTVPDQSTTSANELFAQLADMKGR

>Ta-CrRLK1L14-A

MPAAGRSGGPGQVNIMMGRRKLQVVTLAILCFWSSAGICKAQSVDFKPADSYLVDCGSAKGTTVLGRDFAADGAAPVTVATSQDILAGTSANGVSSFDNPVLYQTARIFTSPSSYTFPIQKQGRHFVRLYFYPFIYQSYDLSTAKFTVSTQDVLLLSDFQQPDKTAPLFKEYSLNITRDQLVISFKPSNGIAFINAIEVISVPDDLIADVANMVNPVQQYSGLTTQSLETVYRVNMGGPKVFPNNDTLSRTWQKDQKYILNPSVTKTAQYGKAINYRKGGATPLTAPDIVYSTATELAASNTSNALFNMTWQFDVDAGFSYLIRFHFCDIVSKALNQLYFNAYVGGFFAQHDLDLSEQSVNQLATAIYVDVVLSSNDASSKLSISIGPSTLNNALPDGILNGLEIMKMGSGSGSAFTVGNNGSNKKLPIIIGSVLGVVGLLIIVLVVVLLCRRKKTDDKQHSKTWMPFSINGLTSLSTGSRTSYGTTLTSGLNGSYGYRFAFNVLQEATNNFDESWVIGVGGFGKVYKGALRDDTKVAVKRGNPKSQQGLNEFRTEIELLSRLRHRHLVSLIGYCDERNEMILVYEYMENGTVKSHLYGSDNPSLNWKQRLEICIGAARGLHYLHTGSAKAIIHRDVKSANILLDENLLAKVADFGLSKTGPELDQTHVSTAVKGSFGYLDPEYFRRQQLTEKSDVYSFGVVMLEVLCARPVIDPSLPREMVNLAEWGMKWQKRGELHQIVDQKLSGAIRPDSLRKFGETVEKCLADYGVERPSMGDVLWNLEYVLQLQDVDSSTVSDVNSMNRIVDLSSQVQHVSAMESISVTMAEDGALHEPDHDLSDVSMSRVFSQLIKAEGR

>Ta-CrRLK1L14-B

MPAAARSGGPGQANIMMGRRKLQAVTLAILCFWSSAGAQTVDFKPADNYLVDCGSAKGTTVLGRDFAADGASPVTVSTSQDILAGTSANGVSSFDNPLLYQTARIFTSPSSYTFPIQKQGRHFVRLYFFPFIYQSYDLSTAKFTVSTQDVLLLSDFQQPDKTAPLFKEYSLNITRDQLVISFKPSNGIAFINAIEVVSVPDDLIADVANMVNPVQQYSGLTTQSLETVYRVNMGGPKVFPSNDTLSRTWQKDQKYILNPSVTKTAQYGKPIKYRKGGATPLTAPDIVYSTATELAAANTSNALFNMTWQFDVDAGFSYLIRFHFCDIVSKALNQLYFNAYVGGFFAQHDLDLSEQSVNQLATAIYVDVVLSSNDASSKLSISIGPSTLNNALPDGILNGLEIMKMGSGSGSAFTVGNNGSNKRLPIIIGSVLGVVGLLIIVLVVVLLCRRKKTDDKQHSKTWMPFSINGLTSLSTGSRTSYGTTLTSGLNGSYGYRFAFNVLQEATNNFDESWVIGVGGFGKVYKGALRDDTKVAVKRGNPKSQQGLNEFRTEIELLSRLRHRHLVSLIGYCDERNEMILVYEYMENGTVKSHLYGSDNPSLNWKQRLEICIGAARGLHYLHTGSAKAIIHRDVKSANILLDENLLAKVADFGLSKTGPELDQTHVSTAVKGSFGYLDPEYFRRQQLTEKSDVYSFGVVMLEVLCARPVIDPSLPREMVNLAEWGMKWQKRGELHQIVDQKLSGAIRPDSLRKFGETVEKCLADYGVERPSMGDVLWNLEYVLQLQDVDSSTVSDVNSMNRIVDLSSQVQHVGAMESISVTMAEDGALHEPDHDLSDVSMSRVFSQLIKAEGR

>Ta-CrRLK1L14-D

MMGRRKLQVVTLAILCFWSSAGVCKAQTVDFKPADSYLVDCGSTKGTTVLGRDFAADGASPVTVSTSQDILAGTSANGVSSFDNPVLYQTARVFTSPSSYTFPIQKQGRHFVRLYFYPFIYQSYDLSTAKFTVSTQDVLLLSDFQQPDKTAPLFKEYSLNITRDQLVISFKPSNGIAFINAIEVVSVPDDLIADVANMVNPVQQYSGLTTQSLETVYRVNMGGPKVFPNNDTLSRTWQKDQKYILNPSVTKTAVYGKAIKYRKGGATPLTAPDIVYSTATELAASNTSNALFNMTWQFDVDAGFSYLIRFHFCDIVSKALNQLYFNAYVGGFFAQHDLDLSEQSVNQLATAIYVDVVLSSNDASSKLSISIGPSTLNNALPDGILNGLEIMKMGSGSGSAFTVGNNGSNKKLPIIIGSVLGVVGLLIIVLVVVLLCRRKKTDDKQHSKTWMPFSINGLTSLSTGSRTSYGTTLTSGLNGSYGYRFAFNVLQEATNNFDESWVIGVGGFGKVYKGALRDDTKVAVKRGNPKSQQGLNEFRTEIELLSRLRHRHLVSLIGYCDERNEMILVYEYMENGTVKSHLYGSDNPSLNWKQRLEICIGAARGLHYLHTGSAKAIIHRDVKSANILLDENLLAKVADFGLSKTGPELDQTHVSTAVKGSFGYLDPEYFRRQQLTEKSDVYSFGVVMLEVLCARPVIDPSLPREMVNLAEWGMKWQKRGELHQIVDQKLSGAIRPDSLRKFGETVEKCLADYGVERPSMGDVLWNLEYVLQLQDVDSSTVSDVNSMNRIVDLSSQVQHVGAMESISVTMAEDGALHEPDHDLSDVSMSRVFSQLIKAEGR

>Ta-CrRLK1L15-A

MNSSANFLSILVLLVFLAAGNARAQPQPILINCASDSTTSVDARTWIGDSSPSNNFTLSFPGAIASAAPAPAPAPGVDGEQDPYGDLYKTARVFNASSSYRLAVAPGSYFLRLHFSQQFANLGAQEPIFSVAANGLRLLSKFSVHGEISWRDSQINSTSSVIVKEYLLNVTSGKLGIEFTPDEGSFAFINAMEVLPVSGTSIFDSVNKVDAHGLKGPFSLDGGGIETMYRLCVGCRDVLTRKEDPGLWRRWDKDDHFIFSLNAANSIFNSSNISYVSADDPTVAPLRLYQSARVPTESSVLGKKFNVSWSFNIDPGFDYLVRLHFCELQYDKAEQRKFKIYINNKTAAEGYDVLARAGGKNKAFYEDFLDAASPQMDTLWVQLGSESSAGSAAADALLNGMEIFKVSREGNLAHPTVRIGGFSGGTSKPKRSPKWVLIGAASGLIIFIAIAAALYLCFNLRRKKNSSASKAKDNPHGAAHTRSPTLLTAGAFGSKRMGRRFTIAEIRTATVNFDESLVIGVGGFGKVYRGIMEDGTRVAIKRGYTDSHQGQGVKEFETEIEMLSRLRHRHLVPLIGYCDEQNEMVLVYEHMANGTLRSHLYGSDLPALTWKQRLEICIGAARGLHYLHTGLDRGIIHRDVKTTNILLDNNLVAKMADFGISKDGPALDHTHVSTAVKGSFGYLDPEYYRRQQLTPSSDVYSFGVVLFEVLCARPVINPTLPRDQINLADWALNRQRHKLLETIIDLRLDGNYTLESIRTFSEIAEKCLADEGVNRPSMGEVLWHLESALQLEQGHLQSTNGDGCSDPQLKPSDVPTHVACIKEVEQSTRPGSHDSDGQVVDVKIEVP

>Ta-CrRLK1L15-B

MKSSANFLSILVLLVFLAAGNARAQPQPILINCGSDSTTSVDARTWIGDSSPSNNFTLSFPGAIASAAPAPGVDGEQDPYGDLYKTARVFNASSSYRLAVAPGSYFLRLHFSQQFANLGAQEPIFNVAANGLRLLSKFSVHGEISWRDSQINSTSSVIVKEYLLNVTSGKLGIEFTPDEGSFAFINAMEVLPVSGTSIFDSVNKVDGHGLKGPFSLDGSGIETMYRLCVGCIDVLARKEDPGLWRRWDKDEHFIFSLNAASSIFNSSNISYVSADDPTVAPLRLYQSARVPTESSVLGKKFNVSWSFNIDPGFDYLVRLHFCELQYDKAEQRKFKIYINNKTAAEGYDVFARAGGKNKAFYEDFLDAASPQMDTLWVQLGSESSAGSAAADALLNGMEIFKVSREGNLAHPTVRIGGISGGARKPKRSPKWVLIGAASGLIIFIAIAGALYFCFNLQRKKNSSANKAKDNLHGVTHTRSPTLRTAGAFGSKRMGRRFTIAEIRTATVNFDESLVIGVGGFGKVYRGIMEDGTRVAIKRGYTDSHQGQGVKEFETEIEMLSRLRHRHLVPLIGYCDEQNEMVLVYEHMANGTLRSHLYGSDLPALTWKQRLEICIGAARGLHYLHTGLDRGIIHRDVKTTNILLDDNLVAKMADFGISKDGPALDHTHVSTAVKGSFGYLDPEYYRRQQLTPSSDVYSFGVVLFEVLCARPVINPTLPRDQINLADWALNRQRHRLLETIIDLRLDGNYTLESIKIFSEIAEKCLADEGVNRPSMGEVLWHLESALQLEQGHPQSTNGDGCSDPQLKPSDVPTRVACIKEVEQSTRPGSHDSDGQVVDVKIEVP

>Ta-CrRLK1L15-D

MKSSANFLSILVLLVFLAAENARAQPQPILINCGSDSTTSVDARTWIGDSSPSNNFTLSFPGAIASAAPAPAPGVDGEQDPYGDLYKTARVFNASSSYRLAVAPGSYFLRLHFSQQFANLGAQEPIFSVAANGLRLLSKFSVHGEISWRDSQINSTSSVIVKEYLLNVTSGKLGIEFTPDEGSFAFINAMEVLPVSGTSIFDSVNKVDAHGLKGPFSLDGDGIETMYRLCVGCIDVLPRKEDPGLWRRWDKDEHFIFSLNAANSIFNSSNISYVSADDPTVAPLRLYQSARVPTESSVLGKKFNVSWSFNIDPGFDYLVRLHFCELQYDKAEQRKFKIYINNKTAAESYDVFARAGGKNKAFYEDFLDAASPQMDTLWVQLGAESSAGSAAADALLNGMEIFKVSREGNLAHPTVRIGGISGGASKPKRSPKWVLIGTASGLIIFIAIAGGLYFGFNLRRKKNSSASKAKDNLHGATHTRSPTLRTAGAFGSNRMGRRFTIAEIRTATVNFDESLVIGVGGFGKVYKGIMEDGTRVAIKRGHTDSHQGQGVKEFETEIEMLSRLRHRHLVPLIGYCDEQNEMVLVYEHMANGTLRSHLYGSDLPALTWKQRLEICIGAARGLHYLHTGLDRGIIHRDVKTTNILLDDNLVAKMADFGISKDGPALDHTHVSTAVKGSFGYLDPEYYRRQQLTPSSDVYSFGVVLFEVLCARPVINPTLPRDQINLADWALNRQRHRLLETIIDLRLDGNYTLASVKKSSKIAEKCLADEGVNRPSMGEVLWHLESALQLEQGHPQSTNADGCSDPQLKPSDVPTRVACIKEDEQSTRPGSHNSDGQVVDVKIEVP

**Supplementary Table S2B. Protein sequences for other species used in phylogenetic analysis.** *B. distachyon* sequences (Bd) were collected from the Ensembl Plants database, and sequences for *Ae. tauschii* (Aet) and *H. vulgare* (Hv) were collected from the NR database at NCBI, Arabidopsis sequences (At) were retrieved from the TAIR database, and rice sequences (Os) were collected from the Rice Genome Annotation Project database (http://rice.plantbiology.msu.edu/) (Nguyen et al., 2014). Sequences and their IDs from the respective databases are given in the brackets.

>Aet-CrRLK1L1(XP_020194102.1)

MPALAILARSMAECKRVPMFLILFILSITSVATTNAIASKVDRFVPQDNYLLSCGASAAVQVDDGRTFRSDPESVSFLSTPTDIKIAAKASLASASPLSPLYLDARVFSDISTYSFFISQPGRHWIRLYFLPITDSQYNLTTATFSVSTDSMVLLHDFSFIASPPNPVFREYLVSAQGDNLKIIFTPKKNSIAFINAIEVVSAPPSLIPNTTTRMGPQDQFDISNSALQVVYRLNMGGALVTSFNDTLGRTWQPDAPFLKLEAAAEAAWVPPRTIKYPDDKTLTPLIAPASIYSTAQQMASTNITNARFNITWQMAAEPGFRYLIRLHFSDIVSKTLNSLYFNVYINGMMAVANLDLSSLTMGLAVAYYKDLIAESSSIINSTLVVQVGPNTIDSGDPNAILNGLEIMKISNEANSLDGLFSPKTSSEVSKTTLTGIAFALAATAALAVVICYRRNRKPAWQRTNSFHSWFLPLNSSSSFMSSCSRLSRNRFGSTRTKSGFSSVFASSAYGLGRYFTFVEIQKATKNFEEKGVIGVGGFGKVYLGATEDGTQLAIKRGNPSSDQGMNEFLTEIQMLSKLRHRHLVSLIGCCDENNEMILVYEFMSNGPLRDHLYGDTNIKPISWKQRLEVCIGAAKGLHYLHTGSAQGIIHRDVKTTNILLDENFVAKVADFGLSKDAPSLEQTHVSTAVKGSFGYLDPEYFRRQQLTDKSDVYSFGVVLFEVLCARPAINPALPRDQVNLGEWARTWHRKGELGKIIDPNIAGQIRPDSLEMFAEAAEKCLADYGVDRPTMGDVLWKLEFALQLQEKGDVVDGASDGIAMKSLEVTNVDSMEKSGNAIPSYVQGR

>Aet-CrRLK1L2(XP_020194829.1)

MAFPALPVTLTCLILLSLLSLAMAADNNSTGLILVNCGASVQGDDDSGRTWDGDTGSKFAPSLKGVAATAPNQDPSLPSTVPFMTARIFTSNYTYSFSVKPGRMFLRLYFYPVAYPNYAVSDAFFSVTTPKLVLLNDFSASQTAQAITSAFLVREFSVNVSSGSSLDLTFAPSAHRNGSYAFVNGIEIVPTPDIFTAPDTRNVGDNTAPFSFDTSSSLQTMYRLNVGGQAISPKGDLGGFYRSWANDAPYIAGGSGVTFSKDDNLTITYTSKVPKYTAPPDVYGTARSMGPTAQINLNYNLTWILPVDAGFFYLLRFHFCEIQYPIIKINQRSFFIYINNQTAQEQMDVIVWSGGIGRTTYTDYVIMAAGFGQVDMWIALHPDLSSRPEYFDAILNGLEVFKLQNYGSPNNLSGLNPPLPQKPADASPSAASGKMKSVAAIIGGAVGGFIVLLAACFGVCIICKRKNKKKKKTSKDPGGKSEDGHWTPLTEYSGSRSAMSGNTATTGSTLPSNLCRHFTFADLQTATKNFDQAFLLGKGGFGNVYLGEIDSGTKVAIKRCNPMSEQGVHEFQTEIEMLSKLRHRHLVSLIGYCEDKSEMILVYDYMAHGTLREHLYNTKNPPLSWKQRLEICIGAARGLYYLHTGVKHTIIHRDVKTTNILLDDKWVAKVSDFGLSKTGPNMDATHVSTVVKGSFGYLDPEYFRRQQLSEKSDVYSFGVVLFEVLCARPALSPTLPKEQISLADWALRCQKQGVLGQVIDPVLQGKIAPQCFLKFTDTAEKCVADRSVDRPSMGDVLWNLEFALQLQESEEDTGSLTEGTLSSSGASPLVMTRLQSDEPSTDASTTTTSTTTMSMTGRSIASMDSDGLTPSAVFSQIMHPDGR

>Aet-CrRLK1L3(XP_020197218.1)

MAATARLRPARARGVLWVVSVLLVCGAAAYKPEDNYLVSCGSSLDTPVGRRLFLADDGASGAVTLTSPRSAAVKAPPDLVSGFRDAALYQNARVFSAPSSYSFAIRHRGRHFLRLHFFPFVYRSYDLAAAARAFKVSTQDAVLLEDGIPAPEPGNASTSTSPQPARLEFLLDVARDTLVVSFVPLADGGIAFVNAVELVSVPDGLVADAADSSTGRPEPIPAVLPLQTAYRLNVGGPAVAPDDDALWREWTTDLRFLSHSVADAVTREVRYNGMLNRLPGQATATDAPDIVYATARELVINGSSFDGQKQMAWQFDVDTSSSYFIRFHFCDIVGKAPHQLHINAYVDDATVKQDLDLAAVGDGALAFPYYTDFVLPASEASGKLAVHVGPLANKIVMPAAILNGIEIMKMHLSAGSVVVVQPAAGAAKSRFAVVLGSVCGALAFISVAVALAIVLRKKEKEKEVEEGAKEQPTPTQSQSSTPWMPLLGRFSVRGAIASGSSSFTTAGNTPGASPRAAAAAAVMPSYRFPLAMLQDATRNFDDSLIIGEGGFGKVYGAVLQDGTKVAVKRASPESQQGAREFRTEIELLSGLRHRHLVSLVGYCDEREEMILLYEYMEHGSLRSRLYGRGGAAPLSWAQRLEACAGAARGLLYLHTAVDKPVIHRDVKSSNILLDGDLAGKVADFGLSKAGPVLDETHVSTAVKGSFGYVDPEYCRTRQLTAKSDVYSLGVVLLEAVCARPVVDPRLPKPMSNLVEWGLHWQGRGELEKIVDRRIAAAARPAALRKYGETVARCLAERGADRPAMEDVVWNLQFVMRLQEGDGLDFSDVSSLNMVTELTPPRRQRSAVDHDGLDYSDVNSLNMVTELTPPQTGSVEGDGEADDDFTDASMRGTFWQMVNVRSR

>Aet-CrRLK1L4(XP_020147554.1)

MRGGPRCALLLLVAAAALVPAARAQGATAPAPSSGVPFVPRDDILLDCGATGKGNDTDGRQWDGDAGSKYAPPKLASASAGAQDPSVPQVPYLTARVSAAPFTYSFPLGPGRKFLRLHFYPANYSNRNAADAFFSVSVPAAKVTLLSNFSAYQTTTALNFAYIVREFSVNVTGQNLDLTFTPEKGHPNAYAFINGIEVVSSPDLFDLATPQLVTGDGNSQPYEMDPAAALQTMYRLNVGGQAISPSKDSGGARSWDDDTPYIYGAGAGVSYQNDPSVAITYPDNVPGYVAPSDVYATARSMGPDKGVNMAYNLTWILQVDAGYQYLVRLHFCEIQSPYTKPNQRVFNIYLNNQTAMQGADVIQWADPNGIGTPVYKDYVVSTVGSGIMDFWVALHPDAETKPQYYDAILNGMEVFKLQLTNGSLVGLNPVPSADPPAHSGSGDKKSLVAPIVGGVIGGLAVLALGYCCFICKRRRKAAKASGMSDGHSGWLPLSLYGHSHTSSSAKSHATGSYASSLPSNLCRHFSFAEIKAATKNFDESRILGVGGFGKVYHGEIDGGTTKVAIKRGNPLSEQGIHEFQTEIEMLSKLRHRHLVSLIGYCEEKNEMILVYDYMAHGTLREHLYKTQNAPLSWRQRLEICIGAARGLHYLHTGAKHTIIHRDVKTTNILLDDKWVAKVSDFGLSKTGPSMDHTHVSTVVKGSFGYLDPEYFRRQQLTEKSDVYSFGVVLFEVLCARPALNPTLAKEEVSLAEWALHCQKKGILDQIVDPYLKGKIVPQCFKKFAETAEKCVADNGIERPSMGDVLWNLEFALQMQESAEESGSIGCGMSDEGTPLVMVGKKDPNDPSIDSSTTTTTTTSLSMGDQSVASIDSDGLTPSAVFSQIMNPKGR

>Aet-CrRLK1L5(XP_020150974.1)

MAVHVVPPLLLLLLATALPYTALAVFSPDFSFFLACGAGADVPFPSDNPTRTFVRDDGYLSQGRPAAVSASASSGAASNPLYAAARADSSAFSYRLAYPATAGASSFLVLRLHFFPFVPASSSTSLSSARFTVSVLDAYALLPTFSPPVAGVVKEFFVPRDGSKDFTIRFTPDAGSSAFVNAVELFSAPPELLWNNTAVPVDPVGSNDLPEWPLDALETVYRLNVGGPMVTKENDTLWRTWLPDGPYLFGAPGQSVVNSTSSPIMYDPSNGYTQDVAPDVVYRTQRAANVTDLLVATTPGLNFNVTWTFPAEQGSRYLVRLHFCDYEVVSSVVGVGIVFNVYVAQAIGTPALSPKDRARQSNEAFYMDYAARAPRAGNLTVSIGWLRQSSGGGILNGLEIMKLQSADPSLTVSHGLTKRSIIIIVLATVLGAAVLACAVLCFFVVRRTKRRQVAPPASTEDKESTQLPWSPYTQEGISGWADESTNRSSEGTTARMQRVSTKLHISLAELKAATDNFHDRNLIGVGGFGNVYKGALADGTPVAVKRAMRASKQGLPEFHTEIVVLSGIRHRHLVSLIGYCNEQAEMILVYEYMEKGTLRSHLYGGSDDEPPLSWKQRLEICIGAARGLHYLHCGYSENIIHRDVKSTNILLGTDDGGSTGGGAIIAKVADFGLSRIGPSLGETHVSTAVKGSFGYLDPEYFKTQQLTDRSDVYSFGVVLFEVLCARPVIDQSLDRDQINIAEWAVRMHGEGKLDKIADARIAGEVNDNSLRKFAETAERCLADYGADRPSMGDVLWNLEYCLQLQETHVNRDAFEDSGAVATQLPADVVVPRWVPSSTSLLMMDDADEAGLSMTELADSQVFSQLNARGEGR

>Aet-CrRLK1L6(XP_020174759.1)

MAVRGILLALLLAMVLPRAILAAFSPGFQYFLACGANSSVSFPSDSPANIFVPDAAYLSPAGAPAVSASSTLASPPALYAAARADISAFSYRLPSPASPDTSSFLVLRLHFFPFFPATSSQYVINILSARFNVSVADAYALLSSFSPPAAGVVKEFFVPRDLFGGHFHVTFTPDAGSTAFVNAIELFSAPPEMLWNGPVTPVGAVVKDDMDLWQRQPLETVYRLNVGGSKVIIENDTLWRTWLPDGPYLYDASGLSVVSNTSSPIIYNSTNGYTREVAPDVVYQTQRMANVTDLLAATTPGLNFNLTWTFPAVKGSRYLVRLHFCDYEVVSSVVGVGIVFNVYIAQAIGTPDLTPKAWATQSNEVFYMDYAARAPSAGNLTVSIGWSSQSSGGGILNGLEIMRLPPVDLSSRRYGRTKQRTIVITVSAVLGAAVLACVVLCFFGVPYTKYSGSGWAEQFMNRWSRERKTGGMESVSRKLHIALAKIKAATDNFHERNLIGVGGFGNVYKGVLGDGTPVAVKRAMRASQQGLPEFQTEIVVLSGIRHRHLVSLIGYCNEQAEMILVYEYMEKGTLRSHLYGSDEPALSWKQRLEICVGAARGLHYLHRGYAENIIHRDVKSTNILLGSDDGSTGGVIAKVADFGLSRIGPSFGETHVSTAVKGSFGYLDPGYFKTQQLTDRSDVYSFGVVLCARPVIDQSLDQSLINIAEWAVRMRGEGRLDKMADPRIAGEVDEESLLKFAETAEKCLAECWVDRPSMGDVLWNLEYCLQLQETNITGDELDNMRPSSTSLLMDETGLSMTNVADSKVFSQRSACGEGR

>Aet-CrRLK1L7(XP_020194435.1)

MPPVLDMLVRLLVASVLLGAASGAFTPADNYLVLCGTSASATVAAGRTFVGDARLPAKSLAAPQSVEANTSRTAVVPSGESELYRSARVFTAPASYTFAVKQPGRHFVRLHFFPFPYRSYDMVADAAFNVSVQGAVLVNGYTPKNGTAELREFSVNVTGDTLVIAFAPTGKLAFVNAIEVVSVPDELIADMARMVDGAVQYTGLSTQALETIHRINMGVPKITPGNDTLGRTWLPDQSFQVNTDLAQHKDAKPLTIKYDEKSALSSAYTAPAEVYATATRLSTAGETSTINVQFNISWRFDAPAGSDYLLRFHWCDIVSKAAMGMAFNVYVGGAVVLDNYEISRDTFNRLSIPVYKDFLLGAKDAKGAITVSIGSSTEDNALPDGFLNGLEIMSIVGSAGAGAAATSPRSSKVKIGIIAGSAVCGATLVMVLGFIAFKMLRGREPEKKKPADAWSPFSASALGSRSRSRSFSKSNGNTVLLGQNGAGAGYRIPFAALQEATGGFDEGMVIGEGGFGKVYKGTMRDETVVAVKRGNRRTQQGLHEFHTEIEMLSRLRHRHLVSLIGYCDERGEMILVYEYMAMGTLRSHLYGAGLPPLSWEQRLEACIGAARGLHYLHTGSAKAIIHRDVKSANILLDESFMAKVADFGLSKNGPELDKTHVSTKVKGSFGYLDPEYFRRQMLTEKSDVYSFGVVLLEALCARTVIDPTLPREMVSLAEWATPCLRNGQLDQIVDQRIAGTIRPGSLKKLADTAEKCLAEYGVERPTMGDVLWCLEFALQLQVGSSDSSDVDTMLPPAPPVPVKTPEVQRSLSAATVATDAAAMTTNLGDLDGMSLSGVFSNMIKSDEVR

>Aet-CrRLK1L8(XP_020150664.1)

MQSELHTARSRAMGGGPRCALLLLAAAAACAALVPAAWAQAPTAPAPSGAPFVPRDDILLDCGATGKGNDTDGREWRGDAGSKYAPPNLASADAGAQDPSVPQVPYLTARVSAAAFTYSFPLGPGRKFLRLHFYPANYSNRDAADAFFSVSVPAAKVTLLSNFSAYQTATALNFAYLVREFSVNVTGPTLDLTFTPEKGRPNAYAFINGIEVVSSPDLFDLATPLFVTGDGNNQPFPMDPGAALQTMYRLNVGGQAISPSKDSGGARSWDDDTPYIYGAGAGVTYPNDPNVTITYPDNVPGYVAPSDVYATARSMGIDKNVNLAYNLTWIVQVDAGFTYLVRLHFCEIQSPITKPNQRVFNIYLNNQTAVEGADVIQWVDPLSTGTPLYKDYVVSTVGSGIMDFWVALHPNTGSKPQYYDAILNGMEVFKLQLSNGSLAGPNPVPSADPPAHTGQEKKNSLVGPIAGGVIGGLVVLALGYCCFICKRRRKVAKDAGMSDGHSGWLPLSLYGNSHTSSSAKSHATGSIASSLPSNLCRHFSFAEIKAATKNFDESRILGVGGFGKVYQGEIDGGTTKVAIKRGNPLSEQGIHEFQTEIEMLSKLRHRHLVSLIGYCEDKNEMILVYDHMAHGTLREHLYKTQNAPLSWRQRLEICIGAARGLHYLHTGAKHTIIHRDVKTTNILLDEKWVAKVSDFGLSKTGPSMDHTHVSTVVKGSFGYLDPEYFRRQQLTEKSDVYSFGVVLFEVLCARPALNPTLAKEEVSLAEWALHCQKKGILDQIVDPYLKGKIVPQCFKKFAETAEKCVADNGIERPSMGDVLWNLEFALQMQESAEESGSFGCGISDEEGTPLVMAGKKDPNDPSIDSSTTTTTTTSLSMGDQSVASIDSDGLTPSAVFSQIMNPKGR

>Aet-CrRLK1L9(XP_020148915.1)

MLRMRILVLAAVSIVFANLQFLKAHGRELFLSCGSNATADADGRRWIGDMAPGLNFTLSSPGIAALLAGSSNGSEIMAPVYRSARFFTTTSWYDFSLLPGNYCVRLHFFPSTFRNFSANGSVFDVVANDFKLVSKFNVSEEIVWRNSVSNSAATAVVKEYFLAVNSSRLQIEFDPRPGSFAFVNAIEVMLTPDNSFNGTVNKVGGVDAHIPPELSGRAVETMYRLNIGGPALASSHDQYLHRPWYTDEAFMFSANAALIVSNTSAIKYVSSNDSSIAPIDVYETARIMGNNMVMDKRFNVTWRFLVHPNFDYLVRLHFCELVYDKPSQRIFKIYINNKTAAENYDVYNRAGGINKAYHEDYFDSLPQQVDSLWLQLGPDSMTSASGTDALLNGLEIFKLSRSGNLDYVLGHIDMGNKRGRSKGRSRIGLWEEVGIGSAAFVALASVALFSWCYVRRKRKAVNEEVPAGWHPLVLHEAMKSTTDARASKKAPLARNSSSIGHRMGRRFSIADIRAATKNFDESLVIGSGGFGKVYKGEVDDGITVAIKRANPLCGQGLKEFETEIEMLSKLRHRHLVAMIGYCEEQKEMILVYEYMAKGTLRSHLYGSGLPPLTWKQRIDACIGAARGLHYLHTGADRGIIHRDVKTTNILLDKNFVAKIADFGLSKTGPTLDQTHVSTAIRGSFGYLDPEYFRRQQLTQKSDVYSFGVVLFEVACARPVIDPSVPKDQINLAEWAMRWQRQRSLEAIADPRLDGDYSPESLKKFGDIAEKCLADDGRTRPSMGEVLWHLEYVLQLHEAYKRNVDCESFGSSELGFADMSFSMPHIREGEEEHHPKKSGIREDSAP

>Aet-CrRLK1L10(XP_020151474.1)

MGTTTGQKTALLLLGTLWVLLGTCNAAEFSPADNYLINCGSTVDANLHDGRVFKADNSGSTILTSHHSVPANTLPDAVISSDNPVLYQTARIFIVPSSYSFNMKSRGRHFVRLHFFGFRYQSYDLAAAKFKVSTQHVVLLDNFTPPSNSSPLVREYSLNITEDMLILSFVPLGNSTSFINAIEVISVPDDLIQDSAQTVNPSGQYLGLATQSFQTFYRINVGGREVTVVNDTLSRSWDTDQNFFINSTTTELFAYQGRLNYQKGAATKEDAPDSVYNTARRLAVQNRTSPASNMTWQFDVDGRSSYLIRFHFCDIVSKAAYSLYFDIYVDGGLALENLDLSEKVFGTLAVPYYTEFVLKSSNPSGKLSVGIGPSSLNNVALDGILNGLEIMKMNISTGTIYVVWPPATPKRKLAIILGPVLGGVGAVSIAIILCFVLRRKKKEKKPRRAPTSRPSSSWSPLTLNGLSFLSIGTRTTSRTTHTSGTNSDVSYRIPFALLQVATKHFDEQMVVGVGGFGKVYKAVLQDSTKVAVKRGNQKSHQGLKEFRTEIELLSGLRHRHLVSLIGYCDEQNEMILVYEYMEKGTLKSHLYGSDMPPLSWKKRVEICIGAARGLHYLHTGFAKSIIHRDVKSANILLDENLMAKVSDFGLSKTGPELDQTHVSTAVKGSFGYLDPEYYRRQKLTDKSDVYSFGVVLLEVICARPVIDPTLPRDMINLAEWAIKWQKRGELGQIVDQRIAGTIRPESLRKYGETVEKCLADYGVDRPTMGDVLWNLEFVLQLQEAGPDISNVDSMNQISELPSGARRMGSLEIGTADEADEGRTNMDYSQMSTNDAFSQLMNTEGR

>Aet-CrRLK1L11(XP_020184558.1)

MAAARGRGVLLAVLLLMMVAFAFVDADIYKPTDSILVHCGSDKDGQDEDGRKWTADKDSKWLPDGGKSSVMGTADVPDPSLPSPVPYMTARVFPKETAYTFPVADADRHWVRLHFYPAAYHGIPADHFFFSVTTSTGVTLLRNFSVYTTAKALTQAYIVREFSLPPSTTGSLSLKFTPTAMNNASYAFVNGIEIISMPSFFGDPATLVGLDDQSLDASAGNLQTMYRLSVGGSYIPPANDSGLSREWFSDTPYVYGAATGVTFEANDTIPIKYPTPADEYAAPVSIYDSFRHMGRDPKMNRNNNLTWVFEVDGNFTYLLRLHFCSLMEDKINQVVFAILVNNKTATTTGSADIIAWAKEKNPANPGAPGKGVPVFKDYAVFMPAAPAGSDTILWLTLRPDTATNPQFVNAFLNGLEVFKVSDASGNLAGPNPDISKMLAEAELGAVDGQFREKPSNVGALIGGAAGGAAAFGLVAAVCFVAYQSKRRRELSSSPSHSSSGWLPVYGGNSQTSVSKSSGGRSAVTLNPNITAMCRHFSFQEIKSATKGFDESLVIGVGGFGKVYRGVVDGDTKVAIKRSNPSSEQGVLEFQTEIEMLSKLRHKHLVSLIGCCEDNGEMILVYDYMAHGTLREHLYKSGKPPLPWRQRLEIVIGAARGLHYLHTGAKYTIIHRDVKTTNILVDEKWVAKVSDFGLSKTGPTVQNQTHVSTMVKGSFGYLDPEYFRRQKLTEKSDVYSFGVVLFEVLCGRPALNPSLPREQVSLADHALSCQRKGTLEEIIDPVLEGKIAPDCLKKFAETAEKCLADQGVDRPSMGDVLWNLEFALQQQDTFENGGKPPEVDDYSSSFTITPPSMEESLAANAAALSLISEDMDEEDIANSVIFSQIAKPTGR

>Aet-CrRLK1L12(XP_020175506.2)

MVDEPPNIARRRPGQTGLNLAWRHPPPLNPRPFLPPPQVSSAPFPLLRLAPPNQREGERAPPQPGPGHHNNKNQHDDVPGVGRCASRWRRRPRCRLERRHNGSVGVRVTRRSTAPKMVRRGALPLALLAVLATLTAVAGQGKPVTDNGSGGGAGPAKFTPKDAFYIDCGGTAAADTKDGKSFKTDAEANSLLSARDNIKVADDKADVPSHLYRSARVFKEEAVYNFPLTAPGWHFIRLYFFPIKSGEADLAAATFDVSTAVNVLLHGFTPEAKAVMKEYIVNATENKLELKFTPQSGSAFINAIEVVNAPDELISKTALTVSPLAETSGLSEAAYQVVCRLNVGGPPIGPVNDTLGRQWEDDEKYLNPKEAGTEVSVPTSAIKYPDAFPATKLVAPTAVYATARHMAESGVANQNFNVSWKVDVDPSFDYLVRLLFADIISTSANDLYFNVYINGRKAISALDLSTITGDLAAPYYKDFVVNSSVNTDGHIIIDVGPLGQDTGRNDALLNGAEVLKMSNSVGSLDGEYGVDGRMVDDGSGTRKVVAAVGFAMMFGAFAGLGCMVVKWHRRPQDWERRNSFSSWLLPIHTGQSFSNGKSKSGYTFSSTAGLGHFFTFAEMSEATKNFDESAIIGVGGFGNVYVGEINDPDEEGSRIKVAIKRGNPSSEQGINEFNTEIQMLSKLRHRHLVSLIGYCDEGEEMILVYEFMQHGPFRDHIYGGPEGLPTLSWKQRLEICIGAARGLHYLHTGTAHGIIHRDVKTTNILLDDKFVAKVADFGLSKDGPGMNQLHVSTAVKGSFGYLDPEYFRCQQLTDKSDVYSFGVVLLETLCARAPIDPQLPREQVSLAEWGLQWKRKGLIEKIMDPNLNGKVNPESLAKFAETAEKCLCEFGSDRLSMGDVLWNLEYALQLQEANPPEGATDADDADASIVSSASGVTTVPDQSTTSANELFAQLADMKGR

>Aet-CrRLK1L13(XP_045086556.1)

MAGAAERTSRPPSSSTPLSAACGRRGDPSPTRTDFYLANIMMGRRKLQVVTLAILCFWSSAGVCKAQTVDFKPADSYLVDCGSTKGTTVLGRDFAADGASPVTVSTSQDILAGTSANGVSSFDNPVLYQTARIFTSPSSYTFPIQKQGRHFVRLYFYPFIYQSYDLSTAKFTVSTQDVLLLSDFQQPDKTAPLFKEYSLNITRDQLVISFKPSNGIAFINAIEVVSVPDDLIADVANMVNPVQQYSGLTTQSLETVYRVNMGGPKVFPNNDTLSRTWQKDQKYILNPSVTKTAVYGKAIKYRKGGATPLTAPDIVYSTATELAASNTSNALFNMTWQFDVDAGFSYLIRFHFCDIVSKALNQLYFNAYVGGFFAQHDLDLSEQSVNQLATAIYVDVVLSSNDASSKLSISIGPSTLNNALPDGILNGLEIMKMGSGSGSAFTVGNNGSNKKLPIIIGSVLGVVGLLIIVLVVVLLCRRKKTDDKQHSKTWMPFSINGLTSLSTGSRTSYGTTLTSGLNGSYGYRFAFNVLQEATNNFDESWVIGVGGFGKVYKGALRDDTKVAVKRGNPKSQQGLNEFRTEIELLSRLRHRHLVSLIGYCDERNEMILVYEYMENGTVKSHLYGSDNPSLNWKQRLEICIGAARGLHYLHTGSAKAIIHRDVKSANILLDENLLAKVADFGLSKTGPELDQTHVSTAVKGSFGYLDPEYFRRQQLTEKSDVYSFGVVMLEVLCARPVIDPSLPREMVNLAEWGMKWQKRGELHQIVDQKLSGAIRPDSLRKFGETVEKCLADYGVERPSMGDVLWNLEYVLQLQDVDSSTVSDVNSMNRIVDLSSQVQHVGAMESISVTMAEDGALHEPDHDLSDVSMSRVFSQLIKAEGR

>Aet-CrRLK1L14(XP_020201562.1)

MKSSANFLSILVLLVFLAAENARAQPQPILINCGSDSTTSVDARTWIGDSSPSNNFTLSFPGAIASAAPAPAPGVDGEQDPYGDLYKTARVFNASSSYRLAVAPGSYFLRLHFSQQFANLGAQEPIFSVAANGLRLLSKFSVHGEISWRDSQINSTSSVIVKEYLLNVTSGKLGIEFTPDEGSFAFINAMEVLPVSGTSIFDSVNKVDAHGLKGPFSLDGDGIETMYRLCVGCIDVLPRKEDPGLWRRWDKDEHFIFSLNAANSIFNSSNISYVSADDPTVAPLRLYQSARVPTESSVLGKKFNVSWSFNIDPGFDYLVRLHFCELQYDKAEQRKFKIYINNKTAAESYDVFARAGGKNKAFYEDFLDAASPQMDTLWVQLGAESSAGSAAADALLNGMEIFKVSREGNLAHPTVRIGGISGGASKPKRSPKWVLIGTASGLIIFIAIAGGLYFGFNLRRKKNSSASKAKDNLHGATHTRSPTLRTAGAFGSNRMGRRFTIAEIRTATVNFDESLVIGVGGFGKVYKGIMEDGTRVAIKRGHTDSHQGQGVKEFETEIEMLSRLRHRHLVPLIGYCDEQNEMVLVYEHMANGTLRSHLYGSDLPALTWKQRLEICIGAARGLHYLHTGLDRGIIHRDVKTTNILLDDNLVAKMADFGISKDGPALDHTHVSTAVKGSFGYLDPEYYRRQQLTPSSDVYSFGVVLFEVLCARPVINPTLPRDQINLADWALNRQRHRLLETIIDLRLDGNYTLASVKKSSKIAEKCLADEGVNRPSMGEVLWHLESALQLEQGHPQSTNADGCSDPQLKPSDVPTRVACIKEDEQSTRPGSHNSDGQVVDVKIEVP

>At-FER(AT3G51550.1)

MKITEGRFRLSLLLLLLLISAATLISAADYSPTEKILLNCGGGASNLTDTDNRIWISDVK

SKFLSSSSEDSKTSPALTQDPSVPEVPYMTARVFRSPFTYTFPVASGRKFVRLYFYPNSY

DGLNATNSLFSVSFGPYTLLKNFSASQTAEALTYAFIIKEFVVNVEGGTLNMTFTPESAP

SNAYAFVNGIEVTSMPDMYSSTDGTLTMVGSSGSVTIDNSTALENVYRLNVGGNDISPSA

DTGLYRSWYDDQPYIFGAGLGIPETADPNMTIKYPTGTPTYVAPVDVYSTARSMGPTAQI

NLNYNLTWIFSIDSGFTYLVRLHFCEVSSNITKINQRVFTIYLNNQTAEPEADVIAWTSS

NGVPFHKDYVVNPPEGNGQQDLWLALHPNPVNKPEYYDSLLNGVEIFKMNTSDGNLAGTN

PIPGPQVTADPSKVLRPTTRKSKSNTAIIAGAASGAVVLALIIGFCVFGAYRRRKRGDYQ

PASDATSGWLPLSLYGNSHSAGSAKTNTTGSYASSLPSNLCRHFSFAEIKAATKNFDESR

VLGVGGFGKVYRGEIDGGTTKVAIKRGNPMSEQGVHEFQTEIEMLSKLRHRHLVSLIGYC

EENCEMILVYDYMAHGTMREHLYKTQNPSLPWKQRLEICIGAARGLHYLHTGAKHTIIHR

DVKTTNILLDEKWVAKVSDFGLSKTGPTLDHTHVSTVVKGSFGYLDPEYFRRQQLTEKSD

VYSFGVVLFEALCARPALNPTLAKEQVSLAEWAPYCYKKGMLDQIVDPYLKGKITPECFK

KFAETAMKCVLDQGIERPSMGDVLWNLEFALQLQESAEENGKGVCGDMDMDEIKYDDGNC

KGKNDKSSDVYEGNVTDSRSSGIDMSIGGRSLASEDSDGLTPSAVFSQIMNPKGR

>At-HERK1(AT3G46290.1)

MGIEKFETFILISTISILLCICHGFTPVDNYLINCGSPTNGTLMGRIFLSDKLSSKLLTS

SKEILASVGGNSGSDIYHTARVFTEVSSYKFSVTRGRHWVRLYFNPFDYQNFKMGSAKFA

VSSQSHVLLSDFTVTSSKVVKEYSLNVTTNDLVLTFTPSSGSFAFVNAIEVISIPDTLIT

GSPRFVGNPAQFPDMSMQGLETIHRVNMGGPLVASNNDTLTRTWVPDSEFLLEKNLAKSM

SKFSTVNFVPGYATEDSAPRTVYGSCTEMNSADNPNSIFNVTWEFDVDPGFQYYFRFHFC

DIVSLSLNQLYFNLYVDSMVAATDIDLSTLVDNTLAGAYSMDFVTQTPKGSNKVRVSIGP

STVHTDYPNAIVNGLEIMKMNNSKGQLSTGTFVPGSSSSSKSNLGLIVGSAIGSLLAVVF

LGSCFVLYKKRKRGQDGHSKTWMPFSINGTSMGSKYSNGTTLTSITTNANYRIPFAAVKD

ATNNFDESRNIGVGGFGKVYKGELNDGTKVAVKRGNPKSQQGLAEFRTEIEMLSQFRHRH

LVSLIGYCDENNEMILIYEYMENGTVKSHLYGSGLPSLTWKQRLEICIGAARGLHYLHTG

DSKPVIHRDVKSANILLDENFMAKVADFGLSKTGPELDQTHVSTAVKGSFGYLDPEYFRR

QQLTDKSDVYSFGVVLFEVLCARPVIDPTLPREMVNLAEWAMKWQKKGQLDQIIDQSLRG

NIRPDSLRKFAETGEKCLADYGVDRPSMGDVLWNLEYALQLQEAVIDGEPEDNSTNMIGE

LPPQINNFSQGDTSVNVPGTAGRFEESSIDDLSGVSMSKVFSQLVKSEGR

>At-HERK2(AT1G30570.1)

MSKLRKKYLEHLLCVLIFFTYVIGYGEAQSKSFLVDCGSNATTEVDGRTWVGDLSPNKSV

TLQGFDAITASTSKGSSVYAEIYKTARVFDAVLNYTFEGITQGNYFVRLHFSPFAIENHN

VNESSFSVFADGLRLMLDINIAGEIAHKNLILESTGHNATASSLVKEFLLPTGPGKLVLS

FIPEKGSFGFVNAIEIVSVDDKLFKESVTKVGGSEVELGLGGRGIETMYRLNVGGPKLGP

SKDLKLYRTWETDLSYMVIENAGVEVKNSSNITYALADDSPVAPLLVYETARMMSNTEVL

EKRFNISWKFEVDPNFDYLVRLHFCELLVDKQNQRIFRIYINNQTAAGNFDIFAHAGGKN

KGIYQDYLDPVSSKNDVLWIQLGPDSSVGASGDALLSGLEIFKLSKNGNLAHLIRFDSTG

HSVSDSKMRIIWISVGAGIAIIIFFVFLGILVVCLCKKRRSKSDESKNNPPGWRPLFLHV

NNSTANAKATGGSLRLNTLAASTMGRKFTLAEIRAATKNFDDGLAIGVGGFGKVYRGELE

DGTLIAIKRATPHSQQGLAEFETEIVMLSRLRHRHLVSLIGFCDEHNEMILVYEYMANGT

LRSHLFGSNLPPLSWKQRLEACIGSARGLHYLHTGSERGIIHRDVKTTNILLDENFVAKM

SDFGLSKAGPSMDHTHVSTAVKGSFGYLDPEYFRRQQLTEKSDVYSFGVVLFEAVCARAV

INPTLPKDQINLAEWALSWQKQRNLESIIDSNLRGNYSPESLEKYGEIAEKCLADEGKNR

PMMGEVLWSLEYVLQIHEAWLRKQNGENSFSSSQAVEEAPESFTLPACSNQDSSETEQSQ

TGSALHNSA

>At-THE1(AT5G54380.1)

MVFTKSLLVLLWFLSCYTTTTSSALFNPPDNYLISCGSSQNITFQNRIFVPDSLHSSLVL

KIGNSSVATSTTSNNSTNSIYQTARVFSSLASYRFKITSLGRHWIRLHFSPINNSTWNLT

SASITVVTEDFVLLNNFSFNNFNGSYIFKEYTVNVTSEFLTLSFIPSNNSVVFVNAIEVV

SVPDNLIPDQALALNPSTPFSGLSLLAFETVYRLNMGGPLLTSQNDTLGRQWDNDAEYLH

VNSSVLVVTANPSSIKYSPSVTQETAPNMVYATADTMGDANVASPSFNVTWVLPVDPDFR

YFVRVHFCDIVSQALNTLVFNLYVNDDLALGSLDLSTLTNGLKVPYFKDFISNGSVESSG

VLTVSVGPDSQADITNATMNGLEVLKISNEAKSLSGVSSVKSLLPGGSGSKSKKKAVIIG

SLVGAVTLILLIAVCCYCCLVASRKQRSTSPQEGGNGHPWLPLPLYGLSQTLTKSTASHK

SATASCISLASTHLGRCFMFQEIMDATNKFDESSLLGVGGFGRVYKGTLEDGTKVAVKRG

NPRSEQGMAEFRTEIEMLSKLRHRHLVSLIGYCDERSEMILVYEYMANGPLRSHLYGADL

PPLSWKQRLEICIGAARGLHYLHTGASQSIIHRDVKTTNILLDENLVAKVADFGLSKTGP

SLDQTHVSTAVKGSFGYLDPEYFRRQQLTEKSDVYSFGVVLMEVLCCRPALNPVLPREQV

NIAEWAMAWQKKGLLDQIMDSNLTGKVNPASLKKFGETAEKCLAEYGVDRPSMGDVLWNL

EYALQLEETSSALMEPDDNSTNHIPGIPMAPMEPFDNSMSIIDRGGVNSGTGTDDDAEDA

TTSAVFSQLVHPRGR

>At-ANX1(AT3G04690.1)

MSGKTRILFFLTCLSFLLVFPTRSNGQDLALSCGTSEASADQDKKKWEPDTKFLKTGNSI

HATATYQDPSLLSTVPYMTARIFTAPATYEIPIKGDKRHLLRLYFYPSTYTGLNISNSYF

TVEANDVTLLSNFSAAITCQALTQAYLVKEYSLAPTDKDVLSIKFTPSDKYRDAFAFING

IEVIQMPELFDTAALVGFTDQTMDAKTANLQSMFRLNVGGQDIPGSQDSGGLTRTWYNDA

PYIFSAGLGVTLQASNNFRINYQNMPVSIAPADIYKTARSQGPNGDINLKSNLTWMFQID

KNFTYILRLHFCEFQLSKINQKVFNIYINNRTAQADTTPADIIGWTGEKGIPMYKDYAIY

VDANNGGEEITLQMTPSTFGQPEYYDSSLNGLEIFKMDTMKNLAGPNPEPSPMQAEEEVK

KEFKNEKRHAFIIGSAGGVLAVLIGALCFTAYKKKQGYQGGDSHTSSWLPIYGNSTTSGT

KSTISGKSNNGSHLSNLAAGLCRRFSLPEIKHGTQNFDDSNVIGVGGFGKVYKGVIDGTT

KVAVKKSNPNSEQGLNEFETEIELLSRLRHKHLVSLIGYCDEGGEMCLVYDYMAFGTLRE

HLYNTKKPQLTWKRRLEIAIGAARGLHYLHTGAKYTIIHRDVKTTNILVDENWVAKVSDF

GLSKTGPNMNGGHVTTVVKGSFGYLDPEYFRRQQLTEKSDVYSFGVVLFEILCARPALNP

SLPKEQVSLGDWAMNCKRKGNLEDIIDPNLKGKINAECLKKFADTAEKCLNDSGLERPTM

GDVLWNLEFALQLQETADGTRHRTPNNGGSSEDLGRGGMAVNVAGRDDVSDLSSEDNTEI

FSQIVNPKGR

>At-ANX2(AT5G28680.2)

MNEKLRILFSFLCFFYVLLVSPSQSNGQDISLSCGASEPAVDQDKKKWEPDTKFLKTPNT

VHAPATYQDPSLLSTVPYMTSRIFTAPATYEIPVKGDKRHMLRLHFYPSTYTGLNILDSY

FSVAANDLTLLSNFSAAITCQALTQAYLVREYSLAPSEKDVLSIIFTPSDKHPKAFAFIN

GIEVIPMPELFDTASLVGFSDQTSDTKTANLQTMFRLNVGGQDIPGSQDSGGLTRTWYND

APYIFSAGLGVTLQASNNFRIDYQKMPVSTAPADVYKTARSQGPNGDINMKSNLTWMFQV

DTNFTYIMRLHFCEFQLAKINQKVFNIFINNRTAQGDTNPADILGWTGGKGIPTYKDYAI

YVDANTGGGGEEISLQMTPSTFGQPEYYDSQLNGLEIFKIDTMKNLAGPNPKPSPMQANE

DVKKDFQGDKRITAFVIGSAGGVAAVLFCALCFTMYQRKRKFSGSDSHTSSWLPIYGNSH

TSATKSTISGKSNNGSHLSNLAAGLCRRFSLSEIKHGTHNFDESNVIGVGGFGKVYKGVI

DGGTKVAIKKSNPNSEQGLNEFETEIELLSRLRHKHLVSLIGYCDEGGEMCLIYDYMSLG

TLREHLYNTKRPQLTWKRRLEIAIGAARGLHYLHTGAKYTIIHRDVKTTNILLDENWVAK

VSDFGLSKTGPNMNGGHVTTVVKGSFGYLDPEYFRRQQLTEKSDVYSFGVVLFEVLCARP

ALNPSLSKEQVSLGDWAMNCKRKGTLEDIIDPNLKGKINPECLKKFADTAEKCLSDSGLD

RPTMGDVLWNLEFALQLQETADGSRHRTPSNGGGSVDLGGGGGGVTVNISAGESDLGDDL

SSEENSGIFSQIVNPKGR

>At-BUPS1(AT4G39110.1)

MEIRKKPNIFTVLVIDFSSKPSMALLLAILLFLSGPSASAVAAAAVGPATGFKPADDILI

DCGSKSSSKTPDGRVFKSDQETIQYIEAKEDIQVSAPPSDKVASPIYLTARIFREEATYK

FHLTRPGWHWVRLHFLAFPNDKFDLQQATFSVLTEKYVLLHNFKISNNNNDSQAAVQKEY

LVNMTDAQFALRFRPMKSSAAFINAIEVVSAPDELISDSGTALFPVIGFSGLSDYAYQSV

YRVNVGGPLIMPQNDTLGRTWIPDKEFLKDENLAKDVKTTPSAIKYPPEVTPLIAPQTVY

ATAVEMANSLTIDPNFNVSWNFPSNPSFNYLIRLHFCDIVSKSLNDLYFNVYINGKTAIS

GLDLSTVAGNLAAPYYKDIVVNATLMGPELQVQIGPMGEDTGTKNAILNGVEVLKMSNSV

NSLDGEFGVDGRTTGMGKHGMVATAGFVMMFGAFIGLGAMVYKWKKRPQDWQKRNSFSSW

LLPIHAGDSTFMTSKGGSQKSNFYNSTLGLGRYFSLSELQEATKNFEASQIIGVGGFGNV

YIGTLDDGTKVAVKRGNPQSEQGITEFQTEIQMLSKLRHRHLVSLIGYCDENSEMILVYE

FMSNGPFRDHLYGKNLAPLTWKQRLEICIGSARGLHYLHTGTAQGIIHRDVKSTNILLDE

ALVAKVADFGLSKDVAFGQNHVSTAVKGSFGYLDPEYFRRQQLTDKSDVYSFGVVLLEAL

CARPAINPQLPREQVNLAEWAMQWKRKGLLEKIIDPHLAGTINPESMKKFAEAAEKCLED

YGVDRPTMGDVLWNLEYALQLQEAFTQGKAEETENAKPDVVTPGSVPVSDPSPITPSVTT

NEAATVPVPAKVEENSGTAVDEHSGTAMFTQFANLNGR

>At-BUPS2(AT2G21480.1)

MEIRKKPNIPMCLVLDSSSRPFMTLLFTILLFLTGLASAVGAVGGSPTAGFKPADDILID

CGSKSSTKTPEGRVFKSDSETVQYIEAKDDIQVSAPPSDKLPSPIYLTAKIFREEAIYKF

HLTRPGWHWVRLHFFAFPNDKFDLQQATFSVLTEKYVLLHNFKLSNDNNDSQATVQKEYL

LNMTDAQFALRFKPMKGSAAFINGIELVSAPDELISDAGTSLFPVNGFSGLSDYAYQSVY

RVNVGGPLITPQNDTLGRTWTPDKEYLKDENLAKDVKTNPTAIIYPPGVTPLIAPQTVYA

TGAEMADSQTIDPNFNVTWNFPSNPSFHYFIRLHFCDIISKSLNDLYFNVYINGKTAISG

LDLSTVAGDLSAPYYKDIVVNSTLMTSELQVQIGPMGEDTGKKNAILNGVEVLKMSNSVN

SLDGEFGVDGQRASMGKQGMVATAGFVMMFGAFVGLGAMVYKWKKRPQDWQKRNSFSSWL

LPIHAGDSTFMTSKTGSHKSNLYNSALGLGRYFSLSELQEVTKNFDASEIIGVGGFGNVY

IGTIDDGTQVAIKRGNPQSEQGITEFHTEIQMLSKLRHRHLVSLIGYCDENAEMILVYEY

MSNGPFRDHLYGKNLSPLTWKQRLEICIGAARGLHYLHTGTAQGIIHRDVKSTNILLDEA

LVAKVADFGLSKDVAFGQNHVSTAVKGSFGYLDPEYFRRQQLTDKSDVYSFGVVLLEALC

ARPAINPQLPREQVNLAEWAMLWKQKGLLEKIIDPHLVGAVNPESMKKFAEAAEKCLADY

GVDRPTMGDVLWNLEYALQLQEAFSQGKAEAEEVETPKPVAVPAAAPTSPAATTAAASER

PVSQTEEKDDSTVDQHSGTTMFTQFASLNGR

>At-CURVY1(AT2G39360.1)

MINLKLFLELKLCFLITLLCSSHISSVSDTFFINCGSPTNVTVNNRTFVSDNNLVQGFSV

GTTDSNSGDESTLFQTARVFSDESSSTYRFPIEEHGWFLIRIYFLPLVSASQDLTTARFS

VSAQNFTLIREYKPSTTSVVREYILNVTTDSLLLQFLPRTGSVSFINALEVLRLPETLIP

EDAKLIGTQKDLKLSSHAMETVSRVNMGNLSVSRDQDKLWRQWDSDSAYKAHFGTPVMNL

KAVNFSAGGITDDIAPVYVYGTATRLNSDLDPNTNANLTWTFKVEPGFDYFVRFHFCNII

VDPFGFERQIRFDIFVNSEKVRTIDMTEVLNGTFGAPFFVDAVMRKAKSREGFLNLSIGL

VMDVSSYPVSFINGFEISKLSNDKRSLDAFDAILPDGSSSNKSSNTSVGLIAGLSAALCV

ALVFGVVVSWWCIRKRRRRNRQMQTVHSRGDDHQIKKNETGESLIFSSSKIGYRYPLALI

KEATDDFDESLVIGVGGFGKVYKGVLRDKTEVAVKRGAPQSRQGLAEFKTEVEMLTQFRH

RHLVSLIGYCDENSEMIIVYEYMEKGTLKDHLYDLDDKPRLSWRQRLEICVGAARGLHYL

HTGSTRAIIHRDVKSANILLDDNFMAKVADFGLSKTGPDLDQTHVSTAVKGSFGYLDPEY

LTRQQLTEKSDVYSFGVVMLEVVCGRPVIDPSLPREKVNLIEWAMKLVKKGKLEDIIDPF

LVGKVKLEEVKKYCEVTEKCLSQNGIERPAMGDLLWNLEFMLQVQAKDEKAAMVDDKPEA

SVVGSTMQFSVNGVGDIAGVSMSKVFAQMVREETR

>At-MDS2(AT5G39000.1)

MIRHALLIFSILVSTPIVGEGATSTYEPTDVFLFNCGDTSNNVDVSGRNWTAENQKILSS

NLVNASFTAQASYQESGVSQIPYMTARIFRSEFTYSFPVTPGSNFLRLYFYPTRYGSQFN

AVKSFFSVKVNGFTLLNNFSADLTVKASKPQTEFIIKEFIIPVYQTLNLTFTPSLDSLAF

VNGIEIVSIPNRFYSKGGFDDVITNVGSSVDFHIENSTAFETVYRLNVGGKTVGDSGMFR

RWVSDDEIILSESSGISPIVPDIKINYTEKTPSYVAPDDVYATSRSMGNADHPEQNLNFN

LTWLFTVDAGFSYLVRLHFCETLSEVNKEGQRVFSIFIENQTATLEMDVFRMSGGSWIPM

YLDYTVIAGSGSGRRHDLRLDLHPLVSINPKYYDAILNGVEILKMNDPDGNLAGPNPDPL

VSPDLIPNRATPRIRKNKSHILPITLAVVGSLVVLAMFVVGVLVIMKKKKKSKPSTNSSW

CPLPHGTDSTNTKPAKSLPADLCRRFSIFEIKSATNDFEDKLIIGVGGFGSVYKGQIDGG

ATLVAVKRLEITSNQGAKEFETELEMLSKLRHVHLVSLIGYCDEDNEMVLVYEYMPHGTL

KDHLFRRDKTSDPPLSWKRRLEICIGAARGLQYLHTGAKYTIIHRDIKTTNILLDENFVT

KVSDFGLSRVGPTSASQTHVSTVVKGTFGYLDPEYYRRQVLTEKSDVYSFGVVLLEVLCC

RPIRMQSVPPEQADLIRWVKSNYRRGTVDQIIDSDLSADITSTSLEKFCEIAVRCVQDRG

MERPPMNDVVWALEFALQLHETAKKKNDNVESLDLMPSGEVGTTTDGEDDLFSRTTGHVG

KSTTTDDSVLVVGDERSGSSWGVFSEINEPKAR

>At-MDS3(AT5G39020.1)

MNCNVLFLLSVLVSVTAGVTAAYHPTDVFLFNCGDTSNNVDNSGRNWTVESRQILSSNLV

NASFTSEASYQKAGVSRIPYMKARIFRSEFTYSFPVTPGSIFLRLYFYPTQYKSGFDAVN

SFFSVKVNGFTLLRNFNADSTVQASIPLSNSLIKEFIIPVHQTLNLTFTPSKNLLAFVNG

IEIVSMPDRFYSKGGFDNVLRNVSSDVDFQIDNSTAFESVHRLNVGGQIVNEVDDSGMFR

RWLSDDSFGNSGSIVNVPGVKINYTEKTPAYVAPYDVYATSRLMGNSSNLMFNLTGMFLT

VDAGYNYLVRLHFCETLPQVTKAGQRVFSIFVEDKMAKKETDVIRLSGGPRIPMYLDFSV

YVGFESGMIQPELRLDLVPLKDTNQTYYDAILSGVEILKLNDSDGNLARPNPELLVSTDS

TPDDSNVTPPIKGKPHVLVIILIVVGSVIGLATFIVIIMLLIRQMKRKKNKKENSVIMFK

LLLKQYIYAELKKITKSFSHTVGKGGFGTVYRGNLSNGRTVAVKVLKDLKGNGDDFINEV

TSMSQTSHVNIVSLLGFCYEGSKRAIISEFLEHGSLDQFISRNKSLTPNVTTLYGIALGI

ARGLEYLHYGCKTRIVHFDIKPQNILLDDNFCPKVADFGLAKLCEKRESILSLIDTRGTI

GYIAPEVVSRMYGGISHKSDVYSYGMLVLDMIGARNKVETTTCNGSTAYFPDWIYKDLEN

GDQTWIIGDEINEEDNKIVKKMILVSLWCIRPCPSDRPPMNKVVEMIEGSLDALELPPKP

SRHISTELVLESSSLSDGQEAEKQTQTLDSTII

>At-ANJEA(AT5G59700.1)

MGGEKFGFLIWILSIPCLIFLCYGYVPVDNYLINCGSSTNVTVTSRVFISDNLASNFLTS

PNEILAASNRNSNSDIYQTARIFTGISKYRFSVARGRHWIRLHFNPFQYQNFQMVSAKFS

VSSETHVLLSDFTVSSRVMKEYSLNVATDHLELTFTPSGDSFAFLNALEVVSVPDTLFSG

DPSFAGSPGKFQGLSWQALETVYRVNMGGPRVTPSNDTLSRIWEPDSEFLVEKNLVKSVS

KIASVDYVPGFATEETAPRTVYGTCTEMNSADNPSSNFNVTWDFDVDPGFQYFLRFHFCD

IVSKALNQLYFNLYVDSMDVVENLDLSSYLSNTLSGAYAMDFVTGSAKLTKRIRVSIGRS

SVHTDYPTAILNGLEIMKMNNSKSQLSIGTFLPSGSSSTTKKNVGMIIGLTIGSLLALVV

LGGFFVLYKKRGRDQDGNSKTWIPLSSNGTTSSSNGTTLASIASNSSYRIPLVAVKEATN

SFDENRAIGVGGFGKVYKGELHDGTKVAVKRANPKSQQGLAEFRTEIEMLSQFRHRHLVS

LIGYCDENNEMILVYEYMENGTLKSHLYGSGLLSLSWKQRLEICIGSARGLHYLHTGDAK

PVIHRDVKSANILLDENLMAKVADFGLSKTGPEIDQTHVSTAVKGSFGYLDPEYFRRQQL

TEKSDVYSFGVVMFEVLCARPVIDPTLTREMVNLAEWAMKWQKKGQLEHIIDPSLRGKIR

PDSLRKFGETGEKCLADYGVDRPSMGDVLWNLEYALQLQEAVVDGDPEDSTNMIGELPLR

FNDYNHGDTSVNFSVAKEGRFDEEESSVDDSSGVSMSKVFSQLIKSEGR

>At-CAP1(AT5G61350.1)

MGGDFRHFSSHVSLLLLFLLIVKSSSSFTPADNYLIDCGSSDETKLSDGRNFKSDQQSVA

FLQTDEDIKTSVDSIPITDSNASTLPLYLTARIFAGKSTYSFYISRPGRHWIRLHFYPLN

HPLYNLTNSVFSVTTDTTVLLHDFSAGDTSSIVFKEYLIYAAEKLSLYFKPHKGSTAFIN

AVEIVSVPDELVPDSASSVPQAPDFKGLSSFSLEILHRINIGGDLISPKIDPLSRTWLSD

KPYNTFPEGSRNVTVDPSTITYPDGGATALIAPNPVYATAEEMADAQTSQPNFNLSWRMS

VDFGHDYFIRLHFCDIVSKSLNDLIFNVFINKLSAISALDLSSLTSALGTAYYADFVLNA

STITNGSILVQVGPTPNLQSGKPNAILNGLEIMKLNNAAGSLDGLFGVDGKYKGPIGGMS

SKKLAIAGIGFVMALTAFLGVVVLLVRWQRRPKDWQKQNSFSSWLLPLHASHSSYISSKG

GSTSRRMSIFGSKKSKSNGFSSFFSNQGLGRYFPFTELQTATQNFDENAVCGVGGFGKVY

IGEIDGGTQVAIKRGSQSSEQGINEFQTEIQMLSKLRHRHLVSLIGFCDENKEMILVYEY

MSNGPLRDHLYGSKENDPNPIPTLSWKQRLEICIGSARGLHYLHTGAAQGIIHRDVKTTN

ILLDENLVAKVSDFGLSKDAPMDEGHVSTAVKGSFGYLDPEYFRRQQLTDKSDVYSFGVV

LFEVLCARPVINPQLPREQVNLAEYAMNLHRKGMLEKIIDPKIVGTISKGSLRKFVEAAE

KCLAEYGVDRPGMGDVLWNLEYALQLQEASAQVDLSEDKTTMNIEMDLIPGEEMQSPSHS

IP

>At-MDS1(AT5G38990.1)

MICHVLVIFTILVSAVVDATASYEPTDVFLINCGDTSNNMDYSGRNWTTENPKFMSSNAV

DDASFTSSASYQESGIPQVPYLKARIFRYDFTYSFPVSPGWKFLRLYFYPTRYGSDFDAV

KSFFSVNVNRFTLLHNFSVKASIPESSSLIKEFIVPVNQTLDLTFTPSPNSLAFVNGIEI

ISMPDRFYSKGGFDDVVRNVGRDVDFEIDNSTAFETVYRVNVGGKVVGDVGDSGMFRRWL

SDEGFLLGINSGAIPNITGVKINYTDKTPAYVAPEDVYTTCRLMGNKDSPELNLNFNLTW

LFEVDAGFAYIVRLHFCETQPEVNKTGDRVFSIFFGYQLAMREMDVFRLSGGFRLPMYLD

FKVLVDADGTSQRPSLRVDLTPYKEDYPTYYDAILSGVEILKLSNSDGNLAGLNPIPQLS

PPPQSITPLKGKGKSSHVLPIIIAVVGSAVALAFFVLVVVLVVMKRKKKSNESSVDTTNK

PSTNSSWGPLLHGTGSTNTKSASSLPSDLCRRFSIYEIKSATNDFEEKLIIGVGGFGSVY

KGRIDGGATLVAVKRLEITSNQGAKEFDTELEMLSKLRHVHLVSLIGYCDDDNEMVLVYE

YMPHGTLKDHLFRRDKASDPPLSWKRRLEICIGAARGLQYLHTGAKYTIIHRDIKTTNIL

LDENFVAKVSDFGLSRVGPTSASQTHVSTVVKGTFGYLDPEYYRRQILTEKSDVYSFGVV

LLEVLCCRPIRMQSVPPEQADLIRWVKSNFNKRTVDQIIDSDLTADITSTSMEKFCEIAI

RCVQDRGMERPPMNDVVWALEFALQLHETAKKKNDNVESLDLMPSGEVGTTTDGEDDLFS

RTTGHVGKSTTTDDSVLVVGDERSGSSWGVFSEINEPKAR

>At-MDS4(AT5G39030.1)

MICFILFVFSFLVSVSATAPYKPDDVFLINCGETDVPFDNHGRTWTQEEKNILPKNSDNA

SFSSVVSYKEESGIPQVPYMTARIFRSDFTYSFPVSPGWKFLRLYFYPTSYKSGFDAVNS

FVSVTVNDFTLLQNFSADLTVKASIPESKSLIKEFIVPVYLTLNLTFRPSNNSLAFVNGI

EIVSMPDRFYSKGGFDDLITNVGSLIDFEIDNSTASETVHRLNVGGHMVDEVNDSGMFRR

WLSDDYEFLIGGVSPYMPDVNISYTEKTPAYVAPAYVYSTCRMMGNAQDTYLNLNFNLTW

LFTVDAGFSYLVRLHFFEKYLNKANQRVFSIFLGNQMAREEMDVIRLSGGPRIPIYLDFR

IYVGSESGPRPDLRLDLHPLVKDNPEYYEAILNGVEILKLNNSGNLAIIQDNELKPNPPL

SSNLTPNHVTQQIKGKSSHLLVKIFIAVGPGTGLATFVVVLMLWMRQMKRKNRKEERVVM

FKKLLNMYTYAELKKITKSFSYIIGKGGFGTVYGGNLSNGRKVAVKVLKDLKGSAEDFIN

EVASMSQTSHVNIVSLLGFCFEGSKRAIVYEFLENGSLDQFMSRNKSLTQDVTTLYGIAL

GIARGLEYLHYGCKTRIVHFDIKPQNILLDGNLCPKVSDFGLAKLCEKRESVLSLMDTRG

TIGYIAPEVFSRMYGRVSHKSDVYSFGMLVIDMIGARSKEIVETVDSAASSTYFPDWIYK

DLEDGEQTWIFGDEITKEEKEIAKKMIVVGLWCIQPCPSDRPSMNRVVEMMEGSLDALEI

PPKPSMHISTEVITESSSLSDGGEDV

>AT5G24010.1

MAFPINLTQTLLFFFCPLLHLSFAAFTPTDNYLINSGSNTNTSFFTTRSFLSDSSEPGSS

FLSTDRSISISDTNPSPDSPVLYNTARVFPVGGSYKFQVTTKGTHFIRLHFAPFKASRFN

LRSAKFRVLINGFSVINSFSTSSVVVKEFILKIDDPVLEISFLPFKASGFGFVNAVEVFS

APKDYIMDQGTKLVIPNSAQIFSNLSSQVLETVHRINVGGSKLTPFNDTLWRTWVVDDNY

LLLRAAARRAWTTHSPNYQNGGATREIAPDNVYMTAQEMDRDNQELQARFNISWGFQVDE

KRVLHLVRLHFCDIVSSSLNQLYFNVFINEYLAFKDVDLSTLTFHVLASPLYIDFVAESD

RSGMLRISVGPSDLSNPARVNALLNGVEIMRILSPVSSEVVSGKRNVVWIVVGSVLGGFV

FLSLFFLSVLCLCRRKNNKTRSSESTGWTPLRRFRGSSNSRTTERTVSSSGYHTLRISFA

ELQSGTNNFDRSLVIGVGGFGMVFRGSLKDNTKVAVKRGSPGSRQGLPEFLSEITILSKI

RHRHLVSLVGYCEEQSEMILVYEYMDKGPLKSHLYGSTNPPLSWKQRLEVCIGAARGLHY

LHTGSSQGIIHRDIKSTNILLDNNYVAKVADFGLSRSGPCIDETHVSTGVKGSFGYLDPE

YFRRQQLTDKSDVYSFGVVLFEVLCARPAVDPLLVREQVNLAEWAIEWQRKGMLDQIVDP

NIADEIKPCSLKKFAETAEKCCADYGVDRPTIGDVLWNLEHVLQLQESGPLNIPEEDYGD

VTDPRTARQGLSNGSNIERDYGDGTSGIISSTQVFSQLMTNAGR

>AT2G23200.1

MENFCFQDSVSLFITIMVLVLLPRLSLSDTSTYTRPENFYVNCGSDSNVFYGGQTFVGDT

NSSTNSVSFTNKGTEVINDQSSVAPEIYRTVRIFRHPSSYKFKLDSLGLHFVRLHFSVVF

SRADLLTARFTVSATSGSNHHLKSFSPQNLTNTPRVEEFLLMMNSLEFEIRFVPDHSSLA

LINAIEVFSAPDDLEIPSASDKNLHTIYRLNVGGEKITPDNDTLGRTWLPDDDDFLYRKD

SARNINSTQTPNYVGGLSSATDSTAPDFVYKTAKAMNRSSNEQVGMLMNVTWSFKVKSNH

RHFIRIHFSDILSNLSNSDSDFYLFVNGYWRVDVKPSEQPRLASPFFKDVVNVSDGSGLL

NISIGTKEANKDAGFLNGLEMMEVLSKSGSDYSNRSSSRVHIITGCAVAAAAASALVFSL

LFMVFLKRRRSKKTKPEVEGTVWSPLPLHRGGSSDNRPISQYHNSPLRNLHLGLTIPFTD

ILSATNNFDEQLLIGKGGFGYVYKAILPDGTKAAIKRGKTGSGQGILEFQTEIQVLSRIR

HRHLVSLTGYCEENSEMILVYEFMEKGTLKEHLYGSNLPSLTWKQRLEICIGAARGLDYL

HSSGSEGAIIHRDVKSTNILLDEHNIAKVADFGLSKIHNQDESNISINIKGTFGYLDPEY

LQTHKLTEKSDVYAFGVVLLEVLFARPAIDPYLPHEEVNLSEWVMFCKSKGTIDEILDPS

LIGQIETNSLKKFMEIAEKCLKEYGDERPSMRDVIWDLEYVLQLQMMTNRREAHEEDSTA

INSGGSLVAPRLMVSDSFSTNSIFQNGDESKNRFGFTDSSETRVFSQLKISDAR

>Hv*-*CrRLK1L1(XP_044984910.1)

MPALAILARSMVQCKRVPMFLILFILSITRVVTTDAIGSKVERFVPQDNYLLSCGASAAVQVDDGRTFRSDPESVSFLSTPTDIKITAKASLASASPLSPLYLDARVFSDISTYSFFISQPGRHWIRLYFLPITDSQYNLTTATFSVSTDSMVLLHDFSFIASPPNPVFREYLVSAQGDNLKIIFTPKKNSIAFINAIEVVSAPPSLIPNTTTRMGPQDQFDISNNALQVVYRLNMGGALVTSFNDTLGRTWQPDAPFLKLEAAAEAAWVPPRTIKYPDDKTLTPLIAPPSIYSTAQQMASTNITNARFNITWVMVAETGFRYLIRLHFSDIVSKTLNGLYFNVYINGMMAVANLDLSSLTMGLAVAYYKDLIAESSSIINSTLVVQVGPNTIDSGEPNAILNGLEIMKISNEASSLDGLFSPKTSSEVSKTTLTGIAFALAATAAFAVVICYRRNRKPAWQRTNSFHSWFLPLNSSSSFMSSCSRLSRNRFGSTRTKSGFSSVFASSAYGLGRYFTFIEIQKATKNFEEKGVIGVGGFGKVYLGATEDGTQLAIKRGNPSSDQGMNEFLTEIQMLSKLRHRHLVSLIGCCDENNEMILVYEFMSNGPLRDHLYGDTNIKPLSWKQRLEVCIGAAKGLHYLHTGSAQGIIHRDVKTTNILLDENFVAKVADFGLSKDAPSLEQTHVSTAVKGSFGYLDPEYFRRQQLTDKSDVYSFGVVLFEVLCARPAINPALPRDQVNLAEWARTWHRKGELGKIIDPNIAGQIRPDSLDMFAEAAEKCLADYGVDRPTMGDVLWKLEFALQLQEKGDVVDGASDGIPMKSLEMSNVDSMEKSGNAIPSYVQGR

>Hv*-*CrRLK1L2(XP_044974727.1)

MVLPTLPVTLIFLTLLAFLSIAMAADSNSMASGLILLNCGASGQGDDDIGRTWDGDISSKFAPLLEGVAANAAYEDPSLPSMVPYMTARIFTSNYTYSFPVTAGRVFLRLYFYPIAYGNYVVSDAFFGVTAGNLVLLNGFSASQTAQATSSAYLVREYSVNVSSGSLDLTFAPSTHQTGSYAFVNGIEIVPTPDIFTTADTKFVSGNHTDLFKFTADTGFQTMYRINVGGPYISPKDDSGFYRSWINDAPYRYDDSGVTFSKDDNVTIRYTPTVPNYTAPVDVYASARSMGQNPHVNPNFHLTHKLNYNLTWILPVDAGFFYLLRFHFCEIEYPITKVNQRVFFININNHAAQQKVDVILWSGGIGRTAYRDYAIMATGSSMVDLWIALEADFSDQPEFTDVILNGLEVFKLQGYGTNNLAGLNPPLPQKPSGARKYKGDKLAAIWGTTGGFALILIALTITCVISRQKKVGKSSFKTDCRHLNRPTECRESTCDLVRRFSFAEIQLVTKDFDEAFIIGRGGFGNVYSGEIDGRTKVAIKRFNQKSQQGFHEFQTEIEMLCNFRHRHLVSLIGYCEEKNEMILVYDYMAHGTFREHLYNTGNPPLPWQQRLEICIGAARGLHYLHTGTEQGIIHRDVKTTNILLDDRLMAKVSDFGLSKASPDIDNSHMSTVVKGTFGYLDPEYFRLQRLTKKSDVYSFGVVLFETLCARPVINTELPYEQVSLRDWALSCRKKGVLEEIVDPCVKEEITPQCFRTFAEIAEKCVADRSIDRPSMGDVLWNLEVALQLQDSASYNSSCAEGASSLQTSGVHSGKPSTNSTISVSAQEAIFSDIVHPEGR

>Hv*-*CrRLK1L3(XP_044950518.1)

MASPTLPVTLTCLTLLALLSLAMAADNNNSTGLILVNCGASGQEDDDSGRTWGEDTGSKFAPSLKGVAANAQRQDSSLPSTVPFMTARIFTSNYTYSFPVTPGRMFLRLYFYPTDYPNFAASDSSFSVSVSTPNLVLLDGFNASQTVQAISSAFLVREFSVNVSSGSTLDLTFAPSAHQNGSYAFVNGIEIVPTPDIFTAPDTRTVGDDTSPYTFDTAMAVQTMYRLNVGGQAISPKGDSGFYRSWTNDAPYIAGGSGVTFSKDDNLTITYTSKVPKYTAPADVYGSARSMGTTAQVNLNYNLTWILPVDAGFSYLLRFHFCEIQYPITKINQRSFFIYINNQTAQEQMDVIVWSGGIGRTTYTDYVTMAVGAGQVDMWIALHPDLSSKPEYFDAILNGLEVFKLQNNGSPNNLSGLNPPLPQRPPDAIPSAPSAGGKSVGAIVGAAVGGFAVLLVACFGVCIICKRKNNKKKKISKEPGGKSEDGHWTPLTEYSGSRSTMSGNTATTGSTLPSNLCRHFTFAELQTATKNFDQAFLLGKGGFGNVYLGEVDSGTKVAIKRCNPMSEQGVHEFQTEIEMLSKLRHRHLVSLIGYCEDKSEMILVYDYMAHGTLREHLYSTKNPPLSWKKRLEICIGAARGLYYLHTGVKHTIIHRDVKTTNILLDDKWVAKVSDFGLSKTGPNMDATHVSTVVKGSFGYLDPEYFRRQQLSEKSDVYSFGVVLFEVLCARPALSPSLPKEQISLADWALRCQKQGVLGQIIDPMLQGRIAPQCFVKFTETAEKCVADRSVDRPSMGDVLWNLEFALQLQESDEDTSSLTDGMLSSSGASPLVMTRLQSDEPSTDASTTTTSTTTMSMTGRSIASVDSDGLTPSTVFSQLMRPGGR

>Hv*-*CrRLK1L4(XP_044975236.1)

MAAAARHRPARARGALWIVSVLLVCVAAAYTPEDNYLVSCGSSLDTPVGRRLFLADDGGSGAVTLTSPRSAAVKASPDLVSGFRDAALYQNARVFSAPSSYSFAIKRRGRHFLRLHFFPFVYRSYDLAAAARAFKVSTQDAVLLEDGIPAPEPGNASSSPQPARVEFLLDVERDTLVVSFVPLIDGGIAFVNAVEVVSVPDNLVTDAAATTADSSSGRPELNPAALPLQTAYRVNVGGPVVAPDDDALWREWTTDQPLSDPRVDAVTREVRYNRTLNRLPGQATVTDAPDIVYATARELVITNISMDGQKQMAWQFDVDTRSSYFIRFHFCDIVGNASHQLRMNAYVDDATVKQDLDLAAIGNGALAFPYYTDFVLSASAASGKLAVHVGPRENKIVSPAAILNGIEIMKMHLSAGSVVVVEPAAKAAKSRLAVLLGSVCGAFAFVTIAVALAIVLRKKKNEKEEKEGDKEQPTPTQSQSSTPWMPLLGRFSVRSAIASGSSSFTTAGNTPGASPRAAAAAAAAVMPSYRFPLAMLQDATRNFDDSLVIGEGGFGKVYGAVLQDGTKVAVKRASPESRQGAREFRTEIELLSGLRHRHLVSLVGYCDEREEMILLYEYMEHGSLRSRLYGRSASPLSWAQRLEACAGAARGLLYLHTAVDKPVIHRDVKSSNILLDGDLTGKVADFGLSKAGPVLDETHVSTAVKGSFGYVDPEYCRTRQLTAKSDVYSLGVVLLEAVCARPVVDPRLPKPMSNLVEWGLHWQGRGELEKIVDRRIAAAARPAALRKYGETVARCLAERAADRPTMEDVVWNLQFVMRLQEGDGLDFSDVSSLNMVTELRPPRRQRNSVDCDGLDLSDVNSLKLVTEQTQPQTGSVEGDGVADDDFTDASMRGTFWQMVNVRSR

>Hv*-*CrRLK1L5(XP_044981625.1)

MRGPPRCALLLLLAAAALVPAARAQGAAAPAPSAGAAFVPRDDILLDCGATGKGNDTDGRQWDGDAGSKYAPPNLASASAGAQDPSVPQVPYLTARVSAAPFTYSFPLGPGRKFLRLHFYPANYSNRDAADAFFSVSVPAAKVTLLSNFSAYQTITALNFAYLVREFSVNVTGQNLDLTFTPEKGRPNAYAFVNGIEVVSSPDLFDLATPLFVTGDGNNQPFPMDPAAALQTMYRLNVGGQAISPSKDSGGARSWDDDTPYIYGAGAGVSYQNDPNVTITYPDNVPGYVAPSDVYATARSMGPDKGVNLAYNLTWILQVDAGYQYLVRLHFCEIQSPFTKPNQRVFSIYLNNQTAMKGADVILWADPNGIGTPVYKDYVVSTVGSGTMDFWVALHPDVETKPQYYDAILNGMEVFKLQLTNGSLVGLNPLPSPDPPVNSGSGNKKSIVVPIVGGVVGGLAVLALGYCCFICKRRRKAAKASGMSDGHSGWLPLSLYGHSHTSSSAKSHATGSYASSLPSNLCRHFSFAEIKAATKNFDESRILGVGGFGKVYHGEIDGGTTKVAIKRGNPLSEQGIHEFQTEIEMLSKLRHRHLVSLIGYCEEKNEMILVYDYMAHGTLREHLYKTQNAPLSWRQRLEICIGAARGLHYLHTGAKHTIIHRDVKTTNILLDEKWVAKVSDFGLSKTGPSMDHTHVSTVVKGSFGYLDPEYFRRQQLTEKSDVYSFGVVLFEVLCARPALNPTLAKEEVSLAEWALHCQKKGILDQIVDPYLKGKIVPQCFKKFAETAEKCVADNGIERPSMGDVLWNLEFALQMQESAEESGSIGCGMSDEGTPLVMVGKKDPNDPSIDSSTTTTTTTSLSMGDQSVASIDSDGLTPSAVFSQIMNPKGR

>Hv*-*CrRLK1L6(XP_044983157.1)

MAVHVLLPLLLLLLVPTVLPYIALAASSPDFTIFLACGAGANISFPSDNPARTFVPDAGYFSPARAPAVSAGASSSAASPLYAAARAGSSDFSYRLTYPDTAGASSFLVLRLHFFPFVPASSSTSLSSARFTVSVLDAYALLRNFSPPADGIVKEFFLPRGRSGDFTVRFSPDAGSSAFVNAVELFPAPPELLWNGSTSVVPVGVLEGTGLPQWQLAALETVYRLNVGGPMVTRENDTLWRTWLPDGPYLFGAPGQSVVNNTSSPIIYNPPTTREVAPDVVYRTQRAANVTDLMRATTPGLNFNVTWTFPAEAGSRYLVRLHFCDYEVVSSVVGVGIVFNVYVAQALATLDLSPEDRARQPNEAFYVDYAAMAPRAGNLTVSIGWSPKSSGGGILNGLEIMKLQSANLSSPRPHGLTKKTIIVIVLATVLGAAVLACAVLCFFVVLRRKRRQVAPPASTEDKESTQLPWSPYTQEGVSGWADESTNRSSEGTTARMQRVSTKLHISLAEVKAATDNFHDRNLIGVGGFGNVYKGALADGTPVAVKRAMRASKQGLPEFQTEIVVLSGIRHRHLVALIGYCNEQAEMILVYEYMEKGTLRSHLYGSDEPTLSWKQRLEICIGAARGLHYLHCGYSENIIHRDVKSTNILLGTDDHGGGSASGGAAIIAKVADFGLSRIGPSLGETHVSTAVKGSFGYLDPEYFKTQQLTDRSDVYSFGVVLFEVLCARPVIDQSLDRDQINIAEWAVRMHGEGKLDKIADARIAGEVNENSLRKFAETAEKCLAEYGADRPSMGDVLWNLEYCLQLQETHVNRDAFEDSGAVATQLPADVVVPRWVPSSTSMLMMDDADETGLSMTEIADSQVFSQLNARGEGR

>Hv*-*CrRLK1L7(XP_044970778.1)

MAVHGRLLVLLLATVLPRAALAALSPGFQFQFFLACGANYSVSFPSNDFPTNTFVPDDAYLSPASAPAVSARFTPYSRPALHAAARADISAFSYRFPSPASPYTPPFVVLRLHFFPFFQATSSQYVINIFSARFNVSVSGHEYALMSSFSPPVNGAVKEFFVPRDLSGGDFHVTFTPDAGSSAFVNAIELFSAPLEMLWCGSVTPVGAVVKDDMDLWQRQPLETVYRLNVGGPEVTIENDTLWRTWLPDGPYLYDARGKSVVSNTSSPIIYDTSNGYTREVAPDVVYQTQRMANVTDWLLATTPGLNFNLTWTFPAVKGSRYLVRLHFCDYEVVSSVVGVSIVFNVYIAQAIGTPDLMPNDRATVSNKAFYMDYAAKAPSTGNLTVSIGLVLKSNGGGILNGLEIMRLPPVDLSSRRSNGQTKRTVLITVAVVLGAAVLACVALCLFGVPYTKYSASGWAEQWTNRWFGEGETSGMESVSRKLHIPLAKIKAATDSFHERNLIGVGGFGNVYKGVLSDGTPVAVKRAMRASQQGLPKFQTEIVVLSGIRHQHLVSLIGYCNEQAEMILVYEYMEKGTLRSHLYGSDEPALSWKQRLEICIGAARGLHYLHRGYAENIIHRDVKSTNILLGSDGGSTGGVITKVADFGLSRIGPSFGETHVSTAVKGSFGYLDPGYFKTQQLTDRSDVYSFGVVLLEVLCARPVIDQSLDHSMINIAEWAMRMRREGRLDKMADPRIAGEVDEESLLKFVETAEKCLADCWVDRPSMGDVLWNLEYCMQLQEMNVIGDEHDNMVPSSTSLLLDDTGLSMTNVADNKVSPARARDEAR

>Hv*-*CrRLK1L8(XP_044981971.1)

MPPLVDMLLLASVLLGAASSAVAGADNYLVVCGTSASATVAPGRTFAGDARLPAKSLAAPQSVEANTSLTAAVPSGESELYRSARVFTAPASYTFAVKQPGRHFVRLHFFPFAYQSYDMAADAAFNVSVQGAVFLNGYTNKNGTAELREFSVNVTGATLVIAFTPTGKLAFVNAIEVVPLPDELIADTASMVDRAVQYTGLSTQALETIHRINMGIPKITPGNDTLGRTWLPDQGFQLNANLAQHKDAKPLTIKYDEKSALSSAFTAPAEVYATATKLSTAGEVSTINVQFNISWRFDAPAGSDYLLRFHFCDIVSKAAIGMAFNVYVGGSVVLKNYEISRDTFNRLSIPVYKDFLLGAEDAKGTITVSIGSSTDDNALPDGFLNGLEIMRLVGSAGAGAAAASPRSSKVKIGIIAGSAVCGATLIMVLGFIAFRTLRRTEPEKKPSDTWSPFSASALGSRSRSRSFSKSSGNTVMLGQNGAGAGYRIPFAALQEATGGFDEGMVIGEGGFGKVYKGTMRDETLVAVKRGNRRTQQGLHEFHTEIEMLSRLRHRHLVSLIGYCDERGEMILVYEYMAMGTLRSHLYGAGLPPLSWEQRLEACIGAARGLHYLHTGSAKAIIHRDVKSANILLDDTFMAKVADFGLSKNGPELDKTHVSTKVKGSFGYLDPEYFRRQMLTEKSDVYSFGVVLLEVLCARTVIDPTLPREMVNLAEWATPCLRNGQLDQIVDQRIAGTIRPGSLKKLADTADKCLAEYGVERPTMGDVLWCLEFALQLQMGSSDGSETDTMLPPPVPGKTPLVQRSLSTATVPTDDAAMTTNLGDLEGMSMSGVFSKMIKSDEVR

>Hv*-*CrRLK1L9(XP_044973269.1)

MRGGPRCALLLLLLAACAALVPAAWAQGGGDAPAPAAPFVPRDDILLDCGATGKGDDTDGRQWAGDAGSKYAPPNLASAAAGAQDPSVPQVPYLTARVSAAPFTYSFPLGPGRKFLRLHFYPANYSDRNAADAFFSVSVPAAKVTLLSNFSAYQTTTALNFAYIVREFSVNVTGPTLDLTFTPEKARRNAYAFINGIEVVSSPDLFDLATPFFVTGDANNQPFPMDPGAALQTMYRLNVGGQAISPSKDSGGARSWDDDTPYIYGAGAGVSYPNDPNITITYPDNVPGYVAPLDVYATARSMGPDKDVNLAYNLTWIVQVDAGFTYLVRLHFCEIQSPITLPNQRVFNIYLNNQTAQTGADVIQWVDPKSIGTPVYKDYVVSTVGSGIMDFWVALHPDTGNKPQYYDAILNGLEVFKLQLSNGSLAGPNPVPSADPPVHTGQGKKSSLVGPIAGGVIGGLALLALGYCCLICKRRRKTAKDTGMSDGHSGWLPLSLYGNSHTSSSDKSHATGSIASSLPSNLCRHFSFAEIKAATKNFDESRILGVGGFGKVYQGEIDGGTTKVAIKRGNPLSEQGIHEFQTEIEMLSKLRHRHLVSLIGYCEDKNEMILVYDHMAHGTLREHLYKTQNAPLSWRQRLEICIGAARGLHYLHTGAKHTIIHRDVKTTNILLDEKWVAKVSDFGLSKTGPSMDHTHVSTVVKGSFGYLDPEYFRRQQLTEKSDVYSFGVVLFEVLCARPALNPTLAKEEVSLAEWALHCQKKGILDQIVDPYLKGKIVPQCFKKFAETAEKCVADNGIERPSMGDVLWNLEFALQMQESAEESGSFGCGMSDEGTPLVMPGKKDPNDPSIDSSTTTTTTTSISMGDQSVASIDSDGLTPSAVFSQIMNPKGR

>Hv*-*CrRLK1L10(XP_044967870.1)

MLQMRLLVLAAVSIVFANLQFLKAHGRELFLSCGSNATADADGRRWIGDMAPDLNFTLSSPGIAALLAGGSNGSEIMAPVYRSARFFTTTSWYDFSLLPGNYCVRLHFFPSTFRNFSASSSVFDVVANEFKLVSKFNVTEEIVWRNSVSNSAATALVKEYFLAVNTSRLQIEFDPRPGSFAFVNAIEVVLAPDNSFNDTVNKVGGVDVHIPPELSGRAVETMYRLNIGGPALASSHDQHLHRPWYTDEAFMFSANAALTVSNTSAIKYVSSNDSSIAPIDVYETARIMGNNMVMDKRFNVTWRFFVHPNFDYLVRLHFCELVYDKPSQRIFKIYINNKTAAENYDVYDRAGGINKAYHEDYFDSLPQQVDSLWLQLGPDSMTSASGTDALLNGLEIFKLSRSGSLDYVLGHIDVGNKRGRSKGRSRIGLWEEVGIGSAAFVVLASVALFSWCYVRRKRKAVDEEVPAGWHPLVLHEAMKSTTDARASKKSPLARNSSSIGHRMGRRFSIADIRAATKNFDESLVIGSGGFGKVYKGEVDDGITVAIKRANPLCGQGLKEFETEIEMLSKLRHRHLVAMIGYCEEQKEMILVYEYMAKGTLRSHLYGSGLPPLTWKQRIDACIGAARGLHYLHTGADRGIIHRDVKTTNILLDKNFVAKIADFGLSKTGPTLDQTHVSTAIRGSFGYLDPEYFRRQQLTQKSDVYSFGVVLFEVACARPVIDPSVPKDQINLAEWAMRWQRQRSLEAIADPRLDGDYSPESLKKFGDIAEKCLADDGRTRPSMGEVLWHLEYVLQLHEAYKRNVDCESFGSSELGFADMSFSMPHIREGEEEHHPKKSDIREDSAP

>Hv*-*CrRLK1L11(XP_044966102.1)

MGTTSEQKIALLLLGTIWVLLGTCNAEFTPADNYLINCGSTVDVHVPGQGFFRADNSGSTILKSDHNVAANTLPDAVISSDNPVLYQTARIFSVPSSYSFNMKSRGRHFVRLHFFGFRYQSYDLAVAKFKVSTQDVVLLDNFTPPSNSSPLVREYSLNITEDKLILTFVPLGNSTSFINAIEVISVPDDLIRDSAQTVNPSGQYLGLTTQSFQTFYRINVGGREVTAANDTLSRSWDTDQNFFLNSTTTELFAYQAKLNYQKGAATKEDAPDSVYNTARRFAVQNRTSLVSNMTWQFDVDGSSSYLIRFHFCDIVSKAAYSLYFDIYVDGRLALENVDLSERVLGTLAVPYYMEFVLKSSDPSGKLSVGIGPSSLNNVAPDGILNGLEIMKMDISTGTVYVVWPPGTPNRKLAIILGTVLGGVGAVSIAIILCFVLRRKKKEKKPRRAPTSRPSSSWSPLTLNGLSFLSTGTRTTSRTTLTSGTNSDASYRIPFALLQVATKHFDEQMVVGVGGFGKVYKAVLQDSTKVAVKRGNQKSHQGLREFRTEIELLSGLRHRHLVSLIGYCDEQNEMILVYEYMEKGTLKSHLYGSDMPPLSWKKRVEICIGAARGLHYLHTGFAKSIIHRDVKSANILLDENLMAKVSDFGLSKTGPELDQTHVSTAVKGSFGYLDPEYYRRQKLTDKSDVYSFGVVLLEVICARPVIDPTLPRDMINLAEWAIKWQKRGELGQIVDQRIAGTIRPESLRKYGETVEKCLADYGVDRPTMGDVLWNLEFVLQLQEAGPDVSNVDSMNQISELPPDTRRMGSLEIGTADESPTNMDYSQMSTNDAFSQLMNTEGR

>Hv*-*CrRLK1L12(XP_044983735.1)

MAAARGRGVLLAVLLAVALALVSVGADIYKPTDSILVDCGSDKEGQDEDGRKWTSDKDSKFLPDGGKSSITATADINDPSLPSSVPYMTARVFPKETAYTFPVSDADRHWVRLHFYPASYHDIPADHFFFSVTTSTGVTLLRNFSVYVTAKALTQGYIVREFSLPPSTTGSLSLKFTPTAMNNASYAFVNGIEILSMPNIFADPAQLVGLGDQTLDASAGNMQTMYRLTAGGSYIPATKDSGLSREWFPDTPYIYGAATGVTYEANDTVPIKYPSPAAEYLAPQSVYDTSRHMGRDGNVNRVNNLTWAFEVDGNFTYLVRLHFCSLMEDKINQVVFSILVNNKTATTTGSADIIAWAKEKNPDSPGKGVPVIRDYAVFTAAAPAGADSVLWVTLRPDLTTTPQFVNAFLNGLEVFKTSDASSNLAGPNPDISKMLAEAEAEADDVEGEFRERASNVGALIGGAAGGAAAFGLVAGLCFVAYQSKKRKELSNSRSHSSSGWLPVYGGGGNSQTSVSKSSGGRSAVTLNPNITAMCRHFSLQEIKSATKGFDESLVIGVGGFGKVYRGVVDGDTKVAIKRSNPSSEQGVLEFQTEIEMLSKLRHKHLVSLIGCCEDNGEMILVYDYMGHGTLREHLYKSGKPPLLWRQRLEILIGAARGLHYLHTGAKYTIIHRDVKTTNILVDDKWVAKVSDFGLSKTGPTVQNQTHVSTMVKGSFGYLDPEYFRRQKLTEKSDVYSFGVVLFEVLCARPALNPSLPREQVSLADHALSCQRRGTLEEIIDPVLEGKVAPDCLKKFAETAEKCLSDQGVDRPSMGDVLWNLEFALQMQDTFDNGGKPPEVDDYSSSFTITPPSMEESLAANAAALSLISEDMDEEDIANSVVFSQLTHPTGR

>Hv*-*CrRLK1L13(XP_044958511.1)

MVRRGALPLALLAALATLTAVSGQGKPVTDNGAGSSQAKFTPKDAFYIDCGGTAAADTKDGKSFKTDAEANNLLSAKDAIKVADDKADVPSHLYRTARVFKAEAIYNFPLTAPGWHFIRLYFFPIKSGEADLAAATFDVTTAANVLLHSFTAEAKAVMKEYVINATENKLELKFTPASGSAAFINAIEVVNAPDQLLNDKALTVSPLAEISGLSEAAYQVVCRLNVGGPVIGPVNDTLGRQWDEDGPYLNPKEAGVEVSVSPNVIKYPETYPVSKLVAPTAVYTTARHMANSGVANQNFNVSWKVDVDASFDYLVRLFFADIISDTMNDLYFNVYINERKAISGLDLSTITGDMAAPFYKDFVVNSSVDTDGHIIFAVGPMGQDTGRIDALLNGAEVLRISNSVGSLDGEFGVDGRMVDDGSGTRKVVAAVGFAMMFGAFAGLGCMVVKWHRRPQDWERRNSFSSWLLPIHTGQSFTNGKGSKSGYTVSSTAGLGHFFSFAEMQEATKNFDDSAIIGVGGFGNVYVGEINDPDEEGSRIKVAIKRGNPSSEQGINEFNTEIQMLSKLRHRHLVSLIGYCDENEEMILVYEFMQHGPFRDHIYGGPEGLPTLSWKQRLEICIGAARGLHYLHTGTAHGIIHRDVKTTNILLDEKFVAKVADFGLSKDGPGMNQLHVSTAVKGSFGYLDPEYFRCQQLTDKSDVYSFGVVLLETLCARAPIDPQLPREQVSLAEWGMQWKRKGLIEKIMDPNLAGKVNPESLAKFAETAEKCLCEFGSDRLSMGDVLWNLEYALQLQEANPPEGATDADDADASIVSSSSGVSTVPDASTTSANELFAQLADMKGR

>Hv*-*CrRLK1L14(KAE8784173.1)

MPAAGRSGGPGQVNIMTGRRKLHVVTLAILCFWSSAGVCKAQTVDFKPADSYLVDCGSAKGTTVLGRNFAADGASPVTVSTSQDILAGTSANGVSSFDNPVLYQTARIFTSPSSYTFPIQKQGRHFVRLYFYPFIYQTYDLSTAKFTVSTQDVLLLSDFQQPDKTAPLFKEYSLNITRDQLVISFKPSNGIAFINAIEVVSVPDDLIADVANMVNPVQQYSGLTTQSLETVYRVNMGGPKVFPSNDTLSRTWQKDQKYILNPSVTKTAQYGKAINYRNGGATPLTAPDIVYSTATELAASNTSNALFNMTWQFDVDAGFSYLIRFHFCDIVSKALNQLYFNAYVGGFFAQHDLDLSEQSVNQLATAIYVDVVLSSNDASSKLSISIGPSTLNNALPDGILNGLEIMKMGSGSGSAFTVGNNGSNKKLPIIIGSVLGVVGLLIIILVVVLLCRRKKTDDKQHSKTWMPFSINGLTSLSTGSRTSYGTTLTSGLNGSYGYRFAFNVLQEATNNFDENWVIGVGGFGKVYKGALRDDTKVAVKRGNPKSQQGLNEFRTEIELLSRLRHRHLVSLIGYCDERNEMILVYEYMENGTVKSHLYGSDNPSLNWKQRLEICIGAARGLHYLHTGSAKAIIHRDVKSANILLDENLLAKVADFGLSKTGPELDQTHVSTAVKGSFGYLDPEYFRRQQLTEKSDVYSFGVVMLEVLCARPVIDPSLPREMVNLAEWGMKWQKRGELHQIVDQKLSGAIRPDSLRKFGETVEKCLADYGVERPSMGDVLWNLEYVLQLQDADSSTVSDVNSMNRIVDLSSQVQHVGAMESISVTMAEDGASHEPDHDLSDVSMSRVFSQLIKAEGR

>Hv*-*CrRLK1L15(XP_044966011.1)

MESSAKFLLMLVAFLTAGNARAQPQPVLINCGSDSATNADARIWIGDSSPSSNFTLSFPGAVATAAPGGQDPYGDLYKTARLFNASSSYRLAVAPGSYFLRLHFSQLFANPGAQEPIFSVAANGLKLLSKFSVHGEISWRDSQINSTSSVIVKEYLLNVTSGKLGIEFTPDEGSFAFINAMEVLPVSGTPIFDSVNKVGAHGLKGPLSLDRGGIETMYRLCVGCIDVLPRKEDPGLWRKWDSDEHFIFSLNAARSIFNSSNISYVSADDPTLAPLRLYQAARVPTESSVLGKKFNVSWSFNIDPGFDYLVRLHFCELEYDKAEQRKFKIYINNKTAAEGYDVFARAGGKNKAFYEDFLDAASPQMDTLWVQLGAESSAGSAAADALLNGMEIFKVSRDGNLAHPTVRIGGISGGVSKPKRSPKWVLIGAASGLIFFIAIIGAVYFCFNLQRKKNSSANKAKDNLHGATHTRSPTLRTAGAFGSNRMGRRFTIAEIRTATLNFDESLVIGVGGFGKVYKGKMEDGTRVAIKRGHTESHQGQGVKEFETEIEMLSRLRHRHLVPLIGYCDEQNEMVLVYEHMANGTLRSHLYGSDLPALTWKQRLEICIGAARGLHYLHTGLDRGIIHRDVKTTNILLDNNLVAKMADFGISKDGPALDHTHVSTAVKGSFGYLDPEYYRRQQLTPSSDVYSFGVVLFEVLCARSVINPTLPRDQINLADWALNRQRHKLLETIIDLRLEGNYTLESIKKFSEIAEKCLADEGVNRPSMGEVLWHLESALQLQQGHPQSTNGDDCSDSQAQPSDVPIRIKEAEQSTRPGSHDSDGQVVDVKIEVP

>Os*-*CrRLK1L1

MLSLQKPSKTPFVPASSALFNPESIFLPSHERSPSSCYRPMLLIPTLAKMARSMLGWKRV

PLFSILLILSITNIATTYAIASQADRFVPRDNYLLSCGAPAAVQLDDGRTFRSDPDSASF

LSTPVDIKITAKNSLASGAPSSQLYLTSRVFSDISTYSFFISQPGHHWIRLHFLPIPDDH

YNLTTATFSVSTDDMVLLHDFSFIATPPNPVLREYIVATQGDTLKIIFTPKKDSIAFINA

IEVVSAPPSLIPNTTTGMAPQGQLDISNNALQVVYRLNMGGPLVTAFNDTLGRIWLPDAP

FLKLQAAANAAWVPPRTIKYPDDKTNTPLIAPANIYSTAQQMASTNTSDARFNITWEMVT

EPGFSYFVRLHFCDIVSKALNSLYFNVYINGMMGVLNLDLSSLTVGLAVPYYRDFIIDSS

SIINSTLIVQIGPGTTDTSNPNAILNGLEIMKISNQENSLDGLFSPKRSSQLGKKTMTGI

GLAMAVMAAALAVVMCCRRRHRPGWQKTNSFQSWFLPLNSTQSSFMSTCSRLSSRNRFGS

TRTKSGFSSIFASSAYGLGRYFTFVEIQKATKNFEEKAVIGVGGFGKVYLGVLEDGTKLA

IKRGNPSSDQGMNEFLTEIQMLSKLRHRHLVSLIGCCDENNEMILVYEFMSNGPLRDHLY

GGTDIKPLSWKQRLEISIGAAKGLHYLHTGAAQGIIHRDVKTTNILLDENFVAKVADFGL

SKAAPSLEQTHVSTAVKGSFGYLDPEYFRRQQLTEKSDVYSFGVVLFEVLCARPAINPTL

PRDQVNLAEWARTWHRKGELNKIIDPHISGQIRPDSLEIFAEAAEKCLADYGVDRPSMGD

VLWKLEFALQLQEKGDIVDGTSNQFPMKSLEVTSGDSMEKSGNVVPSYVQGR

>Os*-*CrRLK1L2

MMHPSLLATTQWVTLSTLLSIAIAADNYFSSSSPIFLNCGASAMQLDSNNRSWDGDTSST

FAPSVKGLAARASYQDPSLPSLVPYMTSRIFISNYTYSFPVIPGRMFVRLHFYPVAYGNY

ASRDAYFGVTTNNLTLLDNFNASQTALAAKYAYILREFSLNVTSGSLDLTFFPSTQNGSY

AFVNGIEIVPTPDIFTTLSPIPPTNGNPDPSDIDSMISFQTMYRLNVGGMTISPQGDSMF

YRSWENDSPYIYGSAFGVTFSKDSNVTITYPSTMPNYIAPADVYGTARSMGPIAQINLHY

SLTWILPVDAGFYYLLRFHFCEIEYPITKVNQRSFFIYINNQTVQEQMDVIVWSGGIGIT

TYTDYVIVTVGSGQMDLWVALHPDLSSGPEYYDAILNGLEVFKLQDIGKKSLAGLNPPLP

PQPKSDVNPKGVSGGGKSKGAVPASIRGAMGSTATMLIACFSVCIICRLKKVAKHSFMTD

KKCMTYRTEFYHSPSNLCRNFTFDEIQVATRNFDESLLLGRGGFGDVYRGEIDNNGENVA

IKRSNPLSVQGVHEFQTEIELLSKLRYCHLVSLIGYCKEKNEMILVYEYMAQGTLREHLY

NSNKPSLPWKQRLKICIGAARGLHYLHMGANQTIIHRDVKTANILLDDKWVAKVSDFGLS

KANPDIDSTHVSTVVKGTFGYLDPEYYRRKQLTQKSDVYSFGVVLFEILCARPAVNIELP

EEQASLRDWALSCQKKGMLGKIIDPHLHGEISPPCLRMFADCAKQCVADRSIDRPLMSDV

LWSLEAALKLQENAENNKKFSEATTSSKRTPDLITIMGTDKPSTYSTMSITGQKIIFSDM

MHPQGR

>Os*-*CrRLK1L3

MMHPSLLATIQWLTLSALLSIAMAADNNSTASAPIFLNCGASGVQPDSYNRSWDGDASSK

FAPSVKGNVARASYQDPSLPSPVPYMTARFFTSNYTYSFPVSPGRMFVRLHFYPTNYNGN

LDSANAYFGVTTNNLILLDNFNASQTALATSSAYFFREFSVNVTSSSLKLTFAPSTRNGS

YAFVNGIEIVPTPDIFTTPTPTSANGGDNVQYGIDPVMGLQTMYRLNVGGQPISPQGDSG

FYRSWDNDSPYIYGAAYGVTFSKDGNVTIKYPNTEPNYTAPVAVYATARSMGPTAQINLN

YNLTWILPVDAGFTYLLRFHFCEIQYPITKVNQRSFFIYINNQTAQNQMDVIVWSGGIGR

TTYTNYVVTTVGSGQTDLWVALHPDLSSKPEYFDAILNGLEVFKLQDLGRNNLAGLNPPL

PPKPGVNPNGGSSRGKSKSVAPAAIGGAVGGLAVLLIACVGLCIICRRKKKVAKDTGKSD

EGRWTPLTDFTKSQSATSGKTTNTGSHSMLPANLCRHFSFAEIQAATNNFDKSFLLGKGG

FGNVYLGEIDSGTRVAIKRGNPLSEQGVHEFQNEIEMLSKLRHRHLVSLIGYCEDRNEMI

LVYDYMAHGTLREHLYNTKNPPLSWKQRLEICIGAARGLYYLHTGAKQTIIHRDVKTTNI

LLDDKWVAKVSDFGLSKAGPNVDNTHVSTVVKGSFGYLDPEYFRRQQLTEKSDVYSFGVV

LFEVLCARNALSPSLPKEQVSLADWALRCQKKGVLGEIIDPLLKGKIAPQCFLKFAETAE

KCVADRSVDRPSMGDVLWNLEFALQLQESTEDSSSLTEGTSASTSPLVVARLHSDEPSTD

VTTTTTTTTSLSITDRSIASVESDGLTPSNIFSQLMTPDGR

>Os*-*CrRLK1L4

MIKLRSALGVLEILSVLCISLVAAYTPVDNYLISCGSSVDTPVGQRLFVADDSGTVVLTS

PASDAVKASPSAVSGLRDDAAMYQSARVFKAPSSYSFRIRDPGRHFVRLHFFPFVYLGYD

LATASFKVSTQDAVLLDGFAPAAAARGNASTTTTTATAAAVCEEFLLDVARDTLVVTFVP

LAGRLAFVNAIEVVSVPDDLIGAADSSLSTSDSTGQQLNPAVMPLQTVYRVNVGGQAVAP

DSDTLWREWTSDQQLLVGPAMTKGVSYNRTPNYLPGQATANDAPAIVYATGRELIIMTNS

TDDGMKQMAWQFDVGRSASYLIRFHFCDIVSSVPGRLHMNAYVDSSNAIQDLDLSAIGNG

TLAFPYYRDFVLAASTPSGKLAVYVGSTSQKITTPAAILNGLEIMRILTTAGNVAVVEPT

MPPGTKKKNNLAVVLGSVCGAFGFVSVAAALVIVLRRKEEKEELRTPTTSQPSTAWMPLL

GRISFRSAPPSAVGSRSPSFTIDTNANTPGGGATPGMAAAASSSPSYRFPFAALQDATGN

FDEGLVIGEGGFGKVYAAVLQDGTKVAVKRANPESRQGAREFRTEIEMLSGLRHRHLVSL

IGYCDEQDEMILLYEYMEHGSLRSRLYGGGAATATATALSWAQRLEACAGAARGLLYLHT

ATAKPVIHRDVKSSNILLDDGLTAKVADFGLSKAGPDMDETHVSTAVKGSFGYVDPEYVR

TRKLTAKSDVYSFGVVLLEALCARPVVDPRLPKPMVNLVEWGLHWQRRDELEKIVDRRIA

GTVRPAALRKYGETVARCLADRGADRPAMEDVVWSLQFVARLQEVDGLDASDVSSLNMVH

QLMPPTSLHARQRSAGESETGRTDADEDSSVVDDDYTDASMRGIFWQMVNVRGR

>Os*-*CrRLK1L5

MGSSRFVLLLLLLLAVAACVARGQGGGNSSSAAAPAPAAGAGPFVPRDDILLDCGATGKGNDTDGRVWSGDAGSKYAPASLGSASAAGQDPSVPQVPYLTARVSAAPFTYSFPLGAGRKFLRLHFYPANYSSRDAADARFSVSVPAANVTLLSNFSAYQTATALNFAYIVREFSVNVTTPTMELTFTPEKGHPNAYAFVNGIEVVSSPDLFDISTPNLVTGDGNNQPFPIDAGTALQTMYRLNVGGQAISPSKDTGGYRSWDDDSPYVFGAAFGVSYPKDDNVTIAYPSNVPEYVAPVDVYATARSMGPDKNVNLAYNLTWIMQVDAGFTYLVRLHFCEIQYPITMINQRVFNIYINNQTAFQGADVIAWTNNNGIGSPVYQDFVVTTVGSGAMDLWVALYPDVQAKPQYYDAILNGLEVFKLPLSNGSLAGLNPVPTVEPSLDGGAVKKSSVGPIVGGVIGGLVVLALGYCCFMICKRRSRVGKDTGMSDGHSGWLPLSLYGNSHSSGSAKSHTTGSYASSLPSNLCRHFSFAEIKAATNNFDESLLLGVGGFGKVYRGEIDGGVTKVAIKRGNPLSEQGVHEFQTEIEMLSKLRHRHLVSLIGYCEEKNEMILVYDYMAHGTLREHLYKTKNAPLTWRQRLEICIGAARGLHYLHTGAKHTIIHRDVKTTNILLDEKWVAKVSDFGLSKTGPSMDHTHVSTVVKGSFGYLDPEYFRRQQLTEKSDVYSFGVVLFEVLCARPALNPTLAKEEVSLAEWALHCQKKGILDQIVDPHLKGKIAPQCFKKFAETAEKCVSDEGIDRPSMGDVLWNLEFALQMQESAEDSGSIGCGMSDEGTPLVMPGKKDPNDPSIESSTTTTTTTSISMGDQSVASIDSDGLTPSAVFSQIMNPKGR

>Os*-*CrRLK1L6

MLCFEPNKSVVSGRGGIGKCDDPISVHSIVHSTIPVFHHALPSGRCPIFLRVHFPVPDST

HHSTASHSSFPLPSNTTTRTVRTLVRFRLILSWTRQWRHDHASTTASMATVIVILLLLPL

LPSTALAAFPYFLACGAASNVSFPGDSPARTFVPDAPFLSSAGRVPAVTSTGSNTIPPLY

AAARAAGSGFSYSFADPDTATVNVSRVLRLHFFPFTSSSSVNLSSASFSVSVRDAYTLLS

SFSPPRDGVVKEYFVPGDGSGEFRVKFTPDAGSTAFVSAIELFPAPPELLWRRPVKPVGA

LVDSVDVNAWPQQALETVYRLNVGGSKVTAANDTLWRTWLPDDPYFSSPRGLSQVNSTST

PIIYGTSIGYTREVAPDSVYKTQRAMNMASQQLFLTPGPFNLTWTFALPPPAPGSDSDYL

VRLHWCDYSLVSSVVATGIVFDVYVAQRLASKDLDRNAADAAEQPNEAFYLDYAATAPTT

GNLTISIGKSDKSDAGGMLNGLEIMKLRRADNLNSAGSHGRRKKILIGTLSAALGVAVLA

CALLCLLAVLRRRRQAPTPAPEEKESTQLPWSQHTQDGSSWVDMSNASGAGMTGGLHRMS

MQLNISLADITAATENFNERNLIGVGGFGNVYSGVLRDGTRVAVKRAMRASKQGLPEFQT

EIEVLSRIRHRHLVSLIGYCNEQSEMILVYEYMEKGTLRSHLYGSEEPPLSWKQRLEICI

GAARGLHYLHTGYSENIIHRDVKSTNILLGDAFIAKVADFGLSRIGPSFGETHVSTAVKG

SFGYLDPEYFKTQQLTDRSDVYSFGVVLFEVLCARTVIDQSLERDEINLAEWAVSLQQKG

ELAKITDPRIAGQVNGNSLRKFAETAEKCLADYGLDRPSMGDVLWNLEYCLQLQETHVNR

DAFEDSGAVATQFPADVVVPRWVPSSTSFLMDDSVTDSGIANSKAFSQLSSGDGR

>Os*-*CrRLK1L7

MATVLEMLVQLAVVVTVLCAAVRAYTPADSYLFLCGTSGNATVDGRTFVGDAGLPASVLM

APQSTEANMPANQVTGAGDDSPALYQSARVFTAPANYAFSAKPGRHFVRLRFFPFRYQSY

DLAADAAFNVSVQGVVFVDGYTPKNGTAVVREFSVNITGRALVIAFTPTGKKVAFVNAIE

VVSHPDELIGDTAPMVNPRNQSQYTGLTAKALETVHRINMGEPKVTPNNDTLWRTWLPDW

TFLHESSFAAHNQVSPAMIKYQSGYATSLTAPSAVYTTVTELNTTAAMVGNTQAQLNLTW

KFNAPAVSDYLLRLHLCDIVSKATLGVVFNVYVGQWRVLQDYESSGDTFSLLATPLYKDF

VLAASDAAKGTITVSIGSSTATNALPGGFLNGLEIMRIVGSTGSIDGATSPRGSKIKTGI

IAGSAVGGAVLAIALGCVAVRMLRRKKKPVKQPSNTWVPFSASALGARSRTSFGRSSIVN

VVTLGQNGAGAGAGYRFPFAALQEATGGFEEEMVIGVGGFGKVYRGTLRDGTQVAVKRGN

RLSQQGLNEFRTEIELLSQLRHRHLVSLIGYCDERGEMILVYEYMAKGTLRSHLYGSDLP

PLPWKQRLEACIGAARGLHYLHTGSAKAIIHRDVKSANILLDDGFMAKVADFGLSKTGPE

LDKTHVSTAVKGSFGYLDPEYFRRQMLTEKSDVYSFGVVLLEVLCARAVIDPTLPREMVN

LAEWATRRLRDGELDRIVDQKIAGTIRPDSLKKFADTAEKCLAEYGVERPSMGDVLWCLE

YALQLQVASPDSSVTTLQRSSSISSVVTDATVSANLGDLDGMSMKRVFSKMLKSEEEGRR

KMH

>Os*-*CrRLK1L8

MMVSSRFVAVLLLVALAPAARGQGGGGGNSSAPAASPPGPFVPRDNILLDCGATGQANDT

DGRLWTGDTGSKYLPANLAAAAATAQDPSVPQVPYLTARFSAAPFTYSFPVGAGRKFLRL

HFYPANYSNRNAADALFSVSIPDPNITLLSNFSAYQTALALNFDYLVREFSVNVTASTLD

LTFTPEKGHPNAFAFVNGIEVVSSPDLFGSSNPMEVTGDGSGTPFPIDAGTAMQTMYRLN

VGGNAISPSKDTGGYRSWEDDTPYIPFASFGVSYANDTNVPINYPDSIPQYVAPADVYST

ARSMGPDNNVNLQYNLTWAMQVDAGYQYLVRLHFCEIQSGISKINQRTFDIYINNQTAFS

GADVIAWSTGLGIPVYKDFVVFPMGSGPMDLWVDLHPNVKNKPQYYNAILNGMEVFKLQL

TNGSLAGLNPVPSIVPTASGGNSGKKSSVGPIIGGVIGGLVVLALGCCCFFVICKRRQRA

GKDSGMSDGHSGWLPLSLYGNSHTSSSAKSHTTGSHASSLPSNLCRHFSFVEIKAATNNF

DESLLLGVGGFGKVYRGEIDGGATKVAIKRGNPLSEQGVHEFQTEIEMLSKLRHRHLVSL

IGYCEEKNEMILVYDYMAHGTLREHLYKTQNAPLSWRQRLDICIGAARGLHYLHTGAKHT

IIHRDVKTTNILLDEKWVAKVSDFGLSKTGPTMDHTHVSTVVKGSFGYLDPEYFRRQQLT

DKSDVYSFGVVLFEVLCARPALNPTLAKEEVSLAEWALHCQKKGILDQIVDPHLKGKIAP

QCFKKFAETAEKCVSDQGIDRPSMGDVLWNLEFALQMQESAEESGSLGCGMSDDSTPLVI

VGKKDPNDPSIESSTTTTTTTSISMGEQSVASIDSDGLTPSAVFSQIMNPKGR

>Os*-*CrRLK1L9

MRLLALAVASIVLANLHLLGVHGRDLLLSCGSNATVDAGGRRWIGDMAPGLNFTLSSPGI

AASQAGSSNGNEIFGLVYHSARFFSTASWYNFSVLPGNYCLRLHFFPYTFGNFSGNDSLF

DVTANDFKLVSKFNVSEEIVWRSTVSNSAINAVVKEYFLLVGSRGLQVEFDPSPGSFAFV

NAIEVMLTPDNLFNDTVNKVGSAGNGQLPLGLSNRGLETMYRLNVGGHALNSSSDQYLHR

PWYTDEAFMFSANAAQIVSNTSSVSYLSNNDSSISPIDVYETARIMSNNMVVDKRFNVSW

RFYVHPNFDYLVRLHFCELFYDKPNQRVFKIYINNKTAAEDYDVYVRAGGINKAYHEDYF

DNLPQQVDSLWLQLGPDSLTSASGTDPLLNGLEIFKLSRNGNLAYVLGHIDMGNQRGISK

DRNRKILWEEVGIGSASFVTLTSVVLFAWCYVRRKRKADEKEAPPGWHPLVLHEAMKSTT

DARAAGKSPLTRNSSSIGHRMGRRFSISEIRAATKNFDEALLIGTGGFGKVYKGEVDEGT

TVAIKRANPLCGQGLKEFETEIEMLSKLRHRHLVAMIGYCEEQKEMILVYEYMAKGTLRS

HLYGSDLPPLTWKQRVDACIGAARGLHYLHTGADRGIIHRDVKTTNILLDENFVAKIADF

GLSKTGPTLDQTHVSTAVKGSFGYLDPEYFRRQQLTQKSDVYSFGVVLFEVACGRPVIDP

TLPKDQINLAEWAMRWQRQRSLDAIVDPRLDGDFSSESLKKFGEIAEKCLADDGRSRPSM

GEVLWHLEYVLQLHEAYKRNNVDCESFGSSELGFADMSFSLPHIREGEEEHHSKPSSIRE

DPDT

>Os*-*CrRLK1L10

MDSTFRKLKLVLALVGIITWIIGTCNAKFTPADNYLVNCGSTVDATVGQRVFVADNSQSI

VLTTPQSQSIAARTTLNSVSGFDNAELFQTARIFTAPSSYSFKMRSSGRHFVRLYFFPFL

YQSYDLASSKFKVSTEDVVLIDNFPQPSNSISVVMEYSLNITRDRLILTFVPEGNSTSFV

NAIEVVSVPDDLITDSAQLLGVGQYLGLAAQPLQTFHRINVGGPKVTAENDTLARTWFAD

QSFFRNPTVAQAVTYQERLNYKDGSATQDDAPDSVYNTARRLVGQRNASSTPNMTWEFNV

DGRSSYLIRFHFCDIVSKAAFQLYFDVYVYNFSAAKDLDLSAREFGTLAAPFYMDIVLPS

SDPSGNLTVSIGPSSLPNATPDGILNGLEIMKMNFSSGSVYVVKPPSAAKQQLPIILGSV

LGGIGAAIIVVVLCVVFRRKKKMKKPQTPLTSRPSSSWTPLSLNALSFLSTGTRTTSRTT

YTSGTNSDTSYRIPFVVLQEATNHFDEQMVIGVGGFGKVYKAVLQDSTKVAVKRGNQKSH

QGIREFRTEIELLSGLRHRHLVSLIGYCDERNEMILVYEYMEKGTLKGHLYGGDQPPLSW

KKRLEICIGAARGLHYLHTGFAKSIIHRDVKSANILLDENLMAKVSDFGLSKTGPEFDQT

HVSTAVKGSFGYLDPEYYRRQKLTDKSDVYSFGVVLLEVICARPVIDPTLPRDMINLAEW

AIKWQKRGELDQIIDKRIAGTIRPESLRKYGETVEKCLAEYGVERPTMGDVLWNLEFVLQ

LQEAGPDMSNIDSMNQISELPSNAQRISSLEISTADESRTAMDYSQMSTSNAFSQLINTE

GR

>Os*-*CrRLK1L11

MASRRHVLIAALIMVGVLEFANADKYKPTESILVNCGSDKEGQDIDGRKWLSDKDSKWLI

DGEKSSIMANADFQDPSLPSPVPYMTARVFTKETMYNFSVGEERHWVRLHFYPASYHDLP

AENFFFSVSTSTGITLLKNFSVYITAKALSQAYIIREFTLPPSTTGSLSLIFTPTAMNNA

SYAFVNGIEIISMPNIFSQAAASVDIAGNEVSTTDSSLQTIYRLNVGGSYVAPTNDSGLS

RDWYDDTPYIYGAAVGVTYQANDTVQIKYPKNDPDAEYAAPASVYLTSRSMGPDPKVNKN

YKLTWVFEVDGNFTYIVRLHFCELLLSKPNQRVFDILINNKTAQSGADVIGWGGQFVPVY

KDYATIMPGGAGDKVLWVQLMPNVGSGSEFFDSLLNGLEIFKMSDSSGNLAGPNPDPSKL

LEEAESSAQGKFKSKPSNLKATVIGGAAGGAAAFGIVAAICIVVYQSKKRKVLNNSASHS

SGWLPVYGGNSHTSTSKSSGGRSAALINPNITAMCRHFSFGEIKSATKNFDESLVIGVGG

FGKVYRGVVDGDTKVAIKRSNPSSEQGVLEFQTEIEMLSKLRHKHLVSLIGCCEDEGEMI

LVYDYMAHGTLREHLYKGGKPALSWKQRLEITIGAARGLHYLHTGAKYTIIHRDVKTTNI

LVDEKWVAKVSDFGLSKTGPTAMNQTHVSTMVKGSFGYLDPEYFRRQQLTEKSDVYSFGV

VLFEVLCARPALNPSLPREQVSLADHAMSCQRKGTLHDIIDPLLNGKIAPDCLKKFAETA

EKCLADHGVDRPSMGDVLWNLEFALQMQETFENGGKTEGADSTSDSTTTSVADSMAANAA

ALSLISEDMDEEDIANSVVFSQLVRPTGR

>Os*-*CrRLK1L12

MIHPSLLTTIQWVALSTLILITIAADNYSSSSSPIFLNCGASTMQLDINNRSWEGDTRSK

FASAMNGIAASATYQDPSLPSLVPYMTSRIFISNYTYSFPISPGRIFVRLYFYPVAYGYY

ASEDAYFGVKTNNLILLDNFNASQTAQAANYAYILREFSLNVTLGSLDLTFFPSTQNGSY

AFVNGIEIVPTPDIFTTRTPTHNTEGNLDPSDIDSMTSFQTMYRLNVGGQAIIPQGDSRF

YRSWEDDSPYIYGAAFGVTFGKDSNVTITYPGTMPNYTAPADVYATARSMGPNWQINLNY

NLTWILSVDAGFYYLLRFHFCEIQYPITKMNQRSFFIYINNQTVQDQMDVIRWSGGIGMA

TYADYLIVTVGSGQMDLWVALHPDLSSRPQYYDAILNGLEVFKLWDIGKKNLAGLNPPLP

PQPKTDVNPKGVSGGGKLKAAVPAAICAVVVLITACFCVCIICRRKKVAKHSGKTDKKCL

TYQTELYKSPSNLCRNFTFHEMQIATSSFDETLLLGRGGFGDVYRGEIDNGTTVAIKRSN

PLSLQGVHEFQTEIETLSKVRHGHLVSLIGYCQEKNEMILVYEYMARGTLREHLYSTKRP

PLPWKERLKICIGAARGLYYLHTGPKETIIHRDVKTANILLDDKWVAKVSDFGLSKVNPD

IDATHVSTVVKGTFGYFDPEYFRLKQLTQRSDVFSFGVVLFEILCARPPVNTELPEEQVS

LREWALSCKKIGTLGEIIDPYLQGEIAPDCLKKFADCAEQCVADRSIDRPEMGDVLRNLE

VALKMQECAENNSKFSEETTSSKTTPDMMTIMDTDKQSTYSTMSITGQRTIFSDMMDPQA

R

>Os*-*CrRLK1L13

MRRRGRMIPPALLLAAAVAAALATAVSGQGRPVTESGAQTAPTPSTFTPKDNFLIDCGST

SPVTTGGKVYKTDAQSNSLLSAKDAIKVATTDADVPSPLYLTARIFRDEAVYSFPLTVPG

WHFVRLYLFPLKNSDFDLTTATFTVSTDTNVLLHSFTAENKPVMKEFLVNATENHLAVKF

YPLKGSAAFINAIEVVNAPDELITDMAMGIAPVGEMTGLAEAAYQVVYRINVGGPAIAPD

KDTLGRQWDVDAPYVQSKEAVKDVSVPVGNIKFPDGTSKLVAPAQVYASCAKMADAGVGS

PSFNMSWKMEVDPAFGYLVRLFFADIVSKSMNDLYFNVFVNGRKAISGLDLSTVTGELSA

AYYKDIVVNSSIATDKLSIQVGPMGEDTGRVDALLSGVEVLKMSNSVGSLDGEFGVDGKK

ADDGSGSRKAVAAVGFAMMFGAFAGLGAMAVKWYKRPQDWERRNSFSSWLLPIHTGQSFT

TSKGGSSKSGYTFSSTLGLGRFFSFAEIQAATKNFEESAIIGVGGFGNVYIGEIDDGTKV

AVKRGNPQSEQGINEFNTEIQMLSKLRHRHLVSLIGYCDENAEMILVYEYMHNGPFRDHI

YGKDLPALTWKQRLEICIGAARGLHYLHTGTAQGIIHRDVKTTNILLDDNFVAKVSDFGL

SKDGPGMNQLHVSTAVKGSFGYLDPEYFRCQQLTDKSDVYSFGVVLLETLCARPPIDPQL

PREQVSLAEWGMQWKRKGLIEKIMDPKLAGTVNQESLNKFAEAAEKCLAEFGSDRISMGD

VLWNLEYALQLQDANPPEGADKPADHDGAGAAPATSSGSGVSTVPDVSTTAAGEMFAQLA

DMKGR

>Os*-*CrRLK1L14

MPAARRSGGRLTEEVNMMVALSGRKRRLQAATMVALCFLSSICVSTAQFKPADNYLVDCGSSKSTTLGTRTFAADGAAPVKVDTSLEILAGTSANGVASFDNSALYQTARIFTSPSSYTFPIQKQGRHFVRLYFFAFAYQSYDLSTAKFTVSTQEMLLLSDFQQPDKTAPLFKEYSLNITQDKLIISFKPSNGIAFINAIEVVSVPDDLIGDSAPMVNPMQQYSGLSTQPLETVYRVNMGGPKVTADNDTLSRTWVTDKKYLVNPSVTREVNGGKVNYMKGGGSTPLIAPDIVYSTATELAASNTTNALFNMTWQFDVDSGFSYLIRFHFCDIVSKALNQLYFNAYVGSFYAQHDIDLSIQSMNQLATAIYLDVVLSSNDASNKLSISIGPSTLNNALPDGILNGLEVMKMSSGSGSAFTVGSSGSNKNLGVIIGSVLGAVGILIIVLVIVLLCRKKKTLEKQHSKTWMPFSINGLTSLSTGSRTSYGTTLTSGLNGSYGYRFAFSVLQEATNNFDENWVIGVGGFGKVYKGVLRDDTKVAVKRGNPKSQQGLNEFRTEIELLSRLRHRHLVSLIGYCDERNEMILVYEYMEKGTLKSHLYGSDNPSLNWKQRLEICIGAARGLHYLHTGSAKAIIHRDVKSANILLDENLLAKVADFGLSKTGPELDQTHVSTAVKGSFGYLDPEYFRRQQLTEKSDVYSFGVVLLEVLCARPVIDPTLPREMVNLAEWGMKWQKRGELHQIVDQRVSGSIRPDSLRKFGETVEKCLADYGVERPSMGDVLWNLEYVLQLQDADSSTVSDVNSMNRIVELPSQVQNIGALESISVTMAEAGASHEPDHDLSDVSMSRVFSQLIKAEGR

>Os*-*CrRLK1L15

MKMESFAWLLLILVLFSILEDVRGSKSKPILINCGSDSTTDVDGRRWIGDSSPKNFTLSL

PGTVATAPDSDGKETYGDLYKNARIFNASSSYKFIVAAAGSYFLRLHFSQLPTNFSTKES

LFDVSANGLKLVSKFNVPAEIYLRNSKINSTSRAIVKEYLLNVTSSNLEIEFSPDAESFA

FINAMEIVPVSGNSVFDSVNKVGGYGLKGPFSLGDSAVETMYRICVGCGKIESKEDPGLW

RKWDSDENFIFSMSAARAISNSSNISYVSSDDSTSAPLRLYETARVTTESSVMDKKFNVS

WSFNVDPDFDYLVRLHFCELEYDKAEQRKFKIYINNKTAAENYDVFAKAGGKNKAFHEDF

LDAASPQMDTLWVQLGSESSAGPAATDALLNGMEIFKVSRNGNLAHPTVRIGGFNSAMGK

PKRSPKWVLIGAAAGLVIFVSIVGVIFVCFYLRWKKKTSANKTKDNPPGWRPLVLHGATT

PAANSRSPTLRAAGTFGSNRMGRQFTVAEIREATMNFDDSLVIGVGGFGKVYKGEMEDGK

LVAIKRGHPESQQGVKEFETEIEILSRLRHRHLVSLIGYCDEQNEMILVYEHMANGTLRS

HLYGTDLPALTWKQRLEICIGAARGLHYLHTGLDRGIIHRDVKTTNILLDDNFVAKMADF

GISKDGPPLDHTHVSTAVKGSFGYLDPEYYRRQQLTQSSDVYSFGVVLFEVLCARPVINP

ALPRDQINLAEWALKWQKQKLLETIIDPRLEGNYTLESIRKFSEIAEKCLADEGRSRPSI

GEVLWHLESALQLHQGLLQSANTDDLSQPELKLSDASCNLGCIEEVEESCRAGSQDVNEE

YVDVKIEVP

>Os*-*CrRLK1L16

MAAIVLLLFLVVGLMPVSNGQTTPFSPRFSVYLACGAGGNVVVTSDSPQRTFVPDDGELS

GKSARFSNPDASPPSPLYAAARAGTSGFSYRLSYAADAAPDGNTTLVLRLHFFPFASQSG

DLLSARFSVSAMGRYVLLPPSFSPPRAGVVREFLLPSDGSGEFDVAFTPESGGLAFVNAI

ELFPAPQELLWKFPLTAVNTDVSPSHQALETLYRLNVGGPTVTPTGDTMWRTWLPDDSYL

SPATVSAVASIQGQIIFDRAQGYTQMVAPDAVYKSQRTTNSTTSNVTWTFAVDGNSSYVV

RLHFCAFEELSSVIGEGVDFNVYLMQAMGTRELKAKDYATLSSPTQAFYMDYVAVVPTAG

ENLTVSIGRAASSDSKKAILNGLEIMKLRAVDMTPASSSGKTSKVVVVAVTAAVLGAAVL

AGVALCVLLVRRRQRRATLPVPEEEEKESVGTPWSPFTPDGEGSFGSAVVTPRRMNMKLH

IPLAEIMVATGDFDDANILGVGGFGNVYRGVLRDGTRVAVKRAKRASRQGFPEFQTEILV

LSSIRHRHLVSLIGYCNERSEMILVYELMAHGTLRSHLYGSDAAAATPPPLSWKQRLEIC

IGAAKGLHYLHTGHSDNIIHRDVKSTNILLGDGFVAKVADFGLSRVGPSTGQTHVSTAVK

GSFGYLDPEYFKTRQLTDRSDVYSFGVVLFEVLCARPAIDQSLPPDEINLAEWAMQWSRR

GRFDKIVDPAVAGDASTNSLRKFAETAGRCLADYGEQRPSMGDVVWNLEYCLQLQESQPS

TETALDLDDSGAHLPRDIVVARRVAPLAPDASADAAGDDMSWSETASFTATGNVFSQIMS

RDGR

>Bd*-*CrRLK1L1(KQK13083)

MPTFAILAESMVEWKRVPMFLIIFILSITGVATTNAIASKTDRFVPQDNYLLSCGASAAV

PLDDGRTFRSDPDSVSFLSTPTDIKIAAKASLASASPLSPLYLTARVFSDISTYSFFISQ

PGRHWIRLYFSPIPESQYNLTTATFSVSTDNMVLLHDFSFIASPPTPILREYLVAVQGDN

LKIVFTPKKNSVAFVNAIEVVSVPPSLIPNTTTRMGPQDQFDISNNALQVIYRLNMGGAL

VTSFNDTLGRTWLPDAPFLKIEAAAEAAWVPPRTIKYPDDKTITPLIAPANIYSTAQKMA

SANITDARFNITWEMAADPGFRYLIRLHFSDIISKTLNSLYFNVYINGMMGVSNLDLSSL

TMGLAVAYYKDFIADSSSIINSTLVVQVGPSTTDSGNPNAILNGLEIMKISNEASSLDGL

FSPKTSSQVSKRTLTGIGLALVVTAALAVVICCRRSHRPEWQKTNSFHSWFLPLNSSHSS

FMSSCSRLSRNRFGSTRTKSGFSSIFASSAYGLGRYFTFAEIQKATKNFEEKGVIGVGGF

GKVYLGSIEDGTKLAIKRGNPSSDQGMNEFLTEIQMLSKLRHRHLVSLIGCCDENNEMIL

VYEYMSNGPLRDHLYGDTNIKPLSWKQRLEVSIGAAKGLHYLHTGAAQGIIHRDVKTTNI

LLDENFVAKVADFGLSKAAPSLEQTHVSTAVKGSFGYLDPEYFRRQQLTEKSDVYSFGVV

LFEVLCARPAINPALPRDQVNLAEWARSWHRKGELNKIIDPHIAGQIRPDSLEMFAEAAE

KCLADYGVDRPSMGDVLWKLEFALQLQEKGDVVEGSNDGIPMKSLEMSNVDNMEKSANVI

PSYVQGR

>Bd*-*CrRLK1L2(KQK06923)

MGFPILLVIILTLLSLILLVVVADNNSTASGHVALNCGASGQNNDDNGRTWDGDTSSKFA

PLVKGVTAPASYQHPSLPSTVPFMTARIFTSNYTYSFPVSAGRMFVRLYFYPIAYGNYAV

SDAFFSVTTRNLSLLNDFNASQTAQAINVAYLVREFSLNVSSGSLDLTFAPSKHWNGSYA

FVNGIEIVPTPDIFTTADTRFVNGGTPAPFQINTDRGFQTMHRLNVGGQAIPPKDDLSFY

RSWANDSPYIFGGSGVAFSRDNNLTIKYTSTVPNYTAPIGVYGTARSMGTNAQVNLNYNL

TWILPVDAGFFYLLRFHFCEIQYPITKVNQRSFFIYINNQTAQRQMDVIAWSGGIGRTAY

TDYVIITTGSGQVDMWVALYPDLSSKPEYYDAILNGLEVFKLQDYGKNNLAGLNPPLPQK

PDVNPNGPSREGNSRGTVLAAICGAIGGFAVLLICFGVCIACRRNKKISKDSDKSDDGCW

TPLADYSRSRSGNSGNTATTGSHASLPSNLCRHFSFAEVQAATNNFDQAFLLGKGGFGNV

YLGEIDSGTKLAIKRCNPMSEQGVHEFQTEIEMLSKLRHRHLVSLIGYCEDKNEMILVYD

YMAHGTLREHLYKTKNPPLSWKQRLEICIGAARGLHYLHTGVKQTIIHRDVKTTNILLDD

KWVAKVSDFGLSKTGPNVDNTHVSTVVKGSFGYLDPEYFRRQQLSEKSDVYSFGVVLFEV

LCARPALSPSLPKEQVNLADWALHCQKKGILGQIIDPLLQGKISPQCFVKFAETAEKCVA

DHSIDRPSMSDVLWNLEFVLQLQESAEDNSSLTGGMSSSDVSSPLVPTRLQSDEPSTETT

TTTTASTMSITQRSIASAESAGLTPSTIFSQLMNPDGR

>Bd*-*CrRLK1L3(KQK21820)

MGRSPRCVLLLLLAACAAALVPAAGAQGGGNSTAPAPAAAGSAPFVPRDDILLDCGATGN

GNDTDGREWGGDAGSKYAPANLGSAIAGAQDPSVPQVPYLTARVSAAPFTYSFPLGPGRK

FLRLHFYPANYSNRNAADAFFSVTVPAAKVTLLSNFSAYQTSTALNFAYLIREFSVNVTG

QTLDLTFTPEKGHPNAYAFINGIEVVSSPDLFDLSTPELVMGDGNNQPYTMEAGTALQTM

YRLNVGGQAISPSKDTGGYRSWDDDTPYIWGAGAGVSYQNDANVTITYPDNVPGYVAPTD

VYATARSMGPDKDVNLAYNLTWIMQVDAGFFYLVRLHFCEIQSPITKPNQRVFDIYINNQ

TAMAGADVILWASPNGIGSPVYKDYVVNTMGSGTMDFWVALHPDVTQKPQYFDAILNGME

VFKLQQSNGSLVGLNPVPSAEPLVDGGSGKKKSTVGPIVGGVVGGLAVLALGYCFIVICK

RRRRAGKDAGMSDGHSGWLPLSLYGNSHTSGSAKSHTTGSYASSLPSNLCRHFSFAEIKA

ATKNFDESLILGVGGFGKVYRGEVDGGTTKVAIKRGNPLSEQGIHEFQTEIEMLSKLRHR

HLVSLIGYCEEKNEMILVYDYMAHGTLREHLYKTQNAPLSWRQRLEICIGAARGLHYLHT

GAKHTIIHRDVKTTNILLDEKWVAKVSDFGLSKTGPSMDHTHVSTVVKGSFGYLDPEYFR

RQQLTEKSDVYSFGVVLFEVLCARPALNPTLAKEEVSLAEWALHCQKKGILDQIVDPYLK

GKIVPQCFKKFAETAEKCVADNGIERPSMGDVLWNLEFALQMQESAEESGSIGCGMSDEG

TPLVMVGKKDPNDPSIDSSTTTTTTTSISMGDQSVASIDSDGLTPSAVFSQIMNPKGR

>Bd*-*CrRLK1L4(KQK23907)

MAVVLLALLLLAATLLLAPSPAALAAFSPSFECFLACGAADNLSFPSDSPARVFVPDAAF

LSPATTPALASSQASSSALYAAARGSSSEFSYSIPCSSPATFLVLRLHFFPLPATPSSSA

RFAVSVRHGAYALTLLQSFSPPPAGVVKEFFLPDAGSNGELRVTFAPASGSSAFVNALEL

FPAPPELLWNENSPYPYTPVGTAADNNATATWPQQALETLHRLNVGGPTVNSTLDTLWRT

WLPDDAFLYGGIAQTTVGSSITPVFDPDNGYTREVAPDVVYKTQRFANVTDYMLATNPGI

SFNVTWTFPAVPGSGGYLVRLHFCDYDMVSSVVGVGIVFDVYVAQAVAARDLKLAELGKR

VPSQAFYFDYAAMAPSAGNLTVSIGKARSTGGMILNGLEIMKLLPLSAVSSHEGMPKRTI

VIAALASVLGAAVLACSVLCLVVLMRRRKRRMRPAPEKASTTMPPWSPFRGGSSWVVDQS

TDHSGEGTGMQRVISTKLHISLSEIRAATEGFHERNLIGVGGFGNVYKGALHDGTPVAVK

RAMRASKQGLPEFQTEIVVLSGIRHRHLVSLIGYCDDQAEMILVYEYMEHGTLRSHLYGF

DDDDDNSEPLSWKQRLEICIGAARGLHYLHTGYSENIIHRDIKSTNILLGSEDGVLVAKV

ADFGLSRIGPSFGETHVSTAVKGSFGYLDPEYFKTQQLTDRSDVYSFGVVLFEMLCARPV

IDQSLDRDQINIAEWAVRMHGQGQLGKIVDPRMAMAAGGVDENSLRKFAETAEKCLADYG

VDRPSMGDVLWNLEYCLQLQETHVSRDAFEDSGAVTATRLPAGVVVPRWVPASTMDDVDD

TGMSIGMSVVADSKVFSQLSAGGEGR

>Bd*-*CrRLK1L5(KQK22263)

MPPHLDLLLLWLLVVSAPVLASAATPSTAFVPADNYLVICGTSGSATDTAGRTFVGDGRL

PASALAAPQSVEANASLSSSNGDEQALYQSARIFTAPASYTFAIKKPGRHFVRLHFFPFR

YQSYDLAAAAAFKVFVQGAVFVDGSYTPKNGTVVVKEFSVNVTGGSLVIAFTPTGKLAFV

NAIEVVSLPDDLIADTAAMAGSARGLYTGLSARALETVHRINMGAPKITPANDTLWRTWL

PDQSFQLDSSLALAEHKEVLPSAIKYTPVATPWTAPVGVYATATKQSTSGGTSTINVQFN

VTWRFGAVAAGSDYLLRFHFCDIVSKAATGLAFNVYVGAWLVLDNYEYSRDTINTLAVPV

YKDFVLGAKDVKGGNITVSIGSSTVGVSNVSPDGFLNGLEIMRVLGSAGAGAEPSKRSSK

VKTWIIAGSAVGGAAVAMALAFIAFRMLCRKRGKPEKKASNSTLSPFSASALGSRSRSSG

KKSNGNTIVLGQNGLGAGYRIPLAVLQEATSGFGEAMVIGEGGFGKVYKGTLPDETPVAV

KRGSRKTLQAMQEFRTEIEMLSRMRHRHLVSLIGYCDARDEMILVYEYMAMGTLRSHLYG

ADDLPPLTWEQRLEACIGAARGLHYLHTSSATAVIHRDVKSSNILLDETLMAKVADFGLS

KAGPELDKTHVSTKVKGSFGYLDPEYFRRQMLTEKSDVYSFGVVLLEVLCARAVIDPTLP

REMVNLAEWAMQWLKKGEVDRIVDQRIAGTIRPQSLKKLADTAEKCLAEYGVERPTMGDV

LWCLEFALQLQVASPDDSVIDGMPLAPVATPQVQRIQSIASVATDTAMTANLGDLDGMSM

SGVFSKMVKSEEVR

>Bd*-*CrRLK1L6(PNT61865)

MFQMRLLVLAAVSIVFANLQFLKAHGRELLLSCGSNATVDADGRRWVGDMAPDLNFTLSS

PGIAALLAGGSNASEIFGPVYRSARLFTTTSWYDFSVLPGNYCIRLHFFPSTFGNFSANS

SVFDVVANDFKLVSKFNVSEEILWRSSVSNLAVTAIVKEYFLAVSTQRLQIEFDPSRGSF

AFVNAIEVMLTPDNSFNDTVHKVGGGDGYLPPGLSSRGVETMYRLNIGGPALASSSDQYL

HRPWYTDEAFMFSANAALTVSNTSAIRYLSSNDSSIAPIGVYETARIMSNNMVVDKRFNV

TWRFFVHPNFDYLVRLHFCELVYDKPSQRIFKIYINNKTAAENYDVYVRAGGINKAYHED

YFDSLPQQVDSLWIQLGPDSMTSASGTDALLNGLEIFKLSRNGELDYVLGHIDMGNQRGP

SKGKRKINIWEEVGIGSASFVMLASVALFSWCYVRRKRKAAEKEAPPGWHPLVLHEAMKS

TTDARASSKSPLARNSSSIGHRMGRRFSISDIRSATKNFDETLVIGSGGFGKVYKGEVDE

GTTVAIKRANPLCGQGLKEFETEIEMLSKLRHRHLVAMIGYCEEQKEMILIYEYMAKGTL

RSHLYGSDLPPLTWKQRLDACIGAARGLHYLHTGADRGIIHRDVKTTNILLDKNFVAKIA

DFGLSKTGPTLDQTHVSTAIRGSFGYLDPEYFRRQQLTQKSDVYSFGVVLFEVACARPVI

DPTLPKDQINLAEWAMRWQRQRSLEAIMDPRLDGDYSPESLKKFGDIAEKCLADDGRTRP

SMGEVLWHLEYVLQLHEAYKRNLDCESFGSSELGFADMSFSMPHIREGEEERQSKQSGIR

EDSDT

>Bd*-*CrRLK1L7(KQK07346)

MGTATNERKLAVVFLWSISVLSGTCNAEFTPADNYLINCGSTVDASVGRRVFEADNSKST

ILTSHQSVAANTFLDSVPASDYAVLYQTARIFGVPSSYSFKMKSRGRHFVRLHFFSFKYQ

SYDLAVAKFKVSTQDAVLLDNFTPPSNSSPVVREYSLNITRGMLILTFVPLGNSTSFINA

IEVISVPDDLILDLGQSVNPIRQYAGLAAQPFQTFYRINVGGRKVTADNDTLWRSWDTDQ

SFFLNSTTTQTVTYEGKLNYQRGAATEEDAPDSVYNTARRLVAQNNTASASNMTWQFNVD

RRASYLIRFHLCDIVSKAMAALYFDVYVDRWSAAEDLDLSEKGFGTLAVPYYTDVILESS

DPSGKLSVSIGPSSLNNVAQDGILNGLEIMKMNISTGTVEIVQPPPSQKRELPIILGSIL

AVCAATAVAILCFVLRRKKNKKPQTASTSRTSSAWTPLTLNGISFLSTGTRTTSRTTLTS

GTNGDATYQIPFVVLQEATNHFDEQMIIGVGGFGKVYKAVLQDGTKVAVKRGNHKSHQGI

KEFRTEIELLSGLRHRHLVSLIGYCNEHNEMILVYEYMEKGTLKGHLYGSDIPALSWKKR

VEICIGAARGLHYLHTGFAKSIIHRDVKSANILLDENLMAKVSDFGLSKTGPELDQTHVS

TAVKGSFGYLDPEYYRRQKLTDKSDVYSFGVVLLEVICARPVIDPSLPREMINLAEWASK

WQKRGELDQIVDQRIAGTIRPESLRKYGETVEKCLAEYGVDRPTMGDVLWNLEFVLQLQE

SGPDITNIDSMNQISELPSEARRVGSLEISTADESHTNINYSQMSTNDAFSQLMNTEGR

>Bd*-*CrRLK1L8(KQK06928)

MRARQHLFLAELLLVISWVLLFCDAEKYEPTETILVDCGSEKDGQDAQGRKWVMDKDSKW

LGDGGKSSMMAAADAQDPSLPSPVPYMSARVFTKEAVYTFPVADADRHWVRLHFYPAAYH

DLPAEQFFFSVSTASGITLLRNFSVYITVKALSQAYIVREFTLPPSTTGSISLKFTPTAM

NNASYAFVNGIEIISMPNIFAEPATLVGLDSQTVDLAAGSLQTMYRLNVGGAYVASTNDS

GLSREWFDDTPYIYGAATGVTFEPNDTFPIKYPSPEGEFAAPADVYITSRSMGPDGRVNK

NNNLTWVFEVDANFTYVLRLHFCGLRVDKVNQVVFDIYINNKTAQDNADIIGWSSAKDVP

VFKDYAVFMPDMPGDKILWLALHPDVDSKPQFFDAILNGLEIFKMSDGSGNLAGPNPDPS

KMLMESEVEQGKFRAKPSNLQATLIGGAAGGAAALGIVAAICLVVYQTKKNRALSSSPSH

SSGWLPVYGGNSHTNASSGSRSAALNPNITAMCRHFSFPEIKSATKNFDEGLVIGVGGFG

KVYKGVVDGDTKVAIKRSNPSSEQGVMEFQTEIEMLSKLRHKHLVSLIGCCEDDGEMILV

YDYMAHGTLREHLYKSGKPPLLWKQRLEIVIGAARGLHYLHTGAKYTIIHRDVKTTNILV

DEKWVAKVSDFGLSKTGPTAQNQSHVSTMVKGSFGYLDPEYFRRQQLTEKSDVYSFGVVL

FEVLCARPALNPSLPREQVSLADHALSCQRKGTLQDIVDPLLKGKIAPDCMKKFAETAEK

CLADHGVDRPSMGDVLWNLEFALQMQETFENGGKPEGGDSVGSSSSGSTPPSMADSMAAN

AAALSLICEDMDEEDIANSVVLSQLVRPTGR

>Bd*-*CrRLK1L9(KQK19638)

MRRGAVPLALALATLAMLAVSVSGQGRPVIDTSGGMTEALPSKFTPKDAFFIDCGGTNPV

TVEGKAFKTDAQANQLLAAQDAIRASVDKADSVSSPVYLTARIFKEEAVYNFPLAVPGWH

FIRLYFFPLKNPDSDLAAATFSVTTDTNVLLHSFTADPKPTMKEYLINATENHLEIKFTP

LKGSAAFINGIEVVNGPDELITDTALAVLPFAEMSGLSEAAYQVIYRLNVGGPGISPGND

TLGRQWDNDEKYVQSKEMVKDVSVPTNTIKYPDTFPVSKLVAPMLVFASAAKMADMDSTV

SNANFNVTWKLDVDPSFDYFVRLFFADIISKSANDLYFNVYIDGRKAISGLDLSGITGDL

AVPYYKDFVVNSSITADGHLSIQIGPLGQDTGRIDALLNGAEVFKMSNSVGSLDGEFGVD

GRKADDGSGGRKVVAVVGFAMMFGAFAGLGAMVVKWHKRPQDWQRRNSFSSWLLPIHTGQ

SFSNGKGSKSGYTFSSTGGLGRFFSFAEMQEATKNFDESAIIGVGGFGNVYVGEIDDGTK

VAIKRGNPQSEQGINEFNTEIQMLSKLRHRHLVSLIGYCDENAEMILVYEYMHYGPFRDH

IYGGDGNLPALSWKQRLEICIGAARGLHYLHTGTAQGIIHRDVKTTNILLDENFVAKVAD

FGLSKDGPGMDQLHVSTAVKGSFGYLDPEYFRCQQLTDKSDVYSFGVVLLETLCARAPID

PQLPREQVSLAEWGLQWKRKGLIEKIMDPKLAGKVNEESLNKFAETAEKCLAEFGSDRIS

MGDVLWNLEYALQMQEQNPPEGAAAGEGGDGDSLDAGISSSSIATTSSSSSGINTVPDAS

TTSAGELFAQLADMKGR

>Bd*-*CrRLK1L10(KQK21184)

MPGRRRKLQVAALLLLFCVCSSAGIICRAQLVDQFKPADSYLVNCGSAKGTTVSERNFAA

DGAAPLTVSTPQEILAGTSANGVSSFDNSALYQTARIFTGPSSYTFPINKQGRHFVRLYF

FPFIYQSYDLSTAKFTVSTQDVLLLSDFQQPDKTAPLFKEYSLNITRDQLIISFKPSNGI

AFVNAIEVVSVPDDLIADVAQMVNPVQQYSGLSTQSLETVYRVNMGGPKVTPNNDTLSRT

WLNDQKFIMNPSVTKKVVYGKTIKYKNGGASSLTAPDVVYSTATELAASNTSNALFNMTW

QFDVDAGFSYLIRFHFCDIVSKALNQLYFNAYVGGFFAQHDLDLSEQSMNQLATAIYVDV

VLSSNDASSKLSISIGPSTLNNAFPDGILNGLEVMKMGSGSGSAFTVGSSGSKKILAVII

GSVIGVIGLLVIVLLLVLLCRRKKTDDKQHSKTWMPFSINGLTSLSTGSRTSYGTTLTSG

LNGSLGYRFAFNVLQEATNNFDENWVIGVGGFGKVYKGVLRDDTKVAVKRGNPKSQQGLN

EFRTEIELLSRLRHRHLVSLIGYCDERNEMILVYEYMENGTVKSHLYGSDNPSLNWKQRL

EICIGAARGLHYLHTGSAKAIIHRDVKSANILLDENFLAKVADFGLSKTGPELDQTHVST

AVKGSFGYLDPEYFRRQQLTEKSDVYSFGVVMLEVLCARPVIDPTLPREMVNLAEWGMKW

QKRGELHQIVDQRLSSTIRPDSLRKFGETVEKCLADYGVERPSMGDVLWNLEYVLQLQDA

DSSTVSDVNSMNRIVDLSSQVQHVGALESISMTMAEAGASHEPDHDLSDVSMSRVFSQLI

KAEGR

>Bd*-*CrRLK1L11(KQK21036)

MKSSTNLLLILVVFLAAENARAQPGPILINCGSDSSSAVVDGRRWIGDSSPSKNFTLSFP

GTIALAAAAPGVDGEEEPYGDLYKTARVFNSSSSYNLGVAAGSYFLRLHFSQLFANLSAE

EPIFDVAANGLKLLSRFSVTGEISWRDSQINSTSKVIVKEYLLNFTSGKLGIEFRPDEGS

FAFVNAMEVVPVSGSSIFDSVNKVGGYGLKGPFSLADGGIETMYRLCVGCSDIARKEDPG

LWRKWDSDEHFIFSLNAAHTITNSSNISYASADDSTLAPLRLYETAKVTTESSVVEKKFN

VSWSFIIDPGFDYLVRLHFCELEYDKAEQRKFKIYINNKTAAESYDVFARAGGKNKAFHE

DFLDVASPQMDTLWVQLGSESSAGAAATDALLNGMEIFKVSREGNLAHPTVRIGGISGGT

RKPKRSPKWVLIGAATGLIVFIAIVGAVYICFCLQRKKRSSANKTKNPPGCQPLALHGSA

NTRSPSLRTAGTLGSSQLGRRFTIAEIRTATQNFDESLVIGVGGFGKVYKGKMESGTLVA

IKRGHTESQQGQGVKEFETEIEMLSRLRHRHLVPLIGYCDERNEMILVYEHMANGTLRSH

LYGSDLPALTWNQRLEICIGAARGLHYLHTGLDRGIIHRDVKTTNILLNGNLVAKMADFG

ISKDGPALDHTHVSTAVKGSFGYLDPEYYRRQQLTPSSDVYSFGVVLLEVLCARPVINPT

LPRDQINLAEWALNCQRQQLLETIIDPRLDGNYTLESMKTFSKIAEKCLADEGVNRPSMG

EVLWHLESALQLHQGHLHADCDEVLSGPELTPSDASITVTHIREAEESTRAARDANDEVV

DVKIEVP
