# Supplementary material for: Genome wide characterization and expression analysis of CrRLK1L gene family in wheat unravels their roles in development and stress-specific responses
Source: Front Plant Sci. 2024 Mar 26;15:1345774. doi: 10.3389/fpls.2024.1345774 (PMC11002176; doi:10.3389/fpls.2024.1345774)
Supplement: Supplementary file 5 [file Table_3.docx]

**Supplementary Table S3A. Intron/exon lengths in *CrRLK1L* genes in *T. aestivum***

| **Gene** | **Length in base pairs** | | |
| --- | --- | --- | --- |
|  | Exon1 | Intron1 | Exon2 |
| Ta*-CrRLK1L11*-D | 450 | 124 | 1950 |
| Ta*-CrRLK1L14*-A | 36 | 1149 | 2535 |
| Ta*-CrRLK1L14*-B | 36 | 998 | 2526 |
| Ta*-CrRLK1L14*-D | 111 | 335 | 2535 |

Note: Note: Intron/Exon lengths for CrRLK1Ls were determined by comparison of NR database retrieved cDNA sequences for CrRLK1L genes and genomic DNA sequences from Ensembl Plants database.

**Supplementary Table S3B. Details of the conserved motifs predicted by MEME suite**

| Sr No. | Motif sequence | Pfam/NCBI-CDD ID | Description of the motif |
| --- | --- | --- | --- |
| 1 | QGLHEFQTEIEMLSKLRHRHLVSLIGYCDEQNEMILVYEYMAHGTLRSHL | PF07714 | Protein tyrosine and serine/threonine kinase |
| 2 | ZTHVSTAVKGSFGYLDPEYFRRQQLTEKSDVYSFGVVLFEVLCARPVIBP | PF07714 | Protein tyrosine and serine/threonine kinase |
| 3 | WKQRLEICIGAARGLHYLHTGAAKGIIHRDVKTTNILLDENLVAKVADFG | PS00108 | Serine/Threonine protein kinases active-site signature |
| 4 | IVDPRJAGKIRPESLRKFAETAEKCLADYGVDRPSMGDVLWNLEFALQLQ | 271060 | Catalytic domain of the Serine/Threonine kinase |
| 5 | RHFSFAEJQAATKNFDESLVIGVGGFGKVYKGEJDDGTKVA | cd14061 | Catalytic domain of the Serine/Threonine Kinases |
| 6 | NLNFNLTWQFDVDAGFSYLVRLHFCDIVSKA | pfam12819 | Malectin like carbohydrate-binding protein of the ER |
| 7 | LPREQVNLAEWALRWQRKGEL | ND | ND |
| 8 | PYPGLSAAALZTMYRLNVGGPAISPSNDT | ND | ND |
| 9 | GSYAFVNAIEVVSAPDELIAD | ND | ND |
| 10 | PGRHFLRLHFFPFAYSSYDLA | ND | ND |

Note: Motifs predicted by MEME suite were searched for their details at Genome Net database (https://www.genome.jp/tools/motif/). Pfam or NCBI Conserved Domain Database (CDD) ID for the IDs are given. ND denotes the motifs that did not find hits in any of the protein databases.
